# Supplementary material for: Unlocking regioselective meta-alkylation with epoxides and oxetanes via dynamic kinetic catalyst control
Source: Nat Commun. 2024 Jan 2;15:31. doi: 10.1038/s41467-023-44219-6 (PMC10761682; doi:10.1038/s41467-023-44219-6)
Supplement: Supplementary file 1 — Supplementary Information [file 41467_2023_44219_MOESM1_ESM.pdf]

Unlocking Regioselective Meta-Alkylation With Epoxides And Oxetanes Via Dynamic Kinetic Catalyst Control

Peng-Bo Bai,[1] Alastair Durie[2], Gang-Wei Wang,[1,*] and Igor Larrosa,[2,*]

1 State Key Laboratory of Applied Organic Chemistry & College of Chemistry and Chemical Engineering, Lanzhou University, Lanzhou, 730000, China

2 School of Natural Sciences, Department of Chemistry, University of Manchester, Oxford Road, Manchester M13 9PL, United Kingdom

Supplementary Information

Table of Contents

I. Supplementary Methods

1. General Information --S2
2. Preparation of Substrates and Characterization Data --------------------------------S3
3. Reaction Optimization ---S14
4. General Procedure --S18
5. Products Characterization Data --S19

II. Supplementary Discussions

1. Mechanistic Studies of Epoxide Involved *meta*-Alkylation Reaction--------------S57
2. Mechanistic Studies of Oxetane Involved *meta*-Alkylation Reaction--------------S70
3. Observations of Catalytic Intermediates ---S74

III. Supplementary Figures---S78

IV. Supplementary References--S156

I. Supplementary Methods

1. General Information

All of the alkylation reactions were carried out in an Argon-filled glove box with oven-dried crimp-cap microwave vials (10 mL). All of the starting materials and solvents were purchased from Acros (Fisher), Aldrich (Merck), Alfa Aesar (Fisher), Fluorochem, Generon, Manchester Organics, Molekula, MP Biomedicals and TCI, and used without further purification. Before transferring into glovebox, all liquid reagents and solvents were dried over 4 Å molecular sieves and degassed with 3 freeze-pump-thaw cycles. Column chromatography was carried out on silica gel, particle size 40-63 μm, using flash techniques. High resolution mass spectra were performed by Thermo Finnigan MAT95XP spectrometer. ^{1}H NMR, ^{19}F NMR and ^{13}C NMR spectra were recorded at 400 or 500 MHz on Bruker machines. ^{1}H NMR are referenced to the residual solvent peak at 7.27 ppm ($CDCl_3$) and quoted in ppm to 2 decimal places with coupling constants (*J*) to the nearest 0.1 Hz. Other abbreviations used are app (apparent), s (singlet), d (doublet), t (triplet), m (multiplet) and br (broad). ^{13}C NMR spectra, recorded at 100 MHz or 126 MHz, are referenced to the solvent peak at 77.00 ppm ($CDCl_3$) and quoted in ppm to 1 decimal place with coupling constants (*J*) to the nearest 0.1 Hz. ^{19}F NMR spectra were recorded at 376 or 471 MHz in $CDCl_3$ and quoted in ppm to 1 decimal place and with coupling constants (*J*) to the nearest 0.1 Hz.

2. Preparation of Substrates and Characterization Data

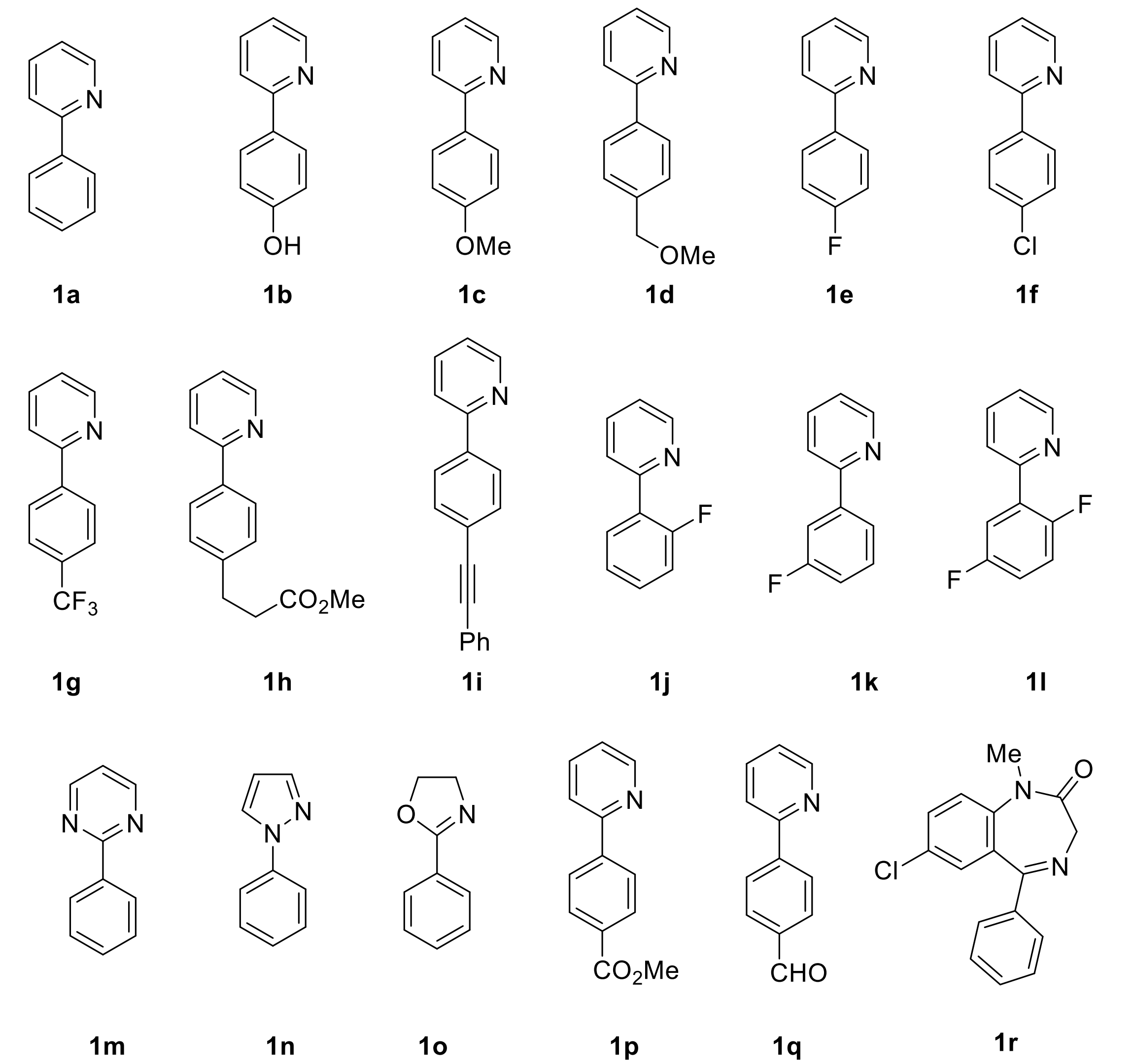

Supplementary Figure 1. Scope of 2-phenylpyridine derivatives

Compounds **1a**, **1c**, **1m**, **1n**, **1o**, **1q** and **1r** are commercially available. The following substrates were prepared according to previously described procedures: **1b**[1], **1e**[1], **1f**[1], **1g**[2], **1i**[3], **1j**[4], **1k**[1], **1l**[1], **1p**[1]. Compound **1d** and **1h** are novel compounds and were synthesized by following method:

2-(4-(Methoxymethyl)phenyl)pyridine 1d

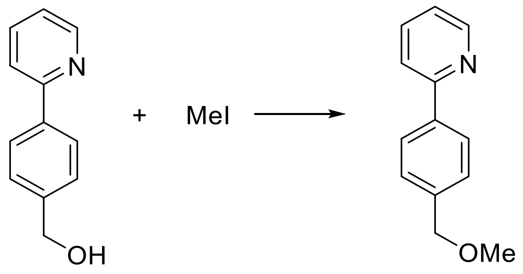

An oven dried reaction flask, fitted with a magnetic stirrer, was charged with (4-(pyridin-2-yl)phenyl)methanol (705 mg, 3.82 mmol) in THF (10 mL) under argon, and the reaction mixture was cooled to 0 °C. NaH (230 mg, 5.73 mmol) was added slowly and the reaction mixture was stirred at 0 ºC for 1 hour. Then CH_3I (0.30 mL, 4.58 mmol) was added dropwise by syringe and the reaction mixture was allowed to stir at room temperature overnight. Water (10 mL) was added slowly and the mixture was extracted with EtOAc (3 × 20 mL/mmol), the organic extracts were combined, dried over Na_2SO_4 and concentrated in *vacuo*. The residue was purified by column chromatography (20% EtOAc/Hex) to give 2-(4-(methoxymethyl)phenyl)pyridine **1d** (692 mg, 3.47 mmol, 91%) as a colorless oil.
^{1}H NMR (400 MHz, $CDCl_3$) δ 8.80 – 8.50 (m, 1H), 7.99 – 7.97 (m, 2H), 7.76 – 7.62 (m, 2H), 7.44 – 7.42 (m, 2H), 7.17 (dd, *J* = 8.6, 4.4 Hz, 1H), 4.49 (s, 2H), 3.39 (s, 3H). ^{13}C NMR (101 MHz, $CDCl_3$) δ 157.1, 149.6, 139.0, 138.7, 136.7, 128.0, 126.9, 122.0, 120.4, 74.3, 58.1. m/z (ESI$^+$) HRMS: Calculated for $[C_{13}H_{14}NO]^+$: 200.1070. Found $[M+H]^+$: 200.1069.

Methyl 3-(4-(pyridin-2-yl)phenyl)propanoate 1h

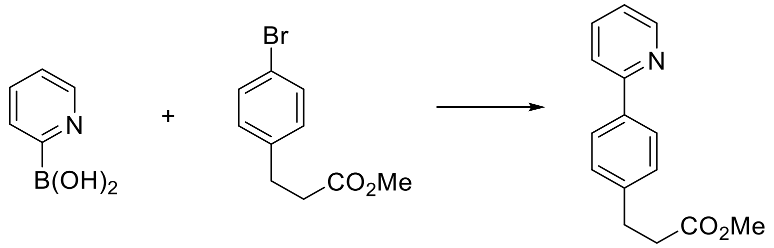

An oven dried reaction flask, fitted with a magnetic stirrer, was charged with methyl 3-(4-bromophenyl)propanoate (1.25 g, 5.0 mmol), 2-pyridineboronic acid (738 mg, 6.0 mmol), $Pd(PPh_3)_2Cl_2$ (53.0 mg, 0.075 mmol) and K_2CO_3 (1.73 g, 12.5 mol) under argon, a mixed solvent of DME/water (10 mL/2.0 mL) were added. The mixture was heated at 100 °C for

12 hours. The suspension was cooled to r.t. and water (20 mL) was added and the mixture was extracted with EtOAc (3 × 20 mL/mmol), the organic extracts were combined, dried over Na_2SO_4 and concentrated in *vacuo*. The residue was purified by column chromatography (20% EtOAc/Hex) to give methyl 3-(4-(pyridin-2-yl)phenyl)propanoate **1h** (483 mg, 2.00 mmol, 40%) as a colorless oil.

^{1}H NMR (400 MHz, CDCl3) δ 8.67 (s, 1H), 7.93 – 7.91 (m, 2H), 7.70 (s, 2H), 7.32 – 7.30 (m, 2H), 7.20 (s, 1H), 3.67 (s, 3H), 3.01 (t, *J* = 6.9 Hz, 2H), 2.67 (t, *J* = 6.9 Hz, 2H). ^{13}C NMR (101 MHz, CDCl3) δ 173.3, 157.3, 149.7, 141.5, 137.6, 136.7, 128.8, 127.1, 122.0, 120.4, 51.7, 35.6, 30.7. m/z (ESI$^+$) HRMS: Calculated for $[C_{15}H_{16}NO_2]^+$: 242.1176. Found $[M+H]^+$: 242.1181.

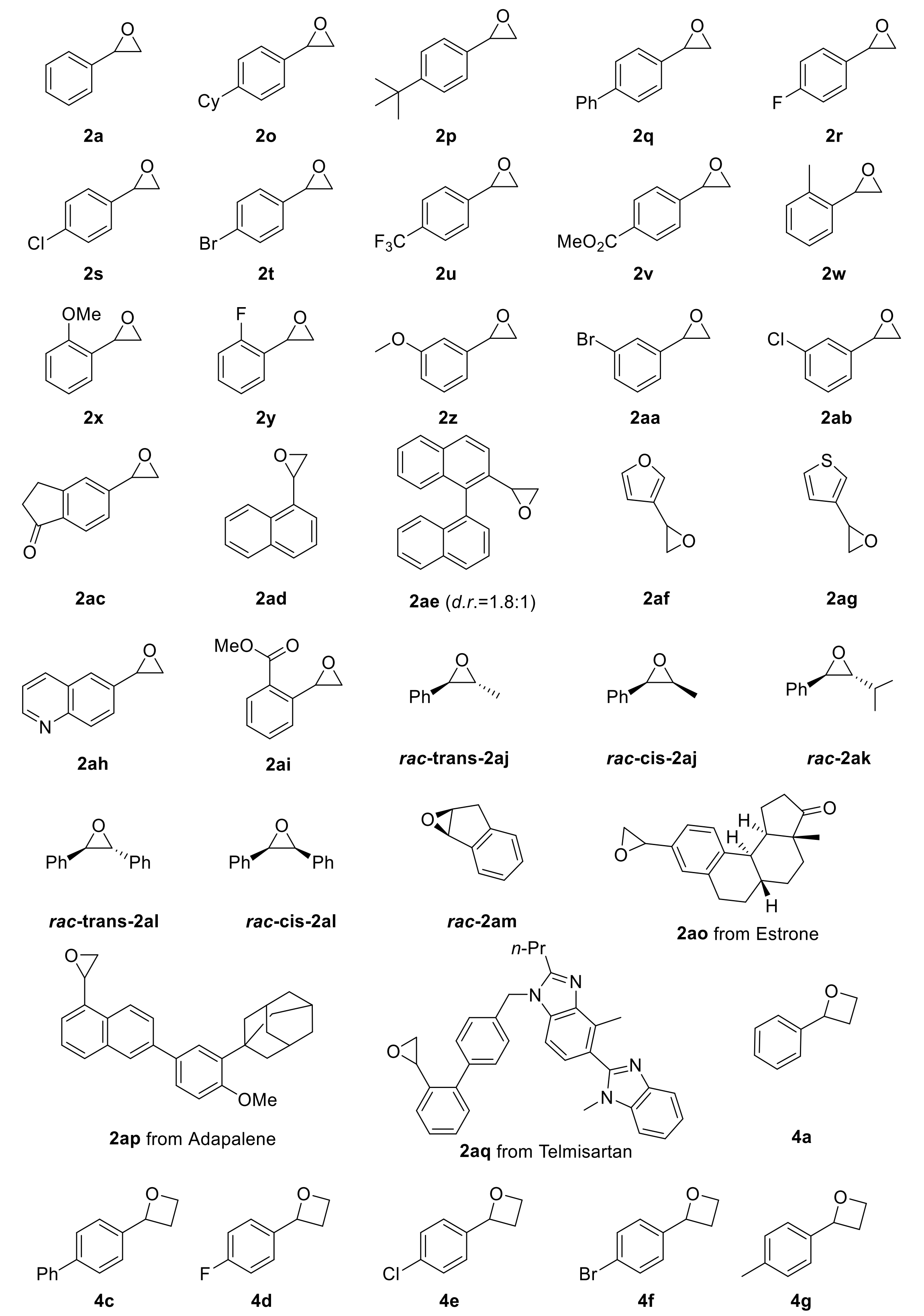

Supplementary Figure 2. Scope of aryl epoxides and oxetanes

Compound **2a** is commercially available. The following substrates were prepared according to previously described procedures: **2p**[6], **2q**[6], **2r**[7], **2s**[7], **2t**[8], **2u**[7], **2v**[7], **2w**[7], **2x**[8], **2y**[7], **2z**[7], **2aa**[8], **2ab**[7], **2ad**[9], **2ai**[10], **trans-2aj**[11], **cis-2aj**[12], **2ak**[13], **trans-2al**[14], **cis-2al**[14], **2am**[14], **2ao**[15], **4a**[16], **4d**[16], **4e**[17], **4f**[18], **4g**[19]. Compound **2o, 2ac, 2ae, 2af, 2ag, 2ah, 2ap, 2aq and 4c** are novel compounds.

2o and **2ad** were synthesized by following methods:

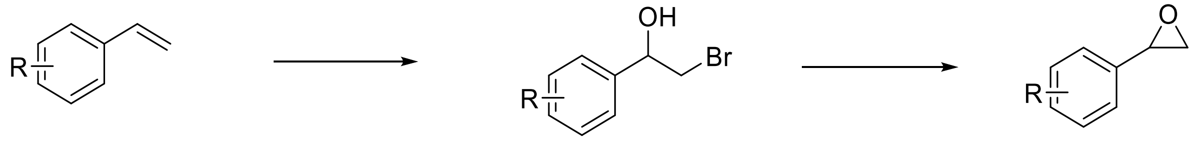

2o/2ad

An oven dried reaction flask, fitted with a magnetic stirrer, was charged with substituted styrene (1. 0 eq), NBS (1.1 eq) and NH_4OAc (10 %). The mixed solvent of acetone/water (4:1, 0.2 M) was added and the mixture was stirred at room temperature for 1 hours. The reaction was concentrated in *vacuo*, the residue was purified by column chromatography (EtOAc/Hex) to give corresponding 2-bromo-1-arylethan-1-ol which was dissolved in THF (0.1 M), then 2M NaOH (3.0 eq) was added and the mixture was stirred at room temperature for 1 hours and concentrated in *vacuo*. The residue was purified by column chromatography (EtOAc/Hex) to give corresponding styrene oxide.

3-(4-Cyclohexylphenyl)oxirane 2o

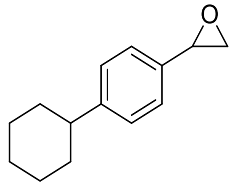

1-Cyclohexyl-4-vinylbenzene (930 mg, 5.0 mmol) was used employed. The crude mixture was purified by column chromatography (5.0% EtOAc/Hex) to yield the title compound 3-(4-cyclohexylphenyl)oxirane **2o** (504 mg, 4.24 mmol, 85%) as a colorless oil.

^{1}H NMR (400 MHz, $CDCl_3$) δ 7.18 (s, 4H), 3.81 (dd, *J* = 4.0, 2.6 Hz, 1H), 3.09 (dd, *J* = 5.5, 4.1 Hz, 1H), 2.78 (dd, *J* = 5.5, 2.6 Hz, 1H), 2.48 (ddd, *J* = 14.8, 8.3, 3.1 Hz, 1H), 1.83 (dd, *J* = 13.0, 6.9 Hz, 4H), 1.76 – 1.70 (m, 1H), 1.43 – 1.34 (m, 4H), 1.24 (dd, *J* = 9.9, 6.7 Hz, 1H). ^{13}C NMR (101 MHz, $CDCl_3$) δ 148.2, 134.9, 127.0, 125.5, 52.3, 51.1, 44.3, 34.4,

26.9, 26.1. m/z (ESI$^+$) HRMS: Calculated for $[C_{14}H_{18}NaO]^+$: 225.1250. Found $[M+Na]^+$:225.1248.

5-(Oxiran-2-yl)-2,3-dihydro-*1H*-inden-1-one 2ac

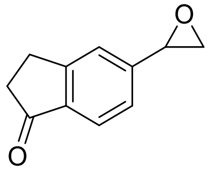

5-Vinyl-2,3-dihydro-1*H*-inden-1-one (790 mg, 5.0 mmol) was used employed. The crude mixture was purified by column chromatography (5% EtOAc/Hex) to yield the title compound 5-(oxiran-2-yl)-2,3-dihydro-*1H*-inden-1-one **2ac** (736 mg, 4.30 mmol, 86%) as a colorless oil.

^{1}H NMR (400 MHz, $CDCl_3$) δ 7.70 (d, J = 7.3 Hz, 1H), 7.49 – 7.32 (m, 2H), 4.10 – 3.95 (m, 1H), 3.32 – 3.12 (m, 3H), 2.82 (dd, J = 5.6, 2.6 Hz, 1H), 2.74 (t, J = 6.0 Hz, 2H). ^{13}C NMR (101 MHz, $CDCl_3$) δ 206.6, 153.2, 137.2, 135.6, 130.0, 128.0, 123.4, 50.5, 49.6, 36.1, 24.1. m/z (ESI$^+$) HRMS: Calculated for $[C_{11}H_{10}NaO_2]^+$:197.0573. Found $[M+Na]^+$:197.0583.

2af, **2ag**, **2ah** and **2ai** were synthesized by the Johnson−Corey−Chaykovsky reaction

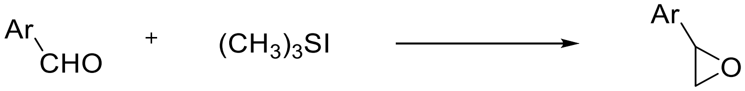

An oven dried reaction flask, fitted with a magnetic stirrer, was charged with sodium hydride (60% mineral oil dispersion, 1.5 eq) under an atmosphere of nitrogen, dry THF (1.0 M) and dry DMSO (1.0 M) were added and the reaction mixture was cooled in an ice bath. A solution of trimethylsulfonium iodide (1.2 eq) in DMSO (3.7 M) was added, and then corresponding aldehyde (1.0 eq) was added in one portion. The reaction mixture was stirred at 0°C for 30 min and additional 12 hours at room temperature. The reaction mixture was slowly quenched with cold water (1.5 mL/mmmol) and extracted with methylene chloride (3×1.5 mL/mmmol). The organic extracts were combined, dried over Na_2SO_4 and concentrated in *vacuo*. The residue was purified by column chromatography or directly used without further purification.

2-([1,1'-Binaphthalen]-2-yl)oxirane (racemic) 2ae

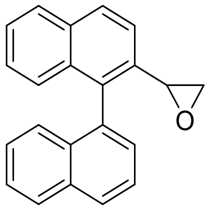

[1,1'-Binaphthalene]-2-carbaldehyde (racemic) (1.41 g, 5.0 mmol) was used employed. The crude mixture was purified by column chromatography (5% EtOAc/Hex) to yield the title compound 2-([1,1'-binaphthalen]-2-yl)oxirane **2ae** (636 mg, 43%) as a colorless oil. *Due to the existence of two stereocenters, **2ae** was obtained as a mixture of two diastereoisomers (1:0.7 ratio), which could not be separated by column chromatography.* The mixture of two diastereoisomers: ^{1}H NMR (400 MHz, CDCl3) δ 7.99 – 7.82 (m, 6.7H), 7.63 – 7.53 (m, 1.7H), 7.50 – 7.39 (m, 6.7H), 7.35 – 7.13 (m, 7H), 3.57 (t, *J* = 3.3 Hz, 0.7H), 3.46 (dd, *J* = 3.8, 2.8 Hz, 1H), 2.88 (dd, *J* = 5.4, 4.3 Hz, 1H), 2.76 (d, *J* = 2.6 Hz, 0.7 H), 2.75 (d, *J* = 2.6 Hz, 0.7H), 2.71 (d, *J* = 3.4 Hz, 1H). ^{13}C NMR (101 MHz, $CDCl_3$) δ 137.1, 136.9, 135.4, 135.4, 133.9, 133.7, 133.7, 133.7, 133.3, 133.2, 133.1, 133.0, 133.0, 132.6, 128.7, 128.6, 128.6, 128.4, 128.3, 128.3, 128.1, 128.0, 128.0, 126.6, 126.4, 126.4, 126.3, 126.3, 126.2, 126.1, 126.0, 126.0, 126.0, 125.6, 125.5, 121.1, 121.0, 51.0, 50.9, 50.8, 50.6. m/z (ESI^{+}) HRMS: Calculated for $[C_{22}H_{16}KO]^{+}$:335.0833. Found $[M+K]^{+}$:335.0831.

3-(Oxiran-2-yl)furan 2af

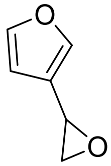

Furan-3-carbaldehyde (1.15 g, 12.0 mmol) was used employed. Title compound 3-(oxiran-2-yl)furan **2af** (1.21 g, 10.8 mmol, 90%) was obtained as a colorless oil without column chromatography purification. *(Note: this product is not stable on the column chromatography)*

^{1}H NMR (400 MHz, $CDCl_3$) δ 7.51 (s, 1H), 7.37 (d, *J* = 1.5 Hz, 1H), 6.27 (d, *J* = 1.5 Hz, 1H), 3.78 (dd, *J* = 3.7, 3.0 Hz, 1H), 3.08 (dd, *J* = 5.2, 3.7 Hz, 1H), 2.86 (dd, *J* = 5.2, 3, 0 Hz, 1H). ^{13}C NMR (101 MHz, $CDCl_3$) δ 143.5, 141.3, 122.9, 107.8, 49.3, 45.9. MS (EI): m/z (%): 110 (72) $[M]^{+}$, 81 (100), 53 (62).

2-(Thiophen-3-yl)oxirane 2ag

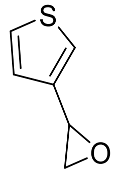

Thiophene-3-carbaldehyde (1.34 g, 12.0 mmol) was used employed. Title compound 2-(thiophen-3-yl)oxirane **2ag** (1.32 g, 10.5 mmol, 88%) was obtained as a colorless oil without column chromatography purification. *(Note: this product is not stable on the column chromatography)*

^{1}H NMR (400 MHz, $CDCl_3$) δ 7.31 – 7.28 (m, 2H), 6.97 – 6.96 (m, 1H), 4.00 – 3.85 (m, 1H), 3.12 (t, J = 4.7 Hz, 1H), 2.89 (dd, J = 5.3, 2.6 Hz, 1H). ^{13}C NMR (101 MHz, $CDCl_3$) δ 139.4, 126.4, 124.9, 122.6, 50.5, 49.3. MS (EI): m/z (%): 126 (28) $[M]^+$, 97 (100), 69 (9).

6- (Oxiran-2-yl)quinoline 2ah

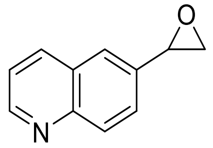

Quinoline-6-carbaldehyde (785 mg, 5.0 mmol) was used employed. The crude mixture was purified by column chromatography (10% EtOAc/Hex) to yield the title compound 2-([1,1'-binaphthalen]-2-yl)oxirane **2ah** (641 mg, 3.8 mmol, 75%) as a colorless oil.

^{1}H NMR (400 MHz, $CDCl_3$) δ 8.84 – 8.83 (m, 1H), 8.05 – 8.02 (m, 2H), 7.68 (s, 1H), 7.51–7.49 (m, 1H), 7.33 –7.30 (m, 1H), 4.01 – 3.92 (m, 1H), 3.16 (dd, J = 5.3, 4.1 Hz, 1H), 2.81 (dd, J = 5.4, 2.5 Hz, 1H). ^{13}C NMR (101 MHz, $CDCl_3$) δ 150.5, 148.1, 136.0, 135.8, 129.8, 128.0, 126.3, 124.7, 121.4, 52.1, 51.4. m/z (ESI^+) HRMS: Calculated for $[C_{11}H_{10}NO]^+$: 172.0757. Found $[M+H]^+$: 172.0750.

2ap and **2aq** were synthesized by the following method

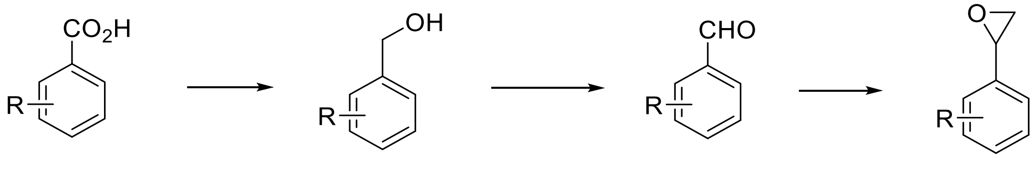

2ap/2aq

An oven dried reaction flask, fitted with a magnetic stirrer, was charged with aryl acid (Adapalene or Telmisartan) (1.0 eq) in THF (0.5 M), $LiAlH_4$ (1.5 eq) was added and the reaction mixture was heated at 60 °C for 12 hours. The suspension was cooled to r.t. and water (2.0 mL/mmmol) was added and the mixture was extracted with EtOAc (3×2.0 mL/mmmol), the organic extracts were combined, dried over Na_2SO_4 and concentrated in *vacuo*. The resulted alcohol product was directly dissolved in DCM (0.25 M) at the room temperature, PCC (2.0 eq) was added and the reaction mixture was allowed to stir at room temperature another 1 hour. The reaction was then concentrated *in vacuo,* the residue was purified by column chromatography to give corresponding aryl aldehyde which was further converted into final epoxides **2ap** and **2aq** using Johnson−Corey−Chaykovsky reaction produces that was described above for the synthesis of **2af**, **2ag**, **2ah** and **2ai**.

2-(6-(3-(Adamantan-1-yl)-4-methoxyphenyl)naphthalen-1-yl)oxirane 2ap

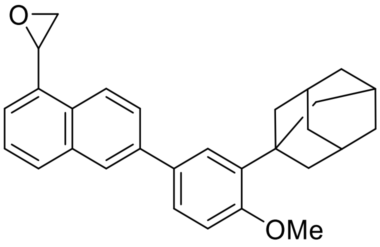

Adapalene (2.06 g, 5.0 mmol) (Cas number: 106685-40-9) was employed as the starting material. The crude mixture from Johnson−Corey−Chaykovsky reaction was purified by column chromatography (20% EtOAc/Hex) to yield the title compound 2-([1,1'-binaphthalen]-2-yl)oxirane **2ap** (1.29 g, 3.2 mmol, 63%) as a colorless oil.

^{1}H NMR (400 MHz, $CDCl_3$) δ 7.99 (s, 1H), 7.87 (s, 2H), 7.83 – 7.72 (m, 2H), 7.61 (s, 1H), 7.54 (d, *J* = 7.3 Hz, 1H), 7.35 (d, *J* = 7.3 Hz, 1H), 7.00 (d, *J* = 7.9 Hz, 1H), 4.05 (s, 1H), 3.91 (s, 3H), 3.24 (s, 1H), 2.94 (s, 1H), 2.21 (s, 6H), 2.12 (s, 3H), 1.82 (s, 6H). ^{13}C NMR (101 MHz, $CDCl_3$) δ 158.7, 139.3, 138.9, 134.7, 133.8, 133.0, 132.0, 128.6, 128.2, 126.3, 125.9, 125.7, 125.0, 124.9, 123.0, 112.1, 55.2, 52.8, 51.4, 40.7, 37.3, 37.2, 29.2. m/z (ESI$^+$) HRMS: Calculated for $[C_{29}H_{30}NaO_2]^+$: 433.2138. Found $[M+Na]^+$: 433.2145.

Telmisartan derivate 2aq

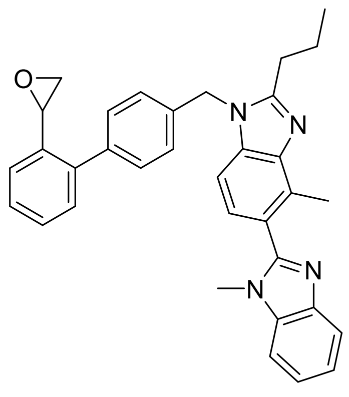

Telmisartan (2.57 g, 5.0 mmol) (Cas number: 144701-48-4) was employed as the starting material. The crude mixture from Johnson−Corey−Chaykovsky reaction was purified by column chromatography (50% EtOAc/Hex) to yield the **2aq** (1.87 g, 3.6 mmol, 73%) as a colorless oil.

^{1}H NMR (400 MHz, $CDCl_3$) δ 7.80 (dd, *J* = 7.0, 1.6 Hz, 1H), 7.41 (s, 2H), 7.34 – 7.27 (m, 5H), 7.25 – 7.19 (m, 4H), 7.11 – 7.09 (m, 2H), 5.39 (s, 2H), 3.73 (dd, *J* = 3.9, 2.7 Hz, 1H), 3.67 (s, 3H), 3.00 (dd, *J* = 5.6, 4.1 Hz, 1H), 2.93 – 2.85 (m, 2H), 2.77 (s, 3H), 2.73 (dd, *J* = 5.7, 2.6 Hz, 1H), 1.84 (dd, *J* = 15.4, 7.6 Hz, 2H), 1.02 (t, *J* = 7.4 Hz, 3H). ^{13}C NMR (101 MHz, $CDCl_3$) δ 156.3, 154.4, 143.0, 142.7, 140.7, 139.6, 136.5, 135.0, 134.9, 134.7, 129.8, 129.4, 129.3, 127.8, 127.6, 126.0, 123.9, 123.7, 123.7, 122.3, 122.1, 119.3, 109.4, 108.6, 51.3, 50.3, 46.7, 31.5, 29.6, 21.6, 16.8, 13.9. m/z (ESI$^+$) HRMS: Calculated for $[C_{34}H_{33}N_4O]^+$: 513.2649. Found $[M+H]^+$: 513.2651.

2-([1,1'-Biphenyl]-4-yl)oxetane 4c

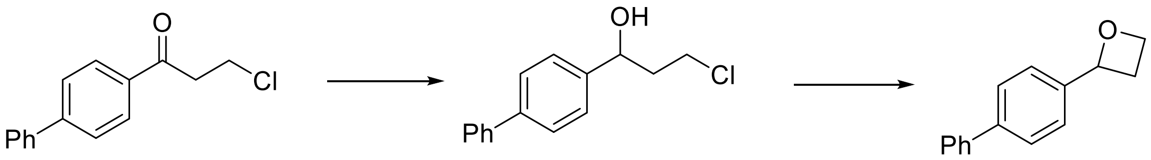

4c

An oven dried reaction flask, fitted with a magnetic stirrer, was charged with 1-([1,1'-biphenyl]-4-yl)-3-chloropropan-1-one (2.87 g, 12.0 mmol) in THF (20.0 mL) at room temperature, $NaBH_4$ (900 mg, 24.0 mmol) was added slowly and the reaction mixture was stirred at room temperature overnight. The reaction mixture was quenched with cold water (10 mL) and extracted with ethyl acetate (3×20 mL). The organic extracts were combined, dried over Na_2SO_4 and concentrated in *vacuo* to provide 1-([1,1'-biphenyl]-4-yl)-3-

chloropropan-1-ol (2.83 g, 11.5 mmol) which was directly dissolved in THF (15 mL), and KO^tBu (4.08 g, 36 mmol) was added and the reaction mixture was allowed to stir at room temperature for 6 hours. The reaction mixture was quenched with water (10 mL) and extracted with ethyl acetate (3×20 mL). The organic extracts were combined, dried over Na_2SO_4 and concentrated in *vacuo*. The residue was purified by column chromatography (petroleum ether/EtOAc/Et_3N 50:1:1) to give 2-([1,1'-biphenyl]-4-yl)oxetane **4c** (792 mg, 3.8 mmol, 32%) as a colorless oil.

^{1}H NMR (400 MHz, $CDCl_3$) δ 7.65 – 7.61 (m, 4H), 7.54 – 7.52 (m, 2H), 7.47 – 7.44 (m, 2H), 7.38 – 7.34 (m, 1H), 5.87 (dd, *J* = 7.5 Hz, 1H), 4.87 (ddd, *J* = 9.2, 8.0, 6.0 Hz, 1H), 4.70 (ddd, *J* = 9.2, 8.0, 6.0 Hz, 1H), 3.14 – 2.98 (m, 1H), 2.80 – 2.65 (m, 1H). ^{13}C NMR (101 MHz, $CDCl_3$) δ 142.7, 141.0, 140.9, 128.9, 127.4, 127.4, 127.2, 125.8, 82.8, 68.4, 30.8. m/z (ESI^+) HRMS: Calculated for $[C_{15}H_{14}ONa]^+$: 233.0937. Found $[M+Na]^+$: 233.0934.

3. Reaction Optimization

Supplementary Table 1. Optimization using RuBnN as catalyst

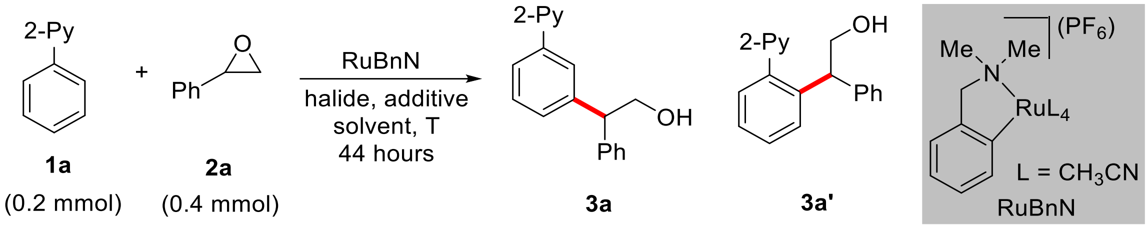

Ru loading	halide	additive	solvent	T	Yield of **3a/3a'**
5%	NaI 30%	C_6H_5COOH (30%)	dioxane (0.4M)	80 °C	27%/trace
5%	NaI 30%	None	dioxane (0.4M)	80 °C	0%/0%
5%	none	C_6H_5COOH (30%)	dioxane (0.4M)	80 °C	0%/0%
5%	NaI 30%	C_6H_5COOH (30%)	MeOH (0.4M)	80 °C	19%/25%
5%	NaI 30%	C_6H_5COOH (30%)	NMP (0.4M)	80 °C	trace/trace
5%	NaI 30%	C_6H_5COOH (30%)	DMF (0.4M)	80 °C	0%/0%
5%	NaI 30%	C_6H_5COOH (30%)	CH_3CN (0.4M)	80 °C	0%/0%
5%	NaI 30%	C_6H_5COOH (30%)	toluene (0.4M)	80 °C	15%/trace
5%	NaI 30%	C_6H_5COOH (30%)	DCE (0.4M)	80 °C	trace/trace
5%	NaI 30%	C_6H_5COOH (30%)	2-MeTHF (0.4M)	80 °C	24%/trace
5%	NaI 30%	C_6H_5COOH (30%)	DMSO (0.4M)	80 °C	0%/0%
5%	NaI 30%	C_6H_5COOH (30%)	H_2O (0.4M)	80 °C	22%/0%
5%	NaI 30%	C_6H_5COOH (30%)	acetone (0.4M)	80 °C	22%/0%
5%	NaI 30%	C_6H_5COOH (100%)	dioxane (0.4M)	80 °C	11%/trace
5%	NaI 30%	C_6H_5COOH (30%)	dioxane (0.4M)	100 °C	14%/trace
5%	NaI 30%	C_6H_5COOH (30%)	dioxane (0.4M)	120 °C	6%/trace
5%	NaI 30%	C_6H_5COOH (30%)	dioxane (0.4M)	60 °C	24%/trace
2.5%	NaI 30%	C_6H_5COOH (30%)	dioxane (0.4M)	80 °C	14%/trace
10%	NaI 30%	C_6H_5COOH (30%)	dioxane (0.4M)	80 °C	19%/trace
5%	NaI 30%	C_6H_5COOH (30%)	dioxane (0.2M)	80 °C	20%/trace
5%	NaI 30%	C_6H_5COOH (30%)	dioxane (0.8M)	80 °C	24%/trace
5%	NaI 30%	MesCOOH (30%)	dioxane (0.4M)	80 °C	9%/trace
5%	NaI 30%	4-$NMe_2C_6H_4COOH$ (30%)	dioxane (0.4M)	80 °C	22%/trace

5%	NaI 30%	4-$NO_2C_6H_4COOH$ (30%)	dioxane (0.4M)	80 ºC	mess
5%	NaI 30%	Ad-COOH (30%)	dioxane (0.4M)	80 ºC	21%/trace
5%	NaI 30%	HOAc (30%)	dioxane (0.4M)	80 ºC	20%/trace

Supplementary Table 2. Screening of different Ru-catalyst

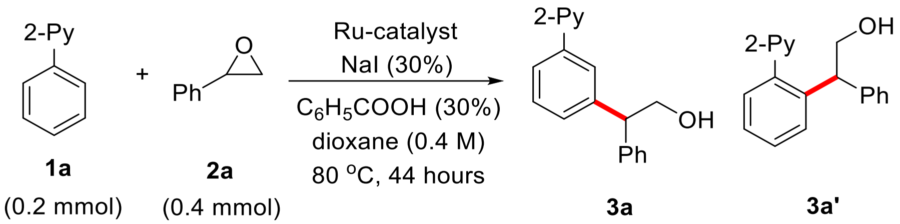

Ru-catalyst	Yield of **3a**/**3a'**
$Ru(PPh_3)Cl_2$ (5%)	15%/5%
[Ru(*p*-cymene)Cl_2]$_2$ (5%)	0%/0%
Ru(*p*-cymene)$(OPiv)_2$ (5%)	0%/0%
Ru(*p*-cymene)$(OAc)_2$ (5%)	0%/0%
Ru(benzene)$(OPiv)_2$ (5%)	8%/trace
Ru(C_6Me_6)$(OPiv)_2$ (5%)	0%/0%

Supplementary Table 3. Optimization using $Ru(PPh_3)Cl_2$ as catalyst

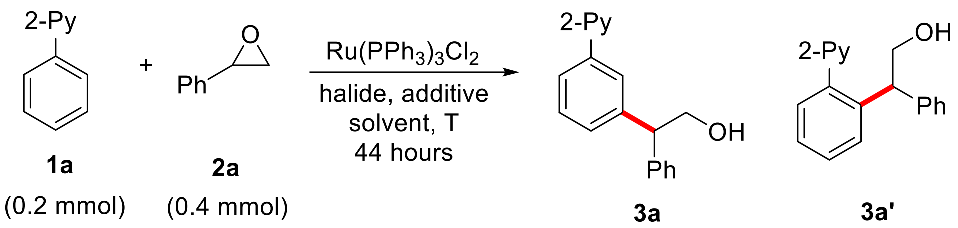

Ru loading	halide	additive	solvent	T	Yield of **3a/3a'**
5%	NaI 30%	C_6H_5COOH (30%)	dioxane (0.4M)	80 °C	15%/5%
5%	NaI 30%	C_6H_5COOH (30%)	MeOH (0.4M)	80 °C	15%/14%
5%	NaI 30%	C_6H_5COOH (30%)	toluene (0.4M)	80 °C	26%/12%
5%	NaI 30%	C_6H_5COOH (30%)	CH_3CN (0.4M)	80 °C	0%/0%
5%	NaI 30%	TsOH.H_2O	dioxane (0.4M)	80 °C	0%/0%
5%	NaI 30%	N-Boc-Proline (30%)	dioxane (0.4M)	80 °C	41%/0%
5%	NaI 30%	PivOH (30%)	dioxane (0.4M)	80 °C	37%/0%
5%	NaI 30%	C_4H_9COOH (30%)	dioxane (0.4M)	80 °C	15%/0%
5%	NaI 30%	1-Me-C_{hex}-COOH (30%)	dioxane (0.4M)	80 °C	48%/0%
5%	NaI 30%	2-ethylbutanoic acid (30%)	dioxane (0.4M)	80 °C	50%/0%
5%	NaI 30%	2-ethylbutanoic acid (30%)	toluene (0.4M)	80 °C	46%/0%
5%	NaI 30%	2-ethylbutanoic acid (30%)	ethyl acetate (0.4M)	80 °C	46%/0%
5%	NaI 30%	2-ethylbutanoic acid (30%)	dioxane (1.3 M)	80 °C	62%/0%
5%	NaI 30%	2-ethylbutanoic acid (30%)	dioxane (0.8 M)	80 °C	46%/0%
5%	NaI 100%	2-ethylbutanoic acid (30%)	dioxane (1.3 M)	80 °C	68%/0%
5%	NaI 100%	2-ethylbutanoic acid (30%)	dioxane (1.3 M)	70 °C	75%/0%
5%	NaI 100%	2-ethylbutanoic acid (30%)	dioxane (1.3 M)	90 °C	64%/0%
5%	*n*BuNI 100%	2-ethylbutanoic acid (30%)	dioxane (1.3 M)	70 °C	73%/0%
5%	NaBr 100%	2-ethylbutanoic acid (30%)	dioxane (1.3 M)	70 °C	46%/0%
5%	NaBr 100%	2-ethylbutanoic acid (30%)	dioxane (1.3 M)	70 °C	19%/0%
5%	None	2-ethylbutanoic acid (30%)	dioxane (1.3 M)	70 °C	15%/0%
5%	NaI 100%	None	dioxane (1.3 M)	70 °C	19%/0%

Supplementary Table 4. Screening of different additional bases

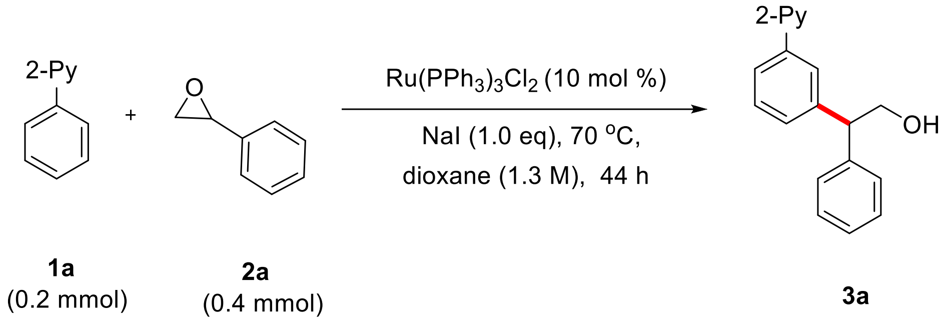

Acid	Base	Yield of 3a
2-ethylbutanoic acid (60 mol %)	K_2CO_3 (15%)	72%
2-ethylbutanoic acid (60 mol %)	Na_2CO_3 (15%)	55%
2-ethylbutanoic acid (60 mol %)	Cs_2CO_3 (15%)	75%
2-ethylbutanoic acid (60 mol %)	Li_2CO_3 (15%)	71%
2-ethylbutanoic acid (60 mol %)	$MgCO_3$ (15%)	72%
2-ethylbutanoic acid (30 mol %)	NaO_2CPh (30%)	69%
2-ethylbutanoic acid (30 mol %)	NaOAc (30%)	68%
2-ethylbutanoic acid (30 mol %)	KOAc (30%)	76%
2-ethylbutanoic acid (30 mol %)	CsOAc (30%)	71%
2-ethylbutanoic acid (30 mol %)	$Mg(OAc)_2$ (30%)	72%

4. General Procedure

General Procedure A: Ru-catalyzed *meta*-C−H Alkylation with Epoxides

In a glove box, an oven-dried crimp-cap microwave vial equipped with a magnetic stirring bar was charged with $Ru(PPh_3)_3Cl_2$ (5.0 mol %), NaI powder (1.0 equiv) and 2-ethylbutyric acid (30 mol %), then substrates **1** (0.20 mmol), epoxide **2** (2.0 equiv) and dioxane (1.3 M) were added. The vial was then capped and taken out of glovebox, stirred at 70°C for 44 h. The reaction was then allowed to cool to room temperature and concentrated *in vacuo*. The residue was purified by column chromatography under the conditions noted to yield the desired product.

Note: Using finely ground NaI powder in the reaction will provide a better result than normal NaI granules. The reaction can also be carried out by using a schlenk line technique with N_2 atmosphere, however, inferior reproducibility was obtained presumably because of the moisture-sensitive properties of NaI powder.

General Procedure B: Ru-catalyzed *meta*-C−H Alkylation with Epoxides

In a glove box, an oven-dried crimp-cap microwave vial equipped with a magnetic stirring bar was charged with $Ru(PPh_3)_3Cl_2$ (10 mol %), NaI powder (1.0 equiv) and 2-ethylbutyric acid (30 mol %), then substrates **1** (0.20 mmol), epoxide **2** (4.0 equiv) and dioxane (1.3 M) were added. The vial was then capped and taken out of glovebox, stirred at 70°C for 44 h. The reaction was then allowed to cool to room temperature and concentrated *in vacuo*. The residue was purified by column chromatography under the conditions noted to yield the desired product.

General Procedure C: Ru-catalyzed *meta*-C−H Alkylation with Oxetanes

In a glove box, an oven-dried crimp-cap microwave vial equipped with a magnetic stirring bar was charged with $Ru(PPh_3)_3Cl_2$ (10 mol %), NaI powder (2.0 equiv) and 2-ethylbutyric acid (30 mol %), then substrates **1** (0.20 mmol), oxetane **4** (4.0 equiv) and dioxane (1.3 M) were added. The vial was then capped and taken out of glovebox, stirred at 70°C for 44 h. The reaction was then allowed to cool to room temperature and concentrated *in vacuo*. The residue was purified by column chromatography under the conditions noted to yield the desired product.

5. Products Characterization Data

2-Phenyl-2-(3-(pyridin-2-yl)phenyl)ethan-1-ol 3a

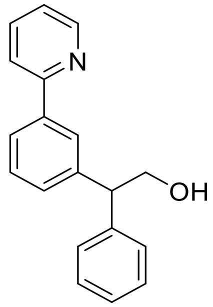

Obtained by using **General procedure A**: 2-Phenylpyridine **1a** (32.0 uL, 0.20 mmol) and 2-phenyloxirane **2a** (46.0 uL, 0.40 mmol) were employed. The crude mixture was purified by column chromatography (40% EtOAc/Hex) to yield the title compound **3a** (41.2 mg, 75%) as a colorless oil; ^{1}H NMR (400 MHz, $CDCl_3$) δ 8.73 – 8.65 (m, 1H), 8.00 (d, *J* = 2.0 Hz, 1H), 7.89 – 7.83 (m, 1H), 7.83 – 7.70 (m, 2H), 7.50 – 7.44 (m, 1H), 7.39 – 7.32 (m, 5H), 7.31 – 7.24 (m, 2H), 4.36 (app. t, *J* = 7.1 Hz, 1H), 4.33 – 4.21 (m, 2H), 2.62 (s, br, 1H). ^{13}C NMR (101 MHz, $CDCl_3$) δ 157.7, 156.7, 148.6, 140.5, 137.5, 130.6, 129.2, 128.7, 128.5, 128.4, 127.0, 126.6, 121.5, 121.0, 117.4, 65.7, 48.1. m/z (ESI^{+}) HRMS: Calculated for $[C_{19}H_{18}NO]^{+}$: 276.1383. Found $[M+H]^{+}$: 276.1378.

2-phenyl-2-(2-(pyridin-2-yl)phenyl)ethan-1-ol 3a’

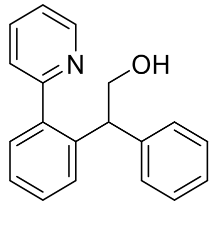

3a’ was the regioisomer of **3a** that obtained during reaction optimization under a few conditions, and it was isolated with a small amount of impurity. ^{1}H NMR (400 MHz, $CDCl_3$) δ 8.67 (d, *J* = 4.4 Hz, 1H), 7.87 (m, 1H), 7.59 (d, *J* = 7.8 Hz, 1H), 7.39 – 7.33 (m, 4H), 7.29 (m, 2H), 7.22 (d, *J* = 7.6 Hz, 1H), 7.15 (m, 1H), 7.03 (m, 2H), 6.33 (s, br, 1H), 4.67 (dd, *J* = 11.0, 4.9 Hz, 1H), 4.43 (dd, *J* = 9.6, 4.9 Hz, 1H), 4.24 (app. t, *J* = 10.3, Hz, 1H). ^{13}C NMR (101 MHz, $CDCl_3$) δ 159.4, 147.9, 142.8, 142.1, 140.1, 137.8, 130.0, 129.4, 129.2,

129.1, 128.5, 128.0, 126.4, 124.9, 122.3, 66.9, 46.7. m/z (ESI^+) HRMS: Calculated for $[C_{19}H_{18}NO]^+$: 276.1383. Found $[M+H]^+$: 276.1386.

*The identification of **3a'** was based on following: 1, the obtained ^1H NMR signal of aliphatic protons indicated that it was not the product resulting from styrene oxide ring opening from the less hindered C-O bond; 2, the unusual chemical shift of aliphatic hydroxy group (6.33 (s, br, 1H)) indicated a potential hydrogen bonding was formed in the structure (with the N atom in the pyridine ring); 3, ^1H NMR signal of the C-H ortho to the pyridine N at 8.67 (d, J = 4.4 Hz, 1H) suggests that no N-alkylation has taken place; 4, the overall ^1H NMR signal of aromatic region is similar to that of 2-(2-(1-phenylethyl)phenyl)pyridine reported in the literature[20].*

2-(2-Hydroxy-1-phenylethyl)-4-(pyridin-2-yl)phenol 3b

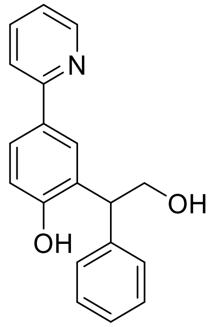

Obtained by using **General procedure A**: 4-(pyridin-2-yl)phenol **1b** (34.2 mg, 0.20 mmol) and 2-phenyloxirane **2a** (46.0 uL, 0.40 mmol) were employed. The crude mixture was purified by column chromatography (50% EtOAc/Hex) to yield the title compound **3b** (46.4 mg, 80%) as a colorless oil; ^1H NMR (400 MHz, $CDCl_3$) δ 8.45 (d, J = 4.9 Hz, 1H), 7.69 – 7.61 (m, 1H), 7.55 (d, J = 2.2 Hz, 1H), 7.51 – 7.43 (m, 2H), 7.23 – 7.06 (m, 7H), 6.75 (d, J = 8.3 Hz, 1H), 4.45 (app. t, J = 7.1 Hz, 1H), 4.15 – 4.01 (m, 2H). ^{13}C NMR (101 MHz, $CDCl_3$) δ 157.6, 156.7, 148.6, 140.4, 137.5, 130.7, 129.2, 128.8, 128.6, 128.4, 127.1, 126.7, 121.5, 120.9, 117.5, 65.8, 48.3. m/z (ESI^+) HRMS: Calculated for $[C_{19}H_{18}NO_2]^+$: 292.1332. Found $[M+H]^+$: 292.1333.

2-(2-Methoxy-5-(pyridin-2-yl)phenyl)-2-phenylethan-1-ol 3c

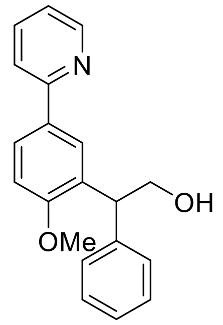

Obtained by using **General procedure A**: 2-(4-methoxyphenyl)pyridine **1c** (37.0 mg, 0.20 mmol) and 2-phenyloxirane **2a** (46.0 uL, 0.40 mmol) were employed. The crude mixture was purified by column chromatography (50% EtOAc/Hex) to yield the title compound **3c** (50.4 mg, 83%) as a colorless oil; ^{1}H NMR (400 MHz, $CDCl_3$) δ 8.58 (d, *J* = 4.8 Hz, 1H), 7.89 (d, *J* = 2.3 Hz, 1H), 7.80 (dd, *J* = 8.5, 2.3 Hz, 1H), 7.70 – 7.64 (m, 1H), 7.59 (d, *J* = 7.9 Hz, 1H), 7.32 – 7.23 (m, 4H), 7.20 – 7.12 (m, 2H), 6.92 (d, *J* = 8.6 Hz, 1H), 4.68 (app. t, *J* = 7.2 Hz, 1H), 4.24 (dd, *J* = 11.1, 7.6 Hz, 1H), 4.15 (dd, *J* = 11.1, 7.1 Hz, 1H), 3.78 (s, 3H). 2.61 (s, br, 1H). ^{13}C NMR (101 MHz, $CDCl_3$) δ 158.5, 157.1, 149.2, 141.4, 136.8, 131.5, 130.2, 128.5, 128.4, 126.8, 126.5, 126.4, 121.4, 120.1, 111.0, 65.2, 55.6, 46.6. m/z (ESI$^+$) HRMS: Calculated for $[C_{20}H_{20}NO_2]^+$: 306.1489. Found $[M+H]^+$: 306.1485.

2-(2-(Methoxymethyl)-5-(pyridin-2-yl)phenyl)-2-phenylethan-1-ol 3d

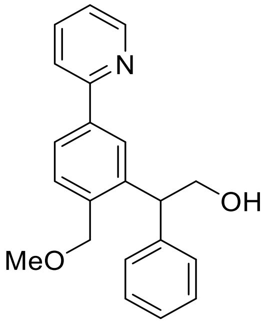

Obtained by using **General procedure A**: 2-(4-(methoxymethyl)phenyl)pyridine **1d** (39.8 mg, 0.20 mmol) and 2-phenyloxirane **2a** (46.0 uL, 0.40 mmol) were employed. The crude mixture was purified by column chromatography (40% EtOAc/Hex) to yield the title compound **3d** (62.0 mg, 97%) as a colorless oil; ^{1}H NMR (400 MHz, $CDCl_3$) δ 8.65 – 8.57 (m, 1H), 7.99 (d, *J* = 1.7 Hz, 1H), 7.79 (dd, *J* = 7.9, 1.8 Hz, 1H), 7.74 – 7.68 (m, 1H), 7.65 (d, *J* = 7.9 Hz, 1H), 7.44 (d, *J* = 7.9 Hz, 1H), 7.29 – 7.22 (m, 4H), 7.21 – 7.16 (m, 2H),

4.62 – 4.52 (m, 2H), 4.34 (d, *J* = 12.0 Hz, 1H), 4.27 – 4.17(m, 2H), 3.34 (s, 3H), 3.16 (s, br, 1H). ^{13}C NMR (101 MHz, $CDCl_3$) δ 157.3, 149.6, 141.4, 140.8, 139.1, 137.6, 136.9, 130.0, 128.6, 128.5, 126.7, 126.2, 125.2, 122.2, 120.9, 72.6, 66.1, 58.2, 48.6. m/z (ESI^+) HRMS: Calculated for $[C_{21}H_{22}NO_2]^+$: 320.1645. Found $[M+H]^+$: 320.1654.

2-(2-Fluoro-5-(pyridin-2-yl)phenyl)-2-phenylethan-1-ol 3e

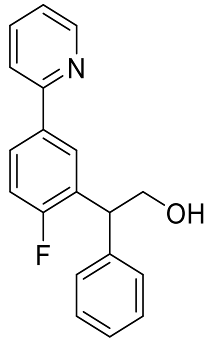

Obtained by using **General procedure A**: 2-(4-fluorophenyl)pyridine **1e** (34.6 mg, 0.20 mmol) and 2-phenyloxirane **2a** (46.0 uL, 0.40 mmol) were employed. The crude mixture was purified by column chromatography (40% EtOAc/Hex) to yield the title compound **3e** (55.3 mg, 94%) as a colorless oil; ^{1}H NMR (400 MHz, $CDCl_3$) δ 8.64 – 8.60 (m, 1H), 8.00 (dd, *J* = 7.1, 2.3 Hz, 1H), 7.80 – 7.70 (m, 2H), 7.64 (dt, *J* = 7.9, 1.2 Hz, 1H), 7.33 – 7.29 (m, 4H), 7.24 – 7.19 (m, 2H), 7.11 (dd, *J* = 9.8, 8.5 Hz, 1H), 4.58 (app. t, *J* = 7.3 Hz, 1H), 4.29 (dd, *J* = 11.2, 7.7 Hz, 1H), 4.21 (dd, *J* = 11.2, 6.9 Hz, 1H), 2.89 (s, br, 1H). ^{13}C NMR (101 MHz, $CDCl_3$) δ 161.9 (d, *J* = 249.0 Hz), 156.5, 149.4, 140.4, 137.0, 135.3 (d, *J* = 3.4 Hz), 129.1 (d, *J* = 15.1 Hz), 128.6, 128.3, 127.8 (d, *J* = 4.9 Hz), 126.9 (d, *J* = 8.8 Hz), 126.8, 122.1, 120.6, 116.0 (d, *J* = 23.3 Hz), 64.8, 46.8 (d, *J* = 1.6 Hz). ^{19}F NMR (376 MHz, $CDCl_3$) δ -116.4 (ddd, *J* = 10.3, 5.6, 5.6 Hz). m/z (ESI^+) HRMS: Calculated for $[C_{19}H_{17}FNO]^+$: 294.1289. Found $[M+H]^+$: 294.1288.

2-(2-Chloro-5-(pyridin-2-yl)phenyl)-2-phenylethan-1-ol 3f

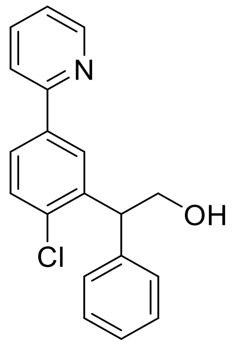

Obtained by using **General procedure A**: 2-(4-chlorophenyl)pyridine **1f** (37.8 mg, 0.20 mmol) and 2-phenyloxirane **2a** (46.0 uL, 0.40 mmol). The crude mixture was purified by column chromatography (40% EtOAc/Hex) to yield the title compound **3f** (53.9 mg, 82%) as a colorless oil; ^{1}H NMR (400 MHz, $CDCl_3$) δ 8.56 (dd, *J* = 4.7, 2.3 Hz, 1H), 7.99 (d, *J* = 2.2 Hz, 1H), 7.73 – 7.65 (m, 2H), 7.60 (d, *J* = 7.9 Hz, 1H), 7.39 (dd, *J* = 8.3, 1.5 Hz, 1H), 7.28 – 7.12 (m, 6H), 4.69 (app. t, *J* = 7.1 Hz, 1H), 4.22 (dd, *J* = 11.5, 7.6 Hz, 1H), 4.12 (dd, *J* = 11.5, 6.7 Hz, 1H), 2.95 (s, br, 1H).^{13}C NMR (101 MHz, $CDCl_3$) δ 156.3, 149.5, 140.1, 139.3, 137.8, 137.0, 136.0, 130.3, 128.6, 128.6, 127.2, 126.8, 126.3, 122.4, 120.7, 65.1, 49.9. m/z (ESI$^+$) HRMS: Calculated for $[C_{19}H_{17}ClNO]^+$: 310.0993. Found $[M+H]^+$: 310.0992.

2-Phenyl-2-(5-(pyridin-2-yl)-2-(trifluoromethyl)phenyl)ethan-1-ol 3g

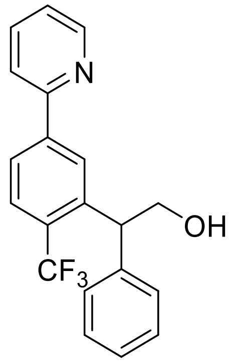

Obtained by using **General procedure A**: 2-(4-(trifluoromethyl)phenyl)pyridine **1g** (44.6 mg, 0.20 mmol) and 2-phenyloxirane **2a** (46.0 uL, 0.40 mmol) were employed. The crude mixture was purified by column chromatography (50% EtOAc/Hex) to yield the title compound **3g** (58.9 mg, 86%) as a colorless oil; ^{1}H NMR (400 MHz, $CDCl_3$) δ 8.65 (d, *J* = 5.9 Hz, 1H), 8.11 (s, 1H), 7.87 (d, *J* = 8.3 Hz, 1H), 7.82 – 7.69 (m, 2H), 7.66 (d, *J* = 7.9

Hz, 1H), 7.38 – 7.25 (m, 5H), 7.24 – 7.17 (m, 1H), 4.73 (app. t, *J* = 7.0 Hz, 1H), 4.23 (app. d, *J* = 7.0 Hz, 2H), 2.71 (d, br, *J* = 1.9 Hz, 1H). ^{13}C NMR (101 MHz, $CDCl_3$) δ 155.8, 149.7, 142.2, 140.6, 137.1, 129.3 (q, *J* = 30.0 Hz), 128.6, 128.5 (q, *J* = 6.7 Hz) 128.3, 128.2, 126.8 (q, *J* = 6.0 Hz), 126.7, 125.8 (q, *J* = 273.0 Hz), 125.0, 123.0, 121.1, 65.7, 48.5. ^{19}F NMR (376 MHz, $CDCl_3$) δ -58.0 (s). m/z (ESI$^+$) HRMS: Calculated for $[C_{20}H_{17}F_3NO]^+$: 344.1257. Found $[M+H]^+$: 344.1249.

Methyl 3-(2-(2-hydroxy-1-phenylethyl)-4-(pyridin-2-yl)phenyl)propanoate 3h

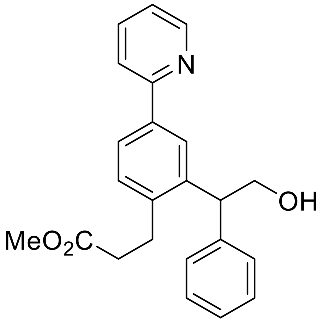

Obtained by using **General procedure A**: methyl 3-(4-(pyridin-2-yl)phenyl)propanoate **1h** (48.2 mg, 0.20 mmol) and 2-phenyloxirane **2a** (48.0 mg, 0.40 mmol) were employed. The crude mixture was purified by column chromatography (40% EtOAc/Hex) to yield the title compound **3h** (48.0 mg, 67%) as a colorless oil; ^{1}H NMR (400 MHz, $CDCl_3$) δ 8.59 (d, *J* = 4.7 Hz, 1H), 8.02 (d, *J* = 1.6 Hz, 1H), 7.76 – 7.70 (m, 2H), 7.67 (d, *J* = 7.9 Hz, 1H), 7.27 – 7.23 (m, 5H), 7.22 – 7.17 (m, 2H), 4.51 (app. t, *J* = 7.2 Hz, 1H), 4.27 (dd, *J* = 11.2, 8.0 Hz, 1H), 4.16 (dd, *J* = 11.2, 6.6 Hz, 1H), 3.64 (s, 3H), 3.15 (s, br, 1H), 3.06 – 2.96 (m, 1H), 2.96 – 2.86 (m, 1H), 2.54 – 2.46 (m, 1H), 2.38 – 2.30 (m, 1H). ^{13}C NMR (101 MHz, $CDCl_3$) δ 173.3, 157.4, 149.6, 141.4, 140.7, 139.6, 137.7, 136.9, 130.2, 128.7, 128.6, 126.8, 125.9, 125.5, 122.1, 120.7, 66.2, 51.7, 49.3, 35.0, 27.6. m/z (ESI$^+$) HRMS: Calculated for $[C_{23}H_{23}NNaO_3]^+$: 384.1570. Found $[M+Na]^+$: 384.1584.

2-Phenyl-2-(2-(phenylethynyl)-5-(pyridin-2-yl)phenyl)ethan-1-ol 3i

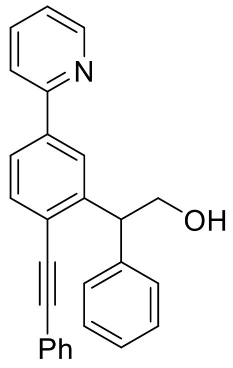

Obtained by using **General procedure A**: 2-(4-(phenylethynyl)phenyl)pyridine **1i** (51.0 mg, 0.20 mmol) and 2-phenyloxirane **2a** (46.0 uL, 0.40 mmol) were employed. The crude mixture was purified by column chromatography (50% EtOAc/Hex) to yield the title compound **3i** (66.1 mg, 88%) as a colorless oil; ^{1}H NMR (400 MHz, $CDCl_3$) δ 8.66 – 8.63 (m, 1H), 8.07 (d, J = 1.8 Hz, 1H), 7.80 (dd, J = 8.1, 1.8 Hz, 1H), 7.77 – 7.66 (m, 2H), 7.63 (d, J = 8.1 Hz, 1H), 7.55 – 7.49 (m, 2H), 7.40 – 7.34 (m, 5H), 7.33 – 7.27 (m, 2H), 7.23 – 7.17 (m, 2H), 4.95 (app, t, J = 7.2 Hz, 1H), 4.37 (dd, J = 11.3, 7.6 Hz, 1H), 4.26 (dd, J = 11.3, 6.9 Hz, 1H), 2.86 (s, br, 1H).^{13}C NMR (101 MHz, $CDCl_3$) δ 156.6, 149.5, 143.6, 141.0, 139.0, 136.9, 133.2, 131.5, 128.6, 128.5, 128.4, 128.4, 126.6, 125.5, 125.0, 124.6, 123.2, 122.4, 120.9, 95.2, 88.0, 65.4, 51.3. m/z (ESI$^+$) HRMS: Calculated for $[C_{27}H_{22}ON]^+$: 376.1696. Found $[M+H]^+$: 376.1700.

2-(2-Fluoro-3-(pyridin-2-yl)phenyl)-2-phenylethan-1-ol 3j

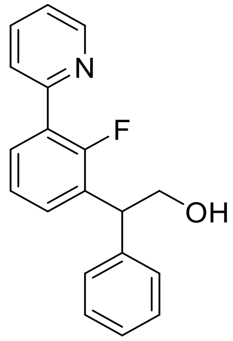

Obtained by using **General procedure A**: 2-(2-fluorophenyl)pyridine **1j** (34.6 mg, 0.20 mmol) and 2-phenyloxirane **2a** (46.0 uL, 0.40 mmol) were employed. The crude mixture was purified by column chromatography (40% EtOAc/Hex) to yield the title compound **3j** (51.4 mg, 88%) as a colorless oil; ^{1}H NMR (400 MHz, $CDCl_3$) δ 8.66 (d, J = 4.8 Hz, 1H),

7.78 – 7.72 (m, 1H), 7.70 – 7.65 (m, 2H), 7.36 – 7.25 (m, 5H), 7.23 – 7.17 (m, 3H), 4.58 (app. t, J = 7.2 Hz, 1H), 4.17 (app. d, J = 7.2 Hz, 2H), 1.97 (s, br, 1H). ^{13}C NMR (101 MHz, $CDCl_3$) δ 158.5 (d, J = 249.9 Hz), 153.5, 149.6, 140.3, 136.4, 129.6 (d, J = 3.3 Hz), 129.4 (d, J = 4.5 Hz), 129.3 (d, J = 15.8 Hz), 128.7, 128.3, 127.8 (d, J = 13.1 Hz), 126.9, 124.6 (d, J = 8.2 Hz), 124.3 (d, J = 4.3 Hz), 122.4, 65.1, 46.5 (d, J = 3.0 Hz). ^{19}F NMR (376 MHz, $CDCl_3$) δ -122.1 (t, J = 6.8 Hz). m/z (ESI^+) HRMS: Calculated for $[C_{19}H_{17}FNO]^+$: 294.1289. Found $[M+H]^+$: 294.1285.

Given the coupling on the ^{19}F NMR, an apparent triplet with less than 7 Hz coupling, indicating two $^4J_{HF}$ couplings, which is consistent with alkylation ortho with respect to fluorine.

2-(3-Fluoro-5-(pyridin-2-yl)phenyl)-2-phenylethan-1-ol 3k

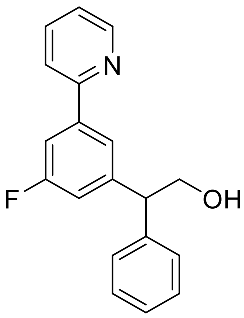

Obtained by using **General procedure A**: 2-(3-fluorophenyl)pyridine **1k** (34.6 mg, 0.20 mmol) and 2-phenyloxirane **2a** (46.0 uL, 0.40 mmol) were employed. The crude mixture was purified by column chromatography (50% EtOAc/Hex) to yield the title compound **3k** (13.7 mg, 23%) as a yellow oil; ^{1}H NMR (400 MHz, $CDCl_3$) δ 8.68 (d, J = 4.8 Hz, 1H), 7.81 – 7.74 (m, 2H), 7.69 (d, J = 8.0 Hz, 1H), 7.59 – 7.54 (m, 1H), 7.39 – 7.19 (m, 6H), 7.04 (d, J = 9.5 Hz, 1H), 4.32 (t, J = 7.1 Hz, 1H), 4.28 – 4.18 (m, 2H), 2.06 (br s, 1H). ^{13}C NMR (101 MHz, CDCl3) δ 163.6 (d, J = 245.7 Hz), 156.2 (d, J = 2.7 Hz), 149.8, 144.7 (d, J = 7.2 Hz), 141.9 (d, J = 8.0 Hz), 140.9, 137.1, 129.0, 128.5, 127.2, 122.9, 122.7 (d, J = 2.5 Hz), 120.9, 115.9 (d, J = 21.9 Hz), 112.5 (d, J = 22.9 Hz), 66.0, 53.7 (d, J = 1.5 Hz). ^{19}F NMR (376 MHz, $CDCl_3$) δ -112.6 (dd, J = 9.6, 9.6 Hz). m/z (ESI^+) HRMS: Calculated for $[C_{19}H_{17}FNO]^+$: 294.1289. Found $[M+H]^+$: 294.1282.

2-(2,5-Difluoro-3-(pyridin-2-yl)phenyl)-2-phenylethan-1-ol 3l

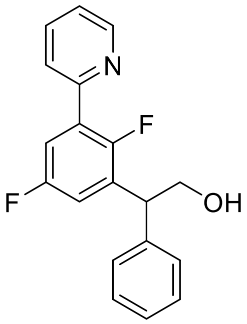

Obtained by using **General procedure A**: 2-(2,3-difluorophenyl)pyridine **1l** (38.2 mg, 0.20 mmol) and 2-phenyloxirane **2a** (46.0 uL, 0.40 mmol) were employed. The crude mixture was purified by column chromatography (40% EtOAc/Hex) to yield the title compound **3l** (16.6 mg, 27%) as a colorless oil; ^{1}H NMR (400 MHz, $CDCl_3$) δ 8.62 (dt, J = 4.8, 1.4 Hz, 1H), 7.67 (dt, J = 5.1, 1.5 Hz, 2H), 7.50 (ddd, J = 9.0, 5.8, 3.3 Hz, 1H), 7.31 – 7.16 (m, 7H), 7.01 (ddd, J = 8.5, 5.2, 3.3 Hz, 1H), 4.52 (t, J = 6.9 Hz, 1H), 4.12 (t, J = 6.3 Hz, 2H), 1.64 (t, *J* = 6.1 Hz, 1H). ^{13}C NMR (101 MHz, CDCl3) δ 158.9 (dd, J = 242.6, 2.4 Hz), 154.7 (dd, J = 245.9, 2.4 Hz), 152.6, 149.9, 139.7, 136.6, 131.4 (dd, J = 18.5, 7.4 Hz), 129.0, 128.5, 127.4, 124.8, 124.7, 123.0, 116.2 (dd, J = 24.8, 4.7 Hz), 115.7 (dd, J = 25.2, 3.5 Hz), 65.1, 46.6. ^{19}F NMR (376 MHz, CDCl3) δ -117.8 (dt, J = 17.7, 8.7 Hz), -127.2 (dt, J = 18.2, 5.4 Hz). m/z (ESI^{+}) HRMS: Calculated for $[C_{19}H_{16}F_2NO]^{+}$: 312.1194. Found $[M+H]^{+}$: 312.1205.

2-Phenyl-2-(3-(pyrimidin-2-yl)phenyl)ethan-1-ol 3m

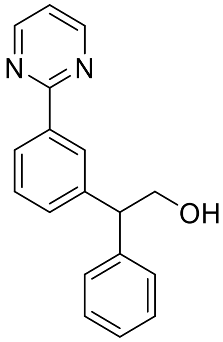

Obtained by using **General procedure A**: 2-phenylpyrimidine **1m** (31.2 mg, 0.20 mmol) and 2-phenyloxirane **2a** (46.0 uL, 0.40 mmol) were employed. The crude mixture was purified by column chromatography (50% EtOAc/Hex) to yield the title compound **3m** (48.8 mg, 88%) as a colorless oil; ^{1}H NMR (400 MHz, $CDCl_3$) δ 8.79 (d, *J* = 4.9 Hz, 2H),

8.41 (d, J = 1.9 Hz, 1H), 8.31 (dt, J = 7.8, 1.5 Hz, 1H), 7.45 (dd, J = 7.6 Hz, 1H), 7.41 – 7.36 (m, 1H), 7.32 (d, J = 5.2 Hz, 4H), 7.25 – 7.21 (m, 1H), 7.21 – 7.16 (m, 1H), 4.36 – 4.21 (m, 3H), 1.83 (s, br, 1H). ^{13}C NMR (101 MHz, $CDCl_3$) δ 164.6, 157.2, 141.9, 141.4, 137.9, 130.9, 129.0, 128.7, 128.3, 127.9, 126.8, 126.7, 119.1, 66.0, 53.7. m/z (ESI$^+$) HRMS: Calculated for $[C_{18}H_{17}N_2O]^+$: 277.1335. Found $[M+H]^+$: 277.1333.

2-(3-(1*H*-Pyrazol-1-yl)phenyl)-2-phenylethan-1-ol 3n

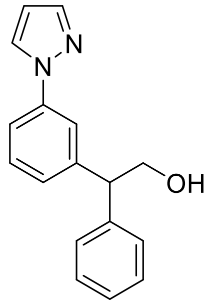

Obtained by using **General procedure A**: 1-phenyl-1H-pyrazole **1n** (28.8 mg, 0.20 mmol) and 2-phenyloxirane **2a** (46.0 uL, 0.40 mmol). The crude mixture was purified by column chromatography (35% EtOAc/Hex) to yield the title compound **3n** (47.5 mg, 90%) as a colorless oil; ^{1}H NMR (400 MHz, $CDCl_3$) δ 7.88 (d, J = 2.4 Hz, 1H), 7.69 (d, J = 1.8 Hz, 1H), 7.67 (d, J = 1.9 Hz, 1H), 7.50 (dd, J = 8.0, 2.1 Hz, 1H), 7.38 (dd, J = 7.9 Hz, 1H), 7.34 – 7.22 (m, 5H), 7.19 (d, J = 7.7 Hz, 1H), 6.44 (dd, J = 2.1 Hz, 2.1 Hz, 1H), 4.26 (dd, J = 7.2 Hz, 7.2 Hz, 1H), 4.23 – 4.14 (m, 2H), 2.09 (s, br, 1H). ^{13}C NMR (101 MHz, $CDCl_3$) δ 143.3, 141.0, 141.0, 140.3, 129.6, 128.7, 128.3, 126.9, 126.5, 119.4, 117.5, 107.6, 65.8, 53.5 *(One of the aromatic carbon signals is missing due to overlap)*. m/z (ESI$^+$) HRMS: Calculated for $[C_{17}H_{17}N_2O]^+$: 265.1335. Found $[M+H]^+$: 265.1330.

2-(3-(4,5-Dihydrooxazol-2-yl)phenyl)-2-phenylethan-1-ol 3o

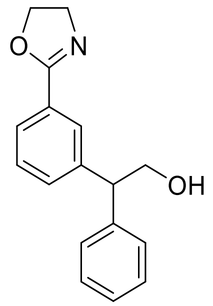

Obtained by using **General procedure A**: 2-phenyl-4,5-dihydrooxazole **1o** (29.4 mg, 0.20 mmol) and 2-phenyloxirane **2a** (46.0 uL, 0.40 mmol) were employed. The crude mixture was purified by column chromatography (80% EtOAc/Hex) to yield the title compound **3o** (28.3 mg, 53%) as a colorless oil; ^{1}H NMR (400 MHz, $CDCl_3$) δ 7.86 (d, *J* = 2.2 Hz, 1H), 7.73 (dd, *J* = 6.6, 2.0 Hz, 1H), 7.29 (d, *J* = 7.0 Hz, 2H), 7.26 – 7.21 (m, 2H), 7.21 – 7.12 (m, 3H), 4.35 (t, *J* = 9.5 Hz, 2H), 4.21 – 4.07 (m, 3H), 3.96 (t, *J* = 9.5 Hz, 2H), 2.01 (s, br, 1H). ^{13}C NMR (101 MHz, $CDCl_3$) δ 164.8, 141.9, 141.1, 131.7, 128.7, 128.3, 127.9, 127.8, 126.9, 126.6, 67.7, 65.8, 54.6, 53.5 *(One of the aromatic carbon signals is missing due to overlap)*. m/z (ESI$^+$) HRMS: Calculated for $[C_{17}H_{18}NO_2]^+$: 268.1332. Found $[M+H]^+$: 268.1339.

4-Phenyl-6-(pyridin-2-yl)isochroman-1-one 3p

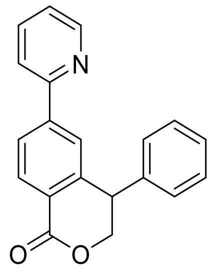

Obtained by using **General procedure A**: methyl 4-(pyridin-2-yl)benzoate **1p** (42.6 mg, 0.20 mmol) and 2-phenyloxirane **2a** (46.0 uL, 0.40 mmol). The crude mixture was purified by column chromatography (35% EtOAc/Hex) to yield the title compound **3p** (46.2 mg, 77%) as a colorless oil; ^{1}H NMR (400 MHz, $CDCl_3$) δ 8.67 (dd, *J* = 4.8, 1.4 Hz, 1H), 8.30 (d, *J* = 8.2 Hz, 1H), 8.05 (dd, *J* = 8.2, 1.8 Hz, 1H), 7.79 – 7.71 (m, 2H), 7.67 (dd, *J* = 8.0, 1.1 Hz, 1H), 7.41 – 7.31 (m, 3H), 7.30 – 7.19 (m, 3H), 4.72 (dd, *J* = 11.0, 4.6 Hz, 1H), 4.64 (dd, *J* = 11.0, 7.3 Hz, 1H), 4.45 (dd, *J* = 7.3, 4.6 Hz, 1H). ^{13}C NMR (101 MHz, $CDCl_3$) δ 164.8, 155.6, 149.8, 144.5, 142.8, 138.3, 137.1, 131.0, 129.1, 128.6, 127.8, 126.4, 126.1, 125.3, 123.2, 121.2, 72.3, 43.7. m/z (ESI$^+$) HRMS: Calculated for $[C_{20}H_{16}NO_2]^+$: 302.1176. Found $[M+H]^+$: 302.1180.

When using 4-(pyridin-2-yl)benzaldehyde **1q** (36.6 mg, 0.20 mmol) and 2-phenyloxirane **2a** (46.0 uL, 0.40 mmol) as substrates under **General procedure A**, the same compound **3p** (27.1 mg, 45%) was obtained.

2-(4-Cyclohexylphenyl)-2-(3-(pyridin-2-yl)phenyl)ethan-1-ol 3q

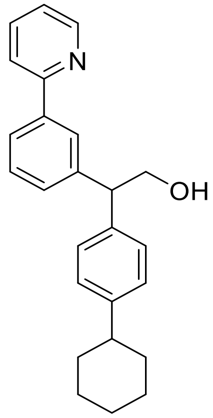

Obtained by using **General procedure A**: 2-Phenylpyridine **1a** (32.0 uL, 0.20 mmol) and 2-(4-cyclohexylphenyl)oxirane **2o** (80.8 mg, 0.40 mmol) were employed. The crude mixture was purified by column chromatography (40% EtOAc/Hex) to yield the title compound **3q** (60.0 mg, 84%) as a colorless oil; ^{1}H NMR (400 MHz, $CDCl_3$) δ 8.64 (d, *J* = 4.8 Hz, 1H), 7.94 (s, 1H), 7.79 (d, *J* = 7.8 Hz, 1H), 7.76 – 7.70 (m, 1H), 7.68 (d, *J* = 7.8 Hz, 1H), 7.43– 7.39 (m, 1H), 7.31 (d, *J* = 7.7 Hz, 1H), 7.23 – 7.17 (m, 3H), 7.15– 7.13 (m, 2H), 4.27 (app. t, *J* = 7.2 Hz, 1H), 4.24 – 4.14 (m, 2H), 2.60 (s, br, 1H), 2.55 – 2.37 (m, 1H), 1.91 – 1.77 (m, 4H), 1.73 (d, *J* = 12.4 Hz, 1H), 1.40– 1.35 (m, 4H), 1.29 – 1.18 (m, 1H). ^{13}C NMR (101 MHz, $CDCl_3$) δ 157.5, 149.6, 146.5, 142.4, 139.6, 138.7, 136.9, 129.2, 129.1, 128.2, 127.1, 127.1, 125.4, 122.2, 120.9, 66.1, 53.5, 44.1, 34.5, 26.9, 26.2. m/z (ESI^{+}) HRMS: Calculated for $[C_{25}H_{28}NO]^{+}$: 358.2165. Found $[M+H]^{+}$: 358.2159.

2-(4-(*tert*-Butyl)phenyl)-2-(3-(pyridin-2-yl)phenyl)ethan-1-ol 3r

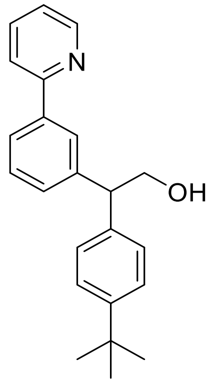

Obtained by using **General procedure A**: 2-Phenylpyridine **1a** (32.0 uL, 0.20 mmol) and 2-(4-(tert-butyl)phenyl)oxirane **2p** (68.0 mg, 0.40 mmol) were employed. The crude mixture was purified by column chromatography (40% EtOAc/Hex) to yield the title compound **3r** (49.0 mg, 74%) as a colorless oil; ^{1}H NMR (400 MHz, $CDCl_3$) δ 8.60 (d, *J* = 4.8 Hz, 1H), 7.93 (s, 1H), 7.77 (d, *J* = 7.7 Hz, 1H), 7.72– 7.64 (m, 2H), 7.40– 7.36 (m, 1H), 7.31– 7.29 (m, 3H), 7.20– 7.16 (m, 3H), 4.26 (app. t, *J* = 7.2, 1H), 4.22 – 4.11 (m, 2H), 2.80 (s, br, 1H), 1.27 (s, 9H). ^{13}C NMR (101 MHz, CDCl3) δ 157.5, 149.5, 149.4, 142.4, 139.6, 138.4, 136.9, 129.2, 129.1, 127.9, 127.0, 125.6, 125.4, 122.2, 120.9, 66.0, 53.4, 34.4, 31.4. m/z (ESI$^+$) HRMS: Calculated for $[C_{23}H_{25}NNaO]^+$: 354.1828. Found $[M+Na]^+$: 354.1819.

2-([1,1'-Biphenyl]-4-yl)-2-(3-(pyridin-2-yl)phenyl)ethan-1-ol 3s

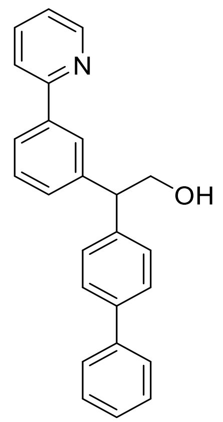

Obtained by using **General procedure A**: 2-Phenylpyridine **1a** (32.0 uL, 0.20 mmol) and 2-([1,1'-biphenyl]-4-yl)oxirane **2q** (78.4 mg, 0.40 mmol) were employed. The crude mixture was purified by column chromatography (40% EtOAc/Hex) to yield the title compound **3s** (56.0 mg, 80%) as a colorless oil; ^{1}H NMR (400 MHz, $CDCl_3$) δ 8.64 (d, *J* = 4.5 Hz, 1H), 8.00 (s, 1H), 7.82 (d, *J* = 7.7 Hz, 1H), 7.72 – 7.70 (m, 2H), 7.61 – 7.50 (m, 4H), 7.45 – 7.41 (m, 3H), 7.38 – 7.32 (m, 4H), 7.23 – 7.19 (m, 1H), 4.36 (app. t, *J* = 7.2 Hz, 1H), 4.30 – 4.20 (m, 2H), 3.13 (s, br, 1H). ^{13}C NMR (101 MHz, $CDCl_3$) δ 157.4, 149.5, 142.3, 140.8, 139.6, 139.5, 136.9, 129.2, 129.1, 128.8, 128.8, 127.3, 127.2, 127.0, 125.5, 122.2, 120.9, 65.9, 53.5. *(Two of the aromatic carbon signals are missing due to overlap).* m/z (ESI$^+$) HRMS: Calculated for $[C_{25}H_{22}NO]^+$: 352.1696. Found $[M+H]^+$:352.1694.

2-(4-Fluorophenyl)-2-(3-(pyridin-2-yl)phenyl)ethan-1-ol 3t

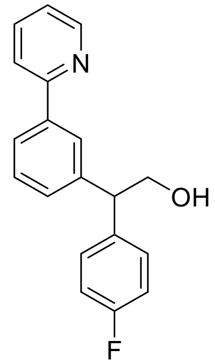

Obtained by using **General procedure A**: 2-Phenylpyridine **1a** (32.0 uL, 0.20 mmol) and 2-(4-fluorophenyl)oxirane **2r** (55.2 mg, 0.40 mmol) were employed. The crude mixture was purified by column chromatography (40% EtOAc/Hex) to yield the title compound **3t** (33.0 mg, 57%) as a colorless oil; ^{1}H NMR (400 MHz, $CDCl_3$) δ 8.63 (d, J = 4.2 Hz, 1H), 7.90 (s, 1H), 7.78 (d, J = 7.8 Hz, 1H), 7.75 – 7.71 (m, 1H), 7.66 (d, J = 7.9 Hz, 1H), 7.42 – 7.38 (m, 1H), 7.26 – 7.20 (m, 4H), 6.99 – 6.95 (m, 2H), 4.28 – 4.11 (m, 3H), 2.60 (s, br, 1H). ^{13}C NMR (101 MHz, $CDCl_3$) δ 161.7 (d, J = 245.1 Hz), 157.4, 149.6, 141.0 (d, J = 227.6 Hz), 137.4 (d, J = 3.3 Hz), 136.9, 129.9 (d, J = 7.9 Hz), 129.2, 129.1, 127.0, 125.6, 122.3, 120.9, 115.6, 115.4, 66.0, 53.0. ^{19}F NMR (376 MHz, $CDCl_3$) δ -116.2 (s). m/z (ESI$^+$) HRMS: Calculated for $[C_{19}H_{17}FNO]^+$: 294.1289. Found $[M+H]^+$: 294.1281.

2-(4-Chlorophenyl)-2-(3-(pyridin-2-yl)phenyl)ethan-1-ol 3u

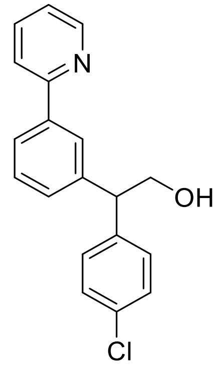

Obtained by using **General procedure A**: 2-Phenylpyridine **1a** (32.0 uL, 0.20 mmol) and 2-(4-chlorophenyl)oxirane **2s** (61.6 mg, 0.40 mmol) were employed. The crude mixture was purified by column chromatography (40% EtOAc/Hex) to yield the title compound **3u**

(50.0 mg, 81%) as a colorless oil; ^{1}H NMR (400 MHz, $CDCl_3$) δ 8.60 (d, J = 4.2 Hz, 1H), 7.88 (s, 1H), 7.80 – 7.69 (m, 2H), 7.66 (d, J = 8.0 Hz, 1H), 7.41 – 7.37 (m, 1H), 7.26 – 7.15 (m, 6H), 4.26 – 4.08 (m, 3H), 3.16 (s, br, 1H). ^{13}C NMR (101 MHz, $CDCl_3$) δ 157.3, 149.5, 141.9, 140.2, 139.7, 137.0, 132.5, 129.8, 129.2, 128.7, 126.9, 125.6, 122.3, 121.0, 65.7, 53.1. *(One of the aromatic carbon signals is missing due to overlap)* m/z (ESI^+) HRMS: Calculated for $[C_{19}H_{17}ClNO]^+$: 310.0993. Found $[M+H]^+$: 310.0993.

2-(4-Bromophenyl)-2-(3-(pyridin-2-yl)phenyl)ethan-1-ol 3v

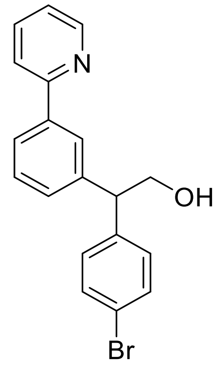

Obtained by using **General procedure A**: 2-Phenylpyridine **1a** (32.0 uL, 0.20 mmol) and 2-(4-bromophenyl)oxirane **2t** (79.2 mg, 0.40 mmol) were employed. The crude mixture was purified by column chromatography (40% EtOAc/Hex) to yield the title compound **3v** (58.0 mg, 82%) as a colorless oil; ^{1}H NMR (400 MHz, $CDCl_3$) δ 8.61 (d, J = 4.8 Hz, 1H), 7.89 (s, 1H), 7.78 (d, J = 7.8 Hz, 1H), 7.76 – 7.71 (m, 1H), 7.66 (d, J = 7.9 Hz, 1H), 7.42 – 7.38 (m, 3H), 7.24 (d, J = 7.6 Hz, 1H), 7.22 – 7.19 (m, 1H), 7.15 – 7.13 (m, 2H), 4.25 – 4.09 (m, 3H), 2.72 (s, br, 1H). ^{13}C NMR (101 MHz, $CDCl_3$) δ 157.3, 149.6, 141.7, 140.8, 139.8, 137.0, 131.7, 130.2, 129.2, 129.1, 127.0, 125.6, 122.3, 120.9, 120.7, 65.7, 53.2. m/z (ESI^+) HRMS: Calculated for $[C_{19}H_{17}BrNO]^+$: 354.0488. Found $[M+H]^+$: 354.0485.

2-(3-(Pyridin-2-yl)phenyl)-2-(4-(trifluoromethyl)phenyl)ethan-1-ol 3w

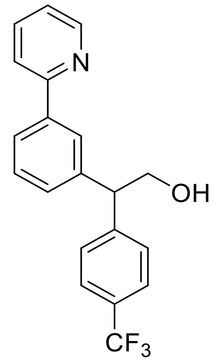

Obtained by using **General procedure A**: 2-Phenylpyridine **1a** (32.0 uL, 0.20 mmol) and 2-(4-(trifluoromethyl)phenyl)oxirane **2u** (75.2 mg, 0.40 mmol) were employed. The crude mixture was purified by column chromatography (40% EtOAc/Hex) to yield the title compound **3w** (44.0 mg, 76%) as a colorless oil; ^{1}H NMR (400 MHz, $CDCl_3$) δ 8.61 (d, *J* = 4.8 Hz, 1H), 7.91 (s, 1H), 7.78 (d, *J* = 7.8 Hz, 1H), 7.76 – 7.72 (m, 1H), 7.67 (d, *J* = 8.0 Hz, 1H), 7.53 (d, *J* = 8.2 Hz, 2H), 7.43 – 7.37 (m, 3H), 7.26 – 7.23 (m, 1H), 7.23 – 7.18 (m, 1H), 4.33 (app. t, *J* = 7.0, Hz, 1H), 4.26 – 4.15 (m, 2H), 2.77 (s, br, 1H). ^{13}C NMR (101 MHz, $CDCl_3$) δ 157.3, 149.6, 145.9, 141.5, 139.9, 137.0, 129.3 (d, *J* = 11.6 Hz), 128.8, 128.5 (q, *J* = 23.5 Hz), 127.0, 125.8, 125.6 (q, *J* = 3.8 Hz), 124.3 (q, *J* = 271.0 Hz), 122.4, 121.0, 65.7, 53.6. *(One of the aromatic carbon signals is missing due to overlap)*. ^{19}F NMR (376 MHz, $CDCl_3$) δ -62.4 (s). m/z (ESI$^+$) HRMS: Calculated for $[C_{20}H_{17}F_3NO]^+$: 344.1257. Found $[M+H]^+$: 344.1253.

Methyl 4-(2-hydroxy-1-(3-(pyridin-2-yl)phenyl)ethyl)benzoate 3x

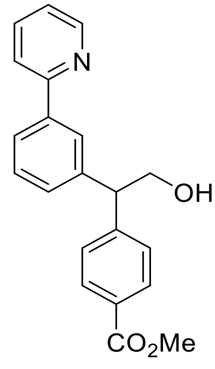

Obtained by using **General procedure A**: 2-Phenylpyridine **1a** (32.0 uL, 0.20 mmol) and methyl 4-(oxiran-2-yl)benzoate **2v** (71.2 mg, 0.40 mmol) were employed. The crude mixture was purified by column chromatography (40% EtOAc/Hex) to yield the title

compound **3x** (54.0 mg, 82%) as a colorless oil; ^{1}H NMR (400 MHz, $CDCl_3$) δ 8.61 –8.59 (m, 1H), 7.94 (d, J = 8.4 Hz, 2H), 7.89 (s, 1H), 7.77 (d, J = 7.9 Hz, 1H), 7.74 – 7.69 (m, 1H), 7.65 (d, J = 8.0 Hz, 1H), 7.41 – 7.37 (m, 1H), 7.33 (d, J = 8.3 Hz, 2H), 7.24 (d, J = 7.7 Hz, 1H), 7.22 – 7.19 (m, 1H), 4.32 (app. t, J = 7.0 Hz, 1H), 4.25 – 4.12 (m, 2H), 3.87 (s, 3H), 3.18 (s, br, 1H). ^{13}C NMR (101 MHz, CDCl3) δ 167.0, 157.3, 149.6, 147.1, 141.6, 139.8, 137.0, 129.9, 129.2, 128.6, 128.5, 127.0, 125.6, 122.3, 120.9, 65.6, 53.7, 52.1. *(One of the aromatic carbon signals is missing due to overlap).* m/z (ESI$^+$) HRMS: Calculated for $[C_{21}H_{20}NO_3]^+$: 334.1438. Found $[M+H]^+$: 334.1442.

2-(3-(Pyridin-2-yl)phenyl)-2-(o-tolyl)ethan-1-ol 3y

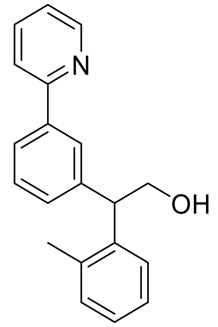

Obtained by using **General procedure A**: 2-Phenylpyridine **1a** (32.0 uL, 0.20 mmol) and 2-(o-tolyl)oxirane **2w** (53.6 mg, 0.40 mmol) were employed. The crude mixture was purified by column chromatography (40% EtOAc/Hex) to yield the title compound **3y** (54.0 mg, 93%) as a colorless oil; ^{1}H NMR (400 MHz, $CDCl_3$) δ 8.63 (d, J = 4.8 Hz, 1H), 7.89 (s, 1H), 7.79 (d, J = 7.8 Hz, 1H), 7.75 – 7.71 (m, 1H), 7.66 (d, J = 8.0 Hz, 1H), 7.40 – 7.36 (m, 1H), 7.33 (d, J = 7.6 Hz, 1H), 7.25 – 7.19 (m, 3H), 7.17 – 7.14 (m, 2H), 4.49 (app. t, J = 7.1 Hz, 1H), 4.20 – 4.18 (m, 2H), 2.45 (s, br, 1H), 2.29 (s, 3H). ^{13}C NMR (101 MHz, $CDCl_3$) δ 157.5, 149.6, 141.9, 139.6, 139.4, 137.1, 136.9, 130.9, 129.4, 129.1, 127.3, 126.8, 126.7, 126.3, 125.4, 122.2, 121.0, 65.9, 49.7, 20.0. m/z (ESI$^+$) HRMS: Calculated for $[C_{20}H_{20}NO]^+$: 290.1539. Found $[M+H]^+$: 290.1535.

2-(2-Methoxyphenyl)-2-(3-(pyridin-2-yl)phenyl)ethan-1-ol 3z

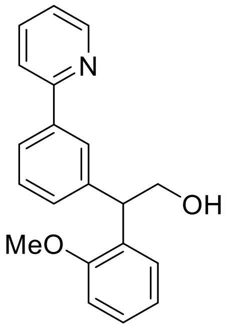

Obtained by using **General procedure A**: 2-Phenylpyridine **1a** (32.0 uL, 0.20 mmol) and 2-(2-methoxyphenyl)oxirane **2x** (60.0 mg, 0.40 mmol) were employed. The crude mixture was purified by column chromatography (40% EtOAc/Hex) to yield the title compound **3z** (61.0 mg, 98%) as a colorless oil; ^{1}H NMR (400 MHz, $CDCl_3$) δ 8.64 (d, *J* = 4.5 Hz, 1H), 7.94 (s, 1H), 7.80 (d, *J* = 7.6 Hz, 1H), 7.75 – 7.69 (m, 1H), 7.67 (d, *J* = 7.8 Hz, 1H), 7.42 – 7.38 (m, 1H), 7.34 (d, *J* = 7.7 Hz, 1H), 7.22 – 7.18 (m, 3H), 6.94 – 6.84 (m, 2H), 4.78 (app. t, *J* = 7.1 Hz, 1H), 4.22 – 4.15 (m, 2H), 3.79 (s, 3H), 2.56 (s, br, 1H). ^{13}C NMR (101 MHz, $CDCl_3$) δ 157.6, 157.3, 149.6, 142.2, 139.5, 136.8, 129.9, 129.5, 128.9, 128.5, 127.8, 127.3, 125.2, 122.1, 120.9, 120.7, 110.8, 65.1, 55.5, 46.5. m/z (ESI$^+$) HRMS: Calculated for $[C_{20}H_{20}NO_2]^+$: 306.1489. Found $[M+H]^+$: 306.1499.

2-(2-Fluorophenyl)-2-(3-(pyridin-2-yl)phenyl)ethan-1-ol 3aa

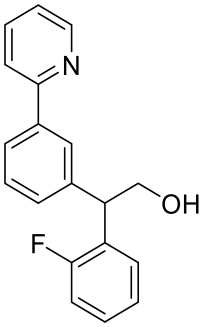

Obtained by using **General procedure A**: 2-Phenylpyridine **1a** (32.0 uL, 0.20 mmol) and 2-(2-fluorophenyl)oxirane **2y** (55.2 mg, 0.40 mmol) were employed. The crude mixture was purified by column chromatography (40% EtOAc/Hex) to yield the title compound **3aa** (58.0 mg, 98%) as a colorless oil; ^{1}H NMR (400 MHz, $CDCl_3$) δ 8.65 – 8.63 (m, 1H), 7.93 (s, 1H), 7.83 – 7.77 (m, 1H), 7.76 – 7.70 (m, 1H), 7.68 – 7.66 (m, 1H), 7.43 – 7.39 (m, 1H), 7.33 – 7.28 (m, 2H), 7.24 – 7.16 (m, 2H), 7.10 – 7.06 (m, 1H), 7.05 – 6.99 (m,

1H), 4.62 (app. t, J = 7.2 Hz, 1H), 4.30 – 4.09 (m, 2H), 2.64 (s, br, 1H). ^{13}C NMR (101 MHz, $CDCl_3$) δ 161.0 (d, J = 246.0 Hz), 157.4, 149.6, 141.2, 139.7, 137.0, 129.3 (d, J = 2.1 Hz), 129.2, 129.2, 128.6 (d, J = 14.6 Hz), 128.4 (d, J = 8.4 Hz), 127.1, 125.7, 124.3 (d, J = 3.6 Hz), 122.3, 121.0, 115.7 (d, J = 22.5 Hz), 64.9, 46.7. ^{19}F NMR (376 MHz, $CDCl_3$) δ -117.0 (s). m/z (ESI^+) HRMS: Calculated for $[C_{19}H_{17}FNO]^+$: 294.1289. Found $[M+H]^+$: 294.1298.

2-(3-Methoxyphenyl)-2-(3-(pyridin-2-yl)phenyl)ethan-1-ol 3ab

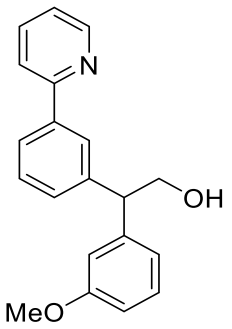

Obtained by using **General procedure A**: 2-Phenylpyridine **1a** (32.0 uL, 0.20 mmol) and 2-(3-methoxyphenyl)oxirane **2z** (60.0 mg, 0.40 mmol) were employed. The crude mixture was purified by column chromatography (40% EtOAc/Hex) to yield the title compound **3ab** (74.0 mg, 99%) as a colorless oil; ^{1}H NMR (400 MHz, $CDCl_3$) δ 8.63 (d, J = 4.8 Hz, 1H), 7.92 (s, 1H), 7.79 (d, J = 7.7 Hz, 1H), 7.74 – 7.70 (m, 1H), 7.66 (d, J = 7.9 Hz, 1H), 7.41 (d, J = 7.7 Hz, 1H), 7.30 (d, J = 7.7 Hz, 1H), 7.23 (d, J = 7.9 Hz, 1H), 7.21 – 7.19 (m, 1H), 6.88 (d, J = 7.7 Hz, 1H), 6.84 (s, 1H), 6.75 (dd, J = 8.2, 2.0 Hz, 1H), 4.27 (app. t, J = 7.1 Hz, 1H), 4.23 – 4.15 (m, 2H), 3.75 (s, 3H), 2.71 (s, br, 1H). ^{13}C NMR (101 MHz, $CDCl_3$) δ 159.8, 157.4, 149.5, 143.2, 142.1, 139.6, 136.9, 129.7, 129.1, 129.1, 127.0, 125.5, 122.2, 120.9, 120.7, 114.6, 111.8, 65.9, 55.2, 53.8. m/z (ESI^+) HRMS: Calculated for $[C_{20}H_{20}NO_2]^+$: 306.1489. Found $[M+H]^+$: 306.1487.

2-(3-Bromophenyl)-2-(3-(pyridin-2-yl)phenyl)ethan-1-ol 3ac

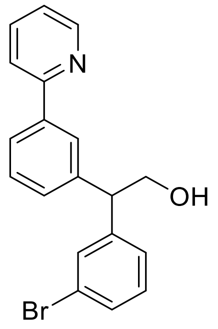

Obtained by using **General procedure A**: 2-Phenylpyridine **1a** (32.0 uL, 0.20 mmol) and 2-(3-bromophenyl)oxirane **2aa** (79.2 mg, 0.40 mmol) were employed. The crude mixture was purified by column chromatography (40% EtOAc/Hex) to yield the title compound **3ac** (70.0 mg, 98%) as a colorless oil; ^{1}H NMR (400 MHz, $CDCl_3$) δ 8.61 (d, J = 4.8 Hz, 1H), 7.88 (s, 1H), 7.78 (d, J = 7.9 Hz, 1H), 7.75 – 7.71 (m, 1H), 7.66 (d, J = 7.9 Hz, 1H), 7.43 – 7.37 (m, 2H), 7.35 – 7.29 (m, 1H), 7.26 – 7.18 (m, 3H), 7.16 – 7.12 (m, 1H), 4.25 – 4.10 (m, 3H), 2.82 (s, br, 1H). ^{13}C NMR (101 MHz, $CDCl_3$) δ 157.3, 149.6, 144.1, 141.5, 139.8, 137.0, 131.5, 130.2, 129.9, 129.2, 129.1, 127.1, 127.0, 125.7, 122.8, 122.3, 121.0, 65.7, 53.4. m/z (ESI^{+}) HRMS: Calculated for $[C_{19}H_{17}BrNO]^{+}$: 354.0488. Found $[M+H]^{+}$: 354.0483.

2-(3-Chlorophenyl)-2-(3-(pyridin-2-yl)phenyl)ethan-1-ol 3ad

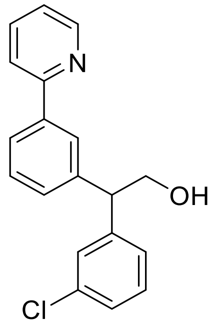

Obtained by using **General procedure A**: 2-Phenylpyridine **1a** (32.0 uL, 0.20 mmol) and 2-(3-chlorophenyl)oxirane **2ab** (62.0 mg, 0.40 mmol) were employed. The crude mixture was purified by column chromatography (40% EtOAc/Hex) to yield the title compound **3ad** (47.0 mg, 76%) as a colorless oil; ^{1}H NMR (400 MHz, $CDCl_3$) δ 8.63 – 8.61 (m, 1H), 7.89 (s, 1H), 7.78 (d, J = 8.1 Hz, 1H), 7.76 – 7.69 (m, 1H), 7.67 (d, J = 7.9 Hz, 1H), 7.42 – 7.39 (m, 1H), 7.26 – 7.25 (m, 2H), 7.23 – 7.21 (m, 1H), 7.20 – 7.12 (m, 3H), 4.25 (app.

t, J = 7.0 Hz, 1H), 4.22 – 4.10 (m, 2H), 2.69 (s, br, 1H). ^{13}C NMR (101 MHz, $CDCl_3$) δ 157.3, 149.6, 143.8, 141.6, 139.8, 137.0, 134.5, 129.9, 129.3, 129.1, 128.6, 127.1, 127.0, 126.7, 125.7, 122.3, 121.0, 65.7, 53.5. m/z (ESI$^+$) HRMS: Calculated for $[C_{19}H_{17}ClNO]^+$: 310.0993. Found $[M+H]^+$: 310.0997.

5-(2-Hydroxy-1-(3-(pyridin-2-yl)phenyl)ethyl)-2,3-dihydro-1*H*-inden-1-one 3ae

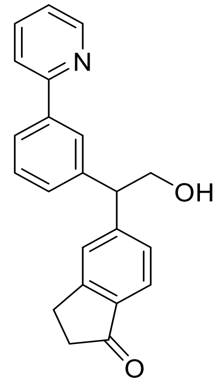

Obtained by using **General procedure A**: 2-Phenylpyridine **1a** (32.0 uL, 0.20 mmol) and 5-(oxiran-2-yl)-2,3-dihydro-1H-inden-1-one **2ac** (70.0 mg, 0.40 mmol) were employed. The crude mixture was purified by column chromatography (40% EtOAc/Hex) to yield the title compound 5-(2-hydroxy-1-(3-(pyridin-2-yl)phenyl)ethyl)-2,3-dihydro-1*H*-inden-1-one **3ae** (63.0 mg, 96%) as a colorless oil; ^{1}H NMR (400 MHz, $CDCl_3$) δ 8.59 (d, J = 4.3 Hz, 1H), 7.88 (s, 1H), 7.79 – 7.68 (m, 2H), 7.65 – 7.60 (m, 3H), 7.39 – 7.33 (m, 2H), 7.22 – 7.20 (m, 2H), 4.43 (app. t, J = 7.0 Hz, 1H), 4.22 (app. d, J = 5.3 Hz, 2H), 3.34 (s, br, 1H), 3.10 – 2.98 (m, 1H), 2.88 – 2.76 (m, 1H), 2.57 – 2.53 (m, 2H). ^{13}C NMR (101 MHz, $CDCl_3$) δ 207.4, 157.1, 154.7, 149.6, 141.0, 139.7, 139.6, 137.4, 137.0, 132.7, 129.2, 127.9, 127.1, 125.7, 122.4, 122.1, 120.9, 65.3, 49.6, 36.1, 24.7. *(One of the aromatic carbon signals is missing due to overlap).* m/z (ESI$^+$) HRMS: Calculated for $[C_{22}H_{20}NO_2]^+$: 330.1489. Found $[M+H]^+$: 330.1481.

2-(Naphthalen-1-yl)-2-(3-(pyridin-2-yl)phenyl)ethan-1-ol 3af

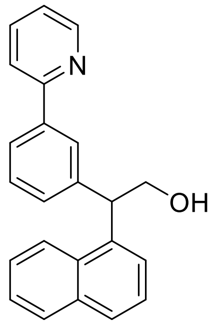

Obtained by using **General procedure A**: 2-Phenylpyridine **1a** (32.0 uL, 0.20 mmol) and 2-(naphthalen-1-yl)oxirane **2ad** (68.0 mg, 0.40 mmol) were employed. The crude mixture was purified by column chromatography (40% EtOAc/Hex) to yield the title compound **3af** (55.0 mg, 85%) as a colorless oil; ^{1}H NMR (400 MHz, $CDCl_3$) δ 8.60 (d, *J* = 4.8 Hz, 1H), 8.12 (dd, *J* = 6.2, 3.5 Hz, 1H), 8.01 (s, 1H), 7.84 (dd, *J* = 6.1, 3.4 Hz, 1H), 7.78 – 7.76 (m, 2H), 7.71 – 7.67 (m, 1H), 7.63 (d, *J* = 7.9 Hz, 1H), 7.50 – 7.41 (m, 4H), 7.38 – 7.34 (m, 1H), 7.30 (d, *J* = 7.8 Hz, 1H), 7.19 – 7.16 (m, 1H), 5.09 (app. t, *J* = 6.9 Hz, 1H), 4.39 – 4.24 (m, 2H), 2.93 (s, br, 1H). ^{13}C NMR (101 MHz, $CDCl_3$) δ 157.4, 149.6, 142.4, 139.7, 137.0, 136.8, 134.2, 132.1, 129.3, 129.1, 128.9, 127.6, 127.2, 126.3, 125.6, 125.5, 125.5, 124.9, 123.7, 122.2, 120.9, 66.0, 49.2. m/z (ESI$^+$) HRMS: Calculated for $[C_{23}H_{19}NNaO]^+$: 348.1359. Found $[M+Na]^+$: 348.1352.

2-([1,1'-Binaphthalen]-2-yl)-2-(3-(pyridin-2-yl)phenyl)ethan-1-ol 3ag

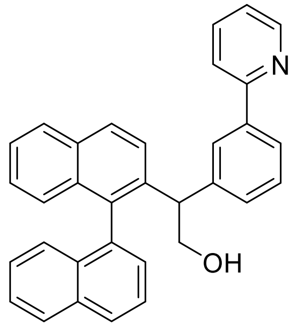

Obtained by using **General procedure A**: **1a** (32.0 uL, 0.20 mmol) and 2-([1,1'-binaphthalen]-2-yl)oxirane **2ae** (59.2 mg, 0.40 mmol) were employed. The crude mixture was purified by column chromatography (40% EtOAc/Hex) to yield the title compound **3ag** (38.0 mg, 42%) as a colorless oil. *Due to the introduction of two stereocenters, **3ag***

was obtained as a mixture of two diastereoisomers (1:1 ratio), which could not be separated by column chromatography.

The mixture of two diastereoisomers: ^{1}H NMR (400 MHz, $CDCl_3$) δ 8.60 – 8.54 (m, 2H), 8.00 – 7.95 (m, 4H), 7.94 – 7.83 (m, 4H), 7.78 – 7.75 (m, 2H), 7.72 – 7.47 (m, 10H), 7.44 – 7.37 (m, 4H), 7.36 – 7.29 (m, 4H), 7.23 – 7.14 (m, 6H), 7.13 – 6.99 (m, 6H), 4.29 – 4.11 (m, 4H), 4.10 – 4.07 (m, 2H), 2.27 (m, br, 2H). ^{13}C NMR (101 MHz, $CDCl_3$) δ 157.5, 157.3, 149.6, 149.4, 142.4, 141.6, 139.4, 139.2, 137.7, 137.6, 137.4, 137.1, 136.8, 136.6, 136.6, 136.4, 133.7, 133.7, 133.6, 133.1, 133.1, 132.3, 132.2, 129.4, 129.0, 128.9, 128.8, 128.8, 128.4, 128.4, 128.3, 128.2, 128.1, 127.8, 127.8, 127.3, 127.0, 127.0, 126.8, 126.6, 126.5, 126.2, 126.2, 126.1, 126.1, 126.0, 125.9, 125.7, 125.7, 125.4, 125.4, 125.3, 125.2, 125.1, 122.2, 122.1, 120.8, 65.7, 65.6, 50.5, 50.2. m/z (ESI$^+$) HRMS: Calculated for $[C_{33}H_{26}NO]^+$: 452.2009. Found $[M+H]^+$: 452.2011.

2-(Furan-3-yl)-2-(3-(pyridin-2-yl)phenyl)ethan-1-ol 3ah

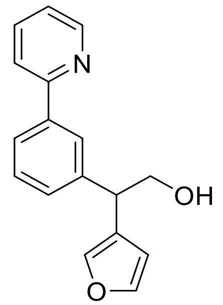

Obtained by using **General procedure C**: 2-Phenylpyridine **1a** (32.0 uL, 0.20 mmol) and 3-(oxiran-2-yl)furan **2af** (88.0 mg, 0.80 mmol) were employed. The crude mixture was purified by column chromatography (40% EtOAc/Hex) to yield the title compound **3ah** (28.0 mg, 53%) as a colorless oil; ^{1}H NMR (400 MHz, $CDCl_3$) δ 8.67 (d, J = 4.8 Hz, 1H), 7.92 (s, 1H), 7.84 (d, J = 7.9 Hz, 1H), 7.77 – 7.73 (m, 1H), 7.69 (d, J = 7.9 Hz, 1H), 7.46 – 7.42 (m, 1H), 7.39 (s, 1H), 7.35 (s, 1H), 7.31 (d, J = 7.6 Hz, 1H), 7.23 (dd, J = 6.7, 5.4 Hz, 1H), 6.32 (s, 1H), 4.18 – 4.11 (m, 1H), 4.08 – 4.07 (m, 2H), 1.91 (s, br, 1H). ^{13}C NMR (101 MHz, $CDCl_3$) δ 157.4, 149.7, 143.3, 141.7, 139.9, 139.9, 137.0, 129.2, 129.0, 127.1, 125.8, 125.4, 122.3, 120.9, 110.6, 66.4, 45.4. m/z (ESI$^+$) HRMS: Calculated for $[C_{17}H_{16}NO_2]^+$: 266.1176. Found $[M+H]^+$: 266.1172.

2-(3-(Pyridin-2-yl)phenyl)-2-(thiophen-3-yl)ethan-1-ol 3ai

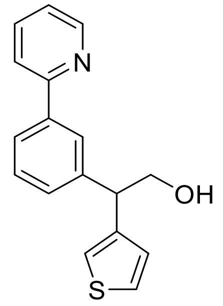

Obtained by using **General procedure C**: 2-Phenylpyridine **1a** (32.0 uL, 0.20 mmol) and 2-(thiophen-3-yl)oxirane **2ag** (100.8 mg, 0.80 mmol) were employed. The crude mixture was purified by column chromatography (40% EtOAc/Hex) to yield the title compound **3ai** (36.0 mg, 65%) as a colorless oil; ^{1}H NMR (400 MHz, $CDCl_3$) δ 8.66 (d, J = 4.6 Hz, 1H), 7.92 (s, 1H), 7.83 (d, J = 7.7 Hz, 1H), 7.76 – 7.72 (m, 1H), 7.68 (d, J = 7.9 Hz, 1H), 7.45 – 7.41 (m, 1H), 7.32 – 7.26 (m, 2H), 7.25 – 7.19 (m, 1H), 7.11 (d, J = 2.5 Hz, 1H), 6.99 (d, J = 4.9 Hz, 1H), 4.35 (app. t, J = 7.0 Hz, 1H), 4.23 – 4.12 (m, 2H), 2.04 (s, br, 1H). ^{13}C NMR (101 MHz, $CDCl_3$) δ 157.4, 149.7, 142.2, 142.0, 139.9, 136.9, 129.2, 129.0, 127.9, 127.1, 126.0, 125.7, 122.3, 121.6, 120.9, 66.5, 49.7. m/z (ESI$^+$) HRMS: Calculated for $[C_{17}H_{16}NOS]^+$: 282.0947. Found $[M+H]^+$: 282.0943.

2-(3-(Pyridin-2-yl)phenyl)-2-(quinolin-6-yl)ethan-1-ol 3aj

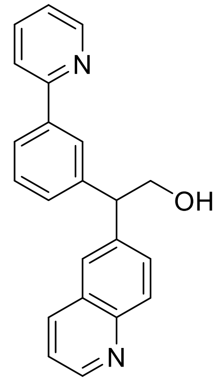

Obtained by using **General procedure A**: 2-Phenylpyridine **1a** (32.0 uL, 0.20 mmol) and 2-(3-methoxyphenyl)oxirane **2ah** (60.0 mg, 0.40 mmol) were employed. The crude mixture was purified by column chromatography (40% EtOAc/Hex) to yield the title compound **3aj** (74.0 mg, 99%) as a colorless oil; ^{1}H NMR (400 MHz, $CDCl_3$) δ 8.80 (dd,

J = 4.2, 1.6 Hz, 1H), 8.62 (dd, J = 4.8, 0.7 Hz, 1H), 8.06 (d, J = 7.6 Hz, 1H), 7.98 – 7.95 (m, 2H), 7.78 (d, J = 7.8 Hz, 1H), 7.74 – 7.68 (m, 2H), 7.65 (d, J = 7.9 Hz, 1H), 7.59 (dd, J = 8.8, 1.9 Hz, 1H), 7.42– 7.39 (m, 1H), 7.35 – 7.29 (m, 2H), 7.21 – 7.18 (m, 1H), 4.48 (app. t, J = 7.0 Hz, 1H), 4.37 – 4.27 (m, 2H), 3.08 (s, br, 1H). ^{13}C NMR (101 MHz, $CDCl_3$) δ 157.3, 150.1, 149.6, 147.2, 141.9, 140.2, 139.9, 137.0, 136.2, 130.7, 129.6, 129.3, 128.3, 127.2, 126.7, 125.7, 122.3, 121.3, 120.9, 65.8, 53.7. *(One of the aromatic carbon signals is missing due to overlap).* m/z (ESI$^+$) HRMS: Calculated for $[C_{22}H_{19}N_2O]^+$: 327.1492. Found $[M+H]^+$: 327.1495.

4-(3-(Pyridin-2-yl)phenyl)isochroman-1-one 3ak

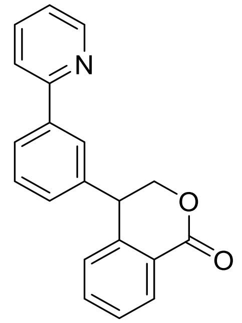

Obtained by using **General procedure B**: 2-Phenylpyridine **1a** (32.0 uL, 0.20 mmol) and methyl 2-(oxiran-2-yl)benzoate **2ai** (142.4 mg, 0.80 mmol) were employed. The crude mixture was purified by column chromatography (35% EtOAc/Hex) to yield the title compound **3ak** (25.0 mg, 41%) as a colorless oil; ^{1}H NMR (400 MHz, $CDCl_3$) δ 8.69 (d, J = 4.3 Hz, 1H), 8.20 (dd, J = 7.7, 1.2 Hz, 1H), 7.96 – 7.88 (m, 2H), 7.79 – 7.74 (m, 1H), 7.70 (d, J = 7.9 Hz, 1H), 7.54 – 7.50 m, 1H), 7.48 – 7.42 (m, 2H), 7.27 – 7.24 (m, 1H), 7.20 (d, J = 7.7 Hz, 1H), 7.07 (d, J = 7.6 Hz, 1H), 4.72 – 4.64 (m, 2H), 4.54 – 4.47 (m, 1H). ^{13}C NMR (101 MHz, $CDCl_3$) δ 165.1, 156.9, 149.8, 142.6, 140.3, 138.8, 137.0, 134.1, 130.6, 129.6, 129.3, 128.1, 127.6, 127.6, 126.5, 125.1, 122.6, 120.8, 72.2, 43.8. m/z (ESI$^+$) HRM: Calculated for $[C_{20}H_{16}NO_2]^+$: 302.1176. Found $[M+H]^+$: 302.1180.

1-Phenyl-1-(3-(pyridin-2-yl)phenyl)propan-2-ol 3al *(two diastereoisomers were obtained and separated by column chromatography, and the relative stereochemistry was not determined)*

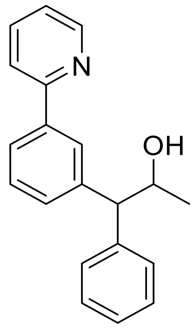

Obtained by using **General procedure A**: 2-Phenylpyridine **1a** (32.0 uL, 0.20 mmol) and 2-methyl-3-phenyloxirane ***rac*-trans-2aj** or ***rac*-cis-2aj** (53.5 mg, 0.40 mmol) were employed (*trans*- and *cis*-isomer delivered the identical result). The crude mixture was purified by column chromatography (20-40% EtOAc/Hex) to yield the title compound **3al-major diastereoisomer** (26.7 mg, 46%) as a colorless oil and **3al-minor diastereoisomer** (16.8 mg, 29%) as a colorless oil;

Major diastereoisomer: ^{1}H NMR (400 MHz, $CDCl_3$) δ 8.63 (d, J = 4.4 Hz, 1H), 8.02 (s, 1H), 7.76 (d, J = 7.4 Hz, 1H), 7.73 – 7.67 (m, 1H), 7.65 (d, J = 7.8 Hz, 1H), 7.44 – 7.37 (m, 2H), 7.31 – 7.30 (m, 2H), 7.27 – 7.23 (m, 2H), 7.20 – 7.14 (m, 2H), 4.67 – 4.60 (m, 1H), 3.91 (d, J = 8.8 Hz, 1H), 2.71 (s, br, 1H), 1.19 (d, J = 6.1 Hz, 3H). ^{13}C NMR (101 MHz, $CDCl_3$) δ 157.4, 149.5, 142.6, 142.4, 139.7, 136.9, 129.5, 129.2, 128.7, 128.3, 127.4, 126.6, 125.5, 122.2, 120.9, 69.9, 60.7, 21.7. m/z (ESI$^+$) HRMS: Calculated for $[C_{20}H_{20}NO]^+$: 290.1539. Found $[M+H]^+$: 290.1539.

Minor diastereoisomer: ^{1}H NMR (400 MHz, $CDCl_3$) δ 8.69 (dd, J = 4.8, 0.6 Hz, 1H), 7.96 (s, 1H), 7.80 – 7.77 (m, 1H), 7.76 – 7.70 (m, 1H), 7.68 (d, J = 7.9 Hz, 1H), 7.46 – 7.44 (m, 2H), 7.42 – 7.38 (m, 1H), 7.37 – 7.31 (m, 3H), 7.24 – 7.20 (m, 2H), 4.67 – 4.60 (m, 1H), 3.93 (d, J = 8.7 Hz, 1H), 1.24 (d, J = 6.1 Hz, 3H). ^{13}C NMR (101 MHz, $CDCl_3$) δ 157.4, 149.7, 143.2, 141.4, 139.7, 136.9, 129.1, 128.9, 128.8, 128.7, 127.1, 127.0, 125.3, 122.2, 120.8, 70.1, 60.6, 21.6. m/z (ESI$^+$) HRMS: Calculated for $[C_{20}H_{20}NO]^+$: 290.1539. Found $[M+H]^+$: 290.1530.

3-Methyl-1-phenyl-1-(3-(pyridin-2-yl)phenyl)butan-2-ol 3am *(two diastereoisomers were obtained and separated by column chromatography, and the relative stereochemistry was not determined)*

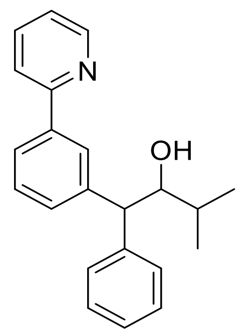

Obtained by using **General procedure A**: 2-Phenylpyridine **1a** (32.0 uL, 0.20 mmol) and 2-isopropyl-3-phenyloxirane ***rac*-trans-2ak** or ***rac*-cis-2ak** (65.0 mg, 0.40 mmol) were employed (*trans*- and *cis*-isomer delivered the identical result). The crude mixture was purified by column chromatography (40% EtOAc/Hex) to yield the title compound 3-methyl-1-phenyl-1-(3-(pyridin-2-yl)phenyl)butan-2-ol **3am-major diastereoisomer** (25.8 mg, 41%) as a colorless oil and **3am-minor diastereoisomer** (17.2 mg, 27%) as a colorless oil.

Major diastereoisomer: ^{1}H NMR (400 MHz, $CDCl_3$) δ 8.69 (d, *J* = 4.3 Hz, 1H), 8.06 (s, 1H), 7.78 (d, *J* = 7.7 Hz, 1H), 7.77 – 7.71 (m, 1H), 7.69 (d, *J* = 7.8 Hz, 1H), 7.51 (d, *J* = 7.7 Hz, 1H), 7.44 – 7.40 (m, 1H), 7.36 – 7.34 (m, 2H), 7.30 – 7.26 (m, 2H), 7.25 – 7.21 (m, 1H), 7.20 – 7.16 (m, 1H), 4.30 – 4.26 (m, 1H), 4.15 (d, *J* = 9.0 Hz, 1H), 2.57 (s, br, 1H), 1.74 – 1.67 (m, 1H), 1.02 (d, *J* = 6.9 Hz, 3H), 0.96 (d, *J* = 6.7 Hz, 3H). ^{13}C NMR (101 MHz, $CDCl_3$) δ 157.5, 149.6, 142.6, 142.5, 139.7, 137.0, 129.6, 129.2, 128.8, 128.3, 127.6, 126.6, 125.5, 122.3, 121.0, 78.1, 56.2, 29.8, 20.7, 15.2. m/z (ESI$^+$) HRMS: Calculated for $[C_{22}H_{24}NO]^+$: 318.1852. Found $[M+H]^+$: 318.1848.

Minor diastereoisomer: ^{1}H NMR (400 MHz, $CDCl_3$) δ 8.69 (d, *J* = 4.2 Hz, 1H), 7.96 (s, 1H), 7.80 (d, *J* = 7.0 Hz, 1H), 7.77 – 7.71 (m, 1H), 7.68 (d, *J* = 7.9 Hz, 1H), 7.49 – 7.47 (m, 2H), 7.42 – 7.35 (m, 2H), 7.35 – 7.31 (m, 2H), 7.25 – 7.19 (m, 2H), 4.25 – 4.21 (m, 1H), 4.16 (d, *J* = 8.4 Hz, 1H), 1.80 – 1.66 (m, 1H), 1.02 (d, *J* = 6.9 Hz, 3H), 0.97 (d, *J* = 6.7 Hz, 3H). ^{13}C NMR (101 MHz, $CDCl_3$) δ 157.4, 149.8, 143.2, 141.5, 139.8, 136.8, 129.1, 128.9, 127.1, 126.9, 125.2, 122.2, 120.8, 78.4, 55.9, 30.0, 20.6, 15.6. *(Two of the aromatic*

carbon signals are missing due to overlap). m/z (ESI^+) HRMS: Calculated for $[C_{22}H_{24}NO]^+$: 318.1852. Found $[M+H]^+$: 318.1852.

1,2-Diphenyl-2-(3-(pyridin-2-yl)phenyl)ethan-1-ol 3an *(two diastereoisomers were obtained and separated by column chromatography, and the relative stereochemistry was not determined)*

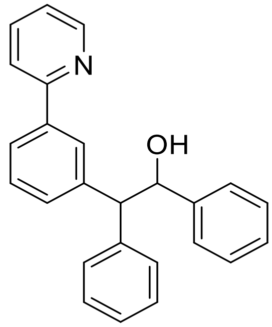

Obtained by using **General procedure A**: 2-Phenylpyridine **1a** (32.0 uL, 0.20 mmol) and 2,3-diphenyloxirane ***rac*-trans-2al** or ***rac*-cis-2al** (78.4 mg, 0.40 mmol) were employed (*trans*- and *cis*-isomer delivered the identical result). The crude mixture was purified by column chromatography (40% EtOAc/Hex) to yield the title compound 1,2-diphenyl-2-(3-(pyridin-2-yl)phenyl)ethan-1-ol **3an-major diastereoisomer** (21.4 mg, 30%) as a colorless oil and **3an-minor diastereoisomer** (12.6 mg, 18%) as a colorless oil;

Major diastereoisomer: ^1H NMR (400 MHz, $CDCl_3$) δ 8.66 (d, *J* = 4.6 Hz, 1H), 8.09 (s, 1H), 7.83 (d, *J* = 7.5 Hz, 1H), 7.75 – 7.69 (m, 1H), 7.67 (d, *J* = 7.8 Hz, 1H), 7.49 (d, *J* = 7.7 Hz, 1H), 7.46 – 7.42 (m, 1H), 7.22 – 7.19 (m, 6H), 7.14 – 7.13 (m, 4H), 7.10 – 7.06 (m, 1H), 5.47 (d, *J* = 9.1 Hz, 1H), 4.37 (d, *J* = 9.1 Hz, 1H), 3.00 (s, br, 1H). ^{13}C NMR (101 MHz, $CDCl_3$) δ 157.4, 149.5, 142.5, 141.9, 141.5, 139.6, 136.9, 129.7, 129.1, 128.7, 128.2, 128.0, 127.6, 127.5, 127.0, 126.4, 125.5, 122.2, 120.9, 76.7, 60.4. m/z (ESI^+) HRMS: Calculated for $[C_{25}H_{22}NO]^+$: 352.1696. Found $[M+H]^+$: 352.1696.

Minor diastereoisomer: ^1H NMR (400 MHz, $CDCl_3$) δ 8.63 – 8.52 (m, 1H), 7.73 (s, 1H), 7.70 – 7.63 (m, 2H), 7.49 (d, *J* = 8.0 Hz, 1H), 7.42 – 7.40 (d, *J* = 7.3 Hz, 2H), 7.33 – 7.29 (m, 2H), 7.25 – 7.18 (m, 6H), 7.17 – 7.12 (m, 3H), 5.45 (d, *J* = 8.4 Hz, 1H), 4.34 (d, *J* = 8.4 Hz, 1H), 2.41 (s, br, 1H). ^{13}C NMR (101 MHz, $CDCl_3$) δ 157.5, 149.7, 142.4, 142.2, 140.7, 139.4, 136.7, 131.0, 129.3, 129.2, 128.9, 128.2, 127.7, 127.5, 127.2, 127.1, 125.2,

122.1, 120.7, 60.3, 29.8. m/z (ESI^+) HRMS: Calculated for $[C_{25}H_{22}NO]^+$: 352.1696. Found $[M+H]^+$: 352.1697.

(1*S, 2*S**)-1-(3-(Pyridin-2-yl)phenyl)-2,3-dihydro-1*H*-inden-2-ol 3am-major diastereomer** ***(racemic)*** and **(1*R**,2*S**)-1-(3-(Pyridin-2-yl)phenyl)-2,3-dihydro-1*H*-inden-2-ol 3ao** ***(racemic)***

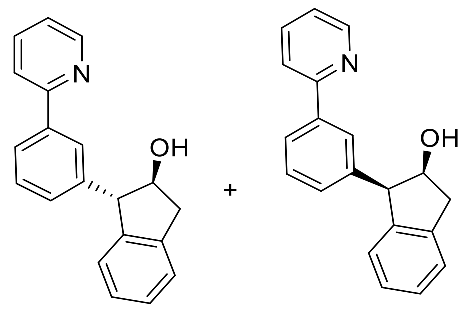

major minor

Obtained by using **General procedure A**: 2-Phenylpyridine **1a** (32.0 uL, 0.20 mmol) and 1a,6a-dihydro-6H-indeno[1,2-b]oxirene ***rac*-2am** (52.8 mg, 0.40 mmol) were employed. The crude mixture was purified by column chromatography (40% EtOAc/Hex) to yield the title compound **3ao-major diastereoisomer** ***(trans, racemic)*** and **3ao-minor diastereoisomer** ***(cis, racemic)*** (49.0 mg, 86% in total, 25:1 ratio) as a colorless oil. ***3ao-Major diastereoisomer*** *and* ***3ao-minor diastereoisomer*** *could not be separated by column chromatography*

Major diastereoisomer : ^{1}H NMR (400 MHz, $CDCl_3$) δ 8.56 (d, *J* = 4.8 Hz, 1H), 7.83 (s, 1H), 7.79 (d, *J* = 7.8 Hz, 1H), 7.73 – 7.67 (m, 1H), 7.64 (d, *J* = 7.9 Hz, 1H), 7.41 – 7.37 (m, 1H), 7.24 (d, *J* = 5.5 Hz, 1H), 7.21 – 7.13 (m, 4H), 6.95 (d, *J* = 7.4 Hz, 1H), 4.51 (ddd, *J* = 7.3, 7.3, 7.3 Hz, 1H), 4.25 (d, *J* = 7.3 Hz, 1H), 3.60 (s, br, 1H), 3.27 (dd, *J* = 15.5, 7.3 Hz, 1H), 2.97 (dd, *J* = 15.5, 7.4 Hz, 1H). ^{13}C NMR (101 MHz, $CDCl_3$) δ 157.5, 149.5, 143.6, 142.7, 140.7, 139.7, 136.9, 129.1, 129.0, 127.6, 127.2, 127.0, 125.7, 125.2, 124.7, 122.2, 121.0, 82.2, 60.3, 40.1. m/z (ESI^+) HRMS: Calculated for $[C_{20}H_{18}NO]^+$: 288.1383. Found $[M+H]^+$: 288.1384.

Minor diastereoisomer: *Characteristic signals only:* 4.70 (ddd, *J* = 5.5, 5.5, 2.7 Hz, 1H), 4.56 (d, *J* = 5.5 Hz, 1H).

Due to the deficiency of useful information in 2D NMR (NOE), the relative stereochemistry of the products was tentatively assigned by analogy to the characterization of trans and cis-2-methoxy-1-phenyl-2,3-dihydro-1H-indene that reported in literature[21]. The trans diastereomer should have three similar coupling constants for CH_1 that directly tethered to the hydroxy group (4.51 (ddd, J = 7.3, 7.3, 7.3 Hz, 1H)), whereas the cis diastereomer should have two similar and one different coupling constants (4.70 (ddd, J =5.5, 5.5, 2.7 Hz, 1H)).

7-Chloro-5-(3-(2-hydroxy-1-phenylethyl)phenyl)-1-methyl-1,3-dihydro-2*H*-benzo[e][1,4]diazepin-2-one 3ap

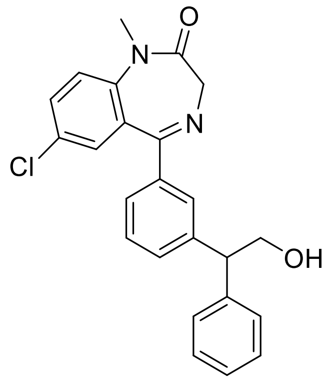

Obtained by using **General procedure A**: Diazepam **1r** (56.9 mg, 0.20 mmol) and 2-phenyloxirane **2a** (46.0 uL, 0.40 mmol) were employed. The crude mixture was purified by column chromatography (0-100% Acetone/Hex) to yield the title compound **3ap** (66.4 mg, 82%) as a brown solid; mp 93 – 96 °C (EtOAc). *Due to conformation of the 7 membered ring, **3ap** was obtained as a mixture of two diastereoisomers (1:1 ratio), which could not be separated by column chromatography.*

The mixture of two diastereoisomers: ^{1}H NMR (400 MHz, $CDCl_3$) δ 7.56 (app. s, br, 1H), 7.58 – 7.57 (m, 1H), 7.51 – 7.48 (m, 2H), 7.42 – 7.40 (m, 1H), 7.38 – 7.28 (m, 10H), 7.26 – 7.19 (m, 9H), 4.78 (app. dd, J = 10.8, 6.3 Hz, 2H), 4.25 – 4.23 (m, 2H), 4.19 – 4.11 (m, 4H), 3.73 (d, J = 10.8 Hz, 2H), 3.36 (s, 6H), 2.39 (s, br, 1H), 2.16 (s, br, 1H). ^{13}C NMR (101 MHz, $CDCl_3$) δ 170.0, 169.9, 169.2, 168.9, 142.7, 142.3, 142.1, 141.3, 141.2, 138.4, 138.3, 131.7, 131.6, 131.2, 130.9, 130.1, 130.0, 130.0, 129.4, 129.4, 129.3, 129.1, 128.9, 128.8, 128.8, 128.7, 128.5, 128.4, 128.4, 128.2, 127.0, 126.9, 122.6, 66.0, 65.8, 57.0, 56.9,

53.6, 35.0. m/z (ESI$^+$) HRMS: Calculated for $[C_{24}H_{21}O_2N_2ClNa]^+$: 427.1184. Found $[M+Na]^+$: 427.1200.

Estrone derivates 3aq

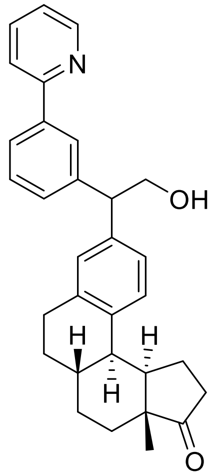

Obtained by using **General procedure A**: 2-Phenylpyridine **1a** (32.0 uL, 0.20 mmol) and Estone **2ao** (118.4 mg, 0.40 mmol) were employed. The crude mixture was purified by column chromatography (40% EtOAc/Hex) to yield **3aq** (106.0 mg, 99%) as a colorless oil. *Due to the introduction of additional stereocenters (Estrone has a fixed absolute configuration), **3ao** was obtained as a mixture of two diastereoisomers (1:1 ratio), which could not be separated by column chromatography, and the signals of the two diastereoisomers in ^{1}H NMR and ^{13}C NMR largely overlapped.*

The mixture of two diastereoisomers: ^{1}H NMR (400 MHz, $CDCl_3$) δ 8.62 (d, J = 4.7 Hz, 1H), 7.93 (d, J = 1.7 Hz, 1H), 7.77 (d, J = 7.6 Hz, 1H), 7.78 – 7.70 (m, 1H), 7.67 (d, J = 7.6 Hz, 1H), 7.41 – 7.38 (m, 1H), 7.31 (d, J = 7.7 Hz, 1H), 7.23 – 7.18 (m, 2H), 7.07 (d, J = 8.0 Hz, 1H), 7.00 (s, 1H), 4.27 – 4.11 (m, 3H), 2.85 (dd, J = 8.7, 3.7 Hz, 2H), 2.48 (dd, J = 18.9, 8.6 Hz, 1H), 2.37 (d, J = 9.4 Hz, 1H), 2.24 (dd, J = 8.4, 8.4 Hz, 1H), 2.13 (dd, J = 18.5, 9.4 Hz, 1H), 2.04 (dd, J = 19.5, 12.0 Hz, 2H), 1.93 (d, J = 9.5 Hz, 1H), 1.66 – 1.54 (m, 2H), 1.51 (d, J = 5.2 Hz, 1H), 1.49 – 1.43 (m, 2H), 1.43 – 1.35 (m, 1H), 0.87 (d, J = 0.8 Hz, 3H). ^{13}C NMR (101 MHz, $CDCl_3$) δ 220.9, 157.4, 149.5, 142.4, 139.5, 138.9, 138.1, 136.8, 136.6, 132.0, 129.1, 129.0, 128.6, 126.9, 125.6, 125.1, 122.2, 120.9, 65.9, 53.4, 50.4, 47.9, 44.2, 38.0, 35.8, 31.5, 29.4, 26.4, 25.6, 21.5, 13.8. m/z (ESI$^+$) HRMS: Calculated for $[C_{31}H_{34}NO_2]^+$: 452.2584. Found $[M+H]^+$: 452.2578.

The ratio between two diastereoisomers (1:1) was determined by using a chiral HPLC (condition: OJ-H column, hexane/isopropanol = 80/20, 1mL/min, 35℃).

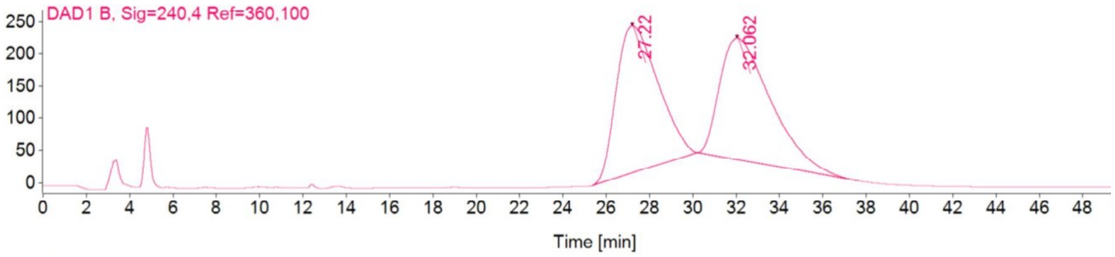

Signal: DAD1 B, Sig=240,4 Ref=360,100

RT [min]	Height	Symm.	Width (50%)	Area	Area%
27.220	228.66173	0.61	2.2800	30529.04102	49.52
32.062	189.56055	0.49	2.4600	31121.64258	50.48

Supplementary Figure 3. Chiral HPLC analysis of Estrone derivates **3aq**

Adapalene derivates 3ar

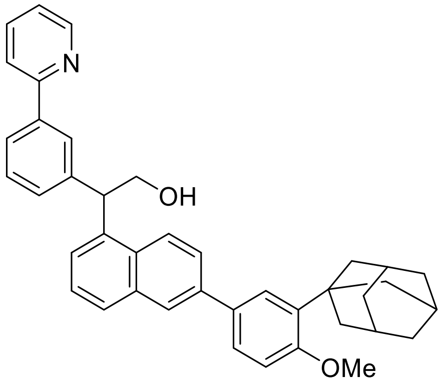

Obtained by using **General procedure C**: 2-Phenylpyridine **1a** (32.0 uL, 0.20 mmol) and 2-(6-(3-(Adamantan-1-yl)-4-methoxyphenyl)naphthalen-1-yl)oxirane **2ap** (328.0 mg, 0.80 mmol) were employed. Dioxane (0.3 ml) is added. The crude mixture was purified by column chromatography (40% EtOAc/Hex) to yield **3ar** (69.0 mg, 61%) as a colorless oil; ^{1}H NMR (400 MHz, $CDCl_3$) δ 8.66 (d, *J* = 4.6 Hz, 1H), 8.00 (s, 1H), 7.95 (s, 1H), 7.85 – 7.82 (m, 3H), 7.78 (s, 1H), 7.74 – 7.67 (m 3H), 7.59 (d, *J* = 2.1 Hz, 1H), 7.52 (dd, *J* = 8.4, 2.1 Hz, 1H), 7.47 – 7.34 (m, 3H), 7.24 – 7.18 (m, 1H), 6.98 (d, *J* = 8.5 Hz, 1H), 4.48 (app. t, *J* = 7.1 Hz, 1H), 4.35 – 4.33 (m, 2H), 3.90 (s, 3H), 2.19 (s, 6H), 2.11 (s, 3H), 1.81 (s, 6H).

^{13}C NMR (101 MHz, $CDCl_3$) δ 158.6, 157.4, 149.7, 142.2, 139.9, 138.9, 138.6, 136.9, 133.2, 132.8, 132.4, 129.2, 129.2, 128.6, 128.2, 127.3, 127.2, 126.5, 126.0, 125.9, 125.6, 125.6, 124.8, 122.3, 120.9, 112.1, 66.0, 55.2, 53.8, 40.7, 37.3, 37.2, 29.2. *(One of the aromatic carbon signals is missing due to overlap)*. m/z (ESI^+) HRMS: Calculated for $[C_{40}H_{40}NO_2]^+$: 566.3054. Found $[M+H]^+$: 566.3053.

Telmisartan derivates 3as

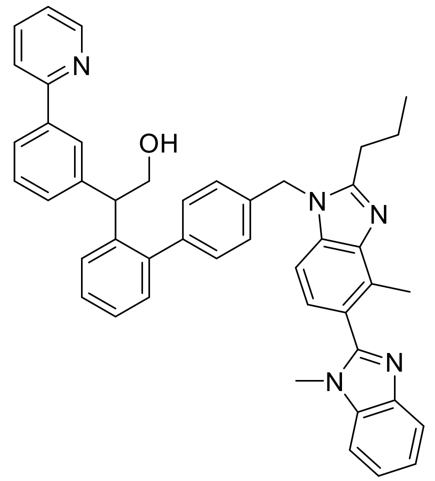

Obtained by using **General procedure A**: 2-Phenylpyridine **1a** (32.0 uL, 0.20 mmol) and from telmisartan **2aq** (200.0 mg, 0.40 mmol) were employed. The crude mixture was purified by column chromatography (40% EtOAc/Hex) to yield **3as** (69.0 mg, 52%) as a colorless oil; ^1H NMR (400 MHz, $CDCl_3$) δ 8.58 (d, J = 4.8 Hz, 1H), 7.73 – 7.70 (m, 3H), 7.68 – 7.61 (m, 1H), 7.53 (d, J = 8.0 Hz, 1H), 7.44 (s, 1H), 7.40 – 7.38 (m, 2H), 7.34 – 7.27 (m, 2H), 7.26 – 7.21 (m, 4H), 7.16 – 7.14 (m, 4H), 7.04 – 7.02 (m, 3H), 5.38 (s, 2H), 4.36 (app. t, J = 7.3 Hz, 1H), 4.12 – 3.98 (m, 2H), 3.72 (s, 3H), 2.97 – 2.86 (m, 2H), 2.75 (s, 3H), 1.92 – 1.79 (m, 2H), 1.04 (t, J = 7.3 Hz, 3H). ^{13}C NMR (101 MHz, $CDCl_3$) δ 157.4, 156.5, 154.7, 149.6, 143.2, 142.8, 142.4, 142.2, 141.3, 139.5, 138.9, 136.8, 136.6, 135.0, 134.6, 130.4, 130.2, 129.4, 129.2, 128.9, 128.0, 127.7, 126.7, 126.5, 126.0, 125.2, 123.9, 122.5, 122.4, 122.1, 120.7, 119.5, 109.6, 109.1, 65.9, 49.3, 47.1, 31.8, 29.8, 22.0, 17.0, 14.1. m/z (ESI^+) HRMS: Calculated for $[C_{45}H_{42}N_5O]^+$: 668.3384. Found $[M+H]^+$: 668.3378.

3-Phenyl-3-(3-(pyridin-2-yl)phenyl)propan-1-ol 5a

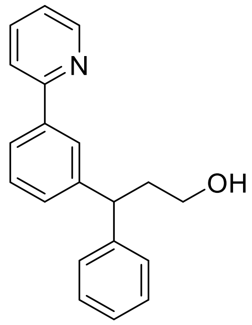

Obtained by using **General procedure B**: 2-Phenylpyridine **1a** (32.0 uL, 0.20 mmol) and 2-phenyloxetane **4a** (107.2 mg, 0.40 mmol) were employed. The crude mixture was purified by column chromatography (40% EtOAc/Hex) to yield the title compound **5a** (40.0 mg, 62%) as a colorless oil; ^{1}H NMR (400 MHz, $CDCl_3$) δ 8.67 (d, *J* = 4.7 Hz, 1H), 7.92 (s, 1H), 7.77 (d, *J* = 7.7 Hz, 1H), 7.75 – 7.70 (m, 1H), 7.68 (d, *J* = 7.9 Hz, 1H), 7.40 – 7.37 (m, 1H), 7.34 – 7.26 (m, 5H), 7.24 – 7.15 (m, 2H), 4.25 (t, *J* = 7.9 Hz, 1H), 3.63 (t, *J* = 6.4 Hz, 2H), 2.40 – 2.35 (m, 2H), 1.93 (s, br, 1H). ^{13}C NMR (101 MHz, $CDCl_3$) δ 157.6, 149.7, 145.1, 144.5, 139.6, 136.8, 129.0, 128.6, 128.6, 128.0, 126.8, 126.4, 125.1, 122.2, 120.9, 61.0, 47.5, 38.3. m/z (ESI^+) HRMS: Calculated for $[C_{20}H_{20}NO]^+$: 290.1539. Found $[M+H]^+$: 290.1537.

Methyl 2-(3-hydroxy-1-phenylpropyl)-4-(pyridin-2-yl)benzoate 5b

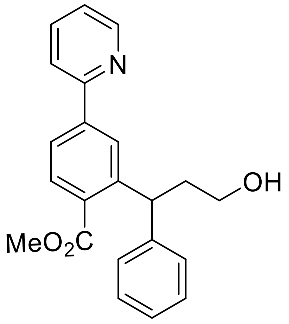

Obtained by using **General procedure B**: methyl 4-(pyridin-2-yl)benzoate **1n** (42.6 mg, 0.20 mmol) and 2-phenyloxetane **4a** (107.2.0 mg, 0.80 mmol) were employed. The crude mixture was purified by column chromatography (40% EtOAc/Hex) to yield the title compound **5b** (22.0 mg, 38%) as a colorless oil; ^{1}H NMR (400 MHz, $CDCl_3$) δ 8.68 (d, *J* = 4.2 Hz, 1H), 7.94 (s, 1H), 7.91 – 7.83 (m, 2H), 7.77 – 7.69 (m, 1H), 7.63 (d, *J* = 8.0 Hz, 1H), 7.31 – 7.28 (m, 4H), 7.26 – 7.22 (m, 1H), 7.20 – 7.13 (m, 1H), 5.24 (dd, *J* = 9.5, 6.1

Hz, 1H), 3.95 (s, 3H), 3.73 – 3.62 (m, 1H), 3.59 – 3.47 (m, 1H), 2.87 (s, br, 1H), 2.55 – 2.46 (m, 1H), 2.41 – 2.33 (m, 1H). ^{13}C NMR (101 MHz, $CDCl_3$) δ 169.3, 156.2, 149.9, 146.2, 144.4, 142.8, 136.9, 130.8, 130.7, 128.5, 128.2, 127.6, 126.3, 124.6, 122.9, 121.1, 60.5, 52.7, 41.2, 38.8. m/z (ESI$^+$) HRMS: Calculated for $[C_{22}H_{21}NNaO_3]^+$: 370.1414. Found $[M+Na]^+$: 370.1421.

3-([1,1'-Biphenyl]-4-yl)-3-(3-(pyridin-2-yl)phenyl)propan-1-ol 5c

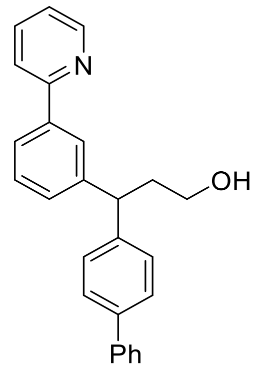

Obtained by using **General procedure B**: 2-Phenylpyridine **1a** (32.0 uL, 0.20 mmol) and 2-([1,1'-biphenyl]-4-yl)oxetane **4c** (168.0 mg, 0.80 mmol) were employed. The crude mixture was purified by column chromatography (40% EtOAc/Hex) to yield the title compound **5c** (33.0 mg, 46%) as a colorless oil; ^{1}H NMR (400 MHz, $CDCl_3$) δ 8.67 (d, J = 4.2 Hz, 1H), 7.97 (s, 1H), 7.79 – 7.75 (m, 1H), 7.75 – 7.70 (m, 1H), 7.70 – 7.67 (m, 1H), 7.57 – 7.55 (m, 2H), 7.53 – 7.51 (m, 2H), 7.44 – 7.39 (m, 3H), 7.39 – 7.32 (m, 4H), 7.23 – 7.20 (m, 1H), 4.31 (t, J = 7.9 Hz, 1H), 3.67 – 3.61 (m, 2H), 2.95 (s, br, 1H), 2.41 (dd, J = 14.1, 6.7 Hz, 2H). ^{13}C NMR (101 MHz, $CDCl_3$) δ 157.6, 149.5, 145.1, 143.6, 140.9, 139.5, 139.2, 137.0, 129.1, 128.8, 128.6, 128.4, 127.3, 127.1, 127.0, 126.9, 125.1, 122.2, 121.0, 60.7, 47.1, 38.3. m/z (ESI$^+$) HRMS: Calculated for $[C_{26}H_{24}NO]^+$: 366.1852. Found $[M+H]^+$: 366.1861.

3-(4-Fluorophenyl)-3-(3-(pyridin-2-yl)phenyl)propan-1-ol 5d

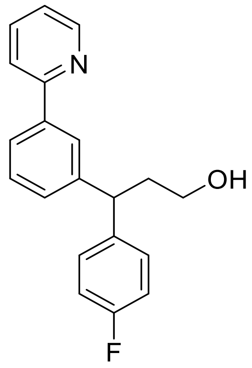

Obtained by using **General procedure B**: 2-Phenylpyridine **1a** (32.0 uL, 0.20 mmol) and 2-(4-fluorophenyl)oxetane **4d** (121.6 mg, 0.80 mmol) were employed. The crude mixture was purified by column chromatography (40% EtOAc/Hex) to yield the title compound **5d** (25.0 mg, 41%) as a colorless oil; ^{1}H NMR (400 MHz, $CDCl_3$) δ 8.64 (dd, *J* = 4.9, 0.8 Hz, 1H), 7.88 (s, 1H), 7.77 – 7.69 (m, 2H), 7.66 (d, *J* = 8.0 Hz, 1H), 7.39 – 7.35 (m, 1H), 7.26 – 7.19 (m, 4H), 6.97 – 6.92 (m, 2H), 4.23 (t, *J* = 7.9 Hz, 1H), 3.60 – 3.56 (m, 2H), 2.87 (s, br, 1H), 2.34 – 2.29 (m, 2H). ^{13}C NMR (101 MHz, $CDCl_3$) δ 161.4 (d, *J* = 244.3 Hz), 157.5, 149.5, 145.0, 140.2 (d, *J* = 3.2 Hz), 139.5, 137.0, 129.3 (d, *J* = 7.8 Hz), 129.1, 128.5, 126.8, 125.1, 122.3, 121.1, 115.3 (d, *J* = 21.1 Hz), 60.5, 46.5, 38.3. ^{19}F NMR (376 MHz, $CDCl_3$) δ -116.9 (s). m/z (ESI$^+$) HRMS: Calculated for $[C_{20}H_{19}FNO]^+$: 308.1445. Found $[M+H]^+$: 308.1449.

3-(4-Chlorophenyl)-3-(3-(pyridin-2-yl)phenyl)propan-1-ol 5e

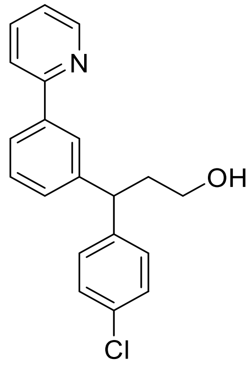

Obtained by using **General procedure B**: 2-Phenylpyridine **1a** (32.0 uL, 0.20 mmol) and 2-(4-chlorophenyl)oxetane **4e** (134.4 mg, 0.80 mmol) were employed. The crude mixture was purified by column chromatography (40% EtOAc/Hex) to yield the title compound **5e** (28.0 mg, 43%) as a colorless oil; ^{1}H NMR (400 MHz, $CDCl_3$) δ 8.66 (d, *J* = 4.5 Hz, 1H),

7.89 (s, 1H), 7.79 – 7.71 (m, 2H), 7.67 (d, *J* = 7.9 Hz, 1H), 7.41 – 7.37 (m, 1H), 7.27 – 7.20 (m, 6H), 4.23 (t, *J* = 7.9 Hz, 1H), 3.61 (t, *J* = 6.4 Hz, 2H), 2.36 – 2.30 (m, 2H), 1.95 (s, br, 1H). ^{13}C NMR (101 MHz, $CDCl_3$) δ 157.5, 149.7, 144.6, 143.1, 139.8, 136.9, 132.1, 129.4, 129.2, 128.7, 128.5, 126.8, 125.2, 122.3, 121.0, 60.7, 46.7, 38.2. m/z (ESI$^+$) HRMS: Calculated for $[C_{20}H_{18}ClNNaO]^+$: 346.0969. Found $[M+Na]^+$: 346.0964.

3-(4-Bromophenyl)-3-(3-(pyridin-2-yl)phenyl)propan-1-ol 5f

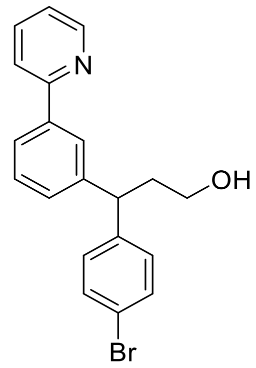

Obtained by using **General procedure B**: 2-Phenylpyridine **1a** (32.0 uL, 0.20 mmol) and 2-(4-bromophenyl)oxetane **4f** (170.4 mg, 0.80 mmol) were employed. The crude mixture was purified by column chromatography (40% EtOAc/Hex) to yield the title compound **5f** (32.0 mg, 44%) as a colorless oil; ^1H NMR (400 MHz, $CDCl_3$) δ 8.66 (d, *J* = 4.3 Hz, 1H), 7.89 (s, 1H), 7.79 – 7.70 (m, 2H), 7.67 (d, *J* = 7.9 Hz, 1H), 7.40 – 7.37 (m, 3H), 7.26 – 7.20 (m, 2H), 7.18 – 7.15 (m, 2H), 4.22 (t, *J* = 7.9 Hz, 1H), 3.61 (t, *J* = 6.4 Hz, 2H), 2.39 – 2.26 (m, 2H), 1.90 (s, br, 1H). ^{13}C NMR (101 MHz, $CDCl_3$) δ 157.5, 149.7, 144.5, 143.6, 139.8, 137.0, 131.7, 129.8, 129.2, 128.5, 126.8, 125.3, 122.3, 121.0, 120.2, 60.7, 46.8, 38.1. m/z (ESI$^+$) HRMS: Calculated for $[C_{20}H_{18}BrNNaO]^+$: 390.0464. Found $[M+Na]^+$: 390.0460.

3-(3-(Pyridin-2-yl)phenyl)-3-(p-tolyl)propan-1-ol 5g

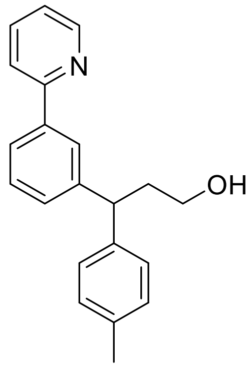

Obtained by using **General procedure B**: 2-Phenylpyridine **1a** (32.0 uL, 0.20 mmol) and 2-(*p*-tolyl)oxetane **4g** (120.0 mg, 0.80 mmol) were employed. The crude mixture was purified by column chromatography (40% EtOAc/Hex) to yield the title compound **5g** (25.0 mg, 42%) as a colorless oil; ^{1}H NMR (400 MHz, $CDCl_3$) δ 8.66 (d, J = 4.5 Hz, 1H), 7.91 (s, 1H), 7.77 – 7.70 (m, 2H), 7.67 (d, J = 7.9 Hz, 1H), 7.39 – 7.36 (m, 1H), 7.28 (d, J = 7.7 Hz, 1H), 7.22 – 7.17 (m, 3H), 7.10 – 7.08 (m, 2H), 4.20 (t, J = 7.9 Hz, 1H), 3.62 (t, J = 6.4 Hz, 2H), 2.36 – 2.30 (m, 2H), 2.29 (s, 3H), 2.12 (s, br, 1H). ^{13}C NMR (101 MHz, $CDCl_3$) δ 157.7, 149.6, 145.4, 141.5, 139.6, 136.9, 135.8, 129.3, 129.0, 128.5, 127.8, 126.7, 125.0, 122.2, 120.9, 61.0, 47.1, 38.4, 21.0. m/z (ESI^{+}) HRMS: Calculated for $[C_{21}H_{21}NNaO]^{+}$: 326.1515. Found $[M+Na]^{+}$: 326.1505.

II. Supplementary Discussions

1. Mechanistic Studies of Epoxide Involved *meta*-Alkylation Reaction

1) For Fig. 4A in the main manuscript

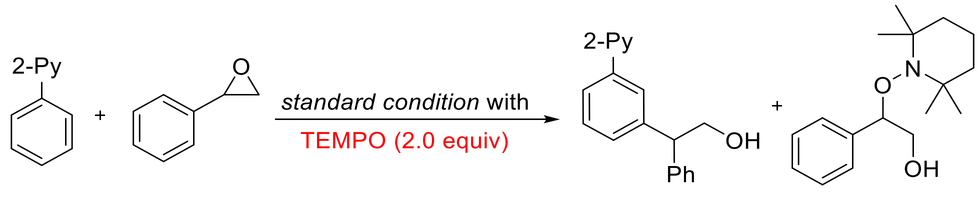

In a glove box, an oven-dried crimp-cap microwave vial equipped with a magnetic stirring bar was charged with $Ru(PPh_3)_3Cl_2$ (9.60 mg, 0.01 mmol), NaI powder (30.0 mg, 0.20 mmol) and 2-ethylbutyric acid (7.50 uL, 0.06 mmol), then substrate **1a** (32.0 uL, 0.20 mmol), epoxide **2a** (46 uL, 0.40 mmol), TEMPO (62.5 mg, 0.40 mmol) and dioxane (0.15 mL) were added. The vial was then capped and taken out of glovebox, stirred at 70 °C for 44 h. The reaction was then allowed to cool to room temperature and concentrated *in vacuo*. The NMR analysis of the crude indicated that only a trace amount of **3a** was obtained, and the TEMPO captured product was detected by MS (M=277). At the same time, the crude mixture was purified by column chromatography (10% EtOAc/Hex) to yield the TEMPO captured product (24.0 mg, 43%) as a colorless oil.

2-Phenyl-2-((2,2,6,6-tetramethylpiperidin-1-yl)oxy)ethan-1-ol. TEMPO captured product

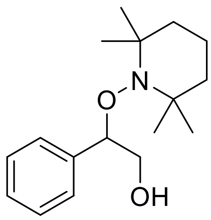

^{1}H NMR (400 MHz, $CDCl_3$) δ 7.39 – 7.32 (m, 4H), 7.32 – 7.27 (m, 1H), 5.88 (s, br, 1H), 5.31 (dd, J = 9.5, 2.7 Hz, 1H), 4.23 (dd, J = 12.2, 9.6 Hz, 1H), 3.73 (d, J = 11.6 Hz, 1H), 1.68 – 1.49 (m, 8H), 1.41 – 1.38 (m, 1H), 1.35 (s, 3H), 1.23 (s, 3H), 1.16 (s, 3H). ^{13}C NMR (101 MHz, $CDCl_3$) δ 139.0, 128.5, 128.0, 126.9, 83.7, 69.9, 61.8, 60.5, 40.5, 40.3, 34.7, 32.9, 20.9, 20.5, 17.3. *This data is consistent with literature report.* [22]

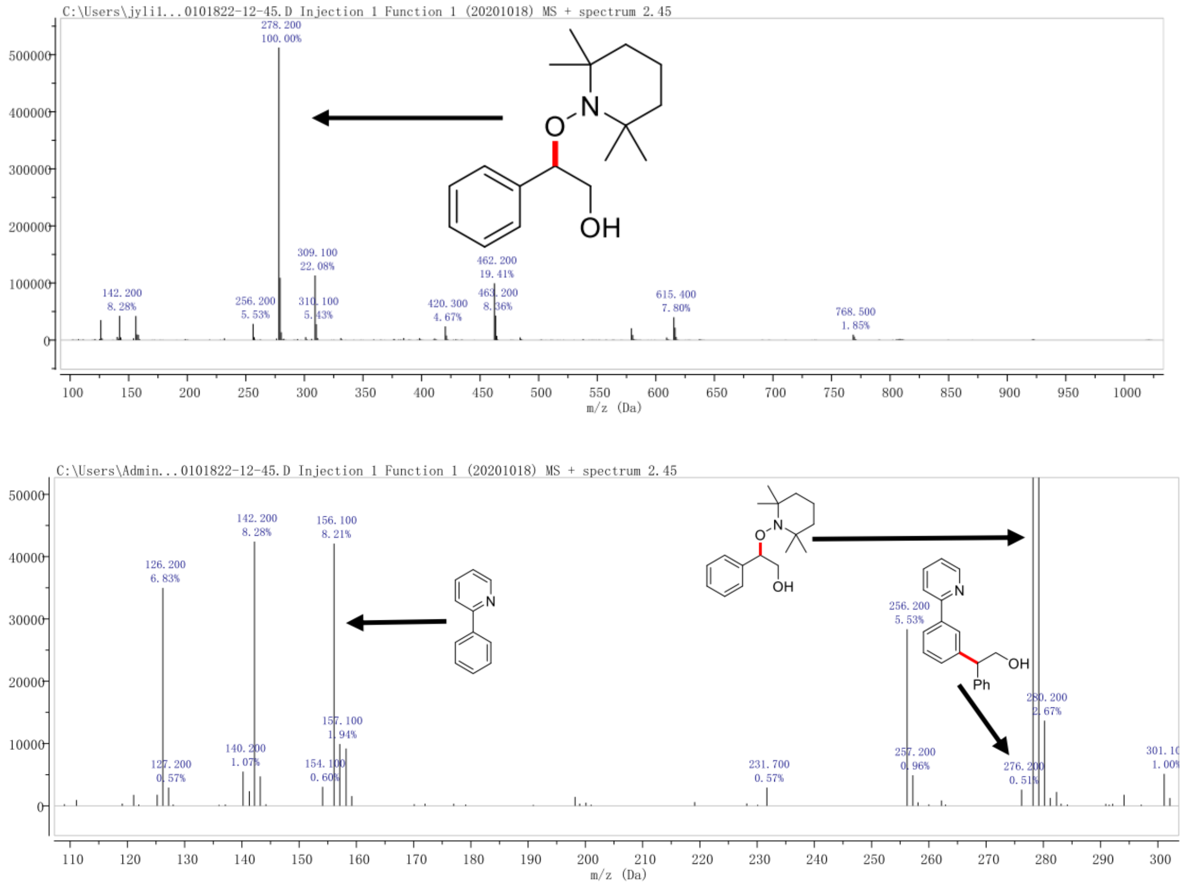

Supplementary Figure 4. MS spectra analysis of radical capture experiment

2) For Fig. 4A in the main manuscript

In a glove box, oven-dried crimp-cap microwave vials equipped with a magnetic stirring bar were charged with epoxide **2a** (46 uL, 0.40 mmol) and other materials as presented in following Supplementary Table 5, then dioxane (0.15 mL) was added to each vial. The vials were capped and taken out of glovebox, stirred at 70°C for 44 h. The reactions were then allowed to cool to room temperature and concentrated *in vacuo*. The NMR analysis of the crude from each vial were undertaken by using dibromomethane as internal standard and results were summarized in the Supplementary Table 5.

Supplementary Table 5. Mechanistic studies of epoxide ring opening process

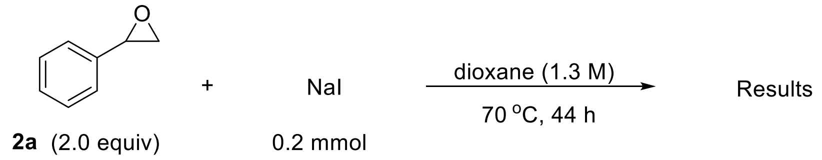

Entries	Condition variation	Results [a]
1	As above	no reaction
2	add 5% $Ru(PPh_3)_3Cl_2$	**6**: < 0.5%; **7:** 7%
3	add 30% 2-ethylbutanoic acid (**A4**)	**6**: 5%; **7**: 25%<br>**8**: 7%[b]; **9**: 21% [b]
4	add 5% $Ru(PPh_3)_3Cl_2$ with<br>30 % 2-ethylbutanoic acid (**A4**)	**6**: 5%; **7**: 24%;<br>**8**: 10%[b]; **9**: 33% [b]
5	no NaI<br>30% 2-ethylbutanoic acid (**A4**)	**8:** 0%; **9:** 0%
6	no NaI, 5% $Ru(PPh_3)_3Cl_2$<br>30% 2-ethylbutanoic acid (**A4**)	**8:** 30%[b]; **9:** 70% [b]

[a] Determined by ^{1}HNMR analysis of crude material, and yields were reported based on NaI as the limiting reagent. [b] Yields were reported based on 2-ethylbutanoic acid (**A4**) as the limiting reagent

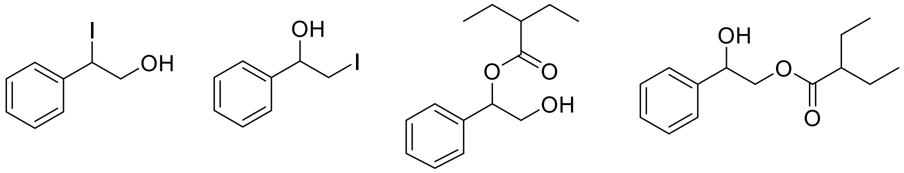

6 **7** **8** **9**

2-iodo-2-phenylethan-1-ol 6

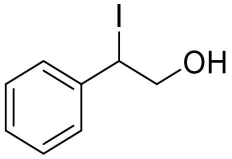

^{1}H NMR (400 MHz, $CDCl_3$) δ 7.45 – 7.40 (m, 2H), 7.36 – 7.26 (m, 3H) 5.21 (t, *J* = 7.2 Hz, 1H), 4.10 (dd, *J* = 12.2, 7.0 Hz, 1H), 3.90 (dd, *J* = 12.3, 7.0 Hz, 1H), 2.15 (s, br, 1H). ^{13}C NMR (101 MHz, $CDCl_3$) δ 140.1, 128.9, 128.5, 127.8, 68.5, 35.6. *This data is consistent with literature report.* [23]

2-iodo-1-phenylethan-1-ol 7

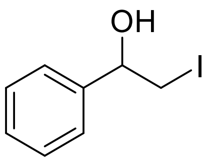

^{1}H NMR (400 MHz, $CDCl_3$) δ 7.48 – 7.29 (m, 5H), 4.83 (ddd, *J* = 8.6, 3.6, 3.6 Hz, 1H), 3.49 (dd, *J* = 10.3, 3.6 Hz, 1H), 3.40 (dd, *J* = 10.3, 8.8 Hz, 1H), 2.63 (d, *J* = 3.6 Hz, 1H). ^{13}C NMR (101 MHz, $CDCl_3$) δ 141.2, 128.7, 128.5, 125.8, 74.0, 15.4. *This data is consistent with literature report.* [24]

2-hydroxy-1-phenylethyl 2-ethylbutanoate 8

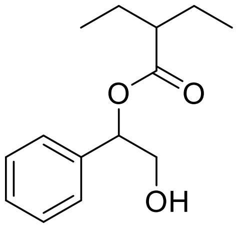

^{1}H NMR (400 MHz, $CDCl_3$) δ 7.37 – 7.29 (m, 5H), 5.88 (dd, *J* = 7.6, 4.1 Hz, 1H), 3.95 – 3.75 (m, 2H), 2.35 – 2.28 (m, 1H), 1.92 (t, *J* = 6.4 Hz, 1H), 1.71 – 1.62 (m, 2H), 1.60 – 1.50 (m, 2H), 0.90 (t, *J* = 7.4 Hz, 3H), 0.84 (t, *J* = 7.4 Hz, 3H). ^{13}C NMR (101 MHz, $CDCl_3$) δ 176.0, 137.4, 128.7, 128.5, 126.8, 76.6, 66.3, 49.2, 25.2, 25.2, 12.0, 11.9. m/z (ESI$^+$) HRMS: Calculated for $[C_{14}H_{20}NaO_3]^+$: 259.1305. Found $[M+Na]^+$: 259.1300.

2-hydroxy-2-phenylethyl 2-ethylbutanoate 9

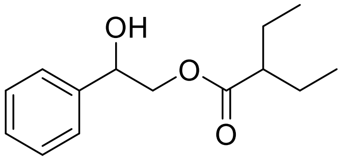

^{1}H NMR (400 MHz, $CDCl_3$) δ 7.42 – 7.34 (m, 4H), 7.34 – 7.28 (m, 1H), 5.03 – 4.91 (m, 1H), 4.32 (dd, *J* = 11.6, 3.4 Hz, 1H), 4.20 (dd, *J* = 11.6, 8.2 Hz, 1H), 2.60 (d, *J* = 3.3 Hz, 1H), 2.30 – 2.22 (m, 1H), 1.68 – 1.48 (m, 4H), 0.88 (td, *J* = 7.4, 2.1 Hz, 6H). ^{13}C NMR (101 MHz, $CDCl_3$) δ 176.6, 140.0, 128.7, 128.3, 126.3, 72.7, 69.1, 48.9, 25.2, 25.1, 11.9. m/z (ESI$^+$) HRMS: Calculated for $[C_{14}H_{20}NaO_3]^+$: 259.1305. Found $[M+Na]^+$: 259.1298.

As an example, the copy of NMR of the crude from reaction in entry 3, Supplementary Table 5 is presented as Supplementary Figure 5.

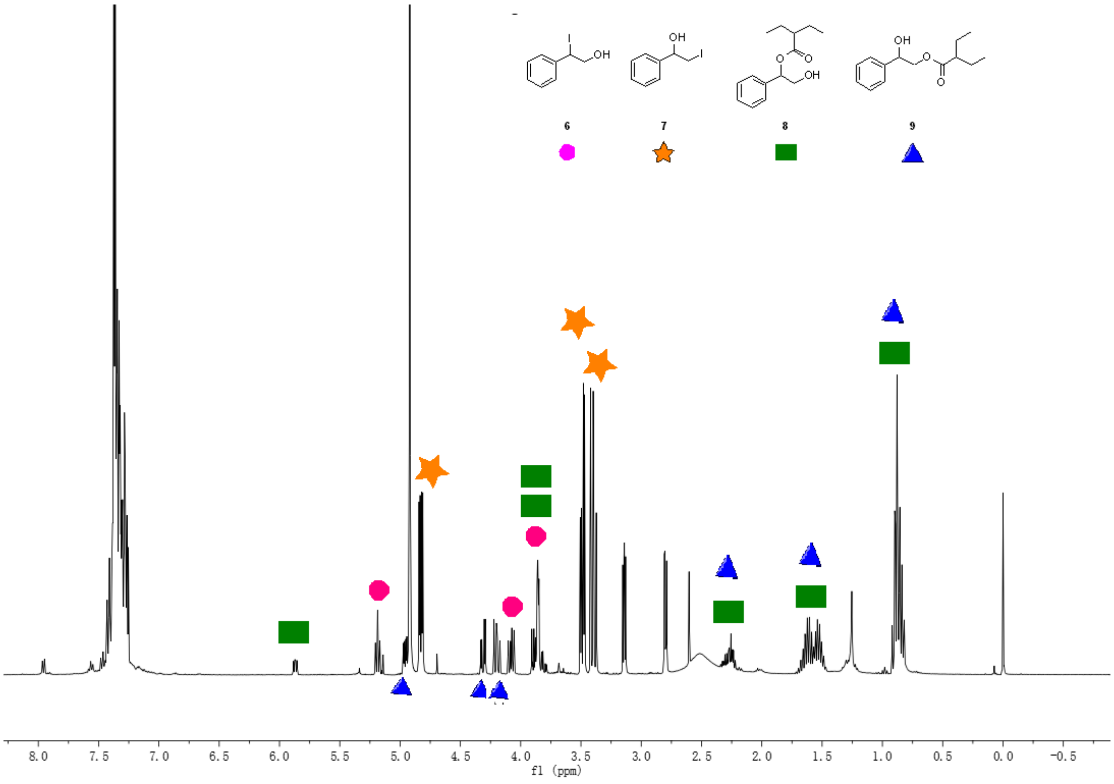

Supplementary Figure 5. Copy of NMR of crude reaction in entry 3, Supplementary Table 5

3) For Fig. 4C in the main manuscript

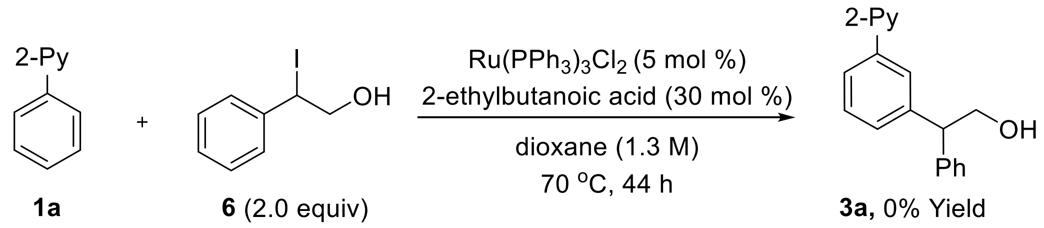

In a glove box, an oven-dried crimp-cap microwave vial equipped with a magnetic stirring bar was charged with $Ru(PPh_3)_3Cl_2$ (9.60 mg, 0.01 mmol) and 2-ethylbutyric acid (7.5 uL, 0.06 mmol), then substrate **1a** (32.0 uL, 0.20 mmol), 2-iodo-2-phenylethan-1-ol **6** (99.2 mg, 0.40 mmol) and dioxane (0.15 mL) were added. The vial was then capped and taken out of glovebox, stirred at 70°C for 44 h. The reaction was then allowed to cool to room

temperature and concentrated *in vacuo*. The NMR analysis of the crude indicated that **3a** was not formed.

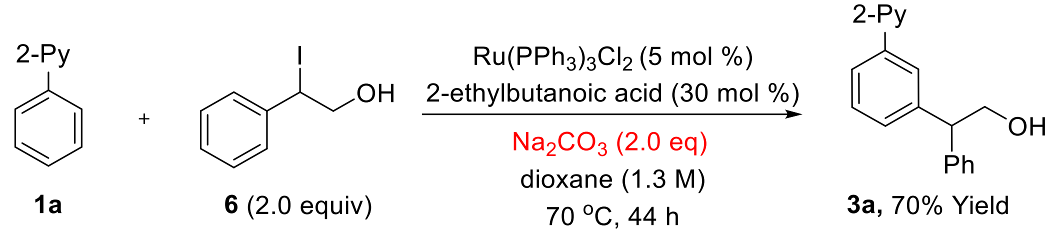

1a **6** (2.0 equiv) **3a,** 70% Yield

In a glove box, an oven-dried crimp-cap microwave vial equipped with a magnetic stirring bar was charged with $Ru(PPh_3)_3Cl_2$ (9.60 mg, 0.01 mmol) and 2-ethylbutyric acid (7.50 uL, 0.06 mmol), then substrate **1a** (32.0 uL, 0.20 mmol), 2-iodo-2-phenylethan-1-ol **6** (99.2 mg, 0.40 mmol), Na_2CO_3 (42.4 mg, 0.4 mmol) and dioxane (0.15 mL) were added. The vial was then capped and taken out of glovebox, stirred at 70°C for 44 h. The reaction was then allowed to cool to room temperature and concentrated *in vacuo*. The crude mixture was purified by column chromatography (40% EtOAc/Hex) to yield the title compound 2-phenyl-2-(3-(pyridin-2-yl)phenyl)ethan-1-ol **3a** (38.5 mg, 70%) as a colorless oil.

4) For Fig. 4D in the main manuscript

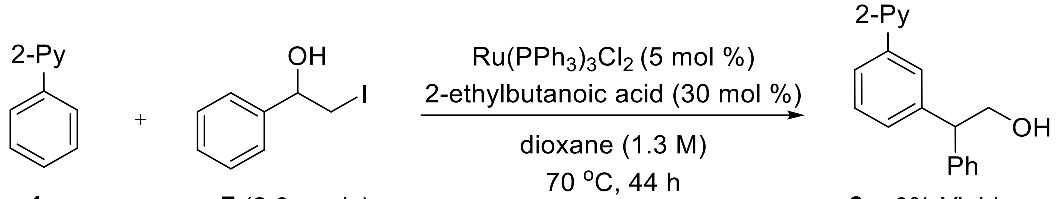

1a **7** (2.0 equiv) **3a,** 0% Yield

In a glove box, an oven-dried crimp-cap microwave vial equipped with a magnetic stirring bar was charged with $Ru(PPh_3)_3Cl_2$ (9.60 mg, 0.01 mmol) and 2-ethylbutyric acid (7.5 uL, 0.06 mmol), then substrate **1a** (32.0 uL, 0.20 mmol), 2-iodo-1-phenylethan-1-ol **7** (99.2 mg, 0.40 mmol) and dioxane (0.15 mL) were added. The vial was then capped and taken out of glovebox, stirred at 70°C for 44 h. The reaction was then allowed to cool to room temperature and concentrated *in vacuo*. The NMR analysis of the crude indicated that **3a** and other corresponding isomers were not formed.

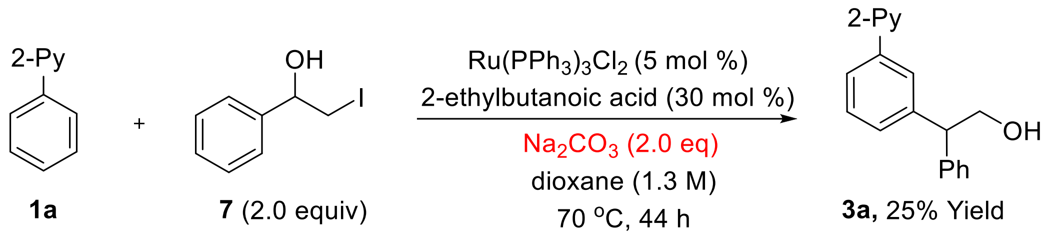

In a glove box, an oven-dried crimp-cap microwave vial equipped with a magnetic stirring bar was charged with $Ru(PPh_3)_3Cl_2$ (9.60 mg, 0.01 mmol) and 2-ethylbutyric acid (7.50 uL, 0.06 mmol), then substrate **1a** (32.0 uL, 0.20 mmol), 2-iodo-1-phenylethan-1-ol **7** (99.2 mg, 0.40 mmol), Na_2CO_3 (42.4 mg, 0.4 mmol) and dioxane (0.15 mL) were added. The vial was then capped and taken out of glovebox, stirred at 70°C for 44 h. The reaction was then allowed to cool to room temperature and concentrated *in vacuo*. The crude mixture was purified by column chromatography (40% EtOAc/Hex) to yield the title compound 2-phenyl-2-(3-(pyridin-2-yl)phenyl)ethan-1-ol **3a** (13.8 mg, 25%) as a colorless oil.

5) For Fig. 4E in the main manuscript

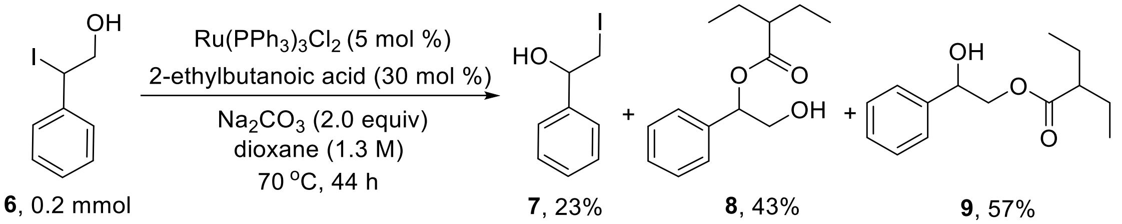

In a glove box, an oven-dried crimp-cap microwave vial equipped with a magnetic stirring bar was charged with $Ru(PPh_3)_3Cl_2$ (9.60 mg, 0.01 mmol), 2-ethylbutyric acid (7.50 uL, 0.06 mmol) and 2-iodo-2-phenylethan-1-ol **6** (49.6 mg, 0.20 mmol), then Na_2CO_3 (42.4 mg, 0.4 mmol) and dioxane (0.15 mL) were added. The vial was capped and taken out of glovebox, stirred at 70°C for 44 h. The reaction was then allowed to cool to room temperature and concentrated *in vacuo*. The NMR analysis of the crude by using dibromomethane as internal standard indicated that compound **7** was formed in 23% yield, **8** was formed in 43% yield (based on 2-ethylbutanoic acid as the limiting reagent) and **9** was formed in 57% yield (based on 2-ethylbutanoic acid as the limiting reagent).

The copy of NMR of the crude is presented as Supplementary Figure 6.

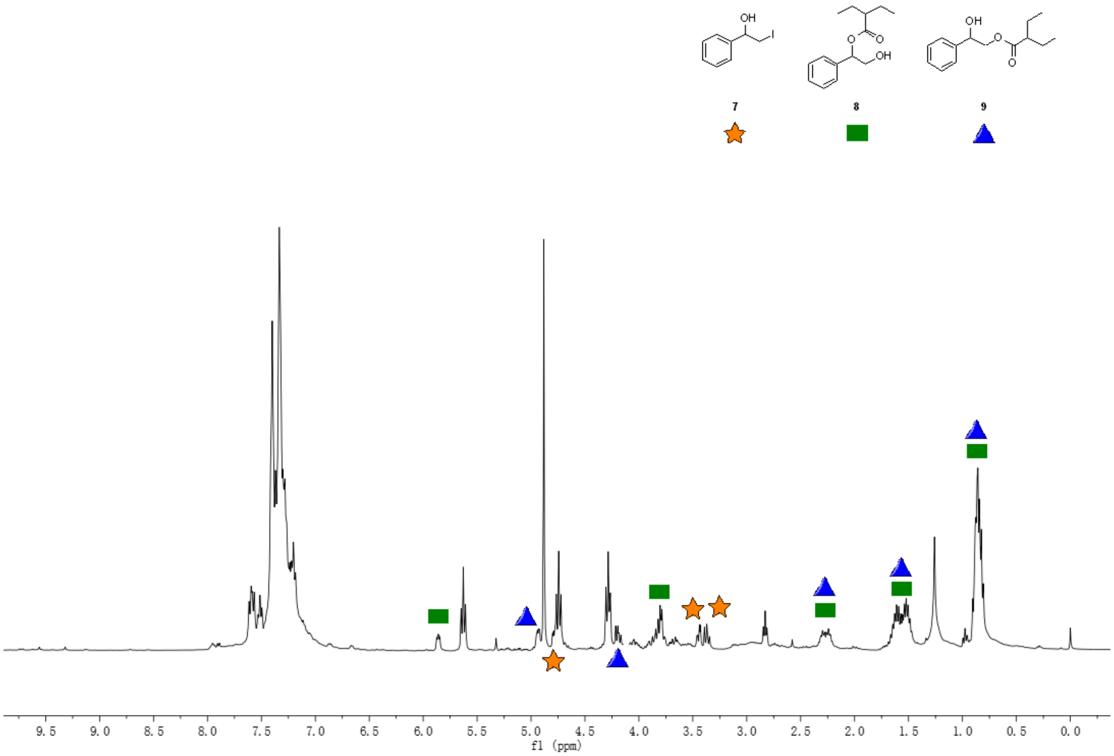

Supplementary Figure 6. Copy of NMR of crude reaction in Fig. 4E

6) For Fig. 4F in the main manuscript

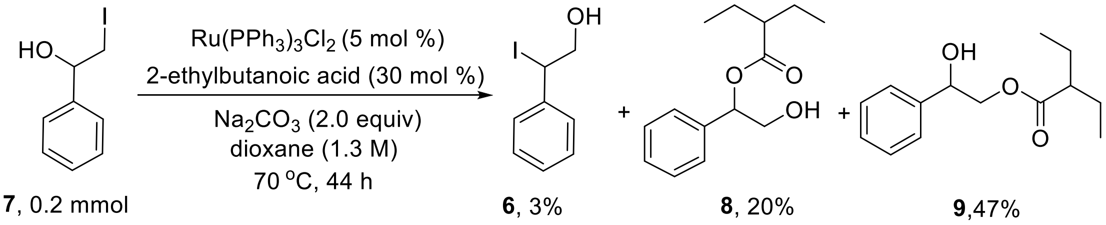

In a glove box, an oven-dried crimp-cap microwave vial equipped with a magnetic stirring bar was charged with $Ru(PPh_3)_3Cl_2$ (9.60 mg, 0.01 mmol), 2-ethylbutyric acid (7.50 uL, 0.06 mmol) and 2-iodo-1-phenylethan-1-ol **7** (49.6 mg, 0.20 mmol), then Na_2CO_3 (42.4 mg, 0.4 mmol) and dioxane (0.15 mL) were added. The vial was capped and taken out of glovebox, stirred at 70°C for 44 h. The reaction was then allowed to cool to room temperature and concentrated *in vacuo*. The NMR analysis of the crude by using dibromomethane as internal standard indicated that compound **6** was formed in 3% yield, **8** was formed in 20% yield (based on 2-ethylbutanoic acid as the limiting reagent) and **9** was formed in 47% yield (based on 2-ethylbutanoic acid as the limiting reagent).

The copy of NMR of the crude is presented as Supplementary Figure 7.

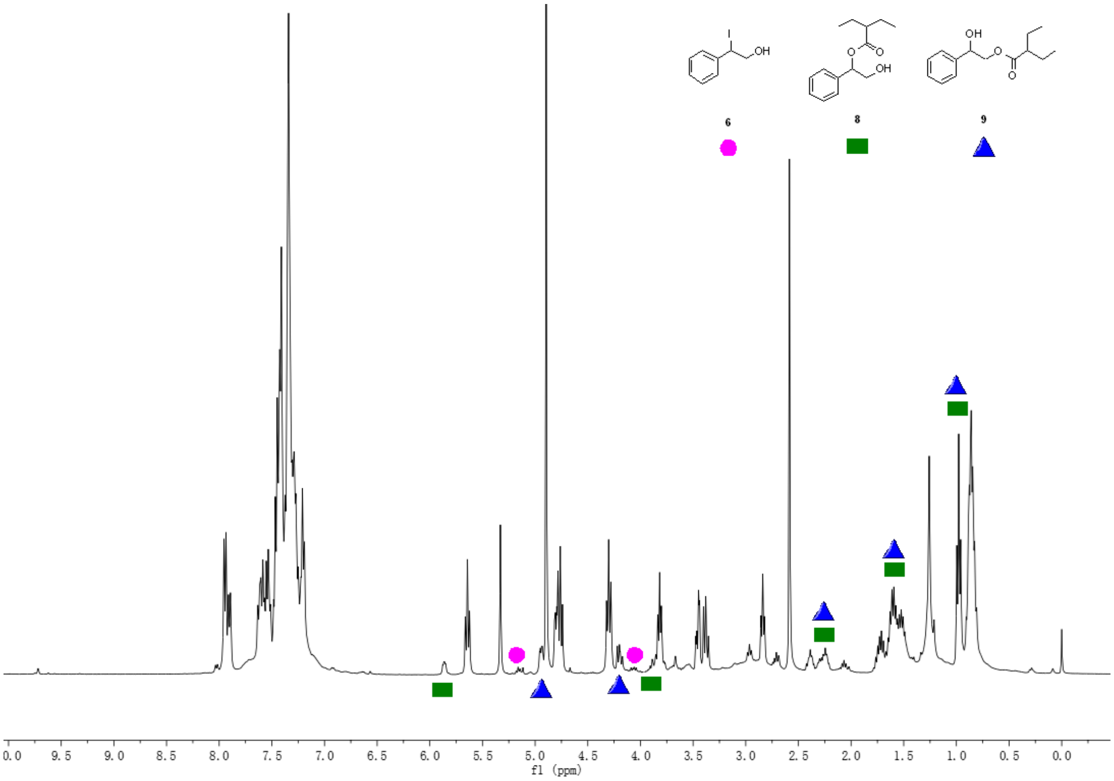

Supplementary Figure 7. Copy of NMR of crude reaction in Fig. 4F

7) For Scheme Fig. 4G in the main manuscript

In a glove box, oven-dried crimp-cap microwave vials equipped with a magnetic stirring bar were charged with $Ru(PPh_3)_3Cl_2$ (9.60 mg, 0.01 mmol), substrate **1a** (32.0 uL, 0.20 mmol), **8** (94.4 mg, 0.40 mmol) and other additives as presented in following Supplementary Table 6, then and dioxane (0.15 mL) was added to each vial. The vials were capped and taken out of glovebox, stirred at 70°C for 44 h. The reactions were then allowed to cool to room temperature and concentrated *in vacuo*. The NMR analysis of the crude from each vial were undertaken by using dibromomethane as internal standard and results were summarized in the Supplementary Table 6.

Supplementary Table 6. Mechanistic studies on **8** as the potential intermediate

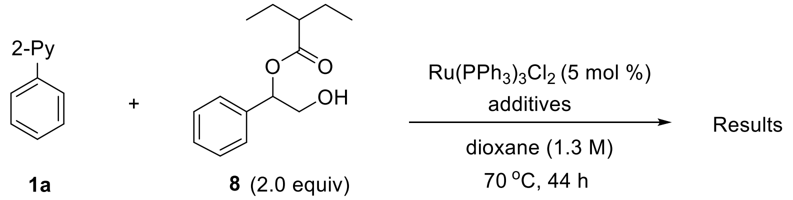

Entries	additives	Results [a]
1	NaI (1.0 eq)	**3**a, 0%; **8**, > 98% (recovered); **9**, 0%
2	Na_2CO_3 (2.0 equiv)	**3**a, 0%; **8**, 29% (recovered); **9**, 67%
3	NaI (1.0 eq) and Na_2CO_3 (2.0 equiv)	**3**a, 0%; **8**, 43% (recovered); **9**,54%

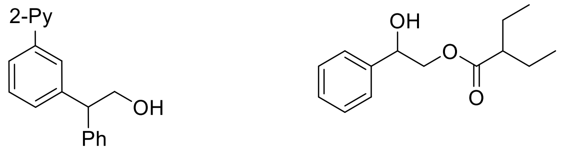

3a **9**

As an example, the copy of NMR of the crude from reaction in entry 2, Supplementary Table 6 is presented as Supplementary Figure 8.

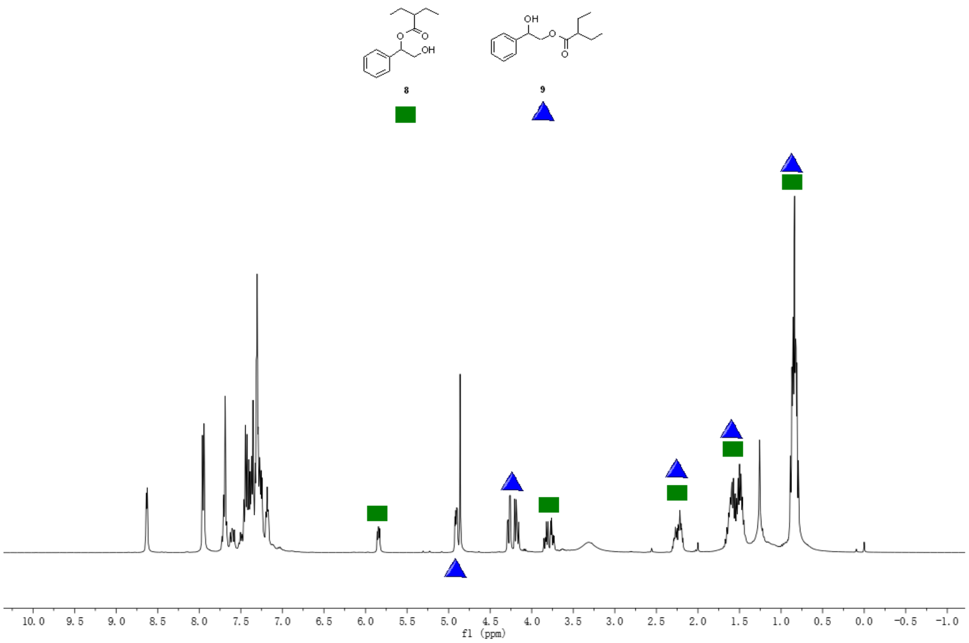

Supplementary Figure 8. Copy of NMR of crude reaction in entry 2, Supplementary Table 6

In a glove box, oven-dried crimp-cap microwave vials equipped with a magnetic stirring bar were charged with $Ru(PPh_3)_3Cl_2$ (9.60 mg, 0.01 mmol), substrate **1a** (32.0 uL, 0.20 mmol), **9** (94.4 mg, 0.40 mmol) and other additives as presented in following Supplementary Table 7, then and dioxane (0.15 mL) was added to each vial. The vials were capped and taken out of glovebox, stirred at 70°C for 44 h. The reactions were then allowed to cool to room temperature and concentrated *in vacuo*. The NMR analysis of the crude from each vial were undertaken and the results were summarized in the Supplementary Table 7.

Supplementary Table 7. Mechanistic studies on **9** as the potential intermediate

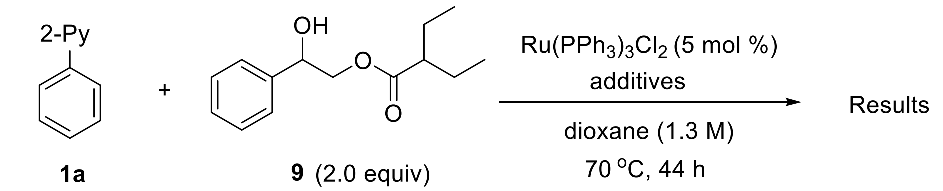

Entries	additives	Results [a]
1	NaI (1.0 eq)	**3**a, 0%; **9**, > 98% (recovered); **8**, 0%
2	Na_2CO_3 (2.0 equiv)	**3**a, 0%; **9**, 78% (recovered); **8**, 20%
3	NaI (1.0 eq) and Na_2CO_3 (2.0 equiv)	**3**a, 0%; **9**, 92% (recovered); **8**, 7%

[a] Determined by ^{1}HNMR analysis of crude material, and yields were reported based on compound **9**.

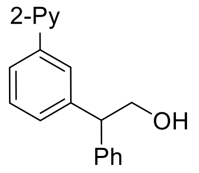

3a

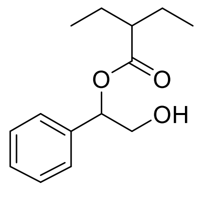

8

As an example, the copy of NMR of the crude from reaction in entry 2, Supplementary Table 7 is presented as Supplementary Figure 9.

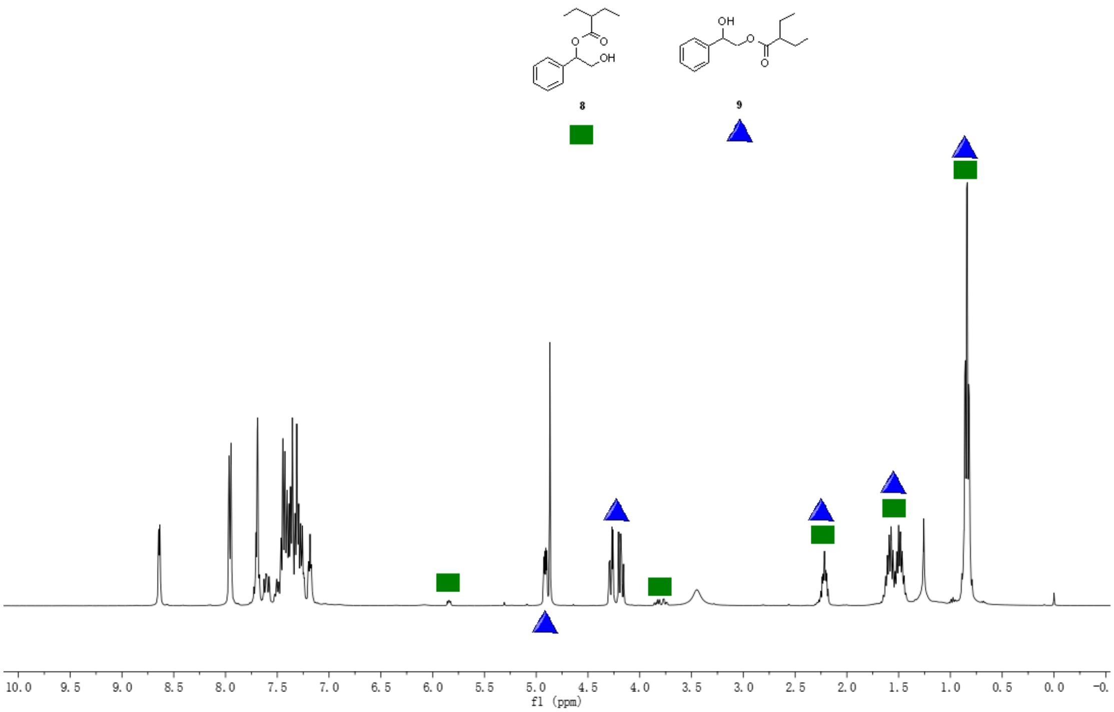

Supplementary Figure 9. Copy of NMR of crude reaction in entry 2, Supplementary Table 7.

8) For Fig. 4H in the main manuscript

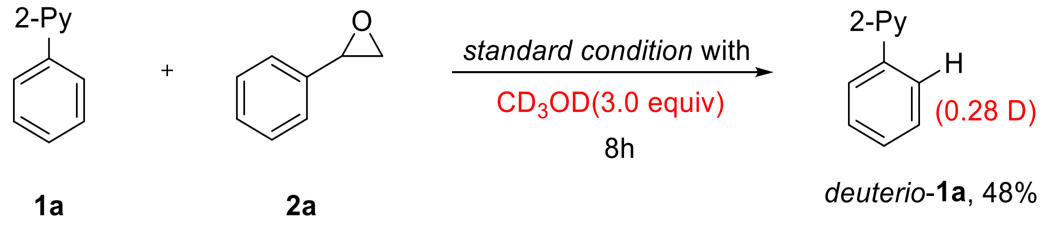

In a glove box, an oven-dried crimp-cap microwave vial equipped with a magnetic stirring bar was charged with $Ru(PPh_3)_3Cl_2$ (9.60 mg, 0.01 mmol), NaI powder (30.0 mg, 0.20 mmol) and 2-ethylbutyric acid (7.50 uL, 0.06 mmol), then substrate **1a** (32.0 uL, 0.20 mmol), epoxide **2a** (46 uL, 0.40 mmol), CD_3OD (24.4 uL, 0.60 mmol) and dioxane (0.15 mL) were added. The vial was then capped and taken out of glovebox, stirred at 70 °C for 8 h. The reaction was then allowed to cool to room temperature and concentrated *in vacuo*. The residue was purified by column chromatography (20% EtOAc/Hex) to yield the

recovered starting material deuterio **1a** (15.0 mg, 48%) as a colorless oil. The ^{1}H NMR analysis of **1a** indicates partial deutration of the *ortho*-C-H bond (28% D).

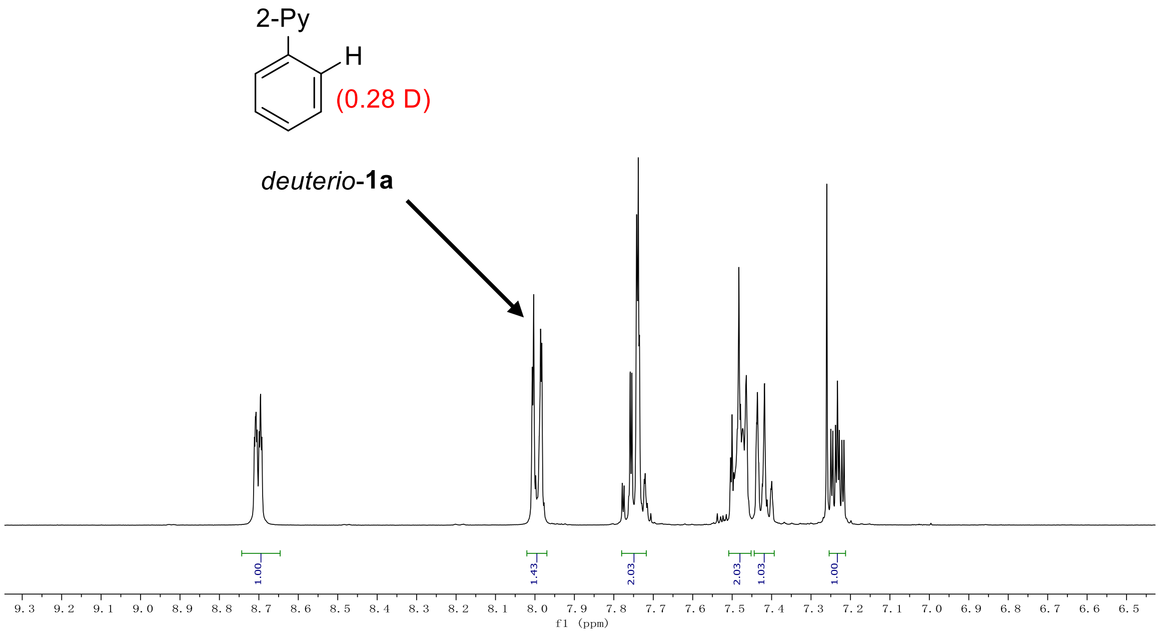

Supplementary Figure 10. Copy of NMR of recovered starting material in CD_3OD involved reaction

9) For Fig. 4I in the main manuscript

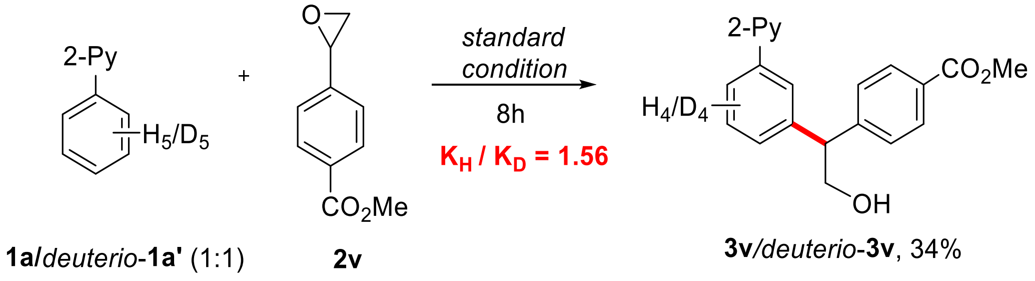

In a glove box, an oven-dried crimp-cap microwave vial equipped with a magnetic stirring bar was charged with $Ru(PPh_3)_3Cl_2$ (9.60 mg, 0.01 mmol), NaI powder (30.0 mg, 0.20 mmol) and 2-ethylbutyric acid (7.50 uL, 0.06 mmol), then substrate **1a** (15.5 mg, 0.10 mmol) , deuterated-**1a’** (20.0 mg, 0.10 mmol), epoxide **2a** (46 uL, 0.40 mmol) and dioxane

(0.15 mL) were added. The vial was then capped and taken out of glovebox, stirred at 70°C for 8 h. The reaction was then allowed to cool to room temperature and concentrated *in vacuo*. The residue was purified by column chromatography (40% EtOAc/Hex) to yield **3v** and deuterio-**3v** (in total 22.5 mg, 34%) as a colorless oil. NMR analysis of the products indicates a ratio of 0.61:0.39 between **3v** and deuterio-**3v**. *The analysis was using the ratio of H_a and D_a in the ^1H NMR to determine the ratio of **3v** and deuterio-**3v**, since its signal is isolated from others and is not likely to go through H/D exchanged during the reaction.*

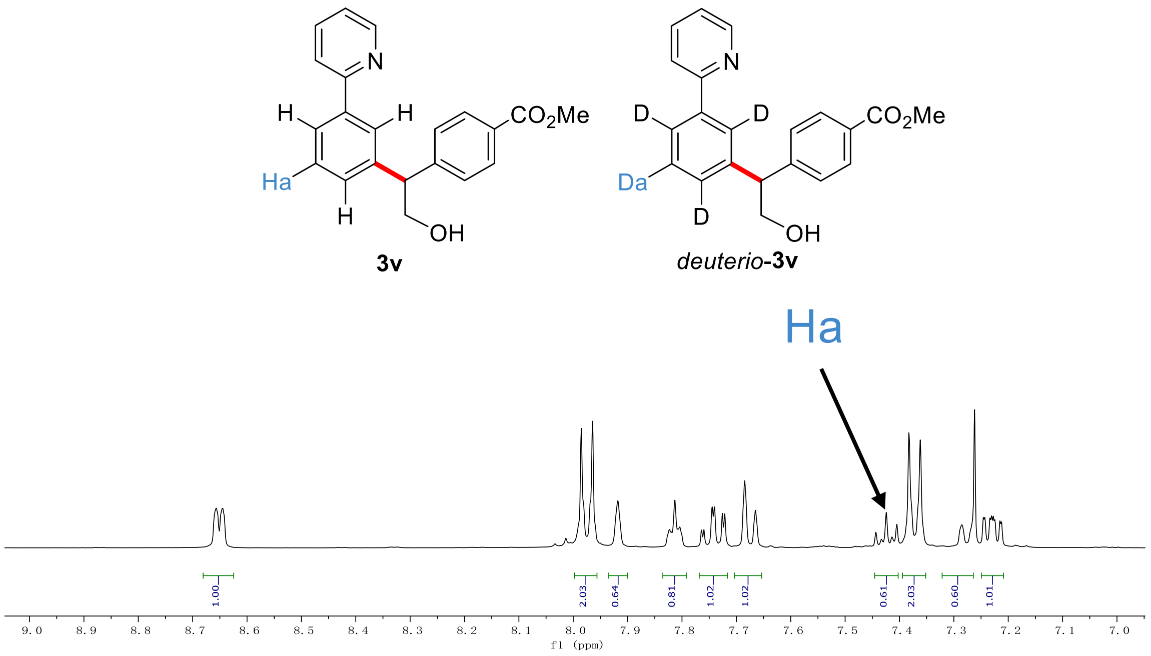

Supplementary Figure 11. Copy of NMR of intermolecular kinetic isotope experiment

2. Mechanistic Studies of Oxetane Involved *meta*-Alkylation Reaction

1)

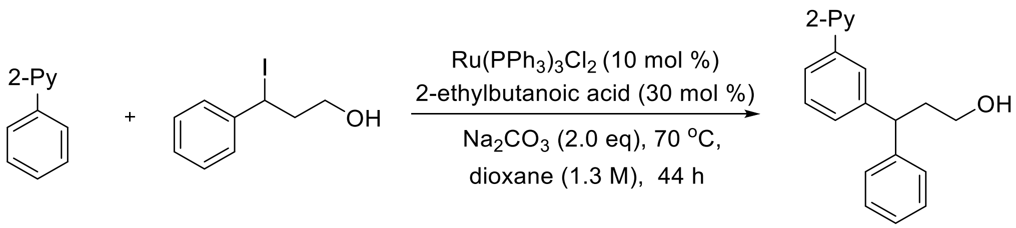

1a **10** (4.0 eq) **5a**, 46%

In a glove box, an oven-dried crimp-cap microwave vial equipped with a magnetic stirring bar was charged with $Ru(PPh_3)_3Cl_2$ (9.60 mg, 0.01 mmol) and 2-ethylbutyric acid (7.50 uL, 0.06 mmol), then substrate **1a** (32.0 uL, 0.20 mmol), 3-iodo-3-phenylpropan-1-ol **10** (210.0 mg, 0.80 mmol), Na_2CO_3 (42.4 mg, 0.4 mmol) and dioxane (0.15 mL) were added. The vial was then capped and taken out of glovebox, stirred at 70°C for 44 h. The reaction was then allowed to cool to room temperature and concentrated *in vacuo*. The crude mixture was purified by column chromatography (40% EtOAc/Hex) to yield the title compound **5a** (26.5 mg, 46%) as a colorless oil.

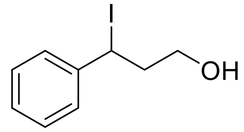

10

3-iodo-3-phenylpropan-1-ol **10** was prepared according to the literature report.[25] ^{1}H NMR (400 MHz, $CDCl_3$) δ 7.41 – 7.36 (m, 2H), 7.30 – 7.25 (m, 2H), 7.24 – 7.19 (m, 1H), 5.34 (dd, *J* = 9.1, 6.3 Hz, 1H), 3.74 – 3.55 (m, 2H), 2.69 (s, 1H), 2.56 –2.47 (m, 1H), 2.23 – 2.10 (m, 1H). ^{13}C NMR (101 MHz, $CDCl_3$) δ 143.7, 128.8, 128.1, 127.2, 61.94, 43.3, 30.3. *Note: this compound is unstable towards silica and need to be purified by a fast column using neutral Al_2O_3 (30% EtOAc/Hex).*

2)

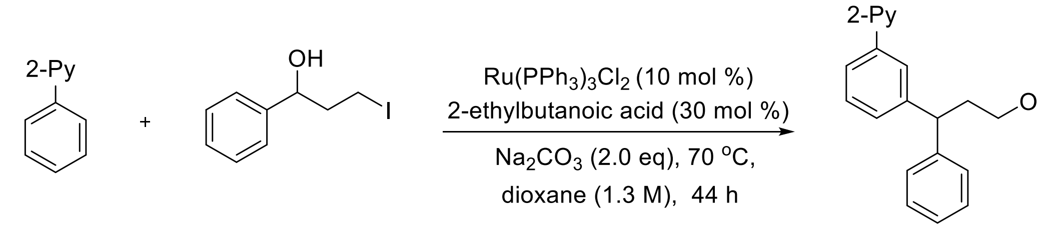

1a **11** (4.0 eq) **5a**, 0%

In a glove box, an oven-dried crimp-cap microwave vial equipped with a magnetic stirring bar was charged with $Ru(PPh_3)_3Cl_2$ (9.60 mg, 0.01 mmol) and 2-ethylbutyric acid (7.50 uL, 0.06 mmol), then substrate **1a** (32.0 uL, 0.20 mmol), 3-iodo-1-phenylpropan-1-ol **11** (210.0 mg, 0.80 mmol), Na_2CO_3 (42.4 mg, 0.4 mmol) and dioxane (0.15 mL) were added. The vial was then capped and taken out of glovebox, stirred at 70°C for 44 h. The reaction was then allowed to cool to room temperature and concentrated *in vacuo*. The NMR analysis of the crude indicated that **5a** was not formed.

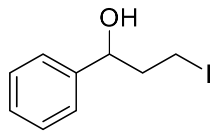

11

3-iodo-1-phenylpropan-1-ol **11** was prepared according to the literature report.[26] ^1H NMR (400 MHz, $CDCl_3$) δ 7.32 – 7.13 (m, 5H), 4.77 (dd, J = 8.3, 4.9 Hz, 1H), 3.58 (ddd, J = 10.8, 8.0, 5.9 Hz, 1H), 3.48 – 3.34 (m, 1H), 3.24 (s, 1H), 2.18 – 2.03 (m, 1H), 2.01 – 1.87 (m, 1H).

3)

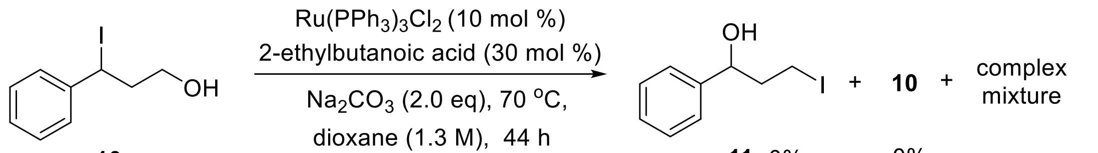

10 **11**, 0% 0%

In a glove box, an oven-dried crimp-cap microwave vial equipped with a magnetic stirring bar was charged with $Ru(PPh_3)_3Cl_2$ (9.60 mg, 0.01 mmol) and 2-ethylbutyric acid (7.50 uL, 0.06 mmol), then 3-iodo-3-phenylpropan-1-ol **10** (210.0 mg, 0.80 mmol), Na_2CO_3 (42.4 mg, 0.4 mmol) and dioxane (0.15 mL) were added. The vial was then capped and taken out of glovebox, stirred at 70°C for 44 h. The reaction was then allowed to cool to room temperature and concentrated *in vacuo*. The NMR analysis of the crude indicated that **11** and **10** were not formed, and a complex mixture was formed.

4)

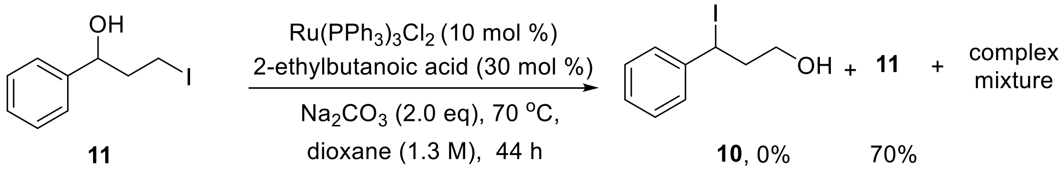

11 **10**, 0% 70%

In a glove box, an oven-dried crimp-cap microwave vial equipped with a magnetic stirring bar was charged with $Ru(PPh_3)_3Cl_2$ (9.60 mg, 0.01 mmol) and 2-ethylbutyric acid (7.50 uL, 0.06 mmol), then 3-iodo-1-phenylpropan-1-ol **11** (210.0 mg, 0.80 mmol), Na_2CO_3 (42.4 mg, 0.4 mmol) and dioxane (0.15 mL) were added. The vial was then capped and taken out of glovebox, stirred at 70°C for 44 h. The reaction was then allowed to cool to room temperature and concentrated *in vacuo*. The NMR analysis of the crude by using dibromomethane as internal standard indicated that compound **10** was not formed and **11** was recovered in 70% yield.

5)

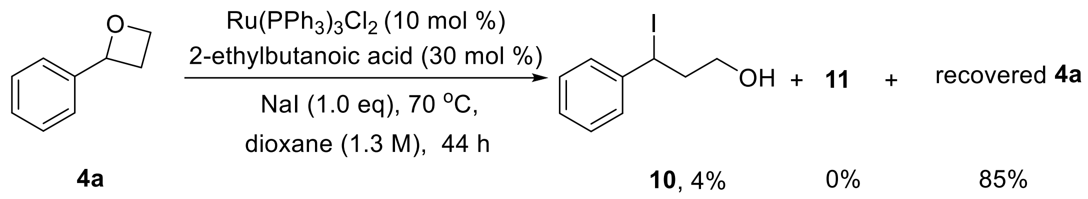

In a glove box, an oven-dried crimp-cap microwave vial equipped with a magnetic stirring bar was charged with $Ru(PPh_3)_3Cl_2$ (9.60 mg, 0.01 mmol) and 2-ethylbutyric acid (7.50 uL, 0.06 mmol), then 2-phenyloxetane **4a** (107.2 mg, 0.80 mmol), NaI (30.0 mg, 0.2 mmol) and dioxane (0.15 mL) were added. The vial was then capped and taken out of glovebox, stirred at 70°C for 44 h. The reaction was then allowed to cool to room temperature and concentrated *in vacuo*. The NMR analysis of the crude by using dibromomethane as internal standard indicated that compound **10** was formed in 4% yield, **11** was not formed and **4a** was recovered in 85% yield.

The copy of NMR of the crude for this reaction is presented as Supplementary Figure 12.

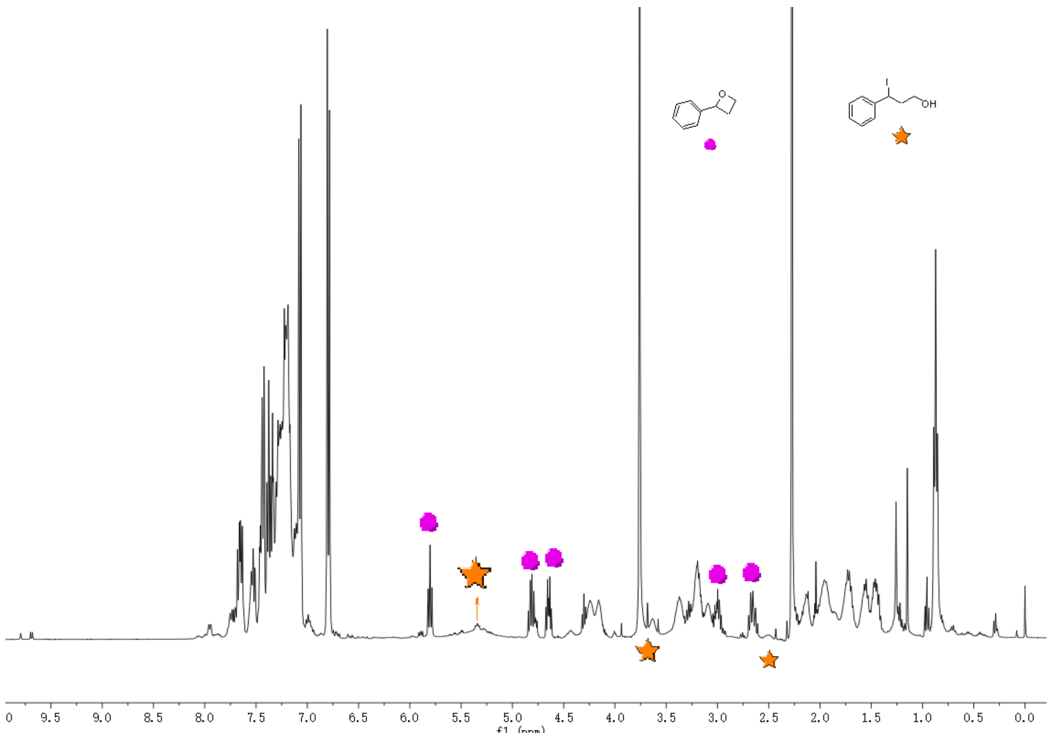

Supplementary Figure 12. Copy of NMR of crude reaction for 2-phenyloxetane opening study

6)

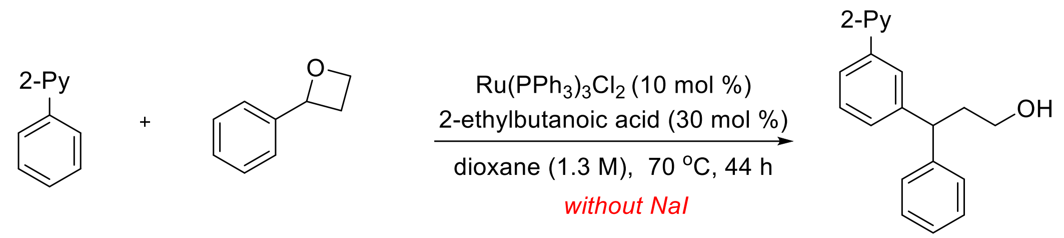

1a **4a** (4.0 eq) **5a**,0%

In a glove box, an oven-dried crimp-cap microwave vial equipped with a magnetic stirring bar was charged with $Ru(PPh_3)_3Cl_2$ (9.60 mg, 0.01 mmol) and 2-ethylbutyric acid (7.5 uL, 0.06 mmol), then substrate **1a** (32.0 uL, 0.20 mmol), 2-phenyloxetane **4a** (107.2 mg, 0.80 mmol) and dioxane (0.15 mL) were added. The vial was then capped and taken out of glovebox, stirred at 70°C for 44 h. The reaction was then allowed to cool to room temperature and concentrated *in vacuo*. The NMR analysis of the crude indicated that **5a** was not formed.

Discussion: In eq 1, the reaction between 2-phenylpyridine **1a** and iodohydrin **10** occurs smoothly to yield **5a** in 46% yield, indicating that iodohydrin **10** may serve as the key intermediate for *meta*-alkylation of **1a** and 2-phenyloxetane **4**. This result parallels those obtained with the epoxide. However, while using the iodohydrin **11** under identical conditions, no detectable amount of **5a** is obtained (eq 2). Furthermore, iodohydrin **10** and **11** do not interconvert under the reaction conditions (eq 3 and eq 4). Moreover, the ring opening study of **4a** in eq 5 demonstrates that iodide promoted oxetane opening can only occur at more hindered benzylic C−O bond. These results are in stark contrast to those obtained with epoxides where a dynamic equilibrium was observed. Finally, the reaction does not occur in the absence of NaI (eq 6). Taken together, these results imply that iodohydrin **10** is generated as the single regioisomer in the reaction when 2-phenyloxetane reacts with iodide, and then functions as the intermediate in the *meta*-alkylation reaction.

3. Observations of Catalytic Intermediates

After heating $RuCl_2(PPh_3)_3$ with the carboxylic acid in dioxane, only PPh_3 and $OPPh_3$ were observed by ^{31}P NMR analysis. However, upon addition of a base (K_2CO_3), $Ru(O_2CR)_2(PPh_3)_2$ was also observed.

A

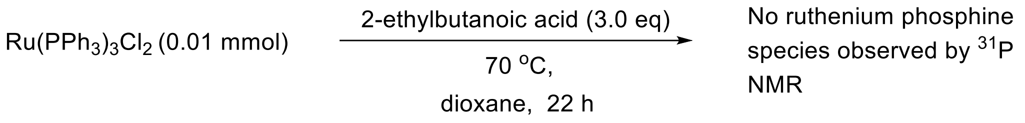

B

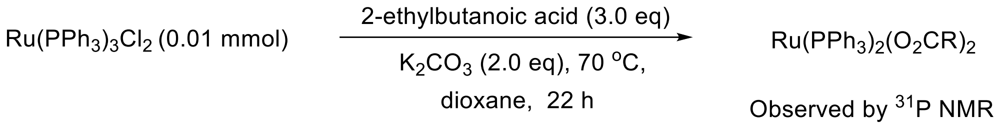

The standard reaction was stopped after 22 h, diluted in dioxane, filtered and submitted for ^{31}P NMR and mass spectral analysis. $Ru(O_2CR)_2(PPh_3)_2$ was not observed by either analysis.

A

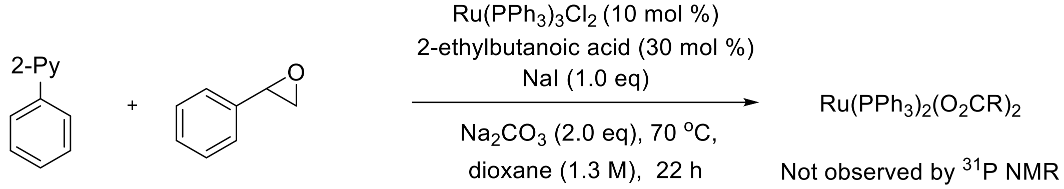

1a **2** (2.0 eq)

The ^{31}P spectrum shows two peaks (23.0 and 32.4 ppm) which are consistent with literature values for mono-cyclometalated phenylpyridine-ruthenium species, with one and two triphenylphosphines bound to the ruthenium respectively.[27] The slight difference in the chemical shift can be explained by change of solvent from d_3-acetonitrile to 1,4-dioxane, which can both coordinate to the ruthenium. There were an extra 2 peaks that could not be assigned at around 42 ppm, but this does not correspond to either $Ru(O_2CR)_2(PPh_3)_2$ or $RuCl(O_2CR)(PPh_3)_3$.[28,29]

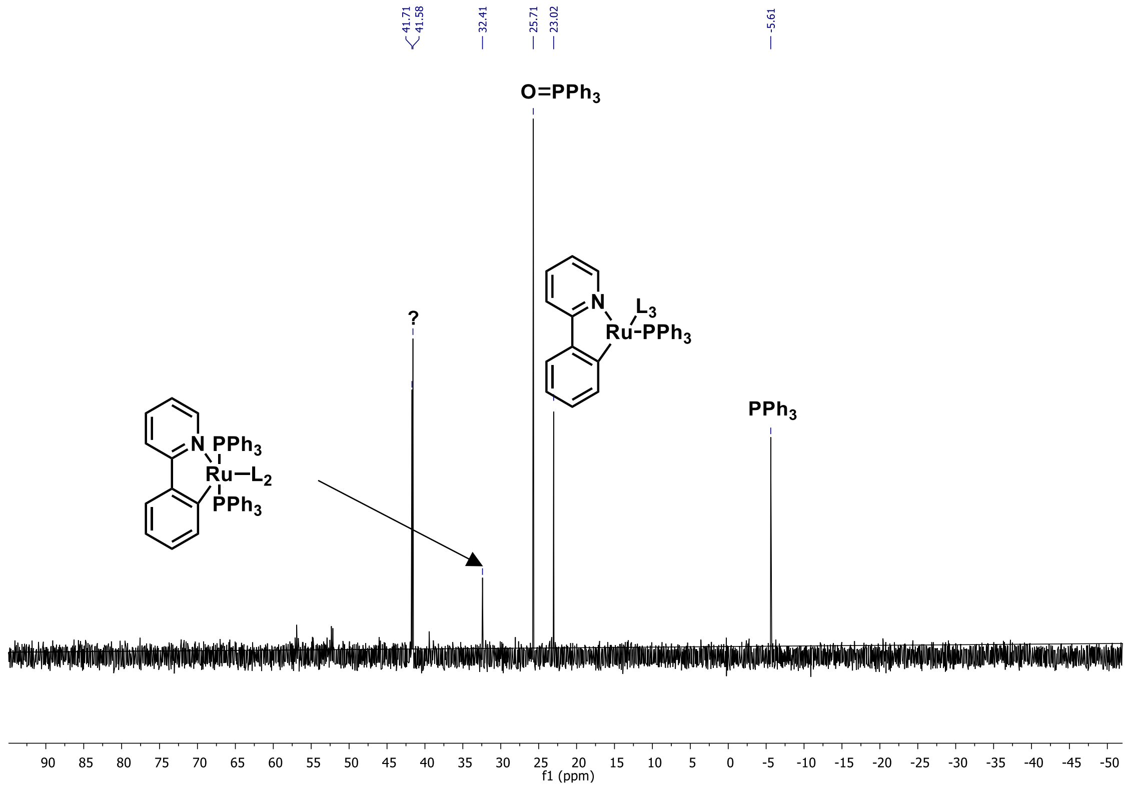

Supplementary Figure 13. The ^{31}P NMR spectrum of the reaction mixture

The ESI-MS of this reaction mixture shows the masses of monocyclometaled-phenylpyridines (both substrate and product) plus one and two triphenylphosphines. This spectrum is consistent with the tentative assignment of the above ^{31}P NMR.

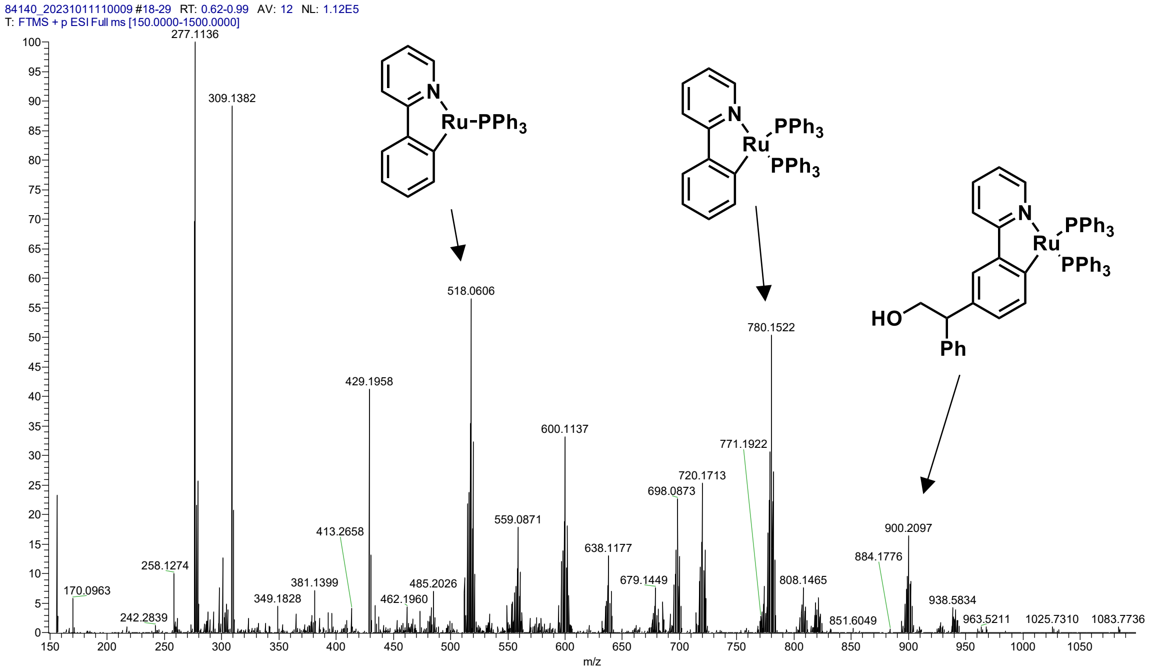

Supplementary Figure 14. The ESI-MS spectrum of reaction mixture

Formation of $Ru(O_2CR)_2(PPh_3)_2$ was attempted, but unfortunately purification of this complex proved difficult and was only able to be obtained as a mixture with PPh_3. This mixture was shown to be a competent precatalysis for the reaction, with and to a lesser extent without the acid. However, these results do not prove that this species is present in the reaction, either as an on-cycle or off-cycle intermediate.

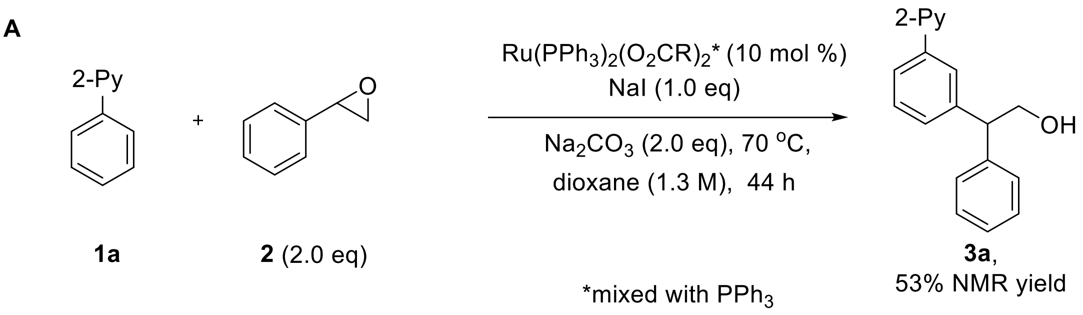

III. Supplementary Figures

Copies of ^{1}H and ^{13}C NMR for Novel Compounds

2-(4-(Methoxymethyl)phenyl)pyridine **1d**

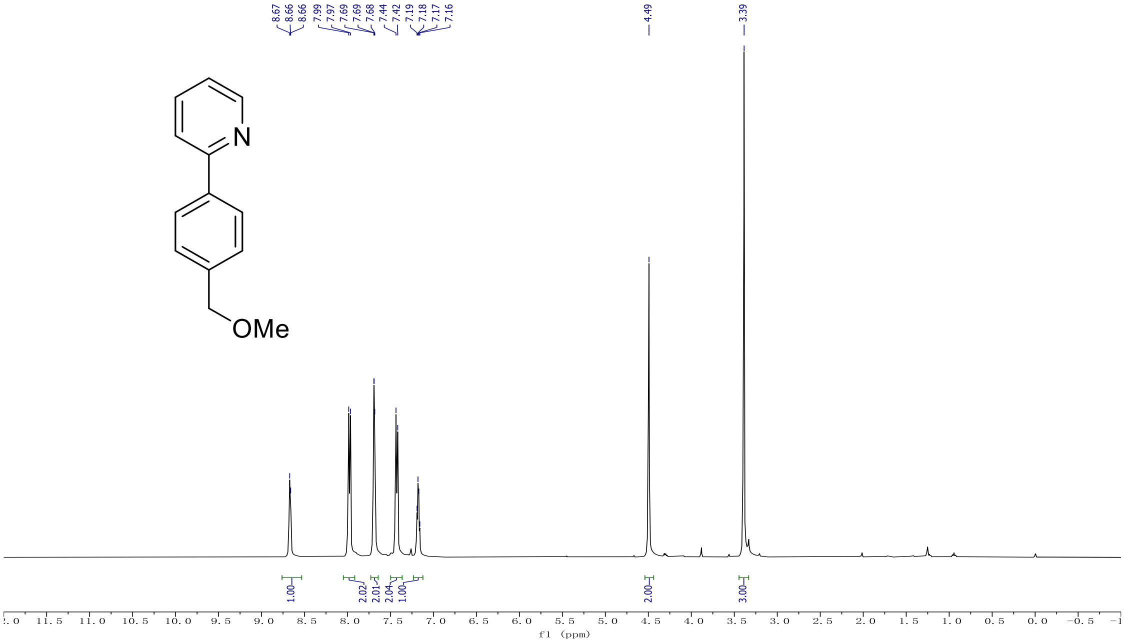

Supplementary Figure 15. ^{1}H NMR spectrum (400 MHz, $CDCl_3$, at rt) of **1d**

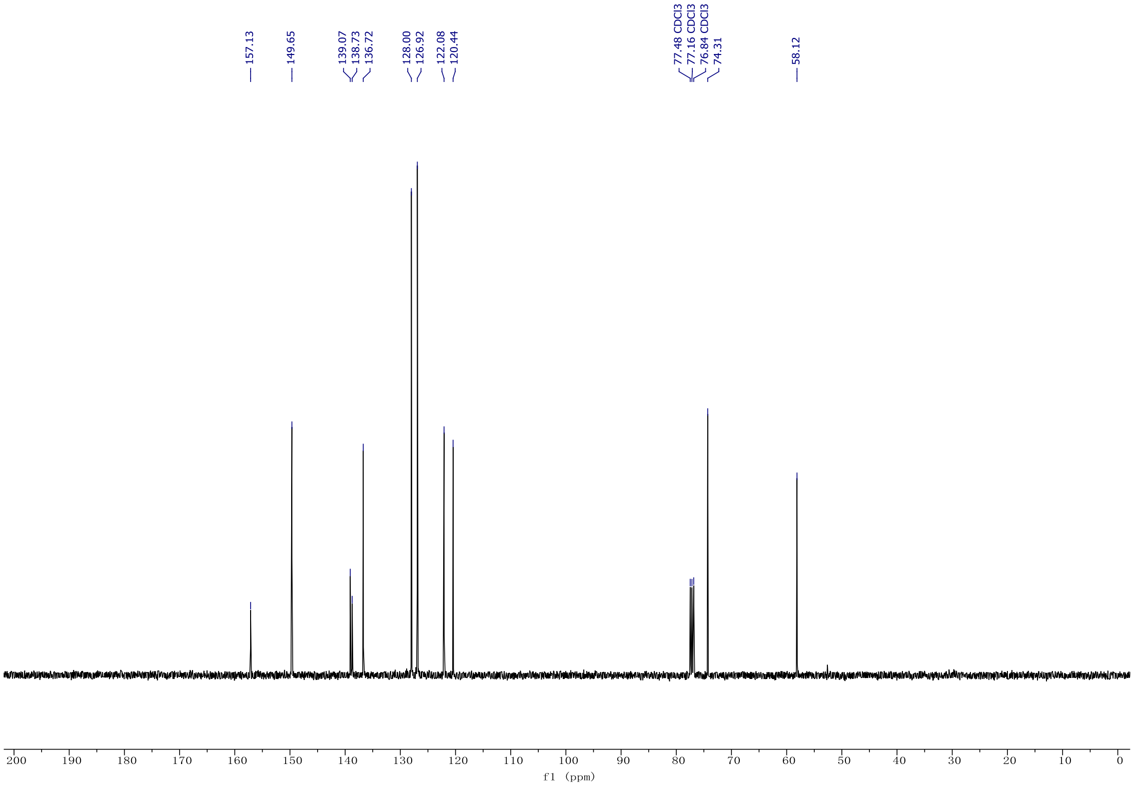

Supplementary Figure 16. ^{13}C NMR spectrum (101 MHz, $CDCl_3$, at rt) of **1d**

Methyl 3-(4-(pyridin-2-yl)phenyl)propanoate **1h**

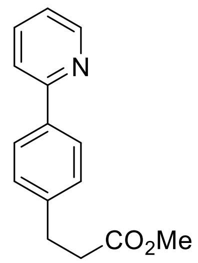

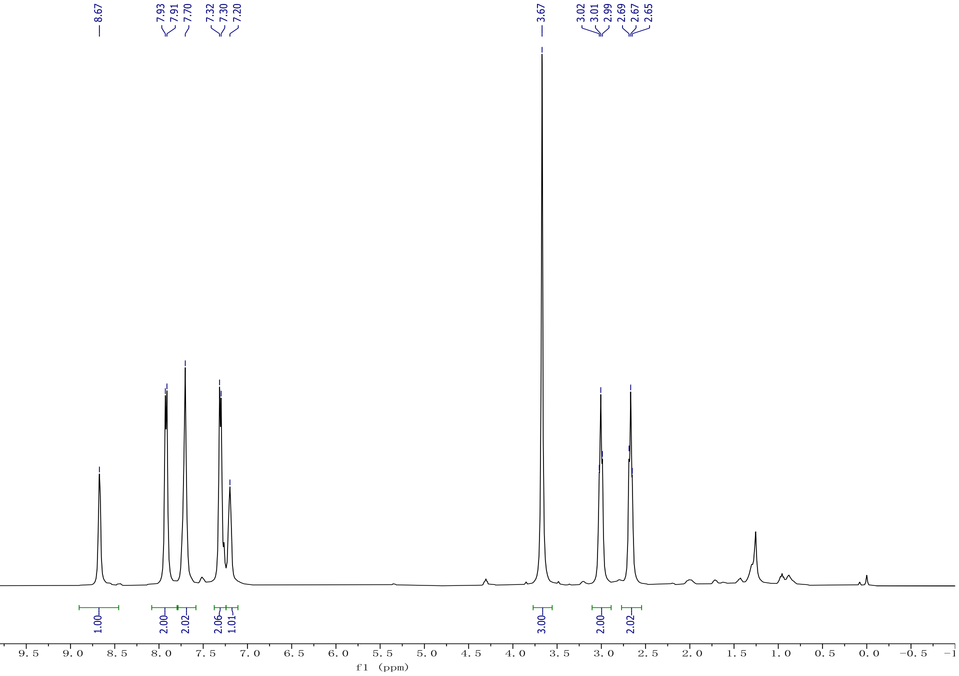

Supplementary Figure 17. ^{1}H NMR spectrum (400 MHz, $CDCl_3$, at rt) of **1h**

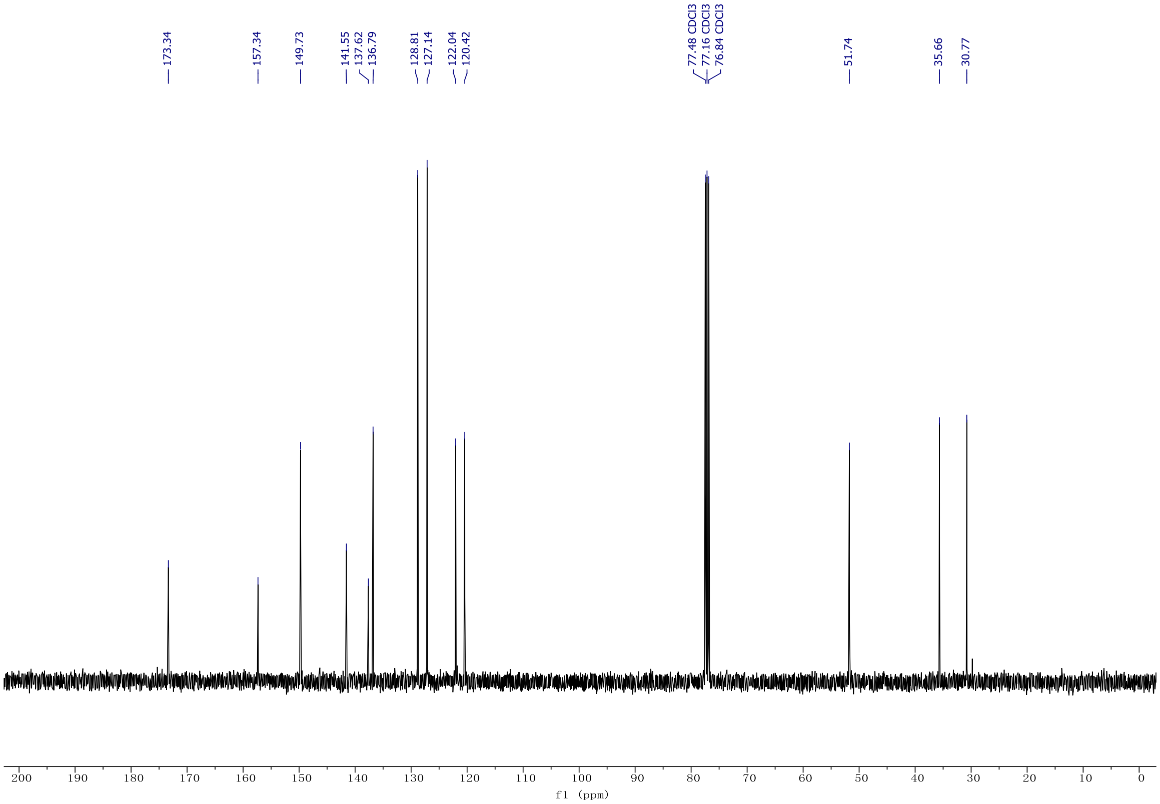

Supplementary Figure 18. ^{13}C NMR spectrum (101 MHz, $CDCl_3$, at rt) of **1h**

3-(4-Cyclohexylphenyl)oxirane **2o**

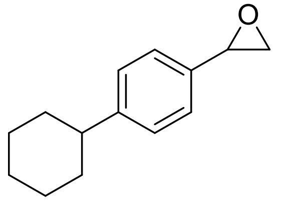

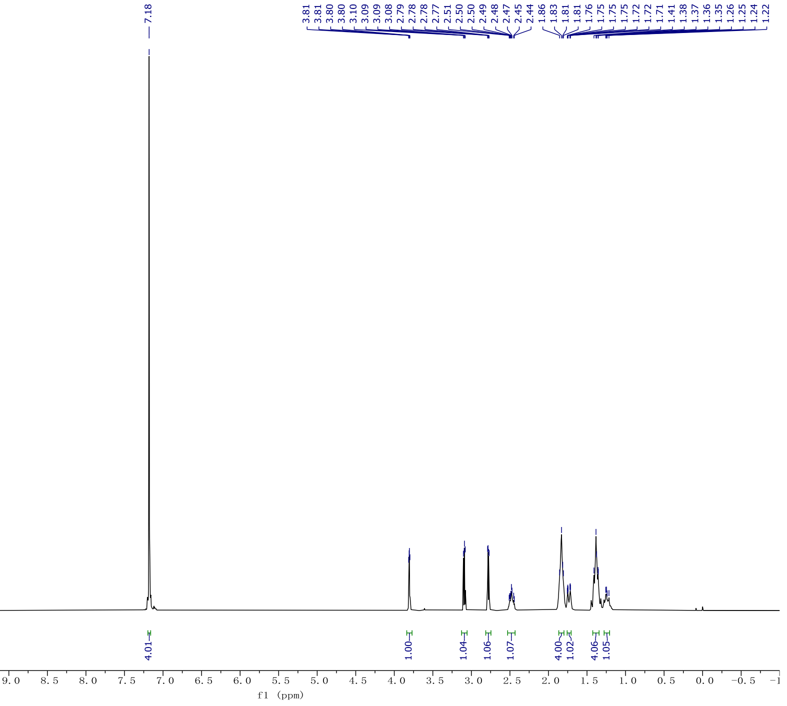

Supplementary Figure 19. ^{1}H NMR spectrum (400 MHz, $CDCl_3$, at rt) of **2o**

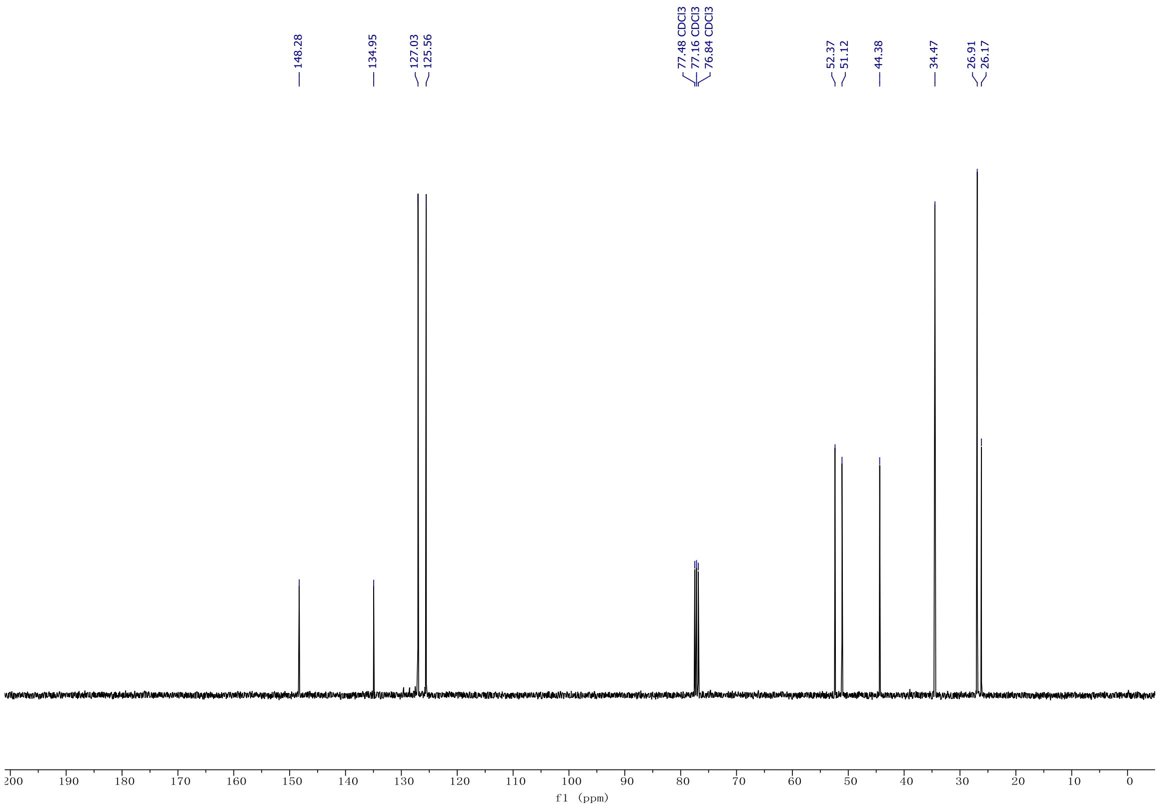

Supplementary Figure 20. ^{13}C NMR spectrum (101 MHz, $CDCl_3$, at rt) of **2o**

5-(Oxiran-2-yl)-2,3-dihydro-*1H*-inden-1-one **2ac**

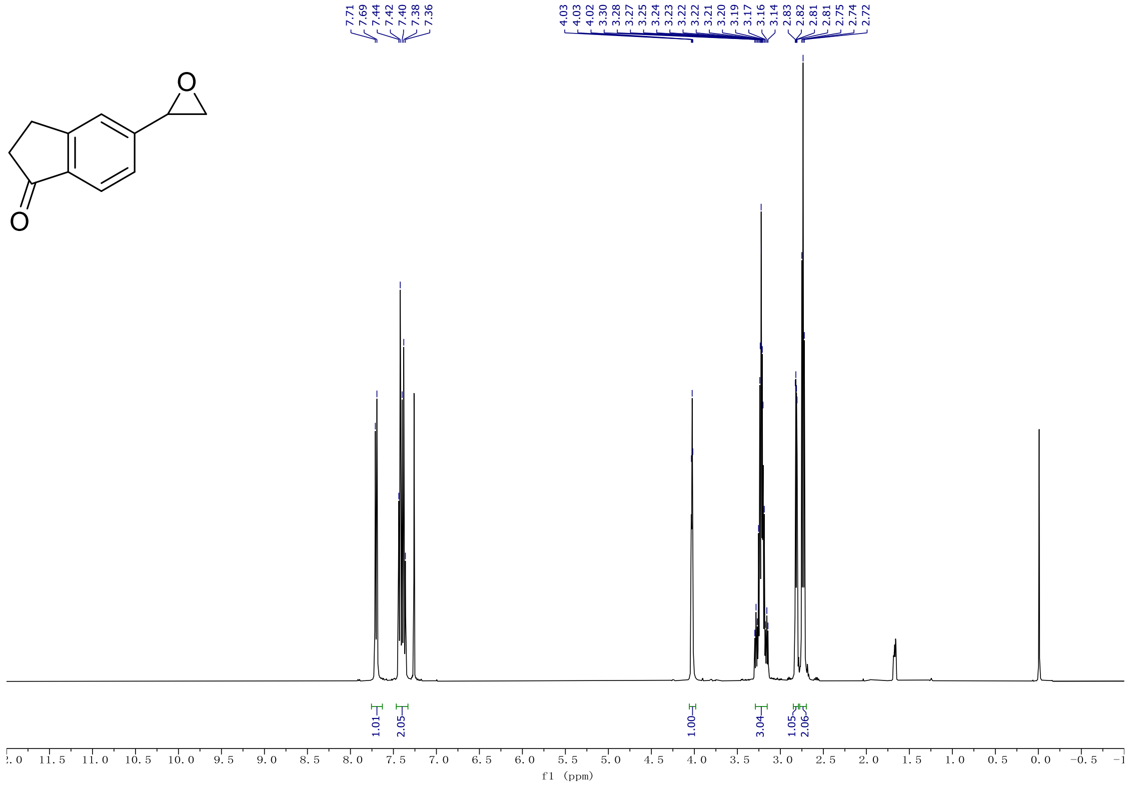

Supplementary Figure 21. ^{1}H NMR spectrum (400 MHz, $CDCl_3$, at rt) of **2ac**

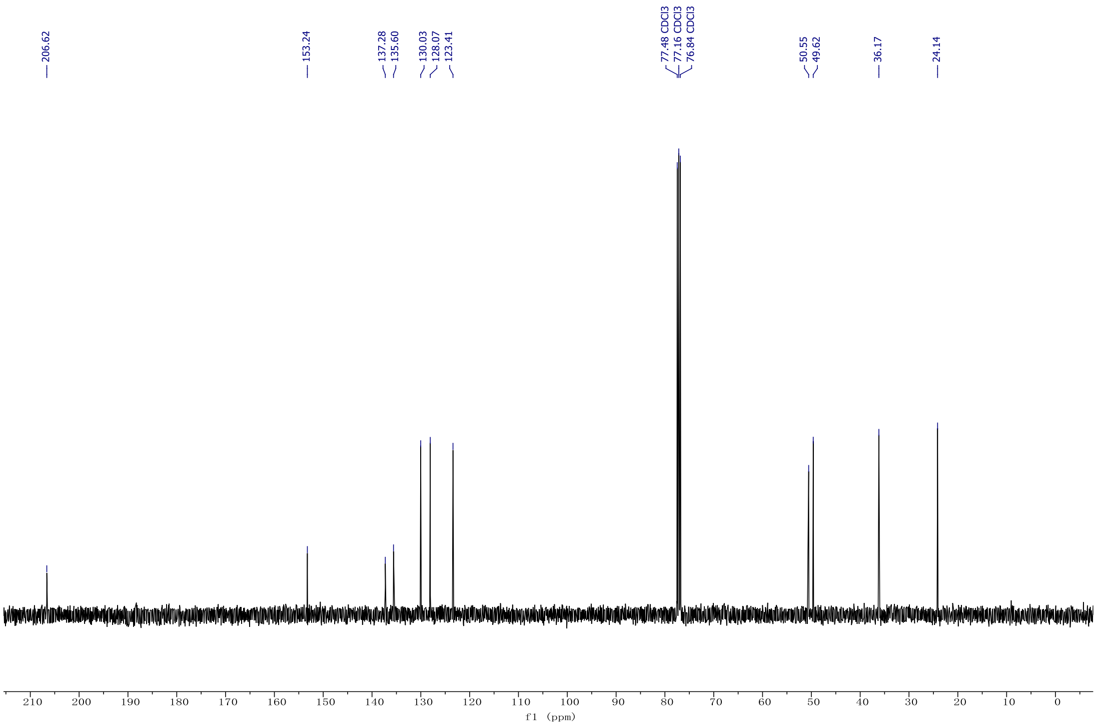

Supplementary Figure 22. ^{13}C NMR spectrum (101 MHz, $CDCl_3$, at rt) of **2ac**

2-([1,1'-Binaphthalen]-2-yl)oxirane **2ae** (two diastereomers mixed, 1:0.6 ratio)

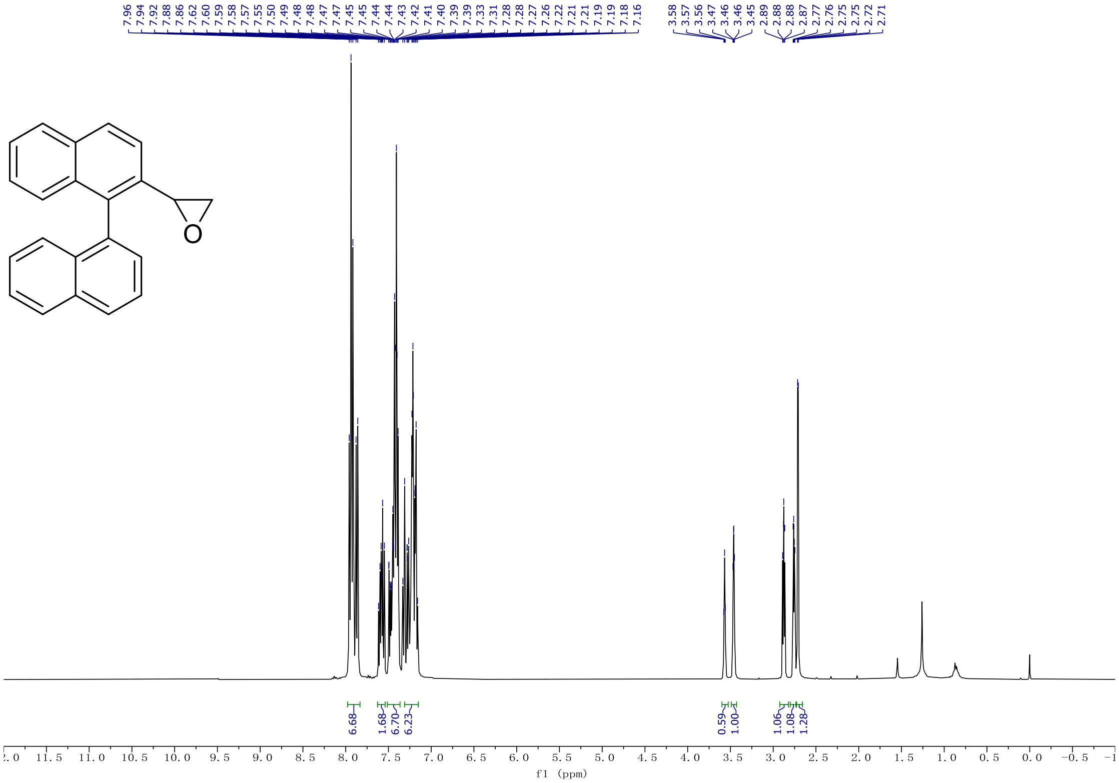

Supplementary Figure 23. ^{1}H NMR spectrum (400 MHz, $CDCl_3$, at rt) of **2ae**

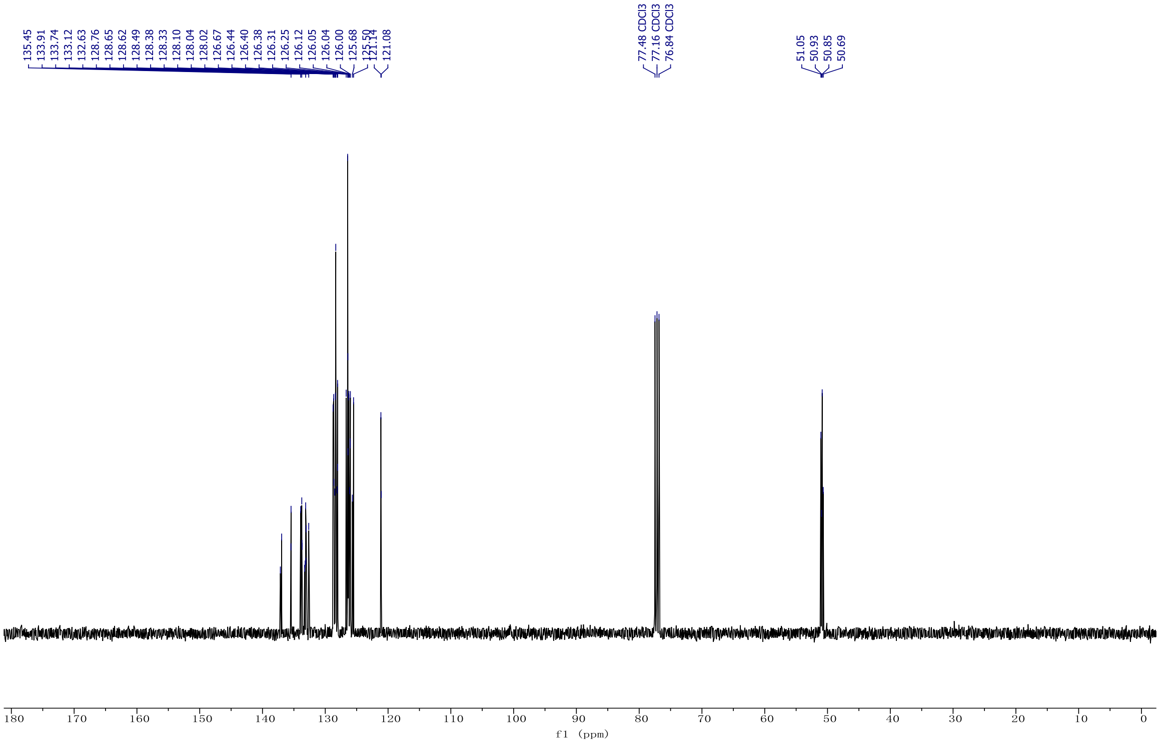

Supplementary Figure 24. ^{13}C NMR spectrum (101 MHz, $CDCl_3$, at rt) of **2ae**

3-(Oxiran-2-yl)furan **2af**

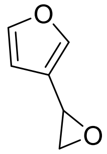

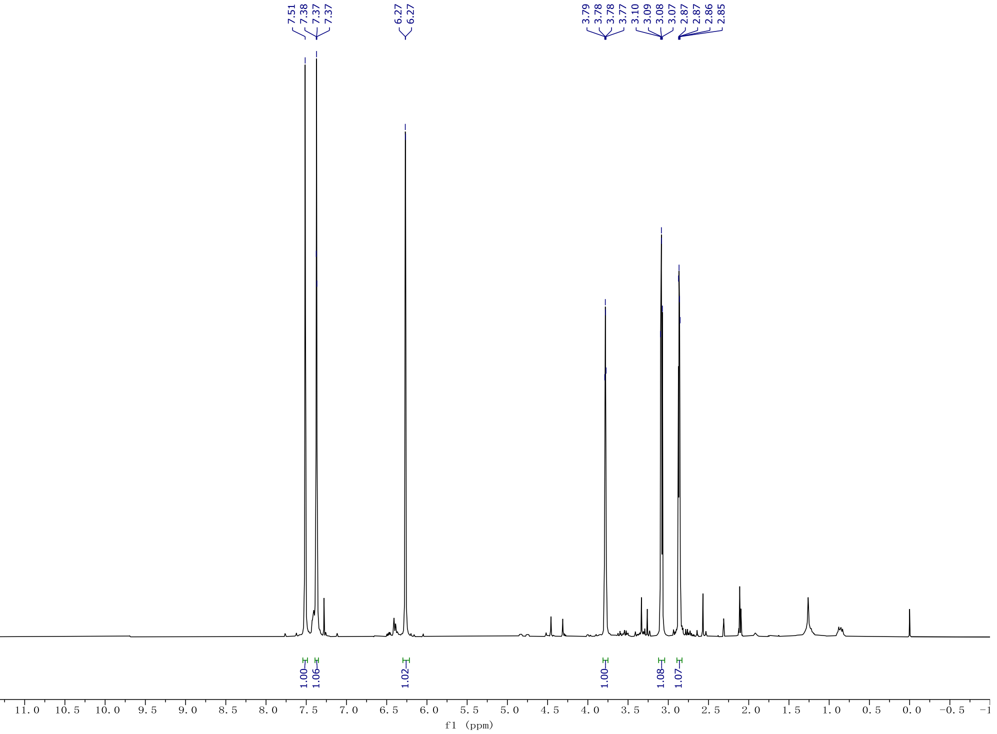

Supplementary Figure 25. ^{1}H NMR spectrum (400 MHz, $CDCl_3$, at rt) of **2af**

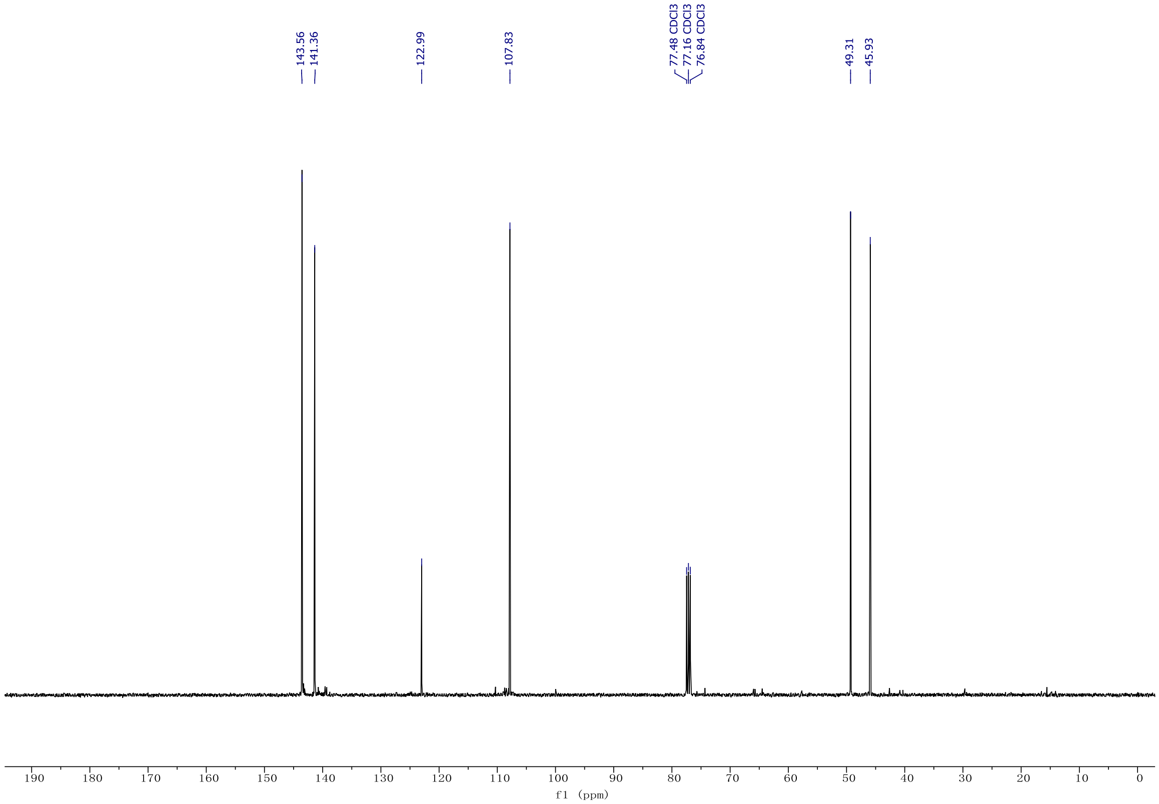

Supplementary Figure 26. ^{13}C NMR spectrum (101 MHz, $CDCl_3$, at rt) of **2af**

2-(Thiophen-3-yl)oxirane **2ag**

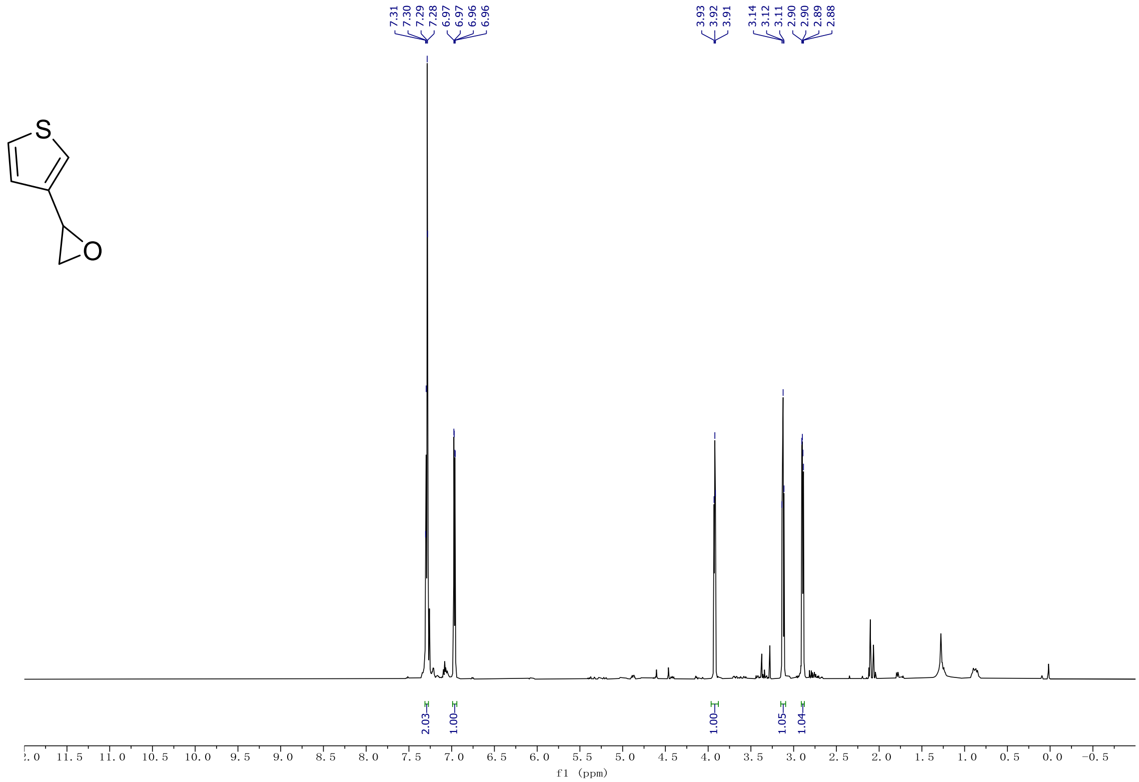

Supplementary Figure 27. ^{1}H NMR spectrum (400 MHz, $CDCl_3$, at rt) of **2ag**

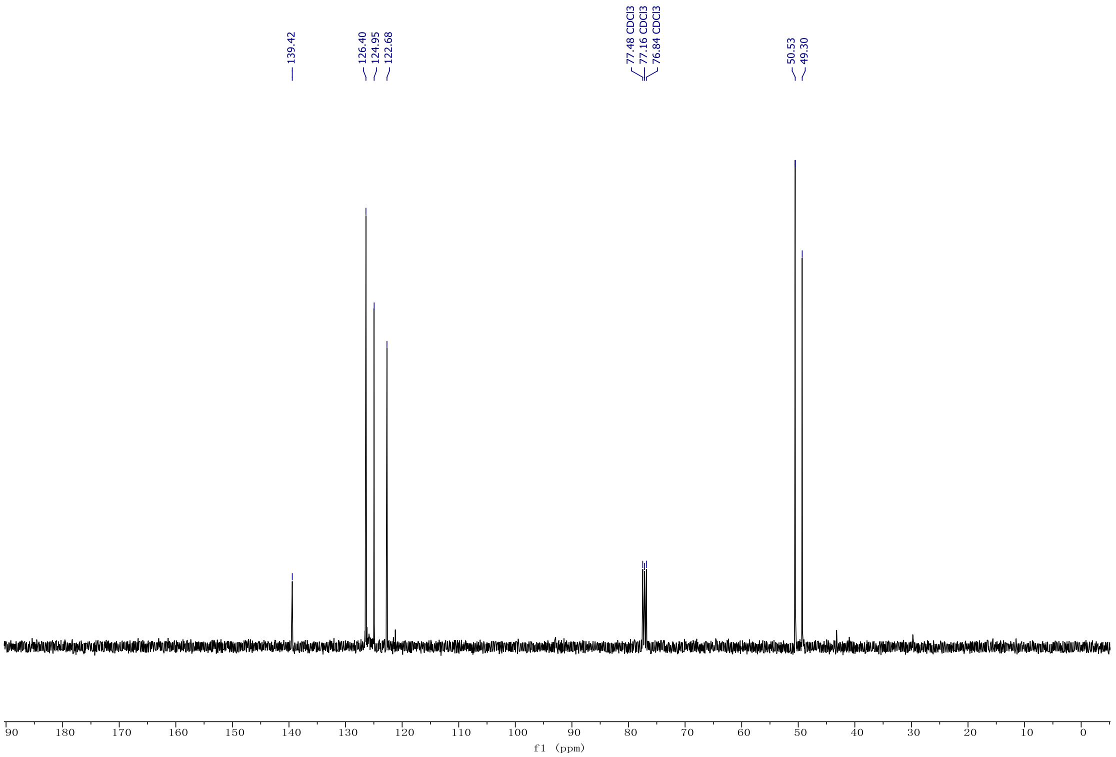

Supplementary Figure 28. ^{13}C NMR spectrum (101 MHz, $CDCl_3$, at rt) of **2ag**

7-(Oxiran-2-yl)quinoline **2ah**

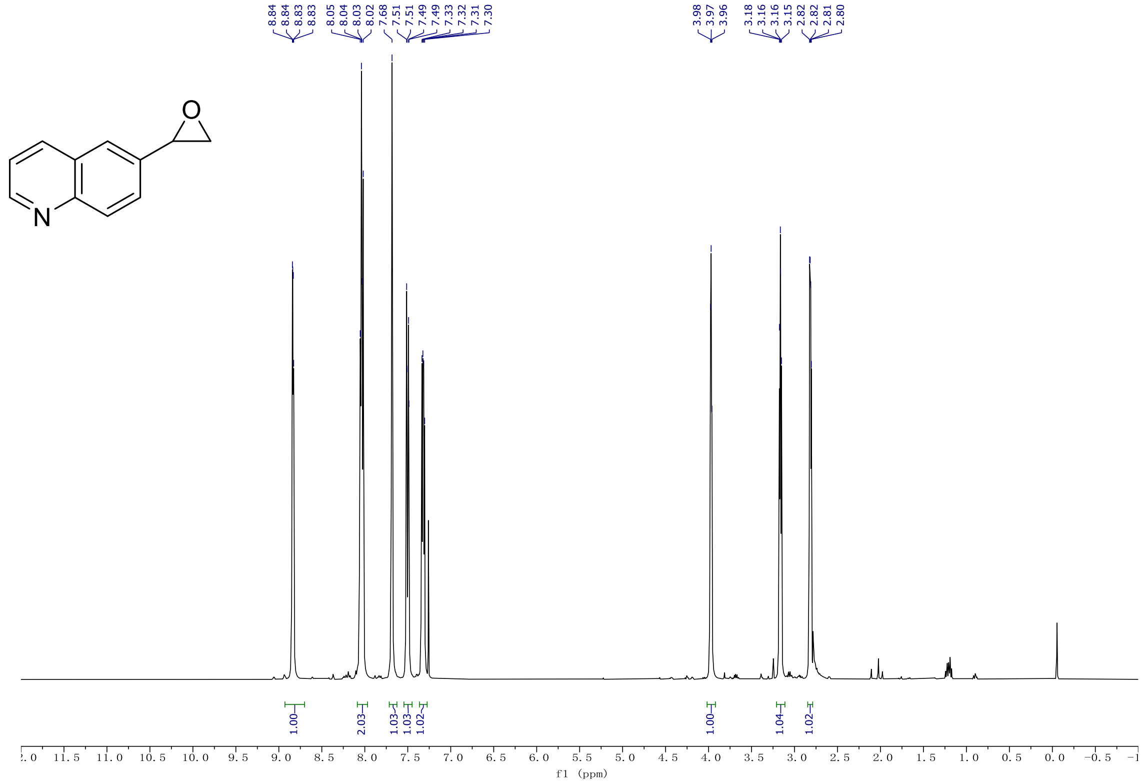

Supplementary Figure 29. ^{1}H NMR spectrum (400 MHz, $CDCl_3$, at rt) of **2ah**

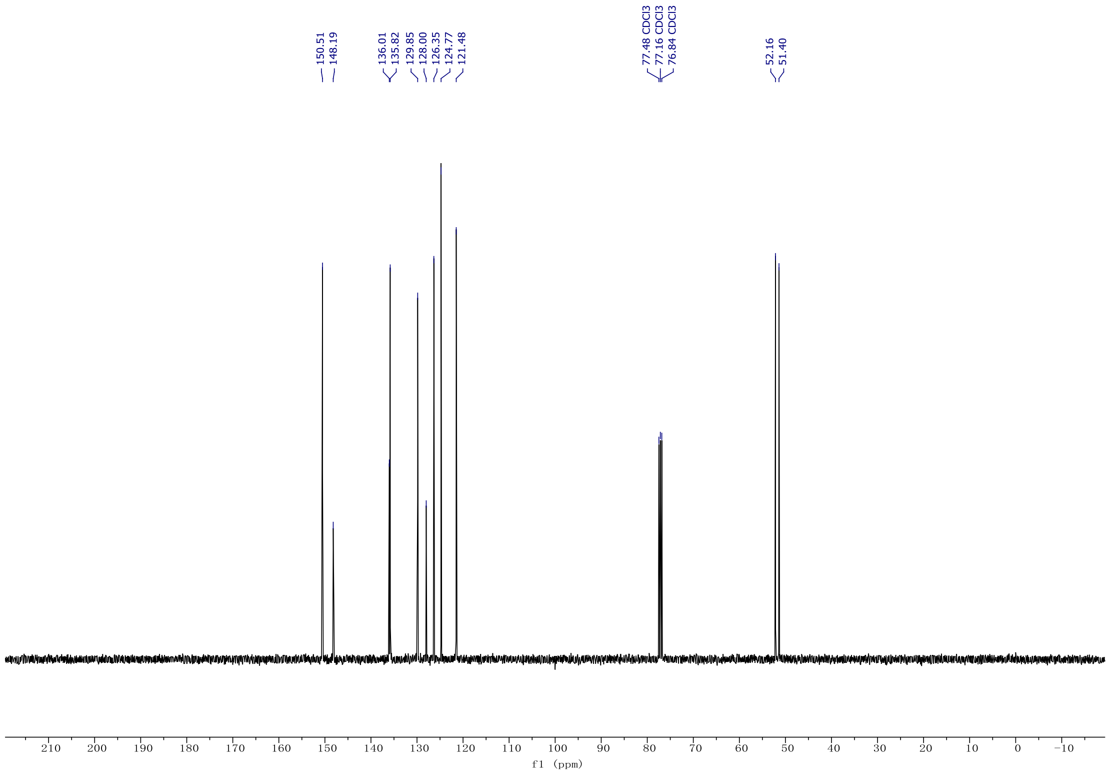

Supplementary Figure 30. ^{13}C NMR spectrum (101 MHz, $CDCl_3$, at rt) of **2ah**

2-(6-(3-(Adamantan-1-yl)-4-methoxyphenyl)naphthalen-1-yl)oxirane **2ap**

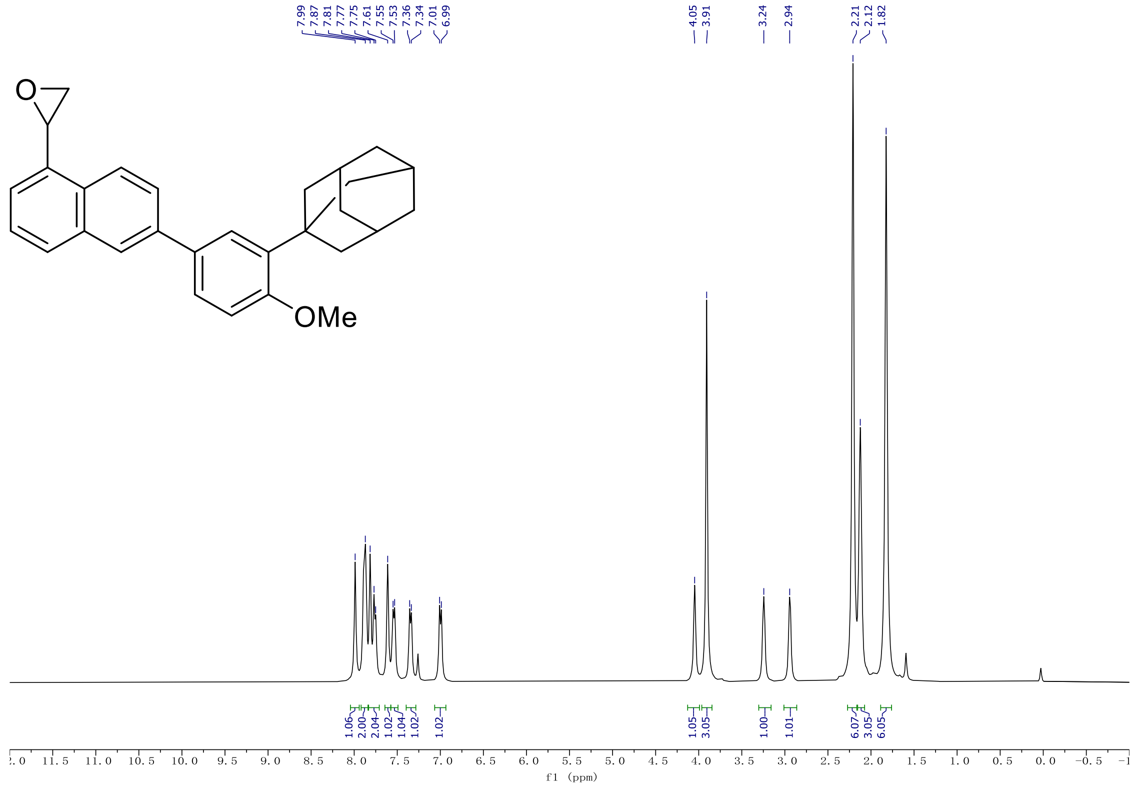

Supplementary Figure 31. ^{1}H NMR spectrum (400 MHz, $CDCl_3$, at rt) of **2ap**

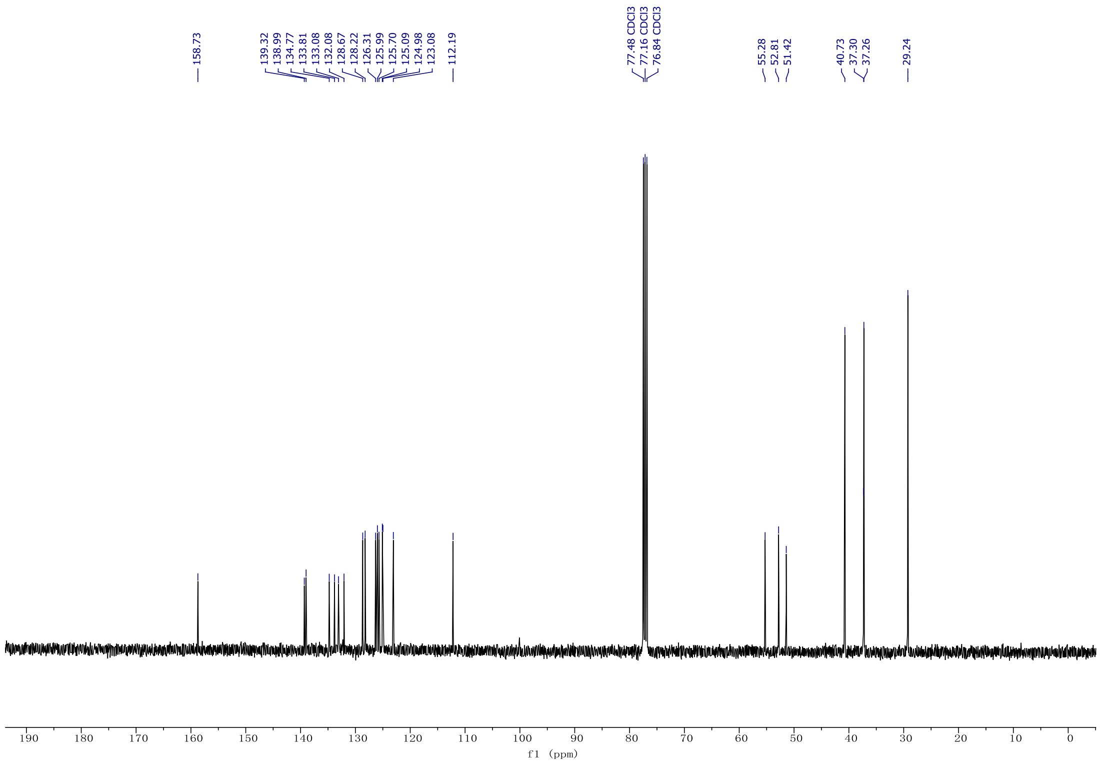

Supplementary Figure 32. ^{13}C NMR spectrum (101 MHz, $CDCl_3$, at rt) of **2ap**

From telmisartan **2aq**

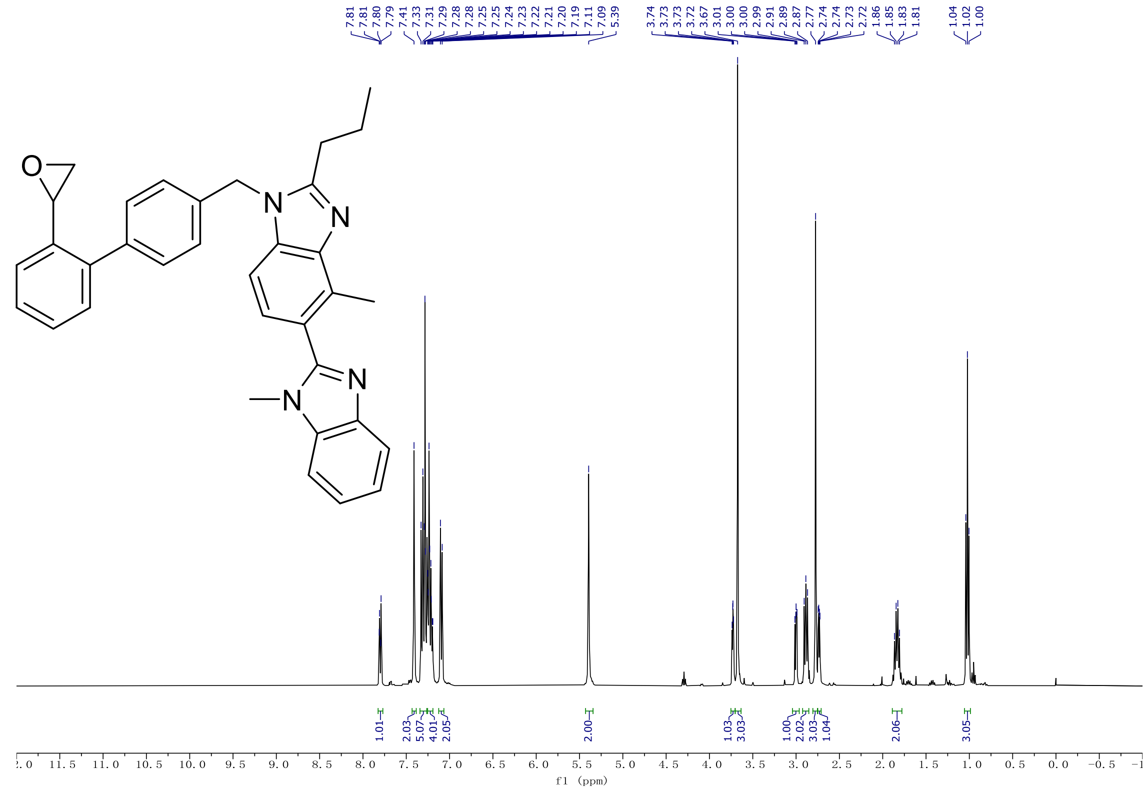

Supplementary Figure 33. ^{1}H NMR spectrum (400 MHz, $CDCl_3$, at rt) of **2aq**

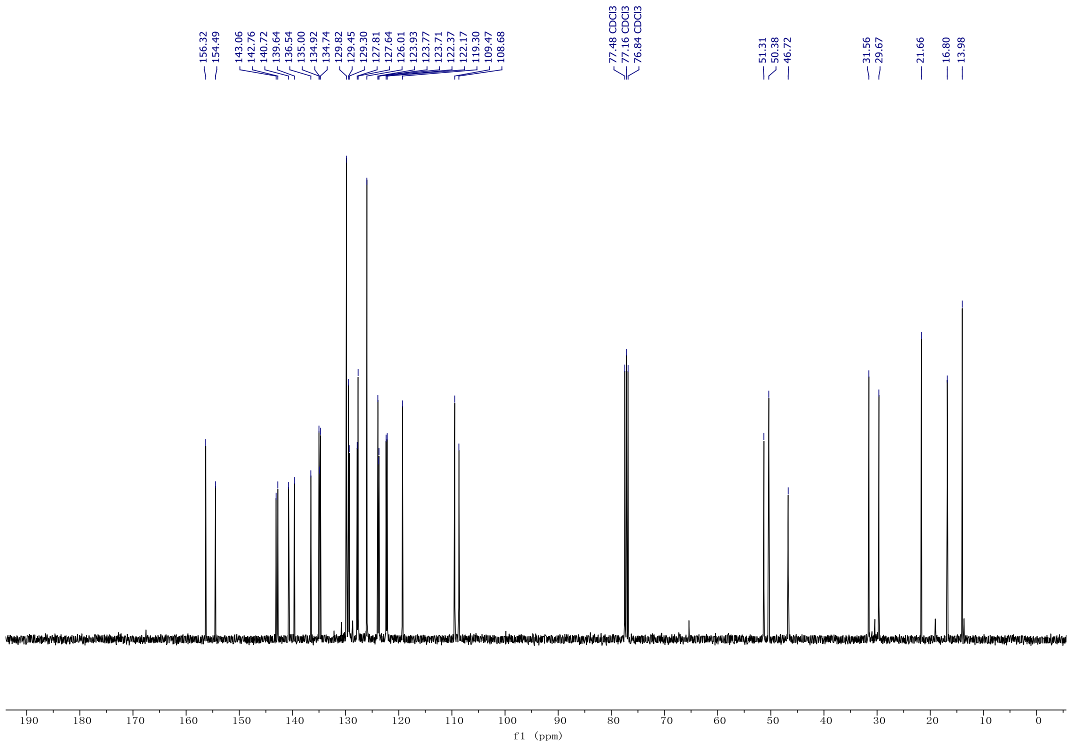

Supplementary Figure 34. ^{13}C NMR spectrum (101 MHz, $CDCl_3$, at rt) of **2aq**

2-([1,1'-Biphenyl]-4-yl)oxetane **4c**

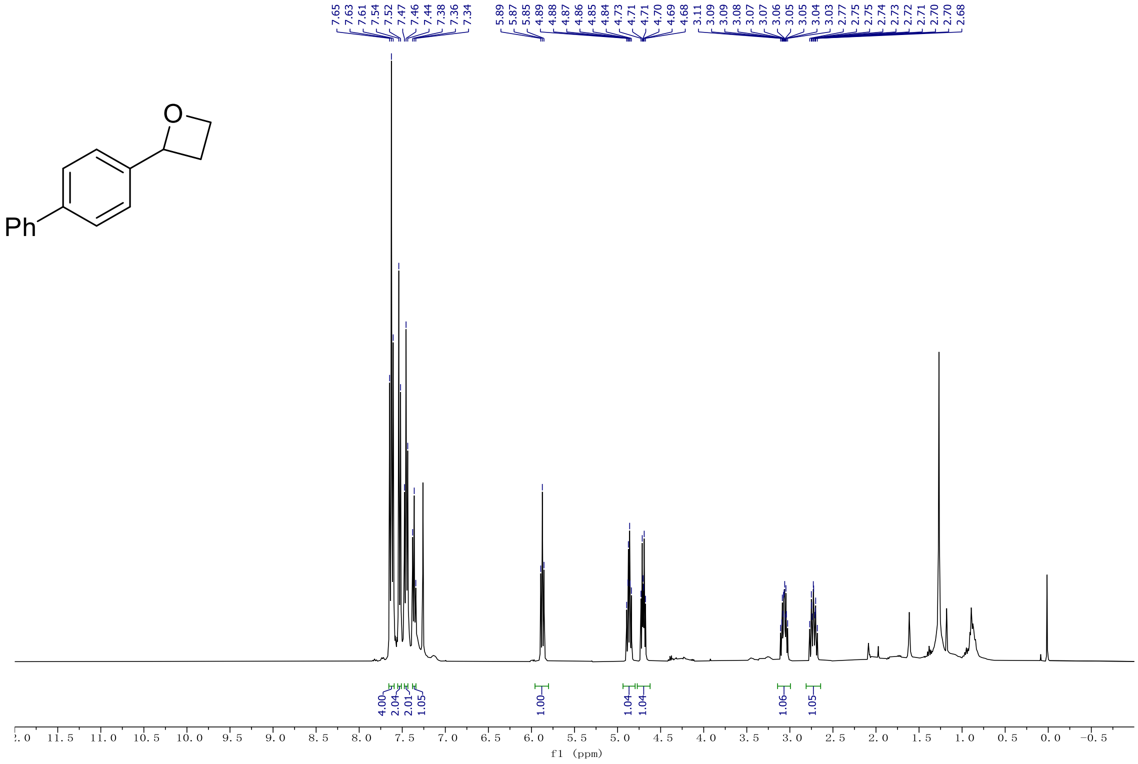

Supplementary Figure 35. ^{1}H NMR spectrum (400 MHz, $CDCl_3$, at rt) of **4c**

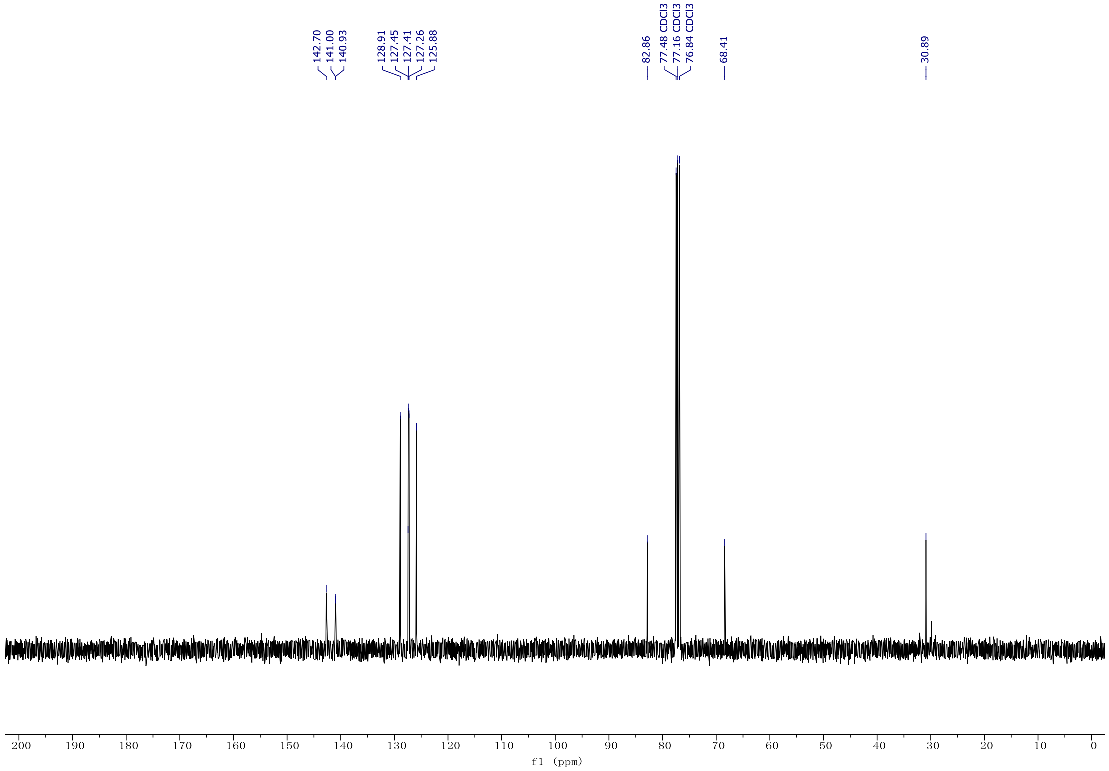

Supplementary Figure 36. ^{13}C NMR spectrum (101 MHz, $CDCl_3$, at rt) of **4c**

2-Phenyl-2-(3-(pyridin-2-yl)phenyl)ethan-1-ol **3a**

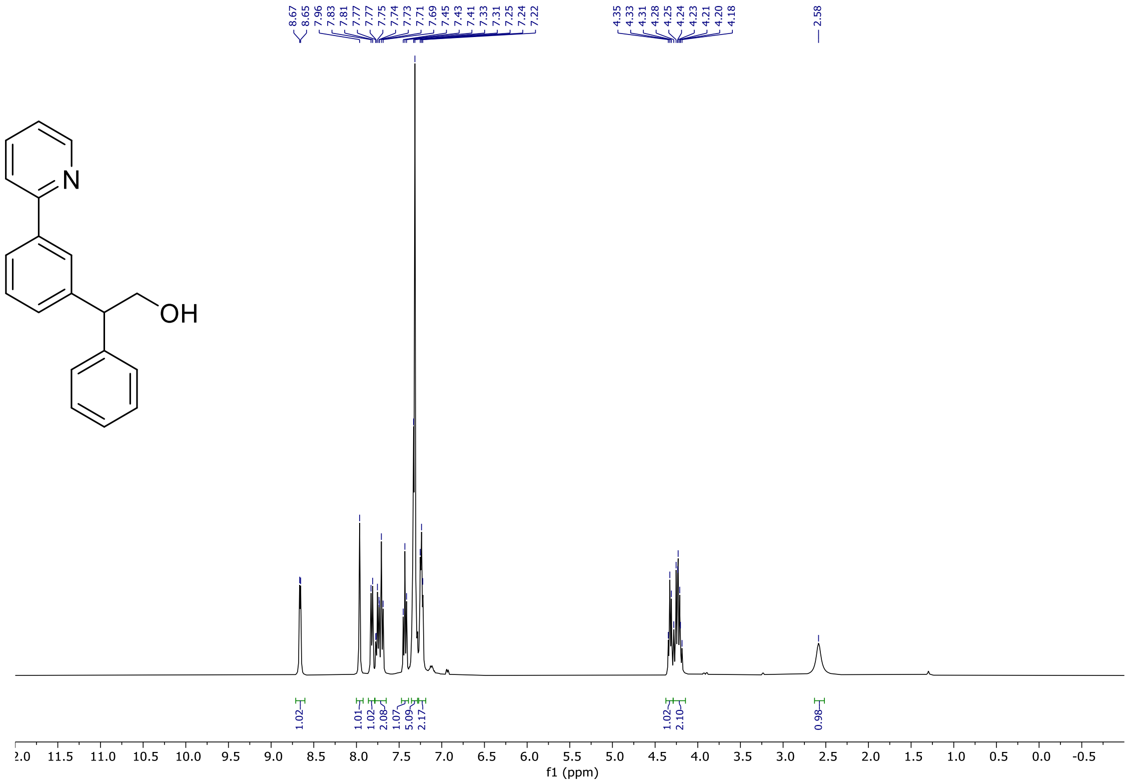

Supplementary Figure 37. ^{1}H NMR spectrum (400 MHz, $CDCl_3$, at rt) of **3a**

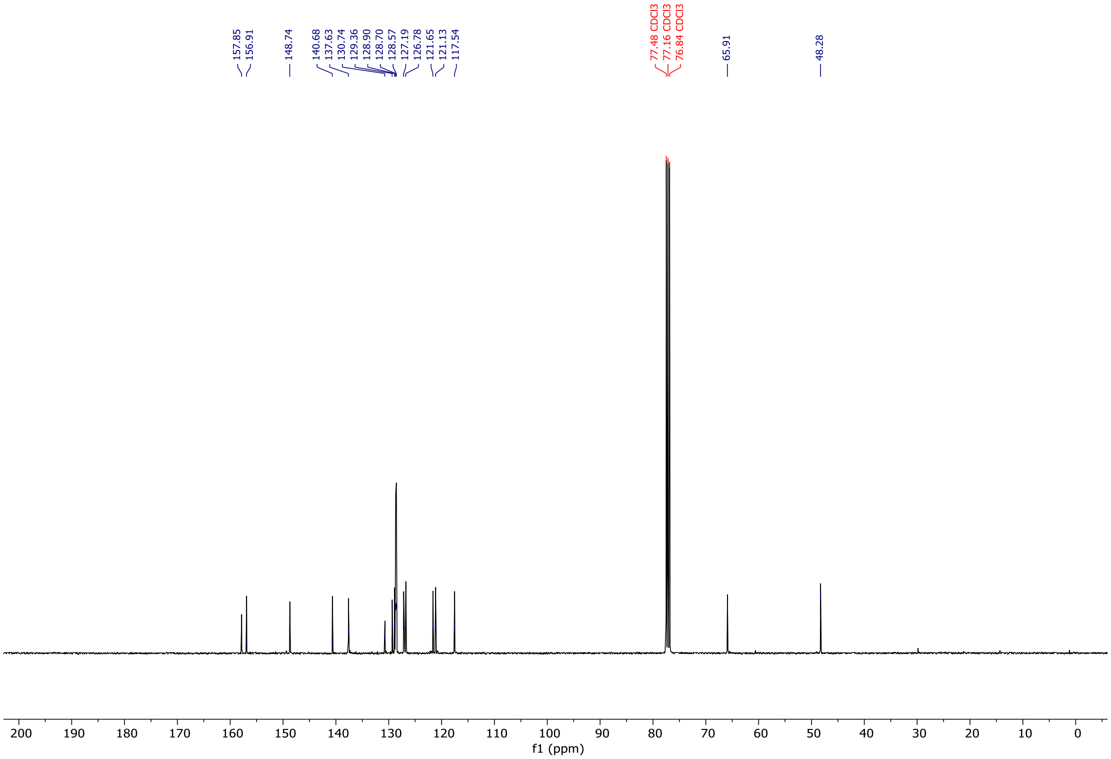

Supplementary Figure 38. ^{13}C NMR spectrum (101 MHz, $CDCl_3$, at rt) of **3a**

2-phenyl-2-(2-(pyridin-2-yl)phenyl)ethan-1-ol **3a’**

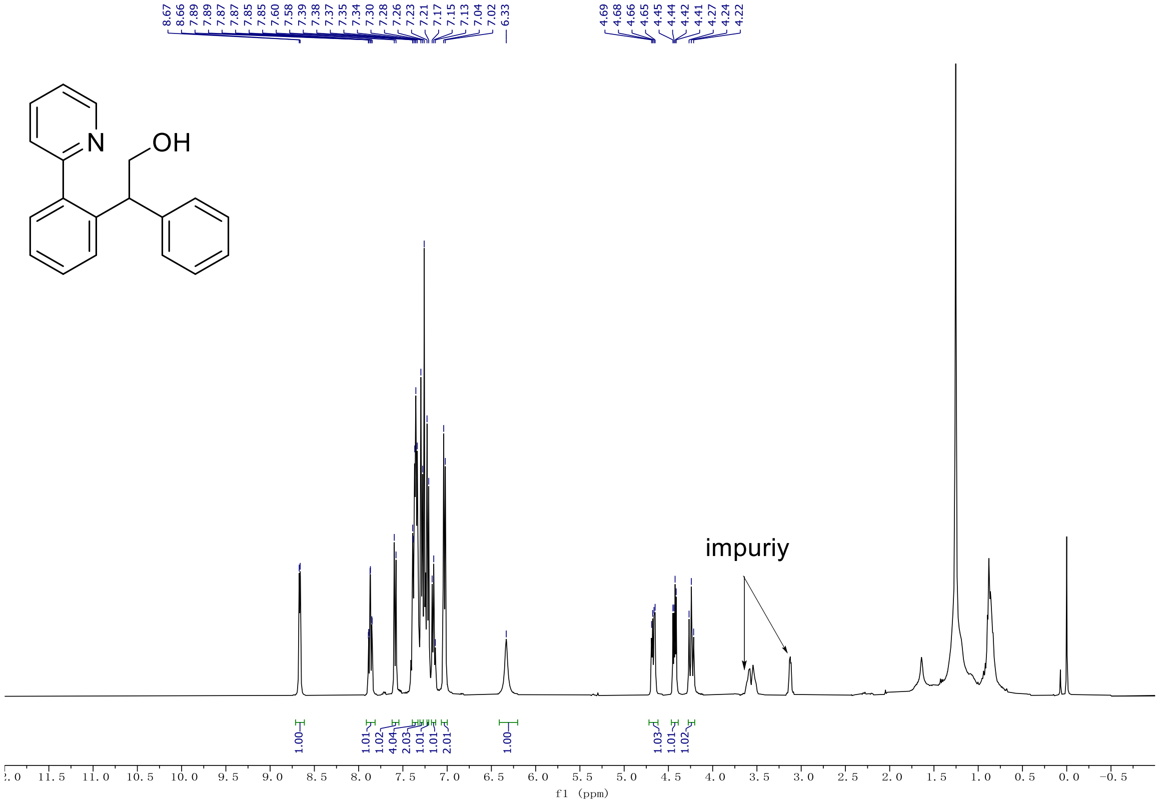

Supplementary Figure 39. ^{1}H NMR spectrum (400 MHz, $CDCl_3$, at rt) of **3a’**

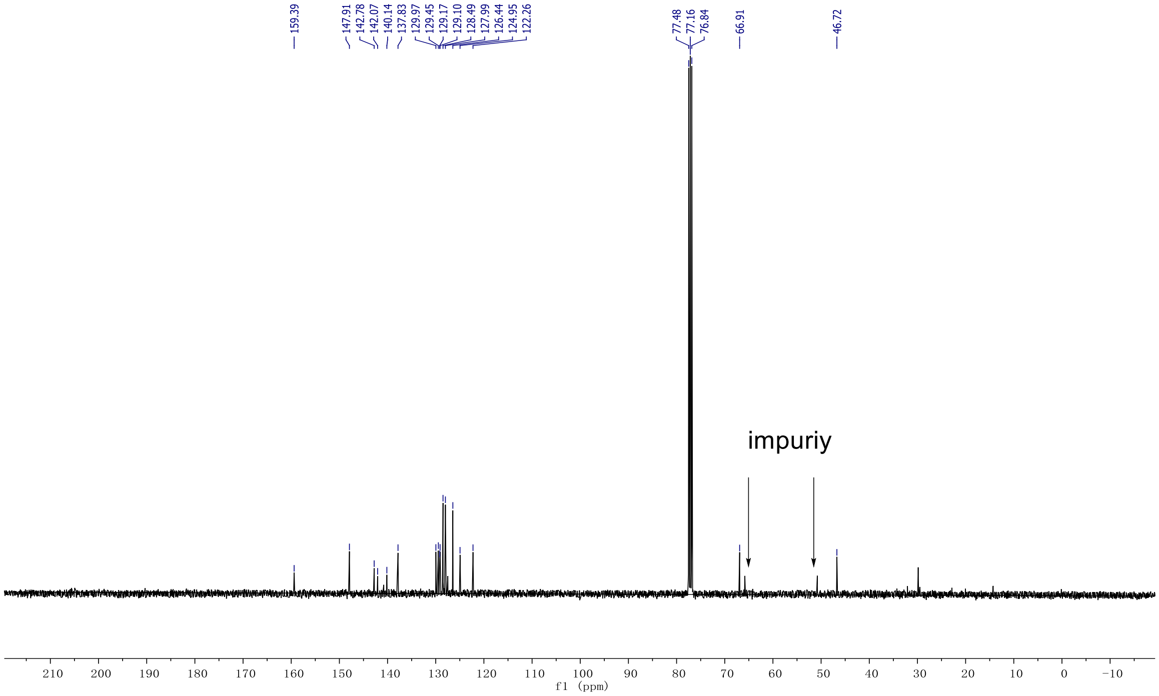

Supplementary Figure 40. ^{13}C NMR spectrum (101 MHz, $CDCl_3$, at rt) of **3a’**

2-(2-Hydroxy-1-phenylethyl)-4-(pyridin-2-yl)phenol **3b**

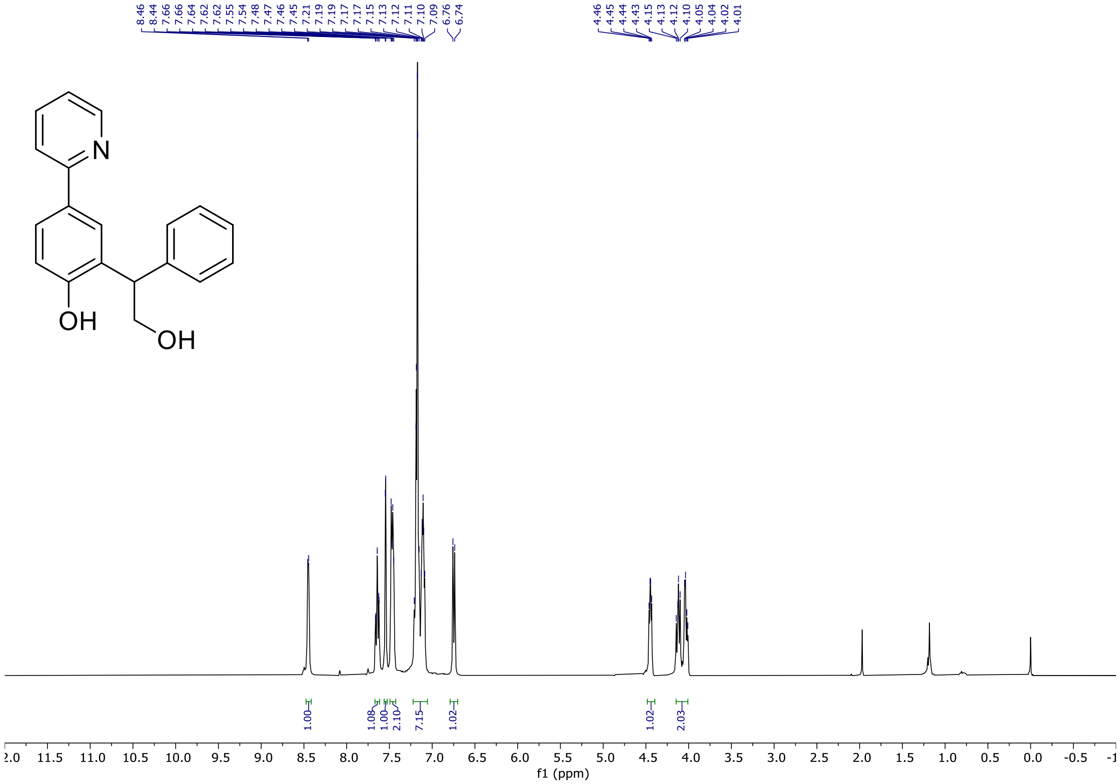

Supplementary Figure 41. ^{1}H NMR spectrum (400 MHz, $CDCl_3$, at rt) of **3b**

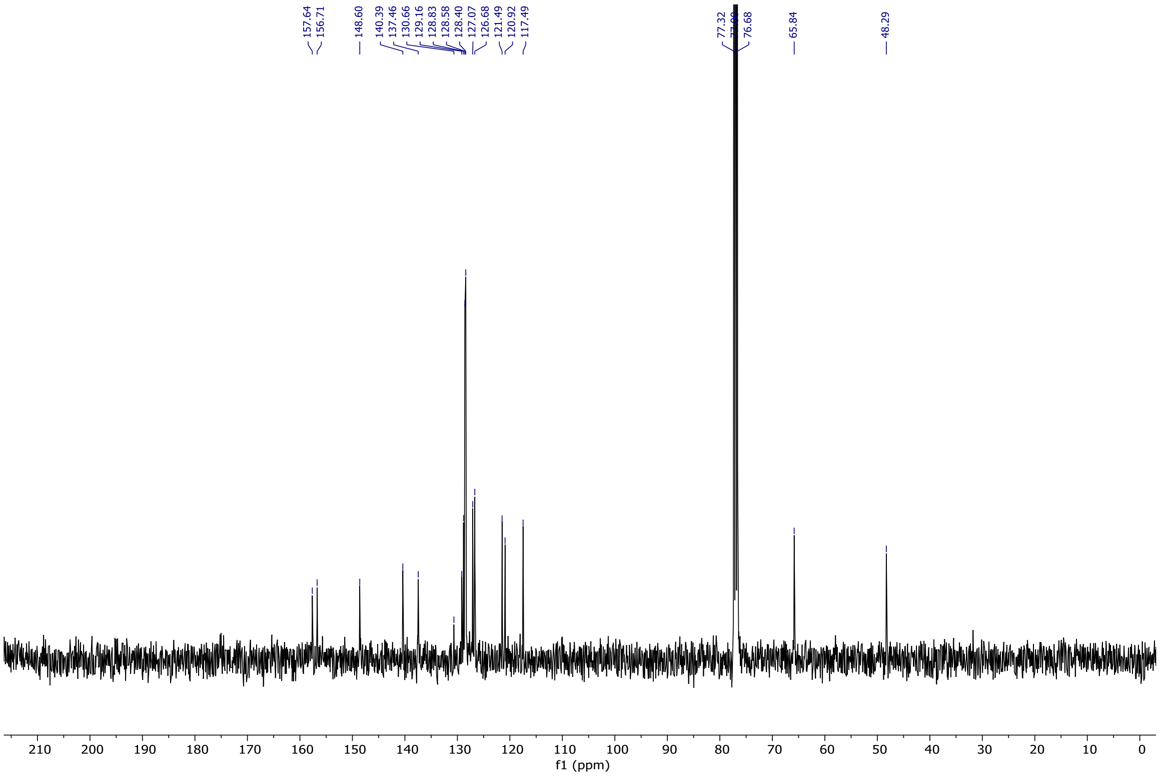

Supplementary Figure 42. ^{13}C NMR spectrum (101 MHz, $CDCl_3$, at rt) of **3b**

2-(2-Methoxy-5-(pyridin-2-yl)phenyl)-2-phenylethan-1-ol **3c**

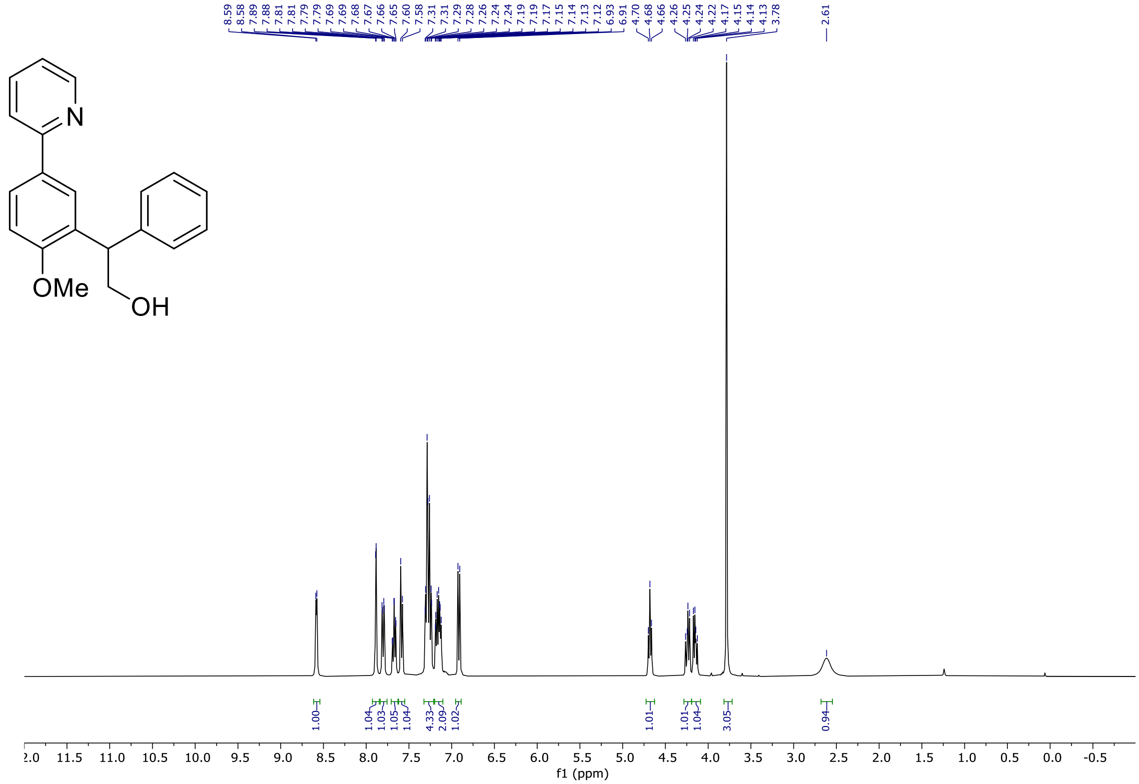

Supplementary Figure 43. ^{1}H NMR spectrum (400 MHz, $CDCl_3$, at rt) of **3c**

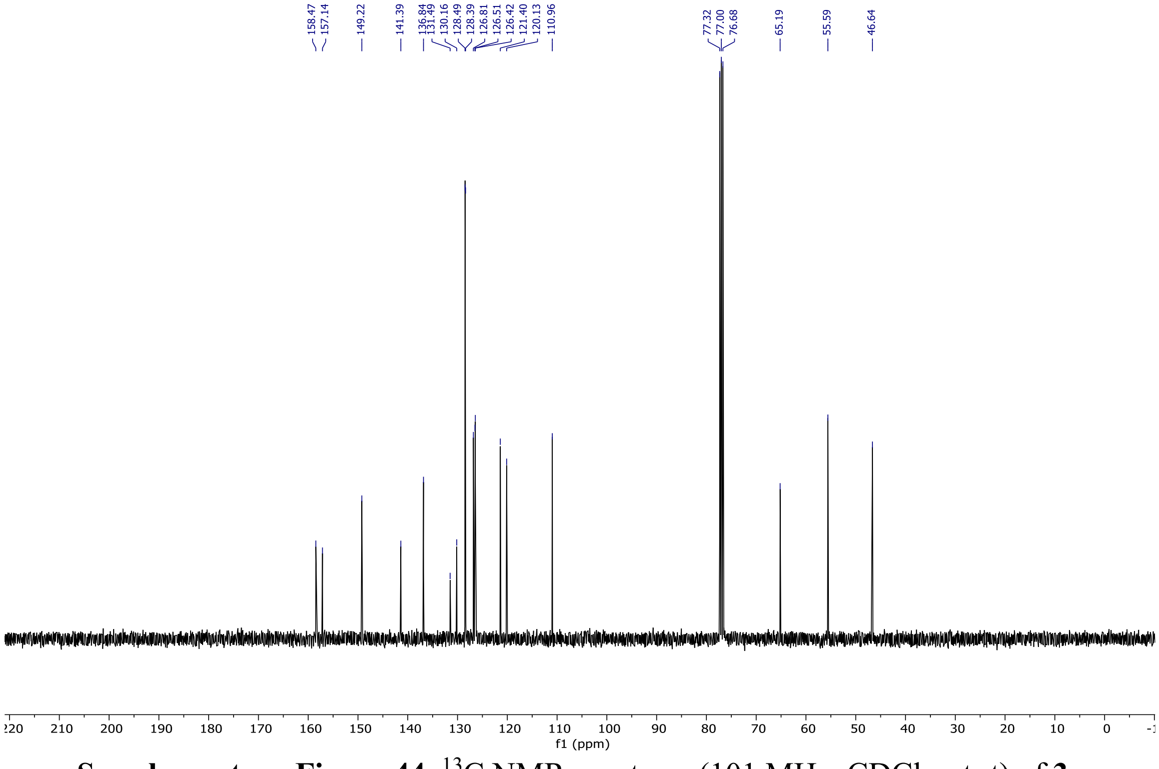

Supplementary Figure 44. ^{13}C NMR spectrum (101 MHz, $CDCl_3$, at rt) of **3c**

2-(2-(Methoxymethyl)-5-(pyridin-2-yl)phenyl)-2-phenylethan-1-ol **3d**

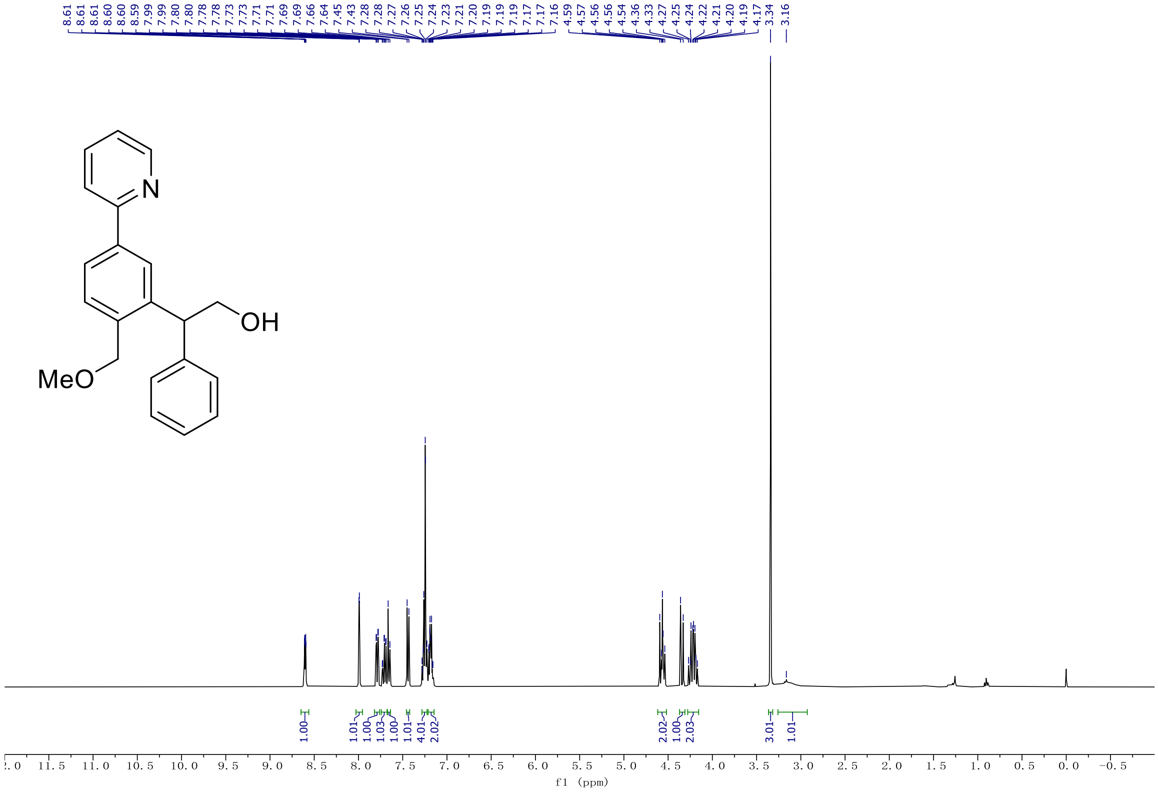

Supplementary Figure 45. ^{1}H NMR spectrum (400 MHz, $CDCl_3$, at rt) of **3d**

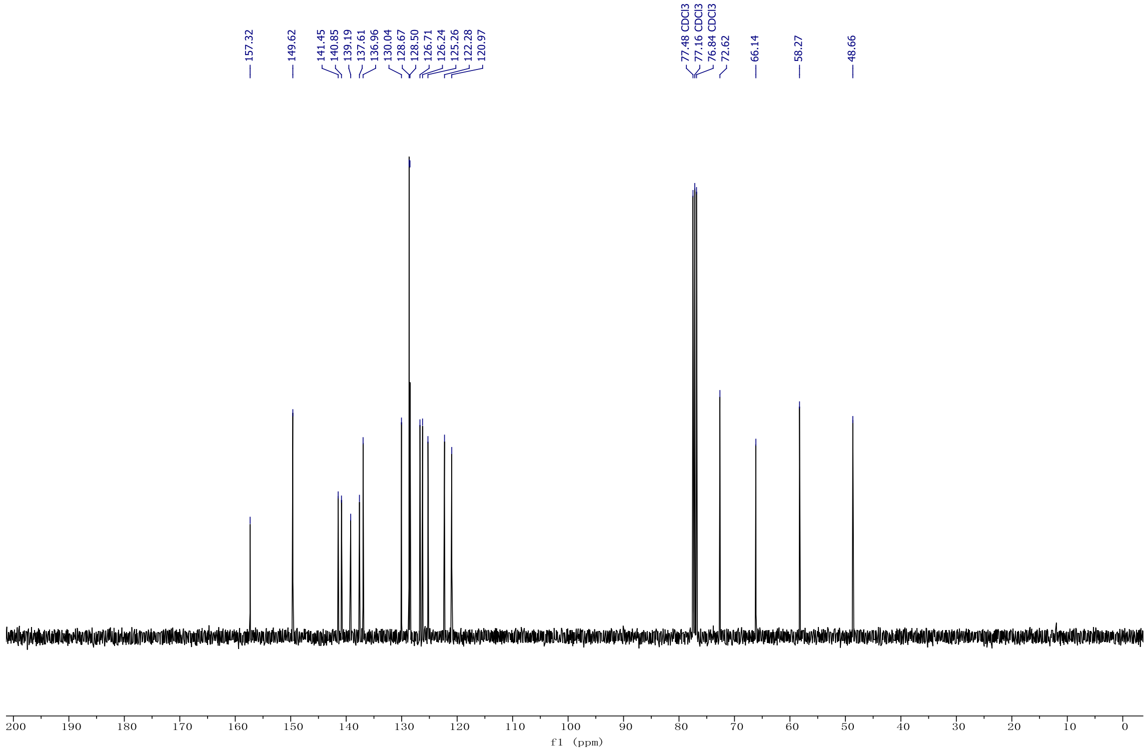

Supplementary Figure 46. ^{13}C NMR spectrum (101 MHz, $CDCl_3$, at rt) of **3d**

2-(2-Fluoro-5-(pyridin-2-yl)phenyl)-2-phenylethan-1-ol **3d**

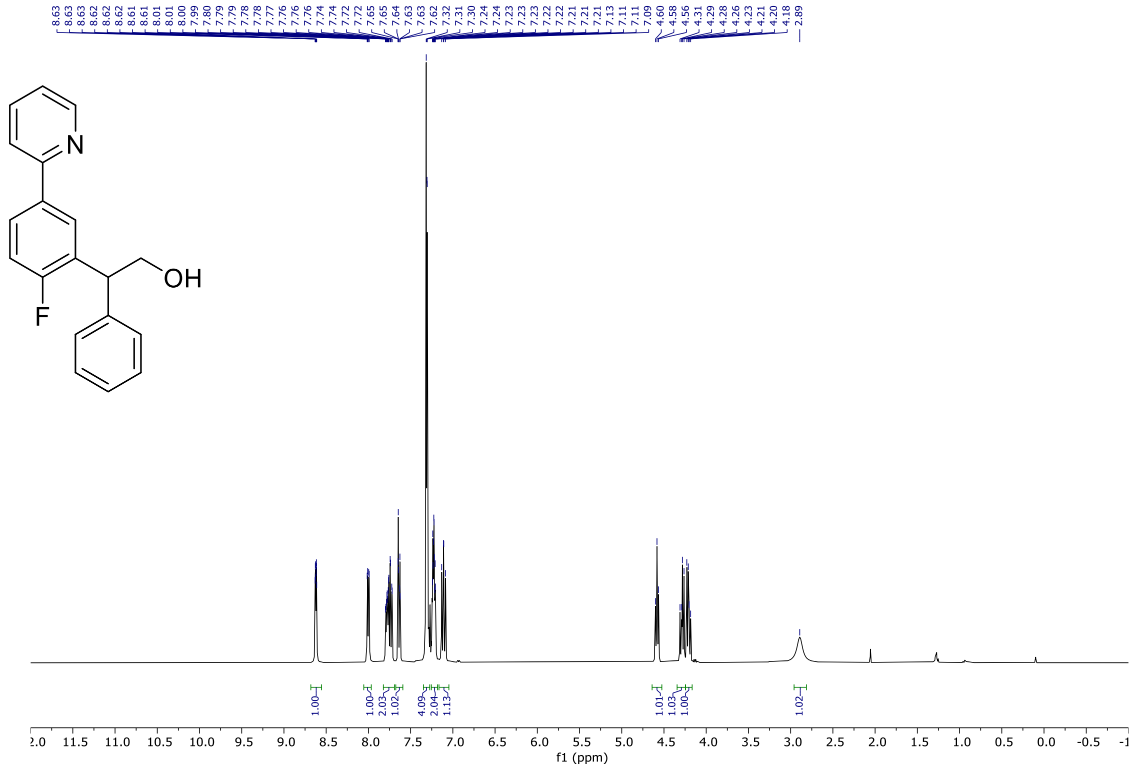

Supplementary Figure 47. ^{1}H NMR spectrum (400 MHz, $CDCl_3$, at rt) of **3d**

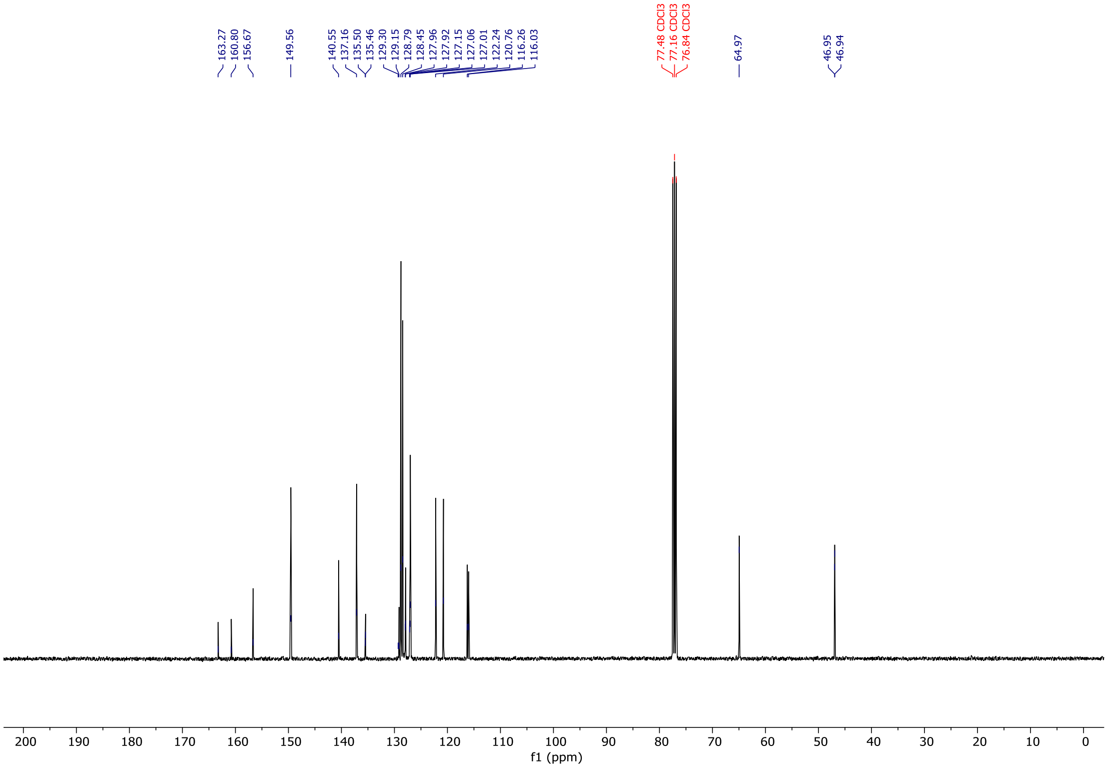

Supplementary Figure 48. ^{13}C NMR spectrum (101 MHz, $CDCl_3$, at rt) of **3d**

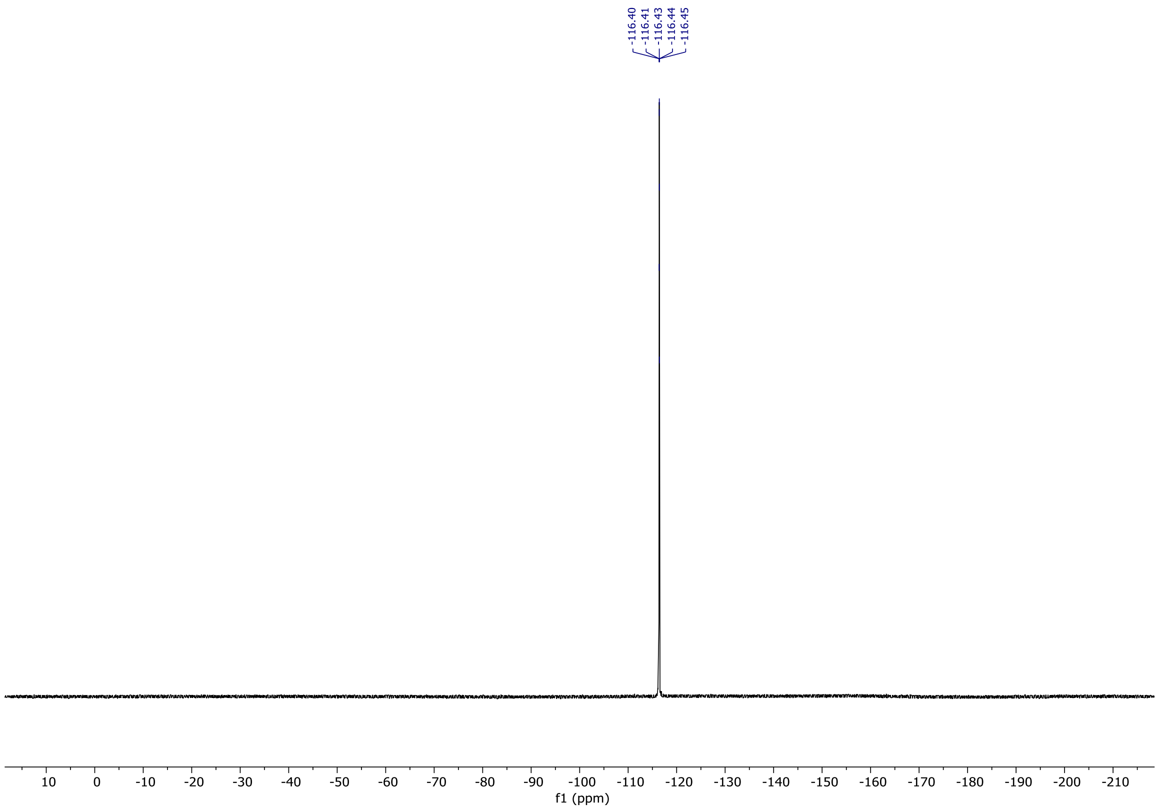

Supplementary Figure 49. ^{19}F NMR spectrum (376 MHz, $CDCl_3$, at rt) of **3d**

2-(2-Chloro-5-(pyridin-2-yl)phenyl)-2-phenylethan-1-ol **3f**

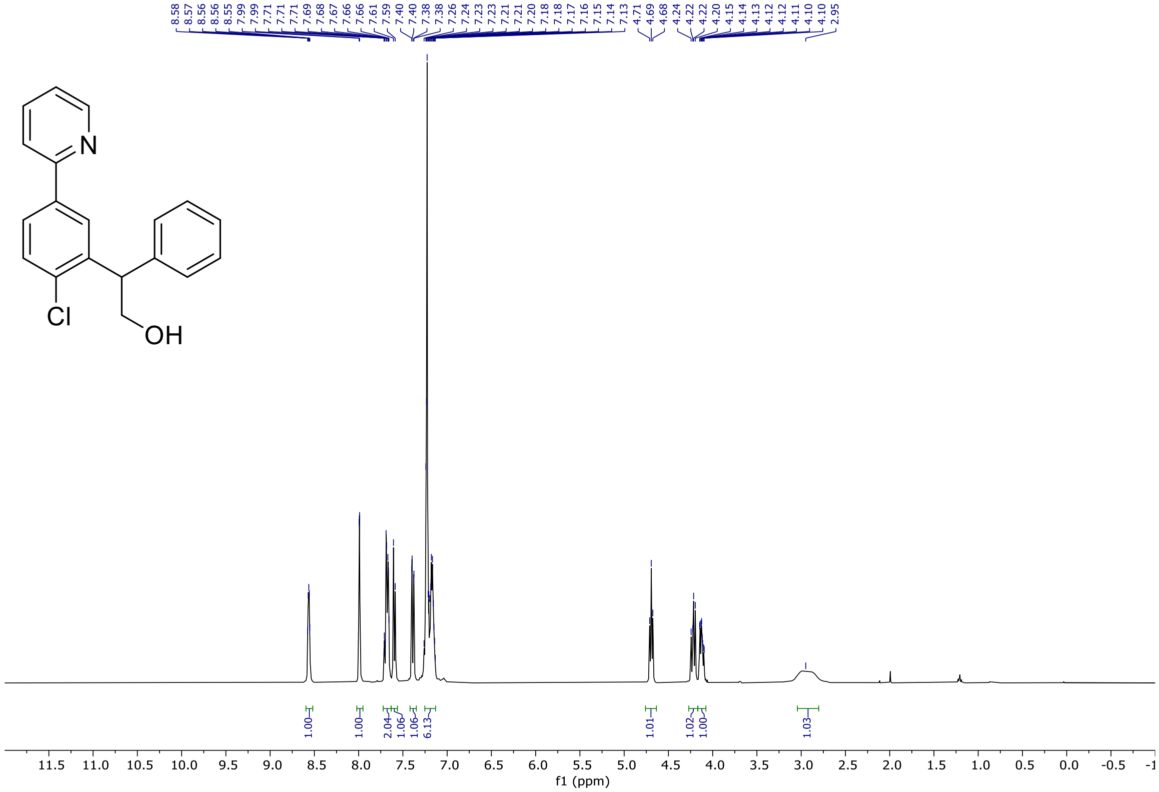

Supplementary Figure 50. ^{1}H NMR spectrum (400 MHz, $CDCl_3$, at rt) of **3f**

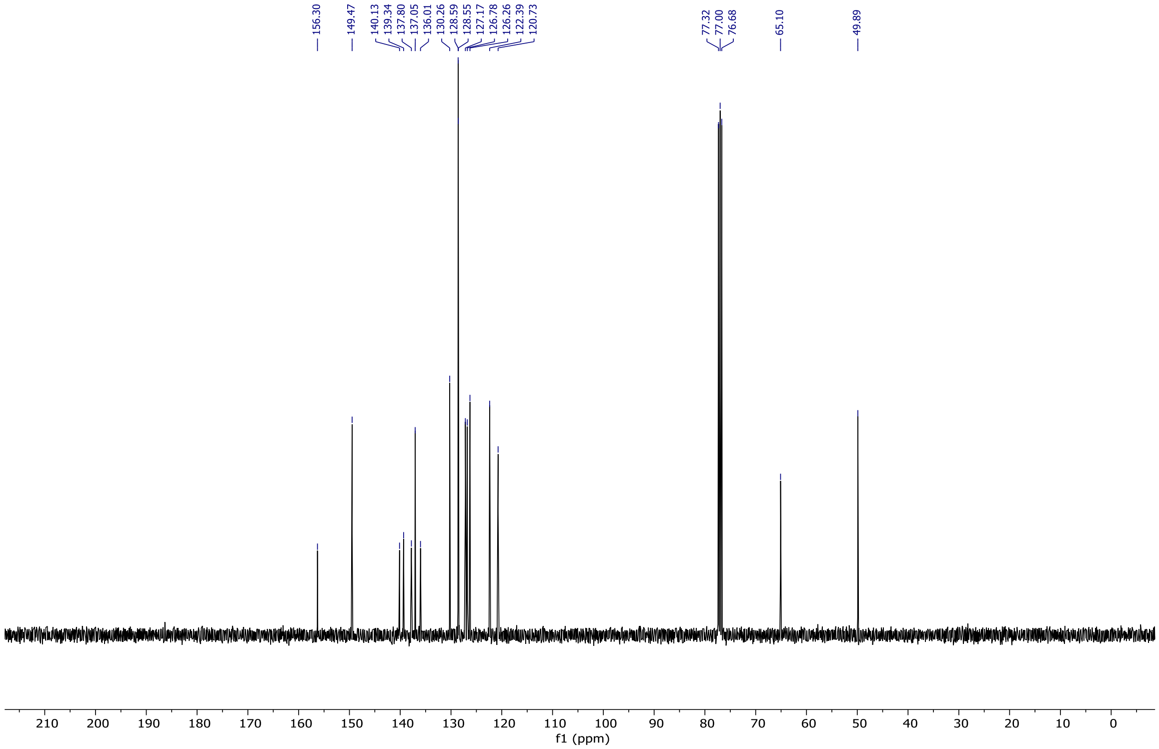

Supplementary Figure 51. ^{13}C NMR spectrum (101 MHz, $CDCl_3$, at rt) of **3f**

2-Phenyl-2-(5-(pyridin-2-yl)-2-(trifluoromethyl)phenyl)ethan-1-ol **3g**

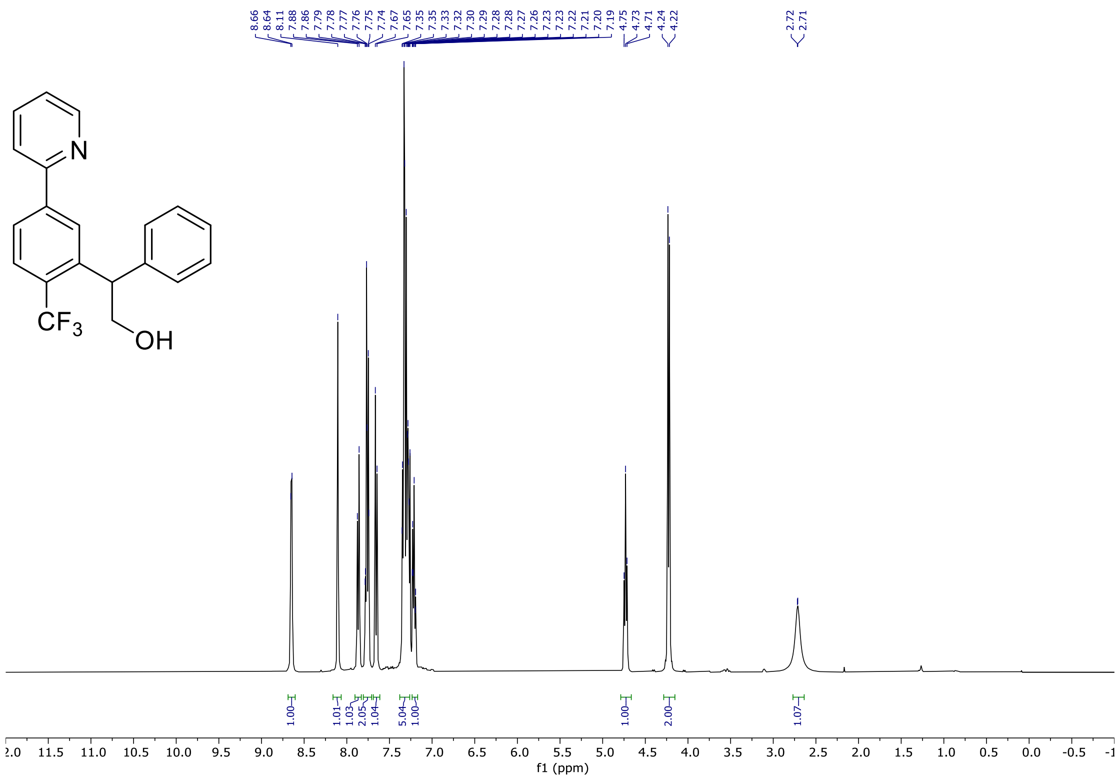

Supplementary Figure 52. ^{1}H NMR spectrum (400 MHz, $CDCl_3$, at rt) of **3g**

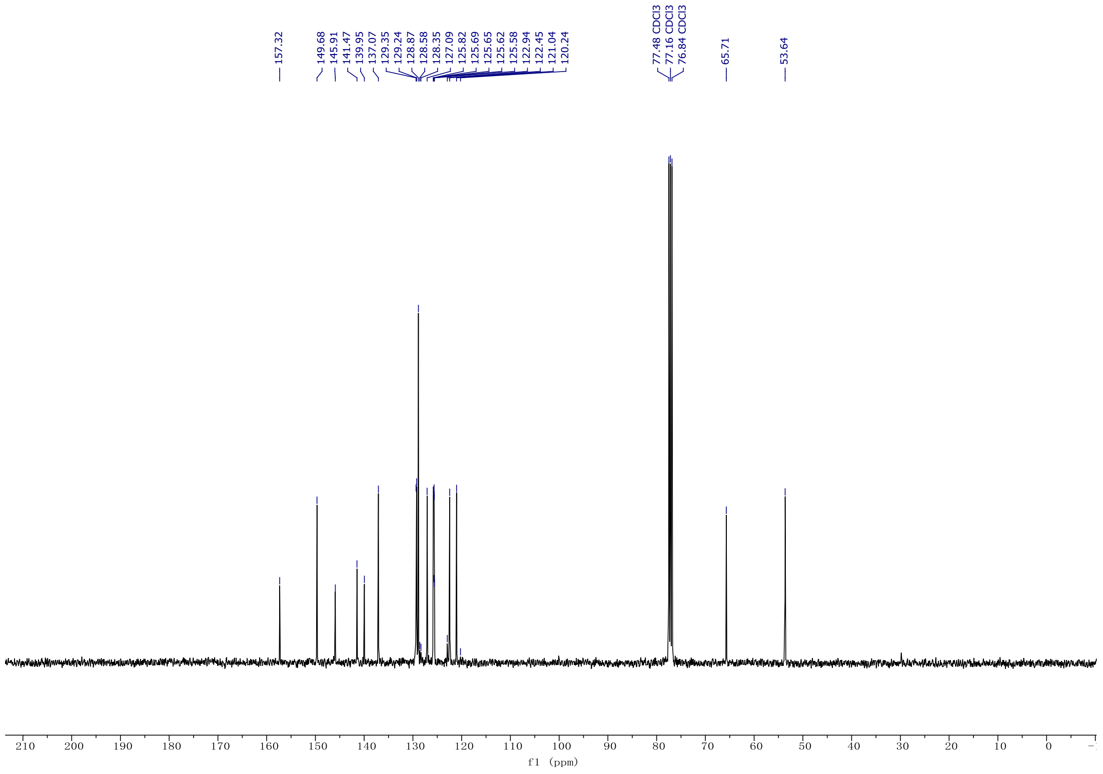

Supplementary Figure 53. ^{13}C NMR spectrum (101 MHz, $CDCl_3$, at rt) of **3g**

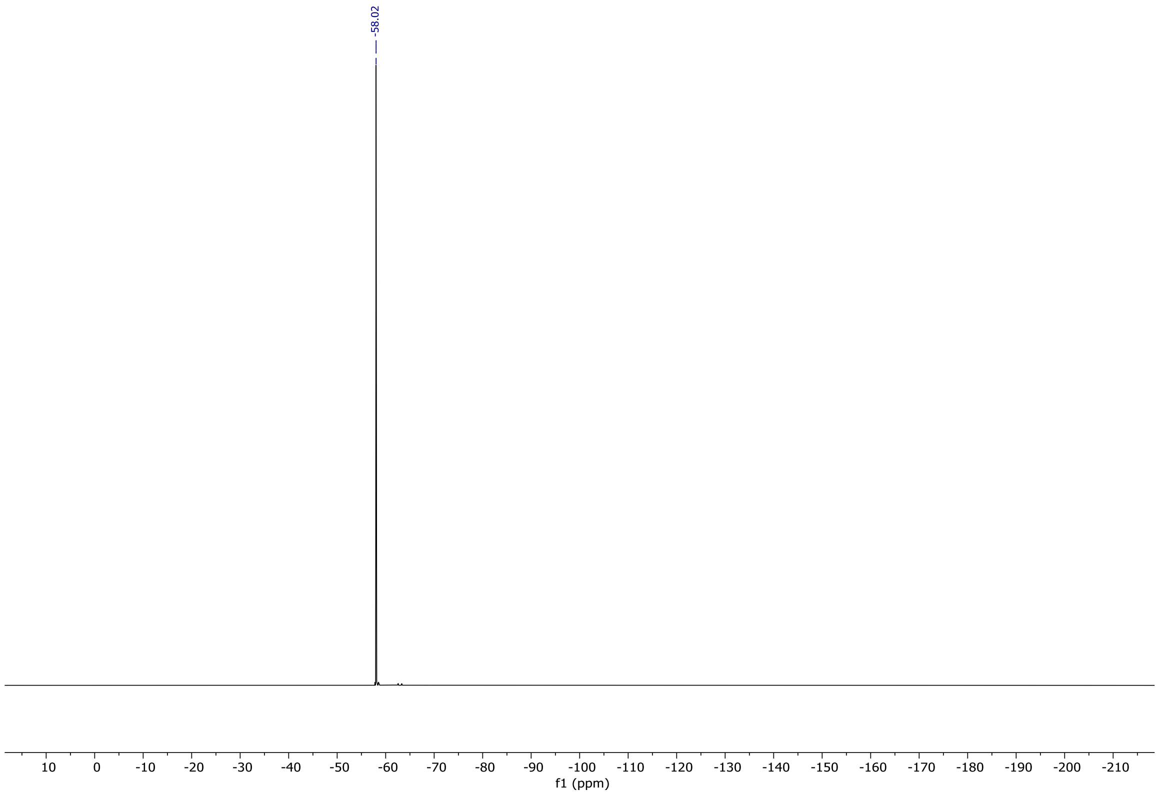

Supplementary Figure 54. ^{19}F NMR spectrum (376 MHz, $CDCl_3$, at rt) of **3g**

Methyl 3-(2-(2-hydroxy-1-phenylethyl)-4-(pyridin-2-yl)phenyl)propanoate **3h**

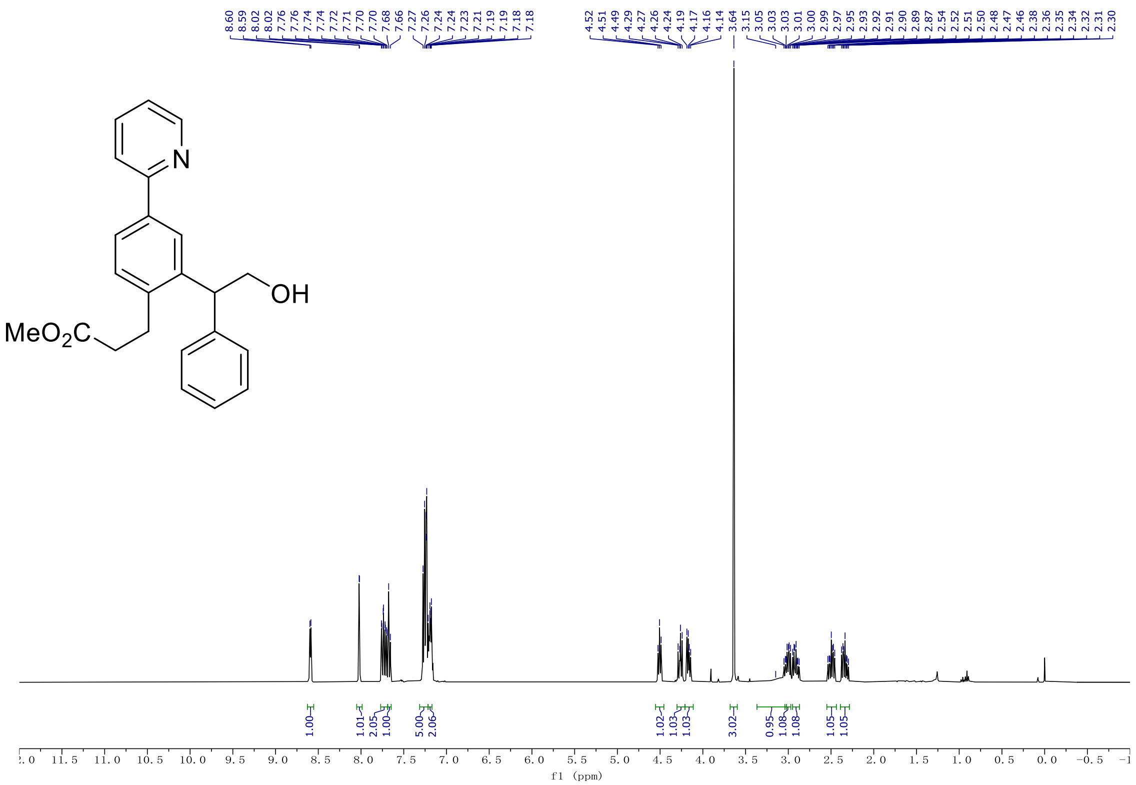

Supplementary Figure 55. ^{1}H NMR spectrum (400 MHz, $CDCl_3$, at rt) of **3h**

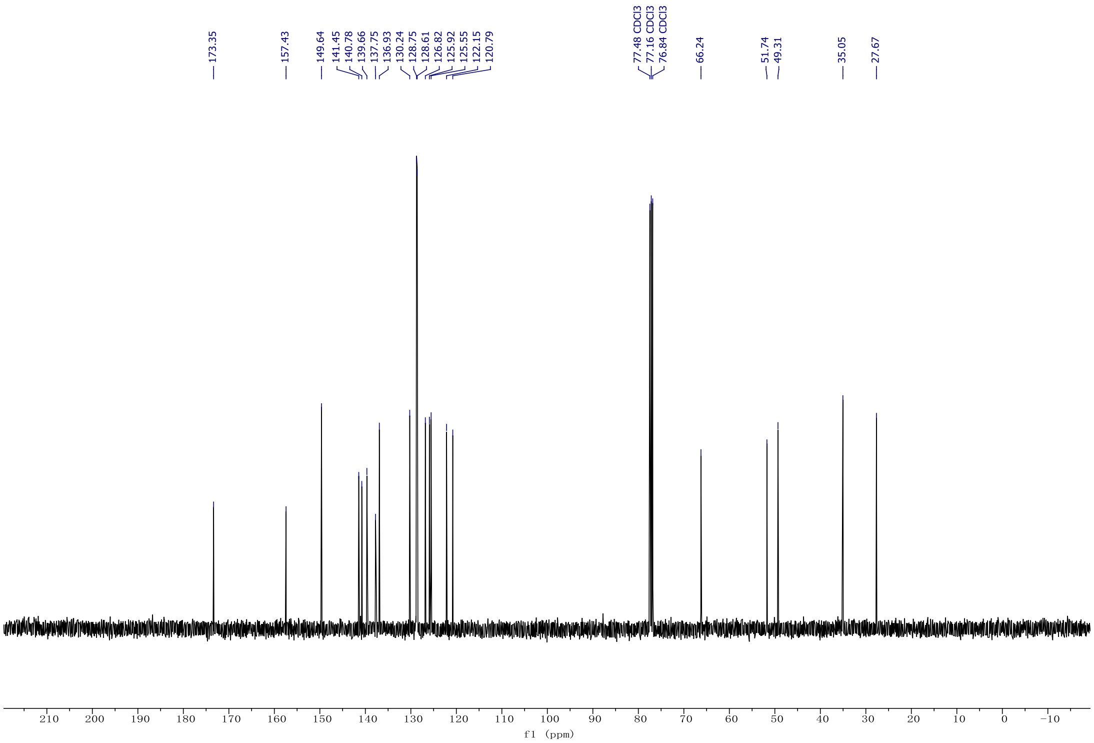

Supplementary Figure 56. ^{13}C NMR spectrum (101 MHz, $CDCl_3$, at rt) of **3h**

2-Phenyl-2-(2-(phenylethynyl)-5-(pyridin-2-yl)phenyl)ethan-1-ol **3i**

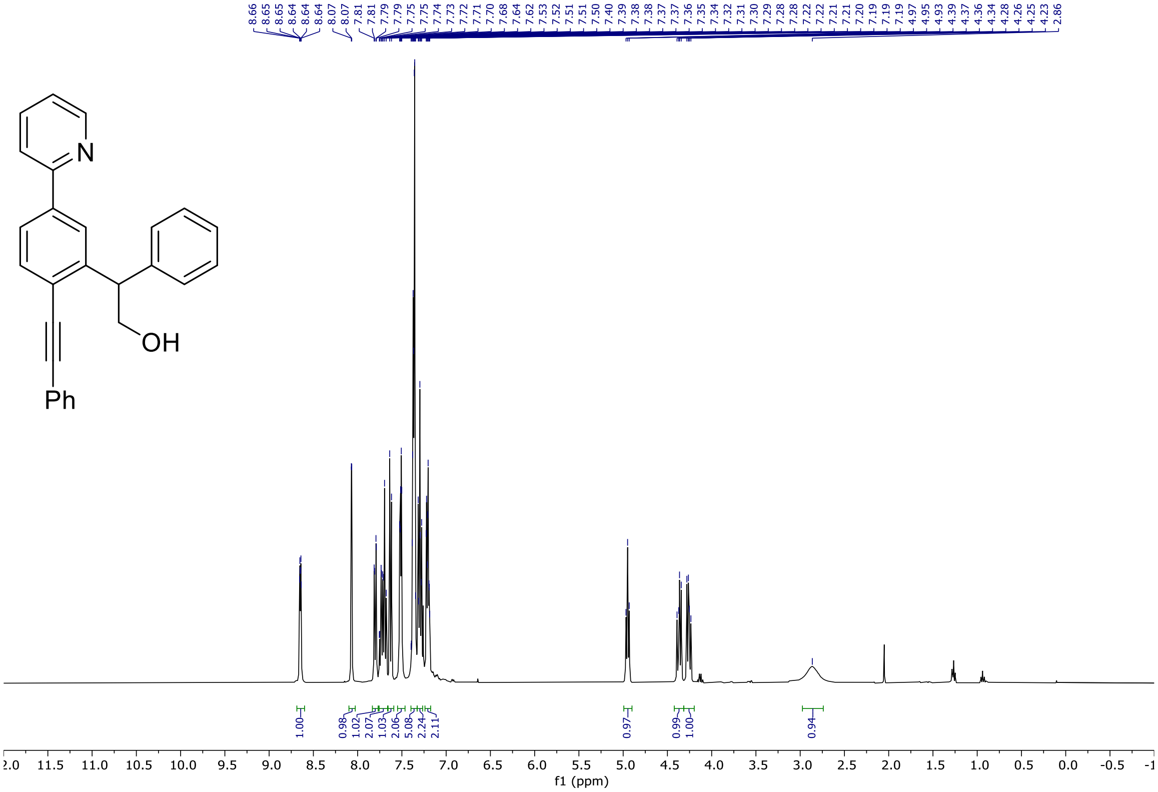

Supplementary Figure 57. ^{1}H NMR spectrum (400 MHz, $CDCl_3$, at rt) of **3i**

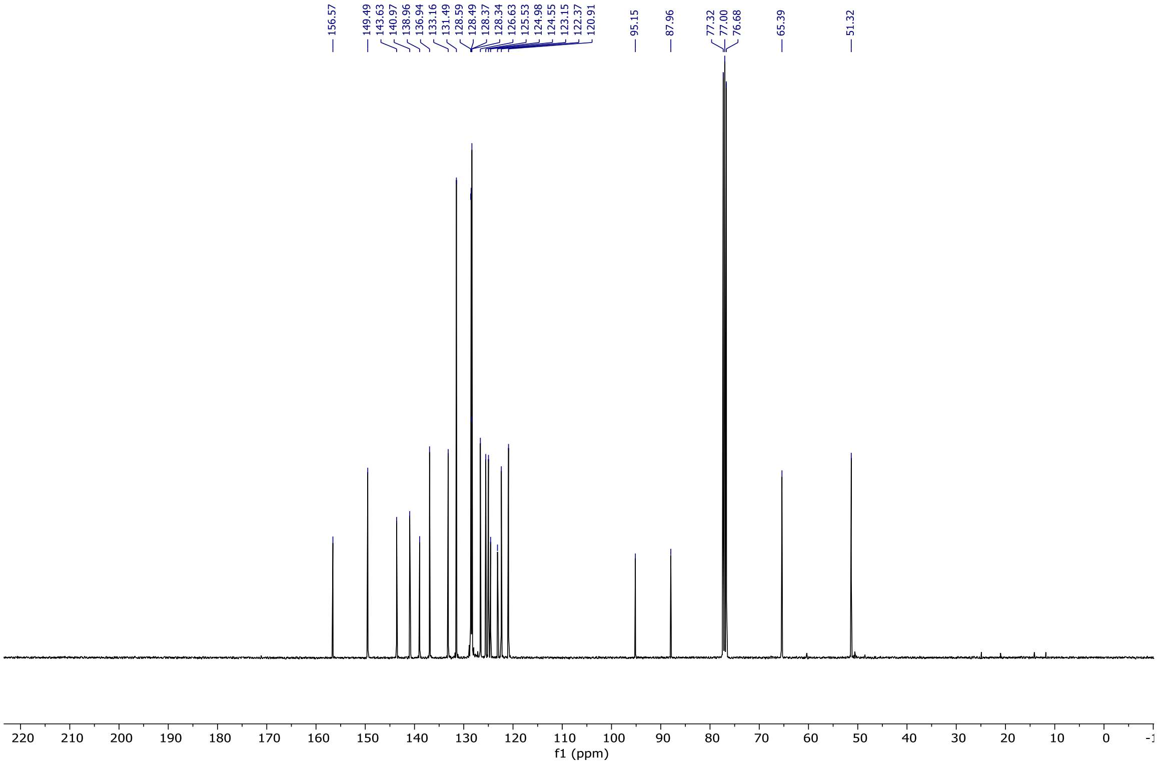

Supplementary Figure 58. ^{13}C NMR spectrum (101 MHz, $CDCl_3$, at rt) of **3i**

2-(2-Fluoro-3-(pyridin-2-yl)phenyl)-2-phenylethan-1-ol **3j**

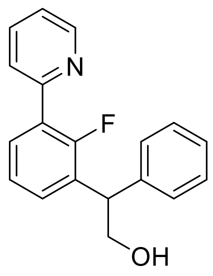

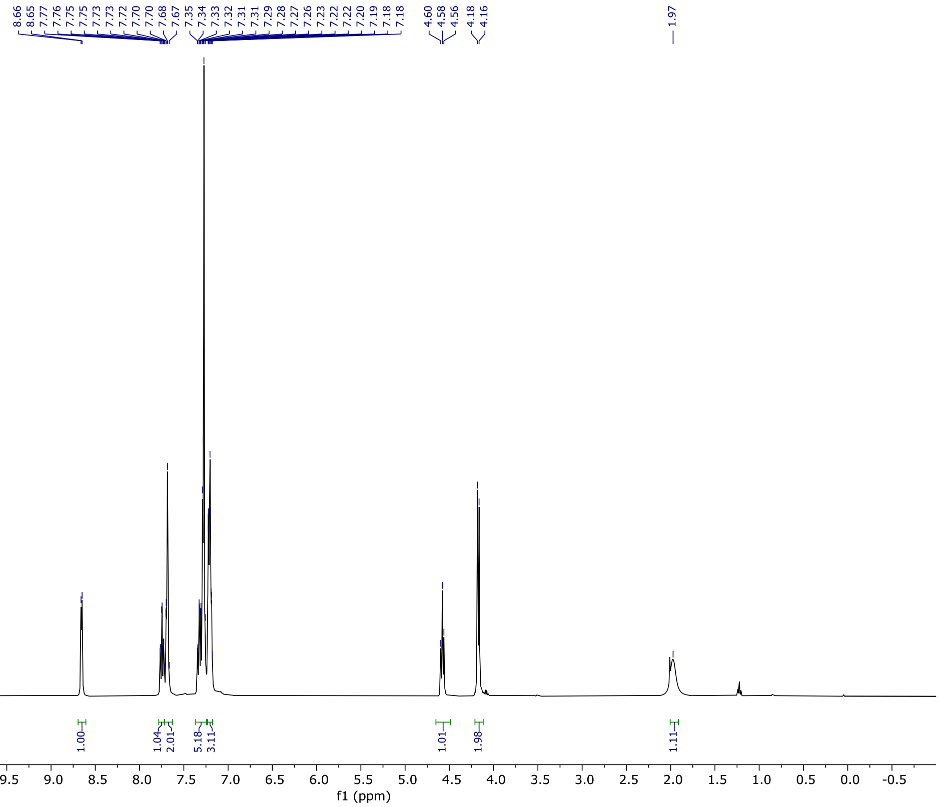

Supplementary Figure 59. ^{1}H NMR spectrum (400 MHz, $CDCl_3$, at rt) of **3j**

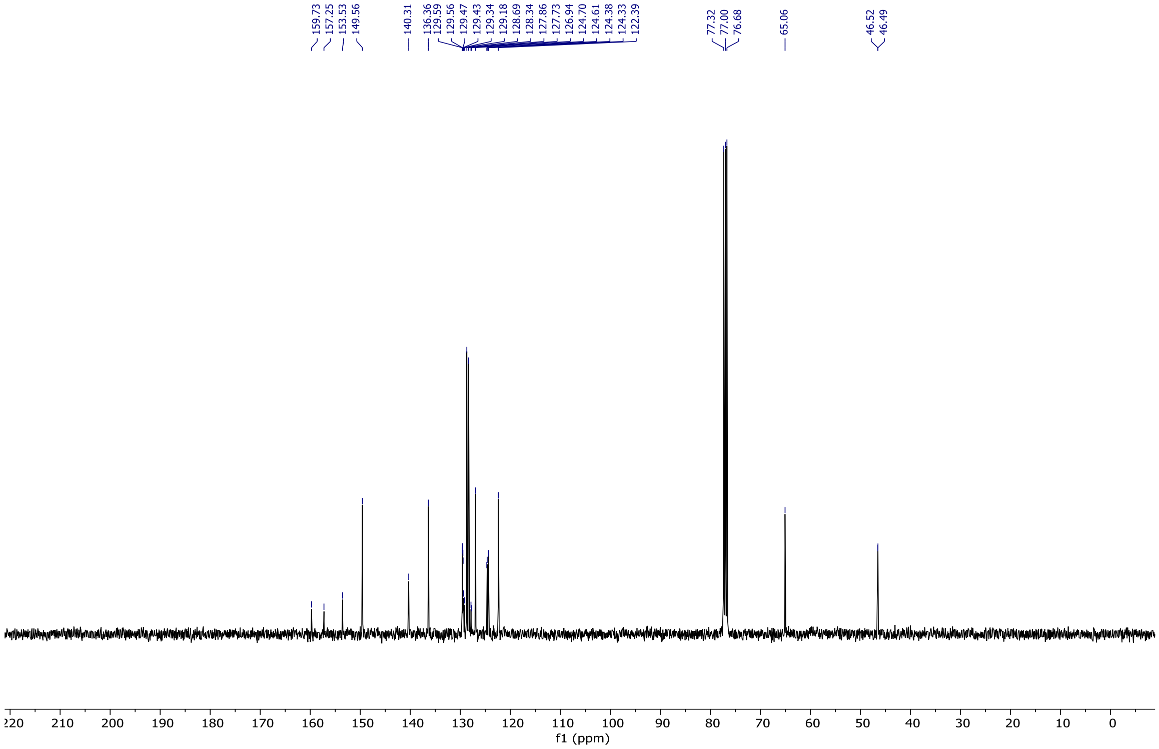

Supplementary Figure 60. ^{13}C NMR spectrum (101 MHz, $CDCl_3$, at rt) of **3j**

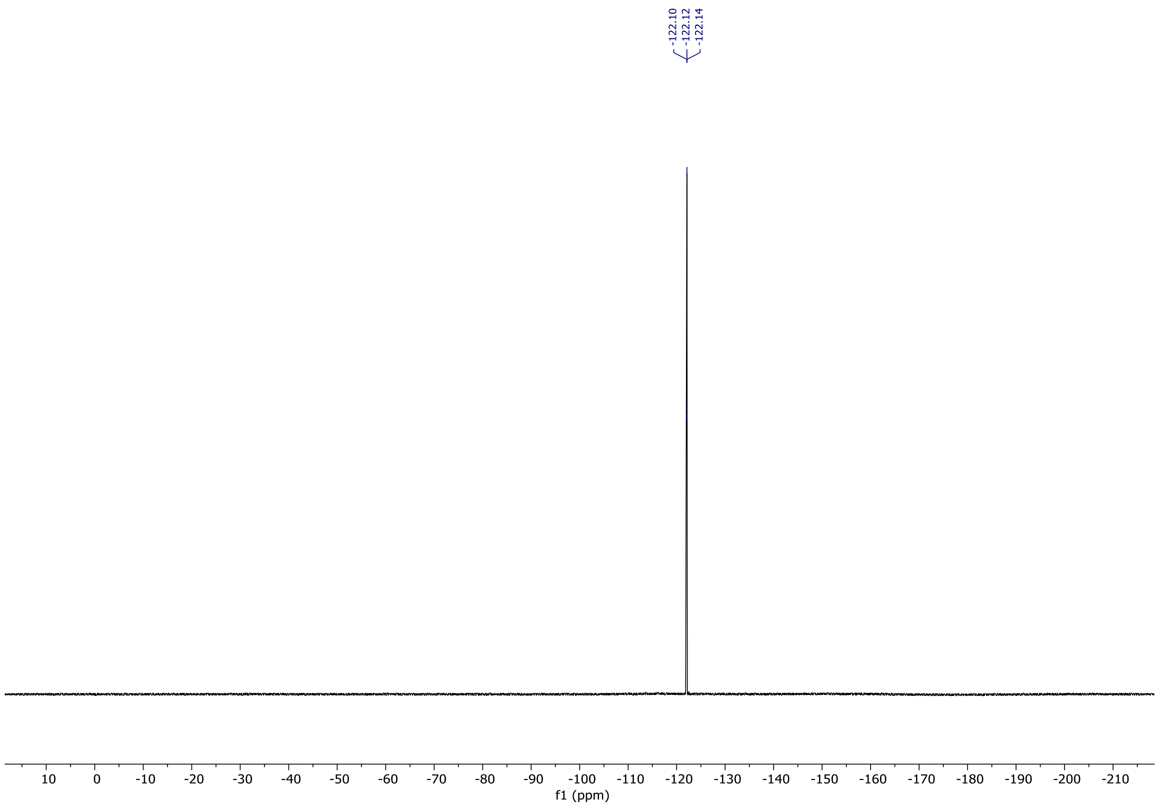

Supplementary Figure 61. ^{19}F NMR spectrum (376 MHz, $CDCl_3$, at rt) of **3j**

2-(3-Fluoro-5-(pyridin-2-yl)phenyl)-2-phenylethan-1-ol **3k**

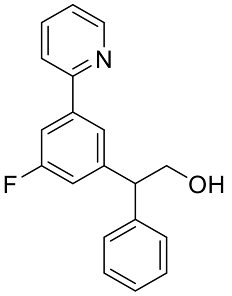

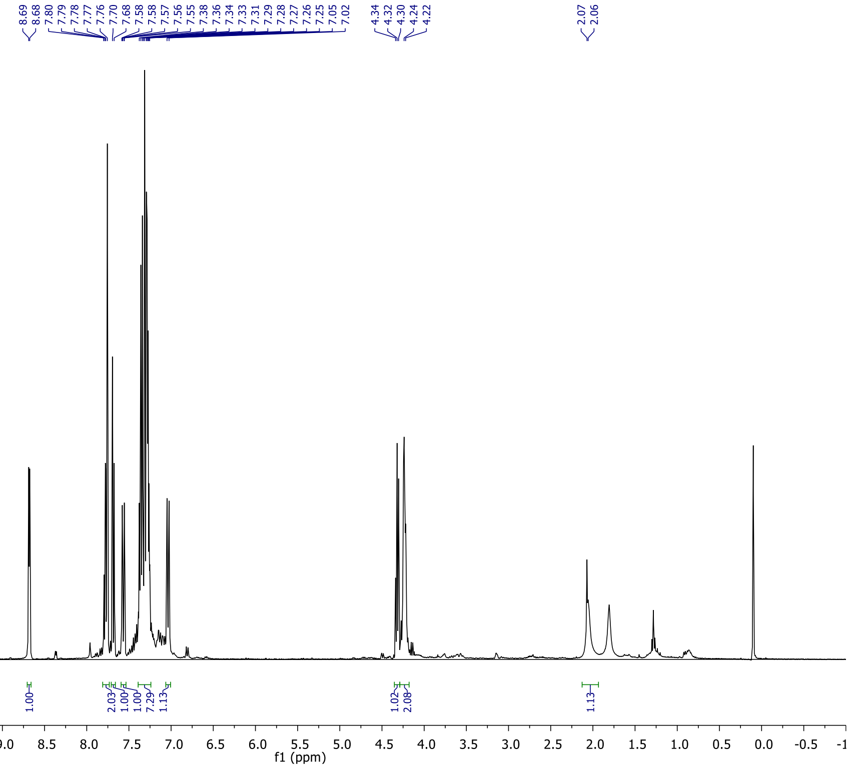

Supplementary Figure 62. ^{1}H NMR spectrum (400 MHz, $CDCl_3$, at rt) of **3k**

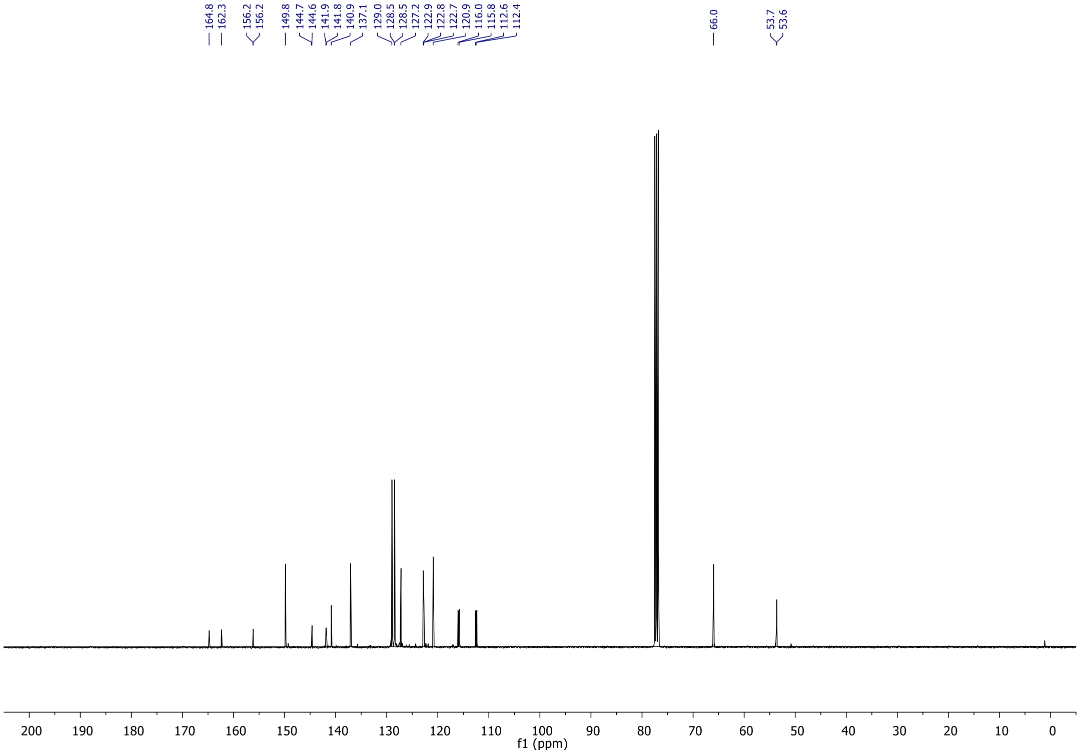

Supplementary Figure 63. ^{13}C NMR spectrum (101 MHz, $CDCl_3$, at rt) of **3k**

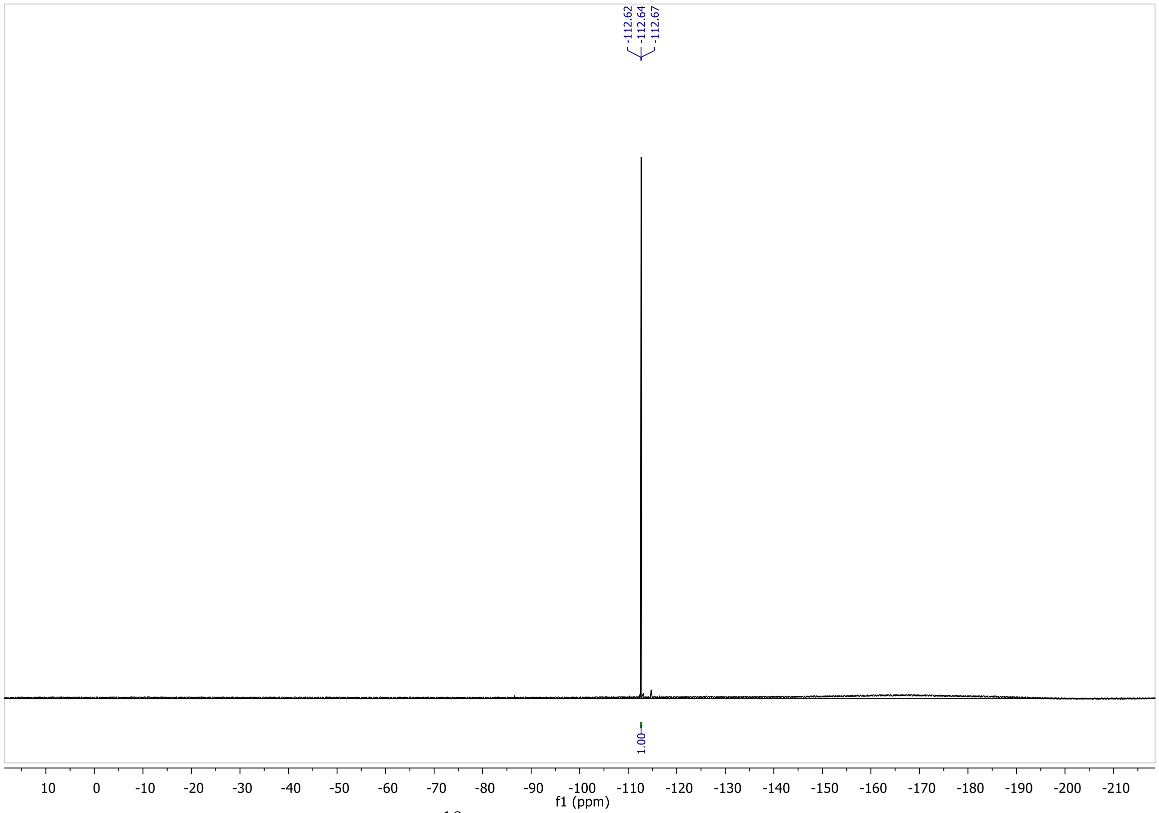

Supplementary Figure 64. ^{19}F NMR spectrum (376 MHz, $CDCl_3$, at rt) of **3k**

2-(2,5-Difluoro-3-(pyridin-2-yl)phenyl)-2-phenylethan-1-ol **3l**

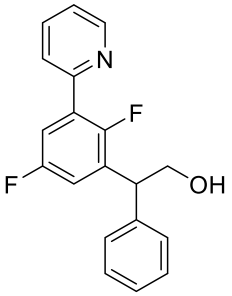

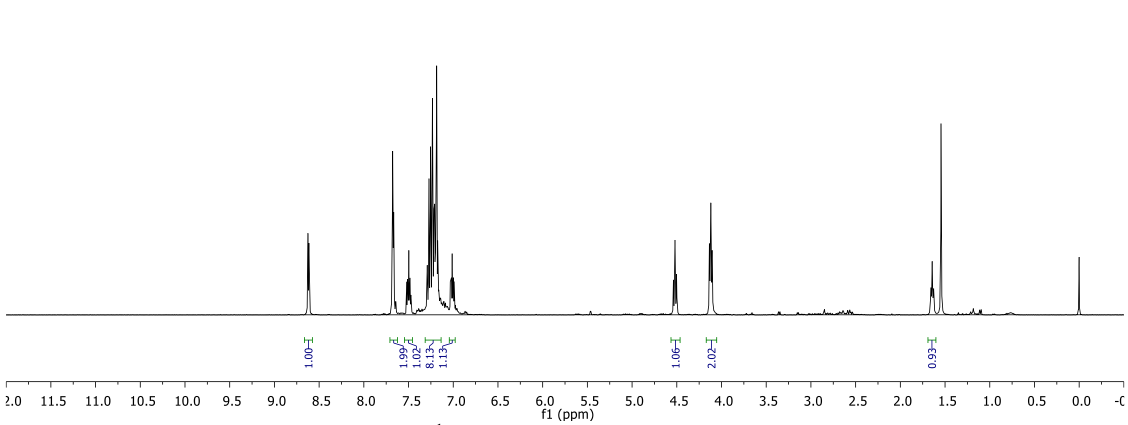

Supplementary Figure 65. ^{1}H NMR spectrum (400 MHz, $CDCl_3$, at rt) of **3l**

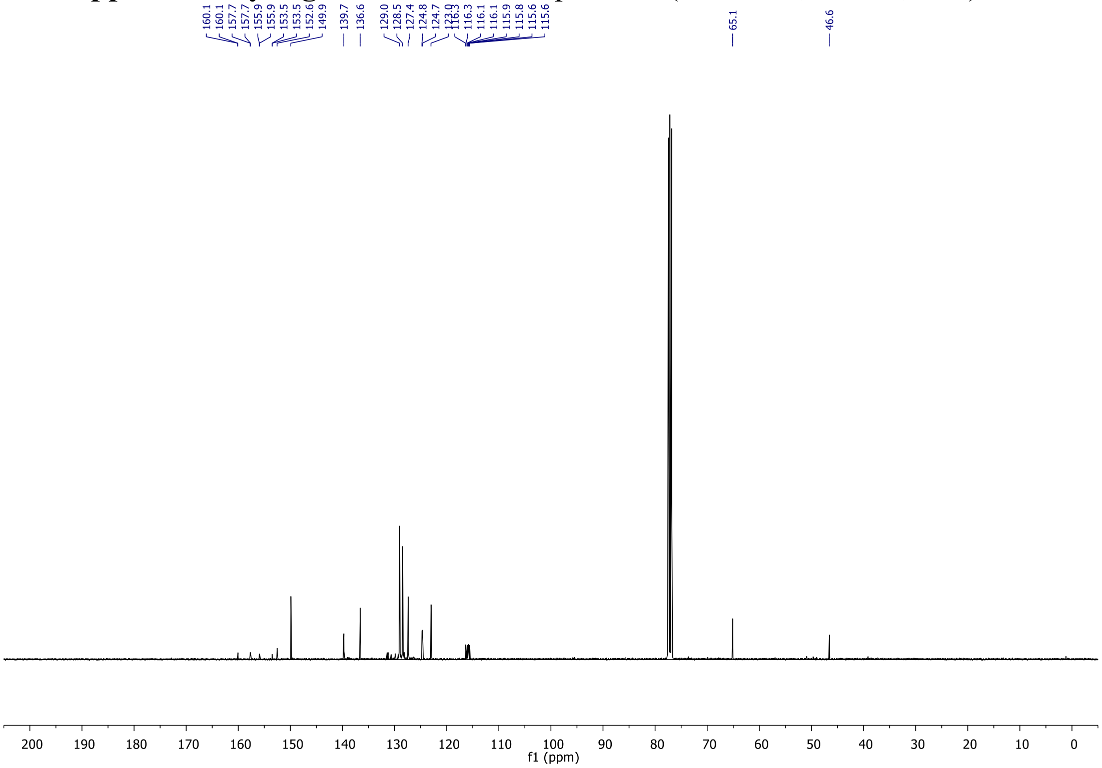

Supplementary Figure 66. ^{13}C NMR spectrum (101 MHz, $CDCl_3$, at rt) of **3l**

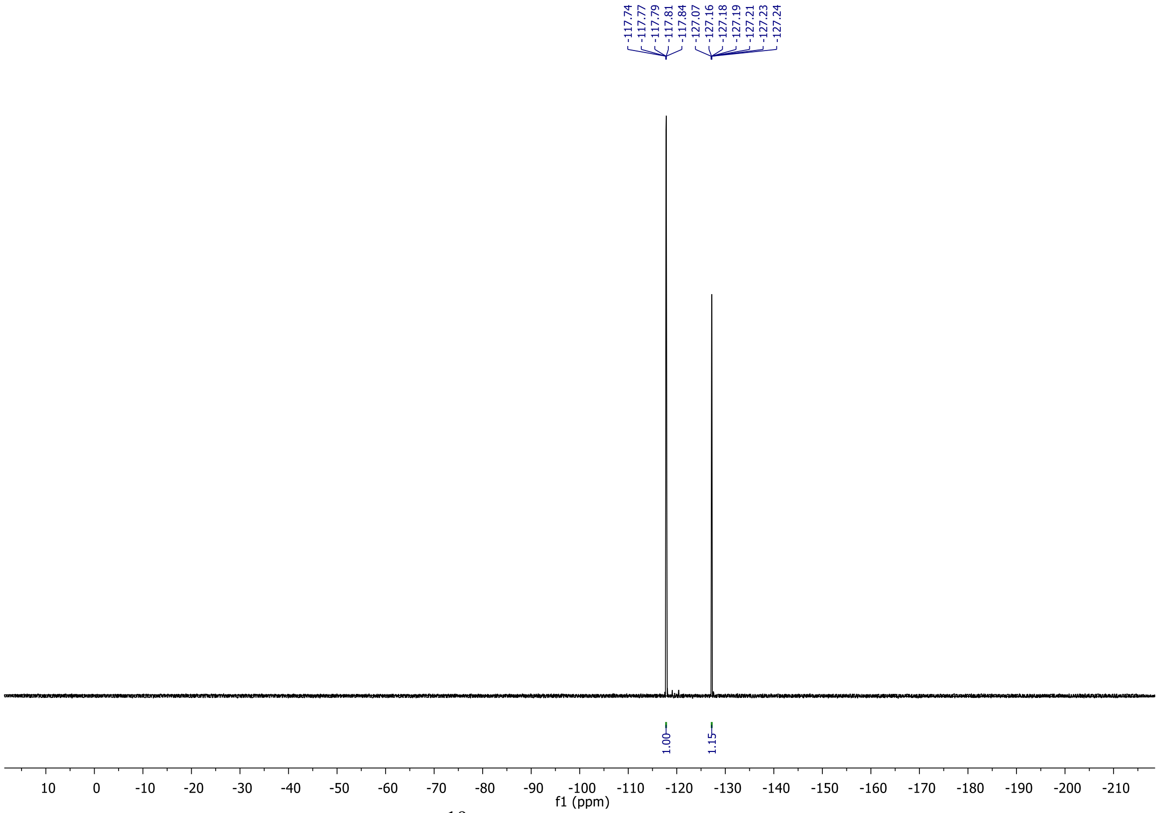

Supplementary Figure 67. ^{19}F NMR spectrum (376 MHz, $CDCl_3$, at rt) of **3l**

2-Phenyl-2-(3-(pyrimidin-2-yl)phenyl)ethan-1-ol **3m**

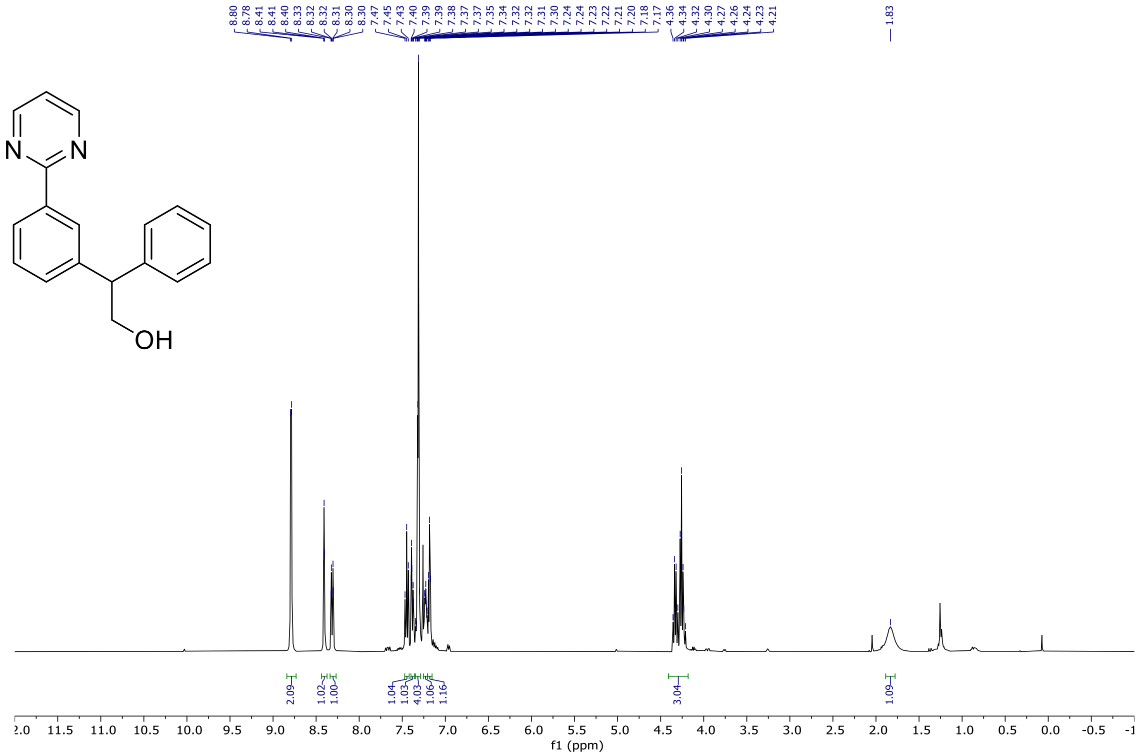

Supplementary Figure 68. ^{1}H NMR spectrum (400 MHz, $CDCl_3$, at rt) of **3m**

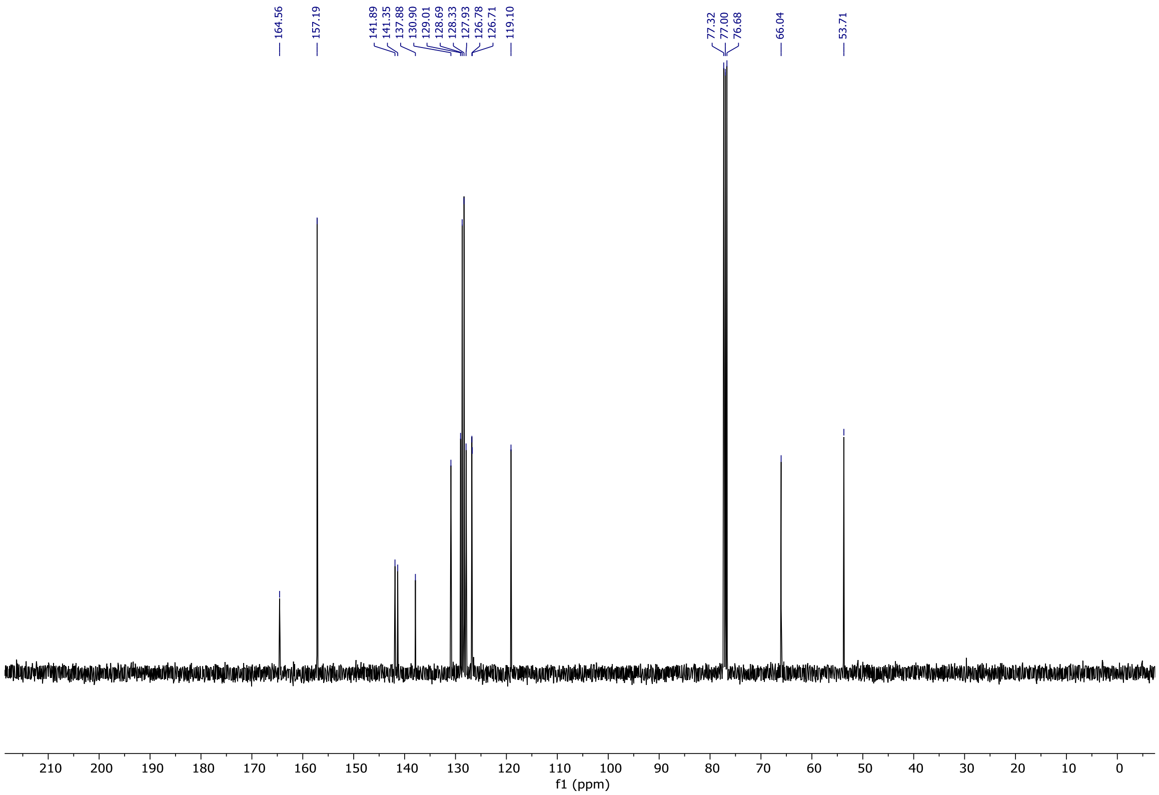

Supplementary Figure 69. ^{13}C NMR spectrum (101 MHz, $CDCl_3$, at rt) of **3m**

2-(3-(1*H*-pyrazol-1-yl)phenyl)-2-phenylethan-1-ol **3n**

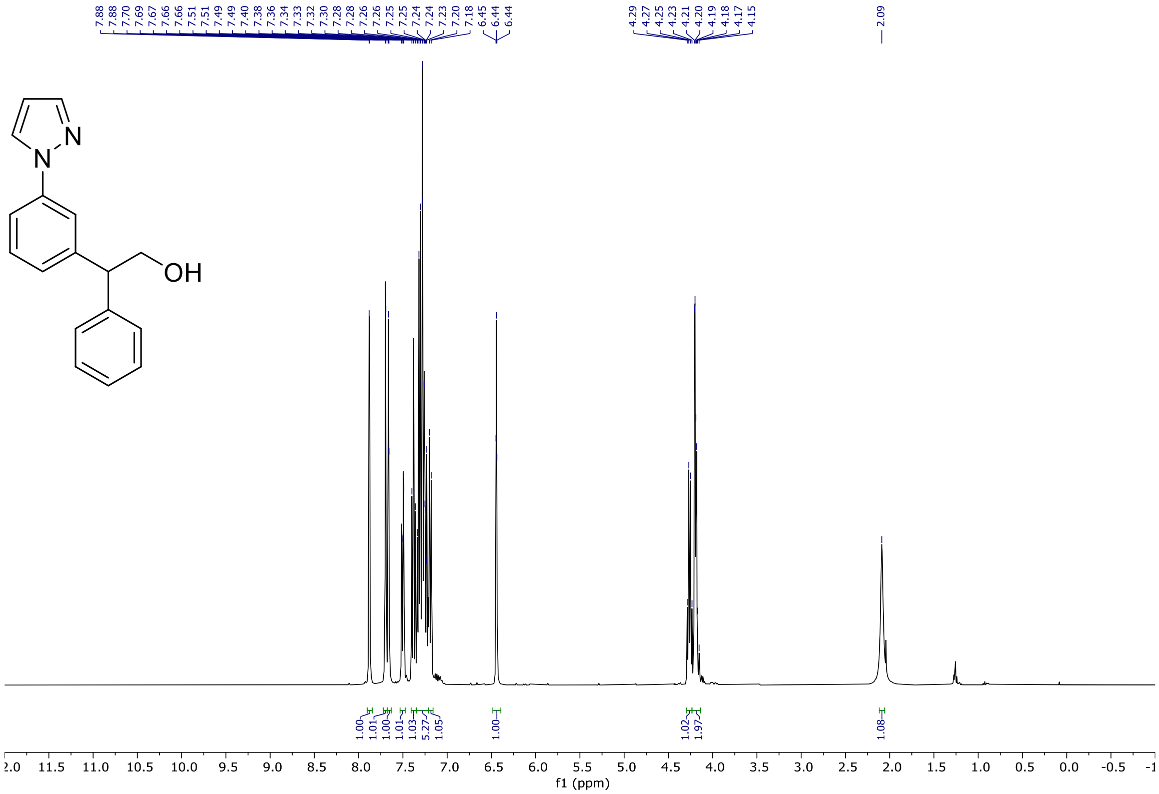

Supplementary Figure 70. ^{1}H NMR spectrum (400 MHz, $CDCl_3$, at rt) of **3n**

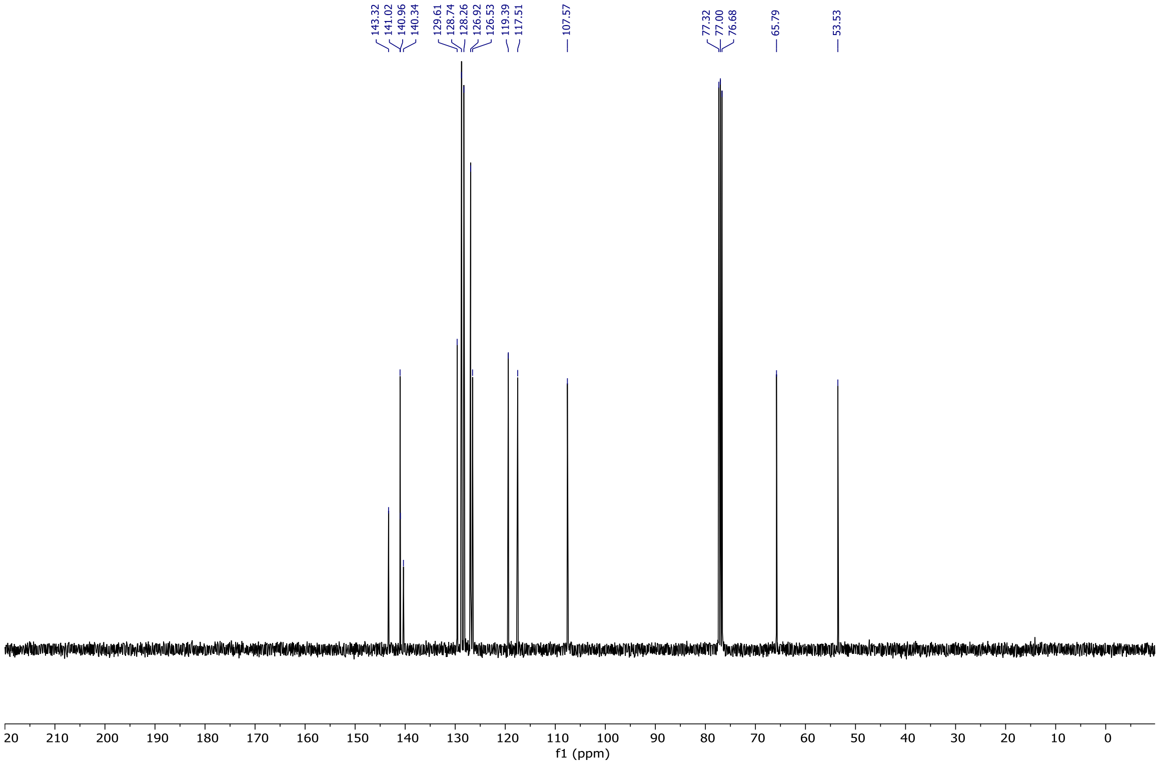

Supplementary Figure 71. ^{13}C NMR spectrum (101 MHz, $CDCl_3$, at rt) of **3n**

2-(3-(4,5-Dihydrooxazol-2-yl)phenyl)-2-phenylethan-1-ol **3o**

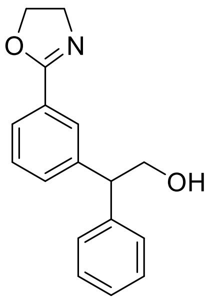

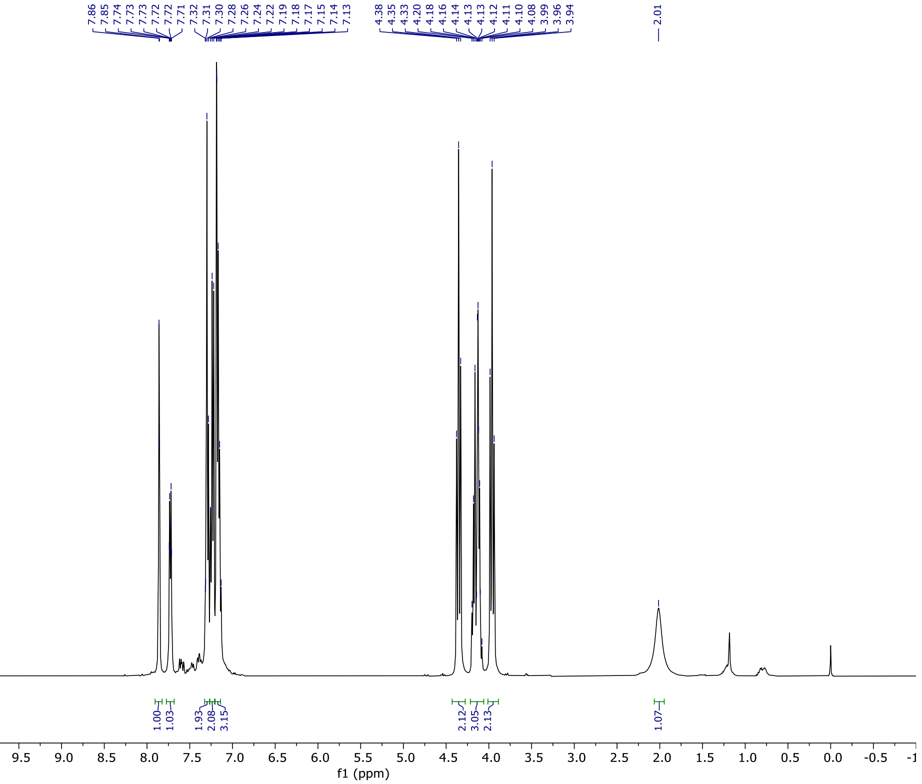

Supplementary Figure 72. ^{1}H NMR spectrum (400 MHz, $CDCl_3$, at rt) of **3o**

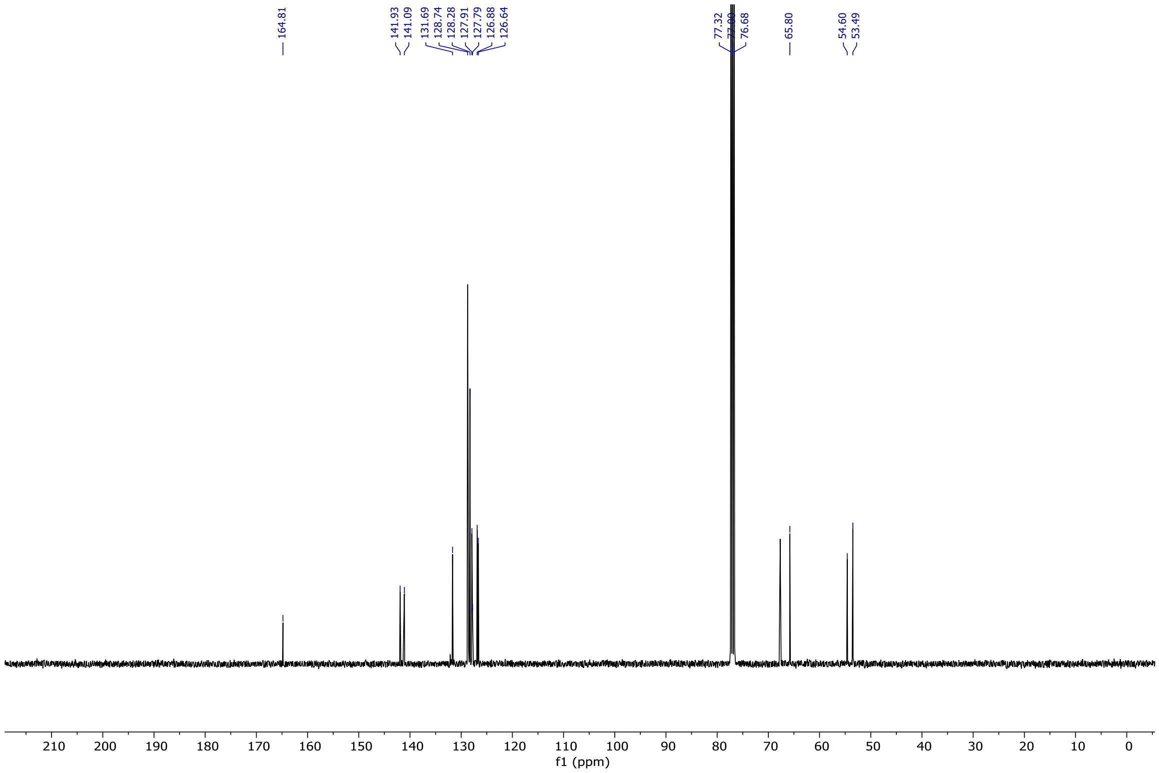

Supplementary Figure 73. ^{13}C NMR spectrum (101 MHz, $CDCl_3$, at rt) of **3o**

4-Phenyl-6-(pyridin-2-yl)isochroman-1-one **3p**

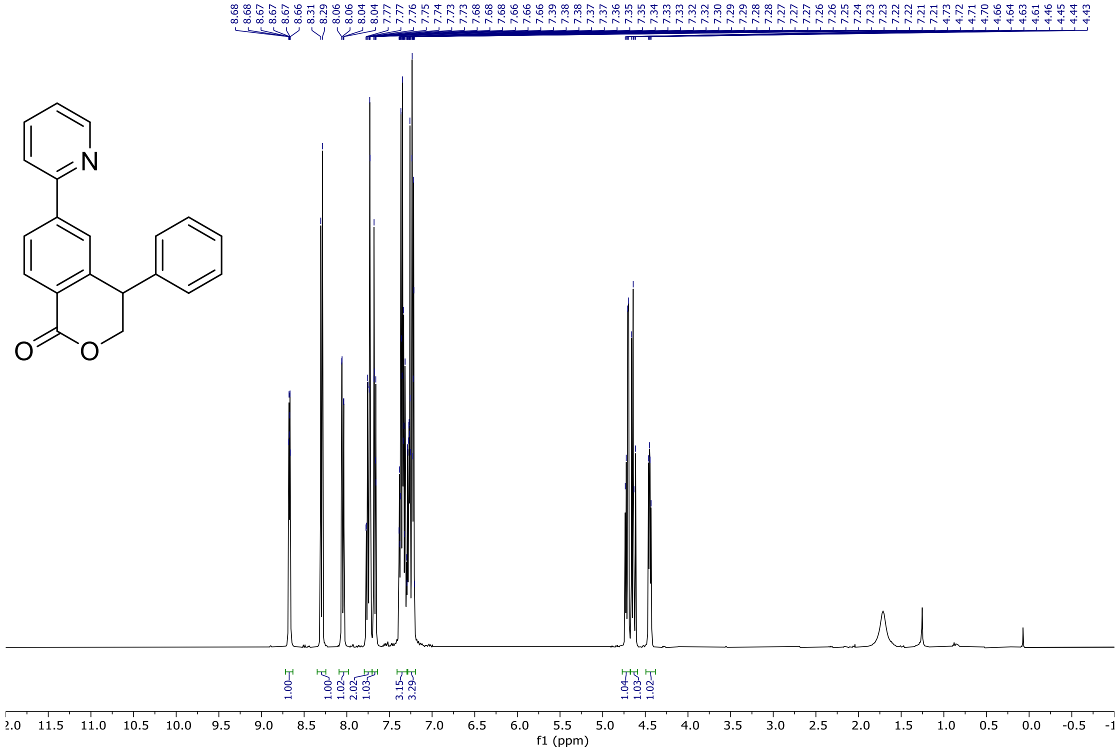

Supplementary Figure 74. ^{1}H NMR spectrum (400 MHz, $CDCl_3$, at rt) of **3p**

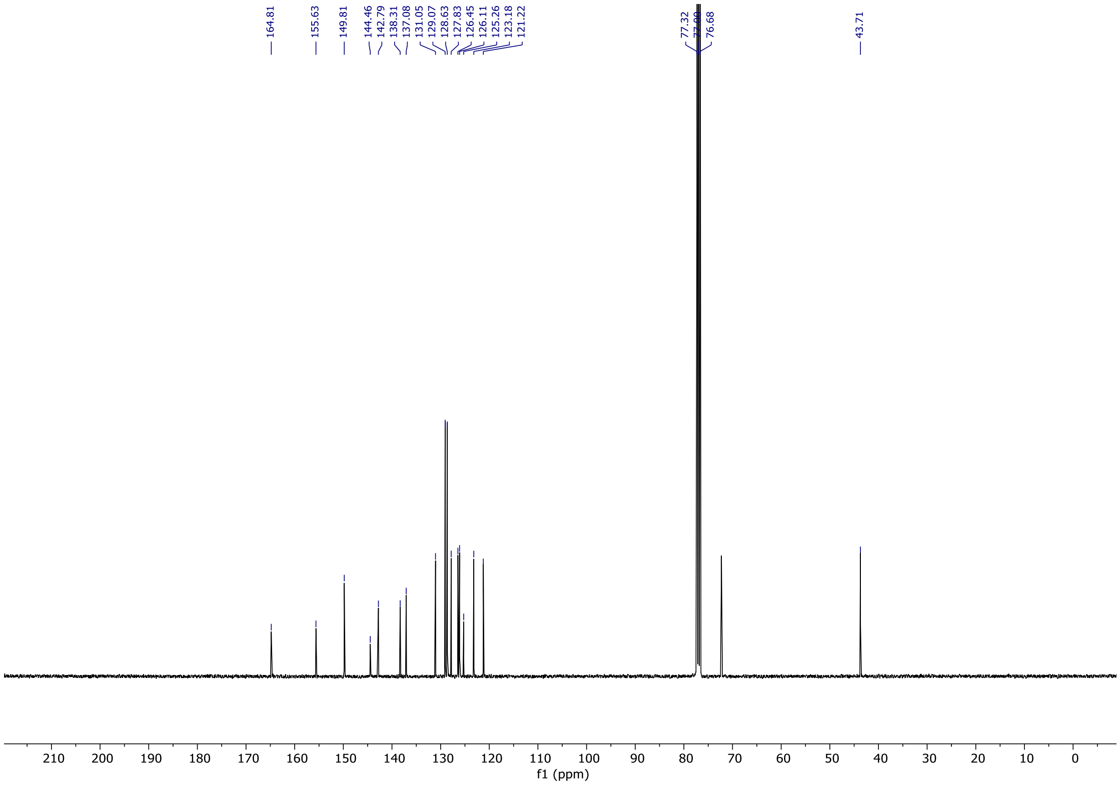

Supplementary Figure 75. ^{13}C NMR spectrum (101 MHz, $CDCl_3$, at rt) of **3p**

2-(4-Cyclohexylphenyl)-2-(3-(pyridin-2-yl)phenyl)ethan-1-ol **3q**

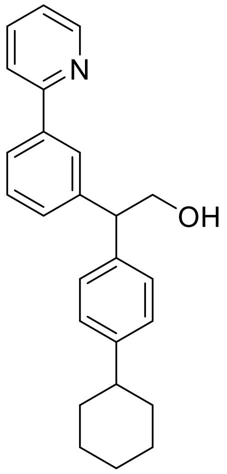

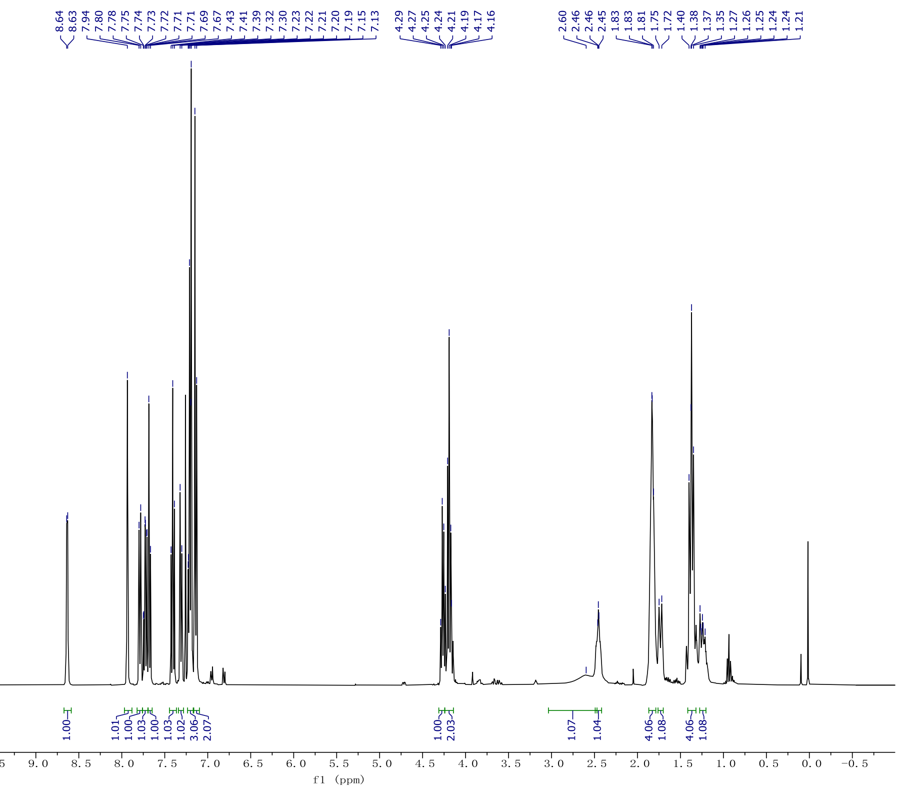

Supplementary Figure 76. ^{1}H NMR spectrum (400 MHz, $CDCl_3$, at rt) of **3q**

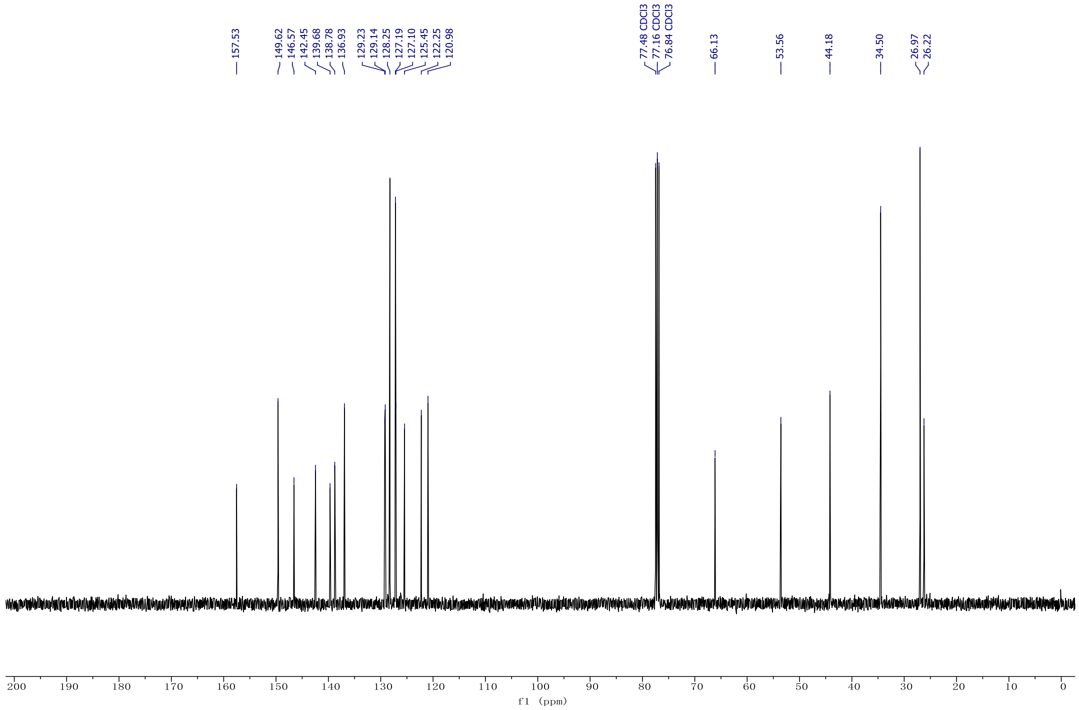

Supplementary Figure 77. ^{13}C NMR spectrum (101 MHz, $CDCl_3$, at rt) of **3q**

2-(4-(*Tert*-butyl)phenyl)-2-(3-(pyridin-2-yl)phenyl)ethan-1-ol **3r**

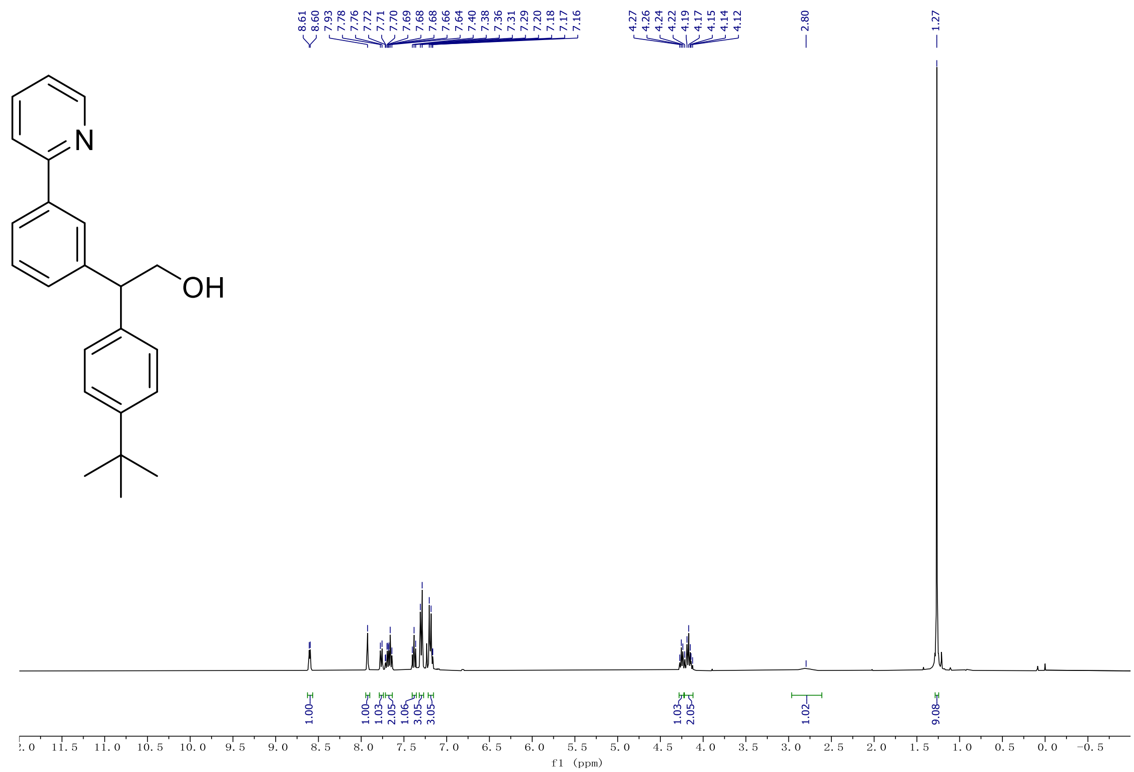

Supplementary Figure 78. ^{1}H NMR spectrum (400 MHz, $CDCl_3$, at rt) of **3r**

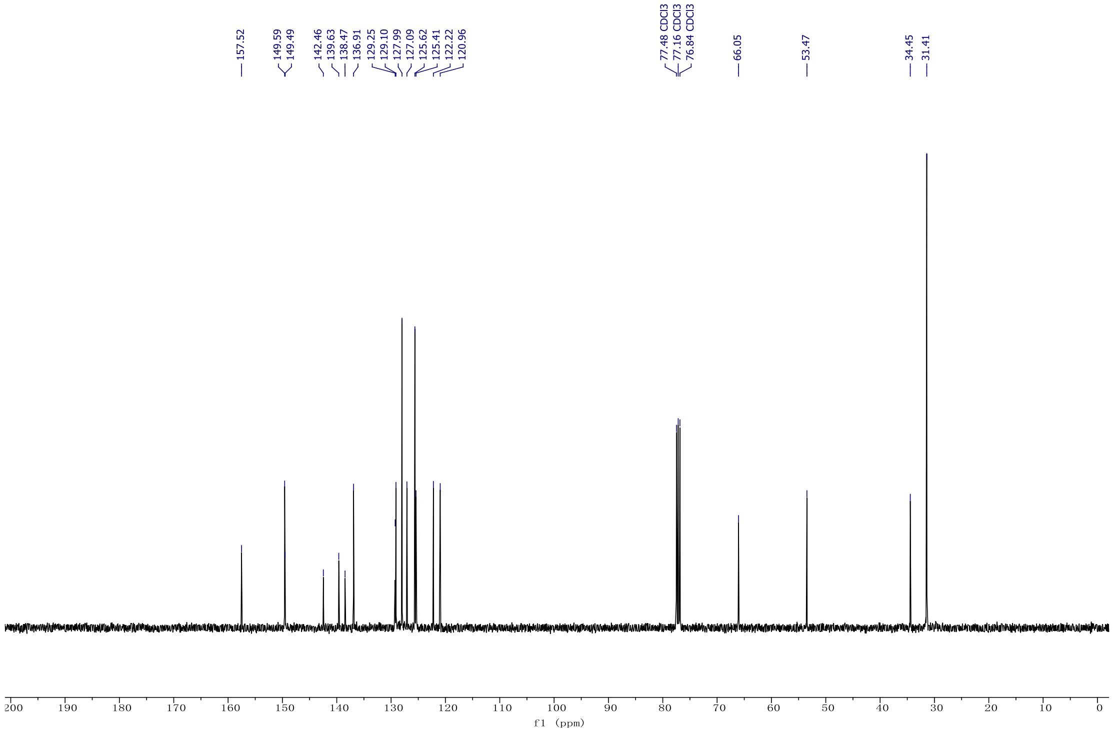

Supplementary Figure 79. ^{13}C NMR spectrum (101 MHz, $CDCl_3$, at rt) of **3r**

2-([1,1'-Biphenyl]-4-yl)-2-(3-(pyridin-2-yl)phenyl)ethan-1-ol **3s**

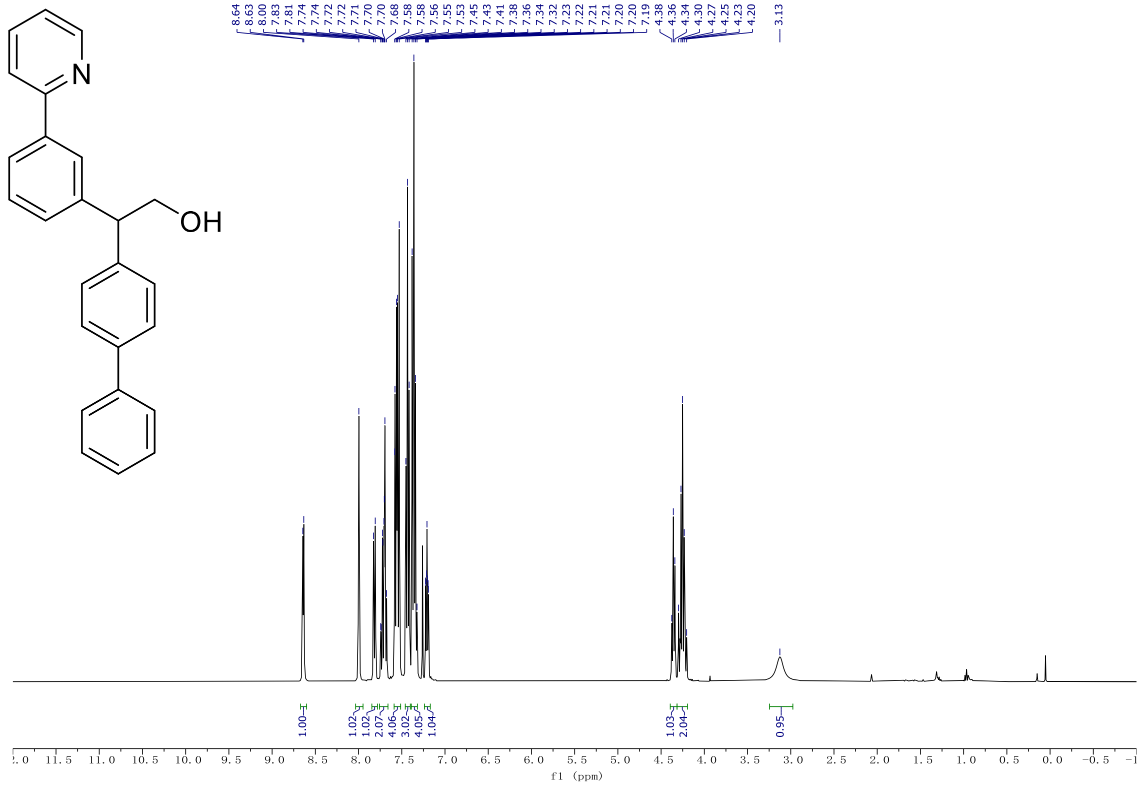

Supplementary Figure 80. ^{1}H NMR spectrum (400 MHz, $CDCl_3$, at rt) of **3s**

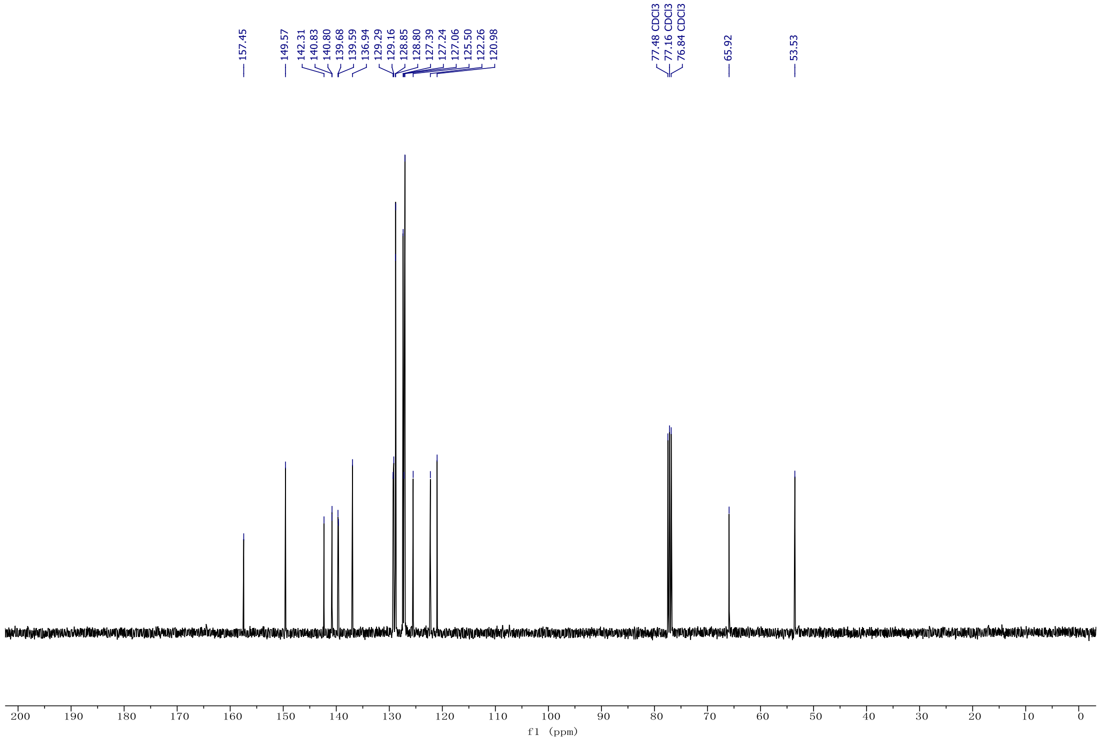

Supplementary Figure 81. ^{13}C NMR spectrum (101 MHz, $CDCl_3$, at rt) of **3s**

2-(4-Fluorophenyl)-2-(3-(pyridin-2-yl)phenyl)ethan-1-ol **3t**

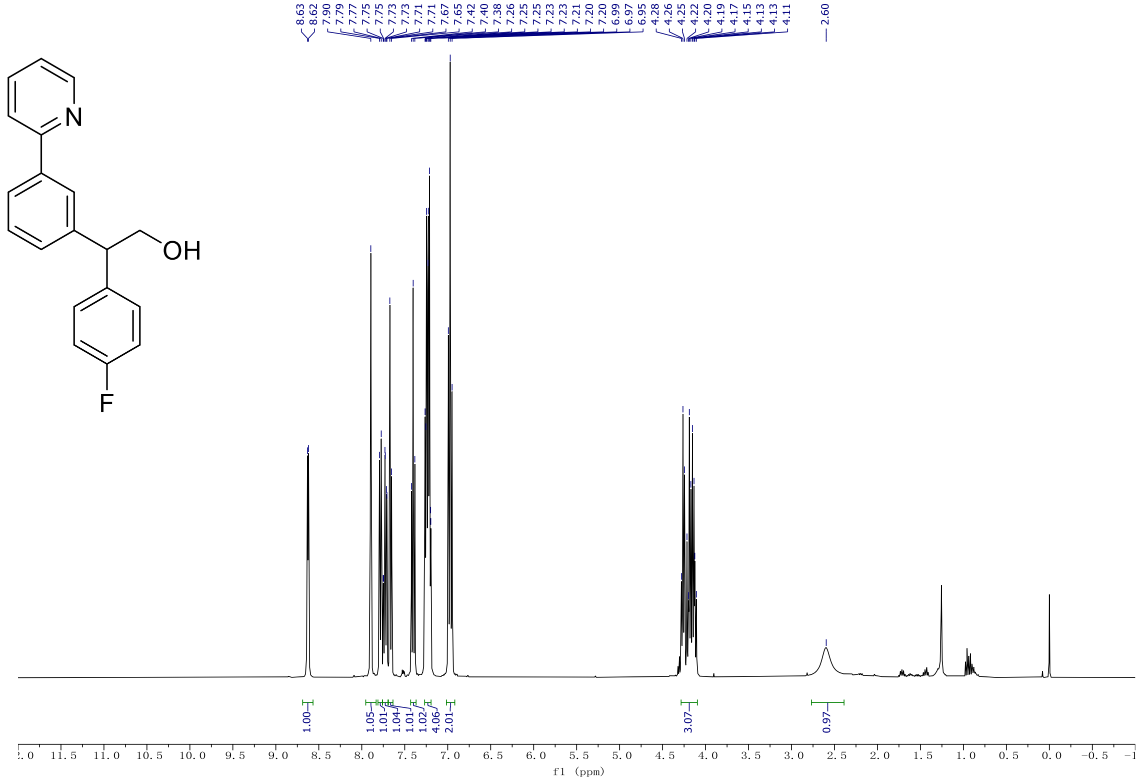

Supplementary Figure 82. ^{1}H NMR spectrum (400 MHz, $CDCl_3$, at rt) of **3t**

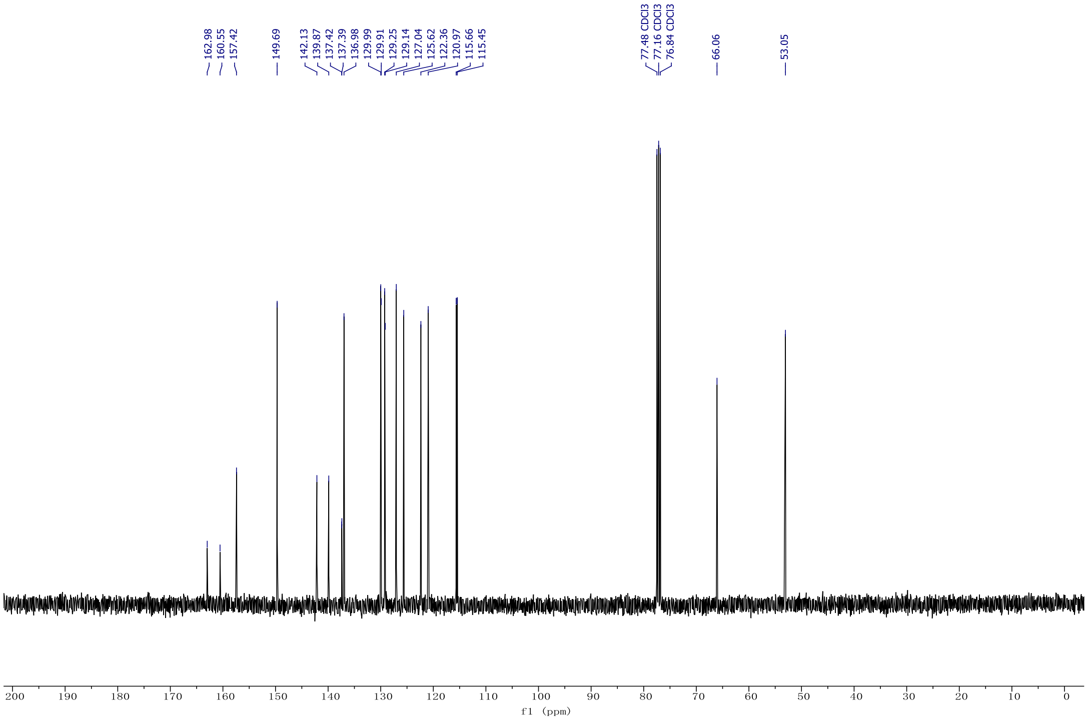

Supplementary Figure 83. ^{13}C NMR spectrum (101 MHz, $CDCl_3$, at rt) of **3t**

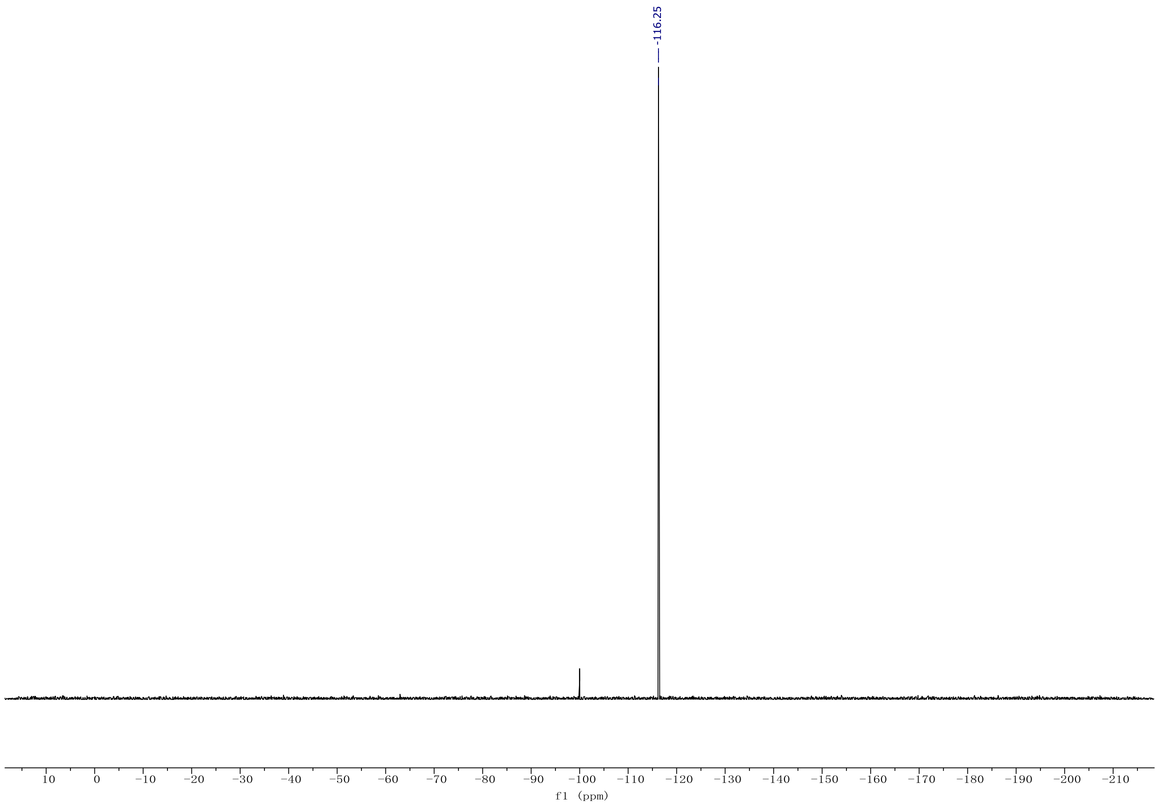

Supplementary Figure 84. ^{19}F NMR spectrum (376 MHz, $CDCl_3$, at rt) of **3t**

2-(4-Chlorophenyl)-2-(3-(pyridin-2-yl)phenyl)ethan-1-ol **3u**

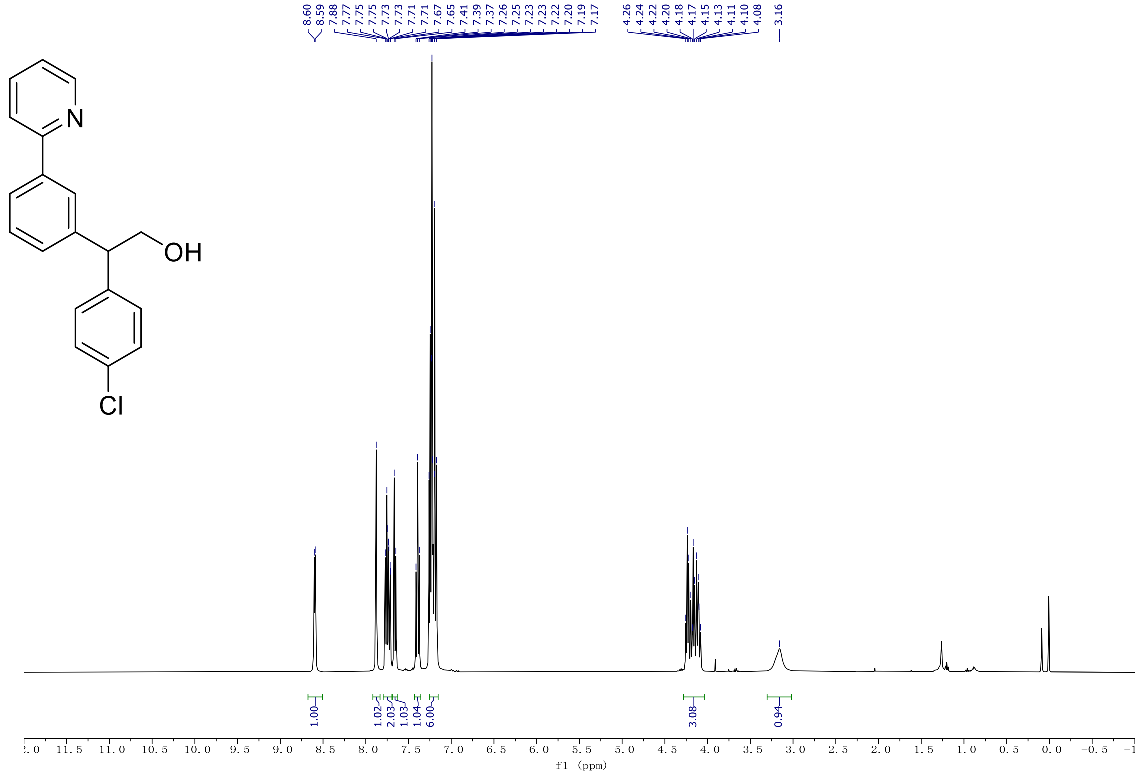

Supplementary Figure 85. ^{1}H NMR spectrum (400 MHz, $CDCl_3$, at rt) of **3u**

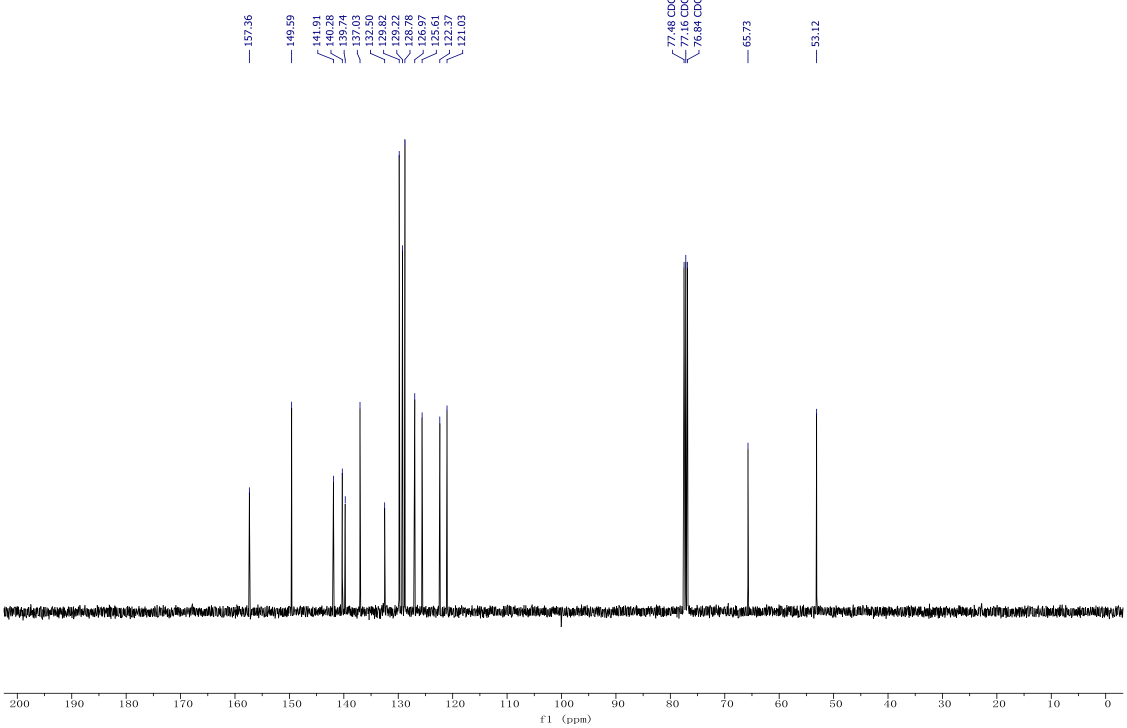

Supplementary Figure 86. ^{13}C NMR spectrum (101 MHz, $CDCl_3$, at rt) of **3u**

2-(4-Bromophenyl)-2-(3-(pyridin-2-yl)phenyl)ethan-1-ol **3v**

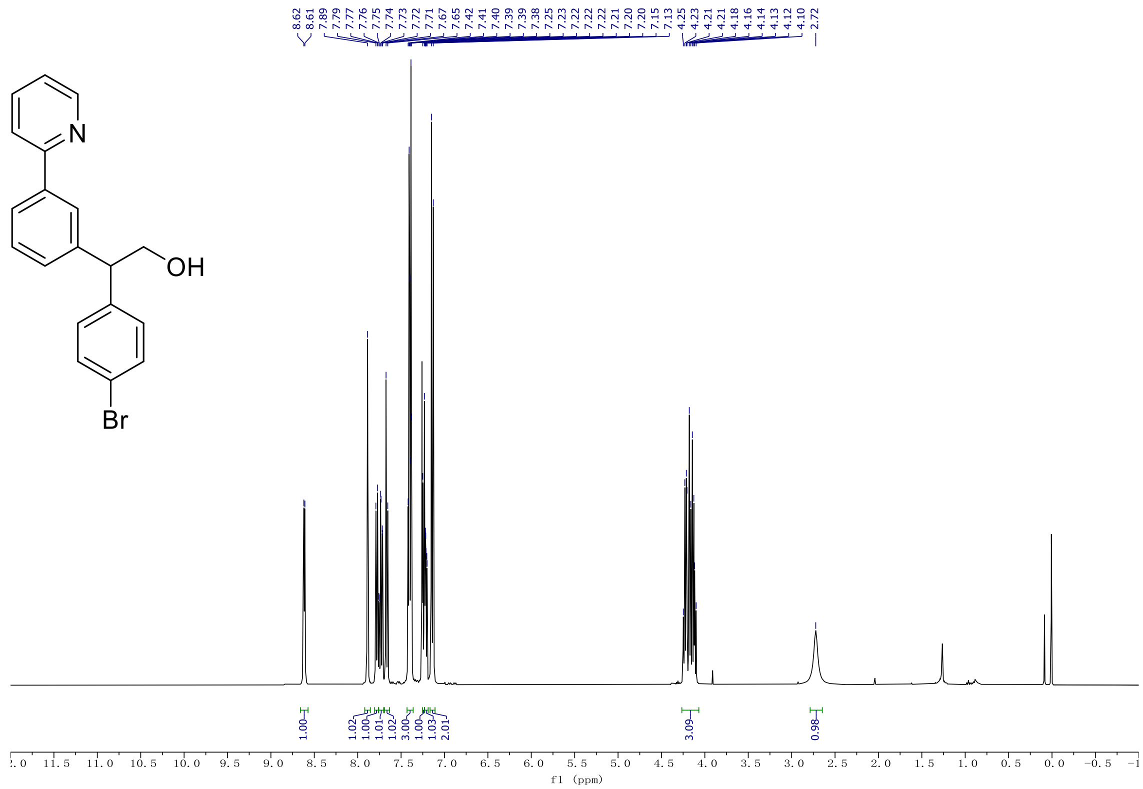

Supplementary Figure 87. ^{1}H NMR spectrum (400 MHz, $CDCl_3$, at rt) of **3v**

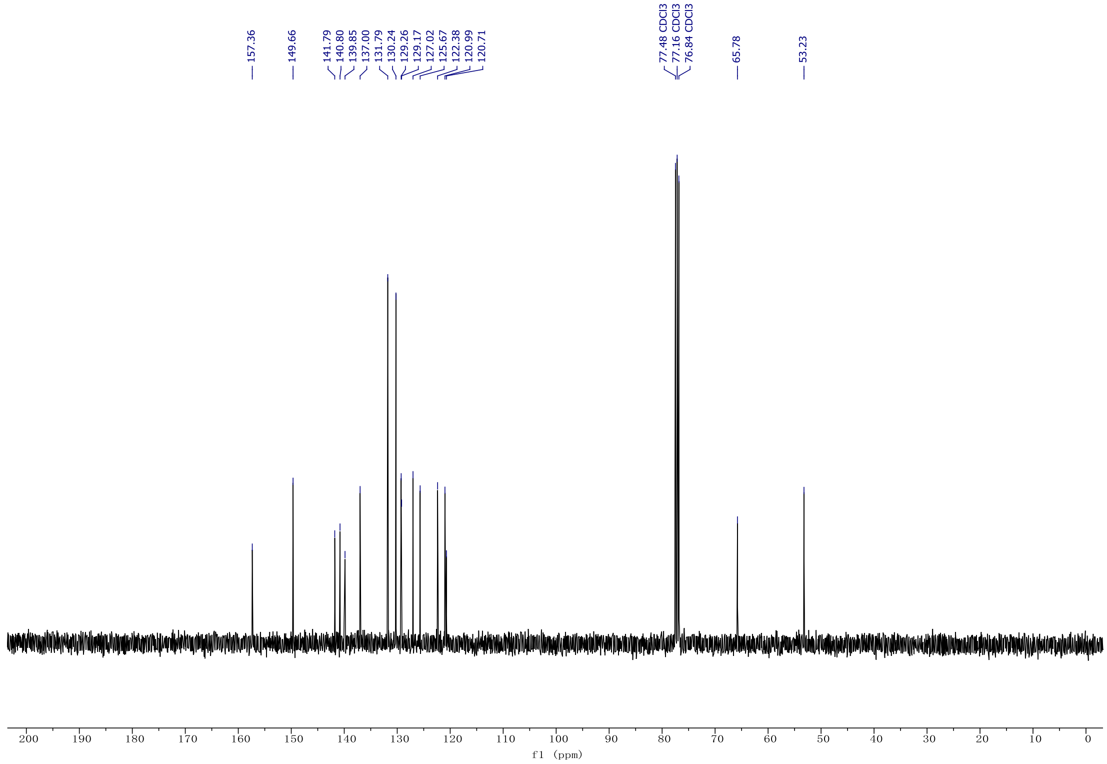

Supplementary Figure 88. ^{13}C NMR spectrum (101 MHz, $CDCl_3$, at rt) of **3v**

2-(3-(Pyridin-2-yl)phenyl)-2-(4-(trifluoromethyl)phenyl)ethan-1-ol **3w**

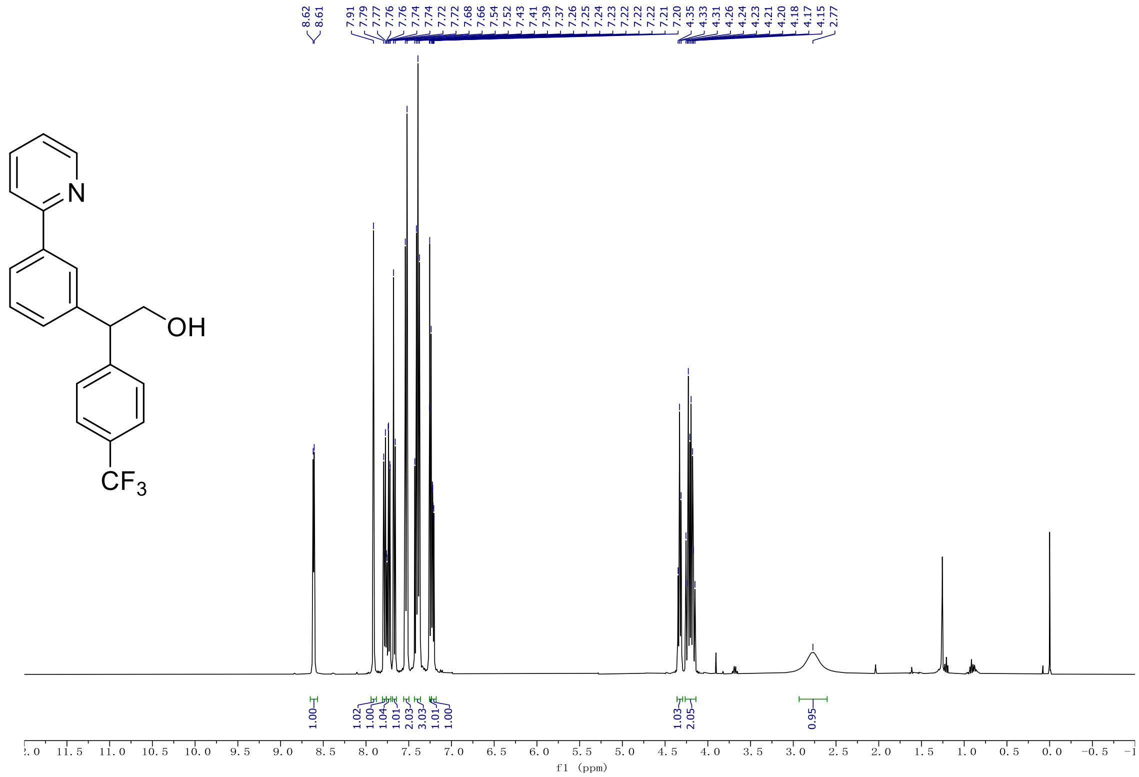

Supplementary Figure 89. ^{1}H NMR spectrum (400 MHz, $CDCl_3$, at rt) of **3w**

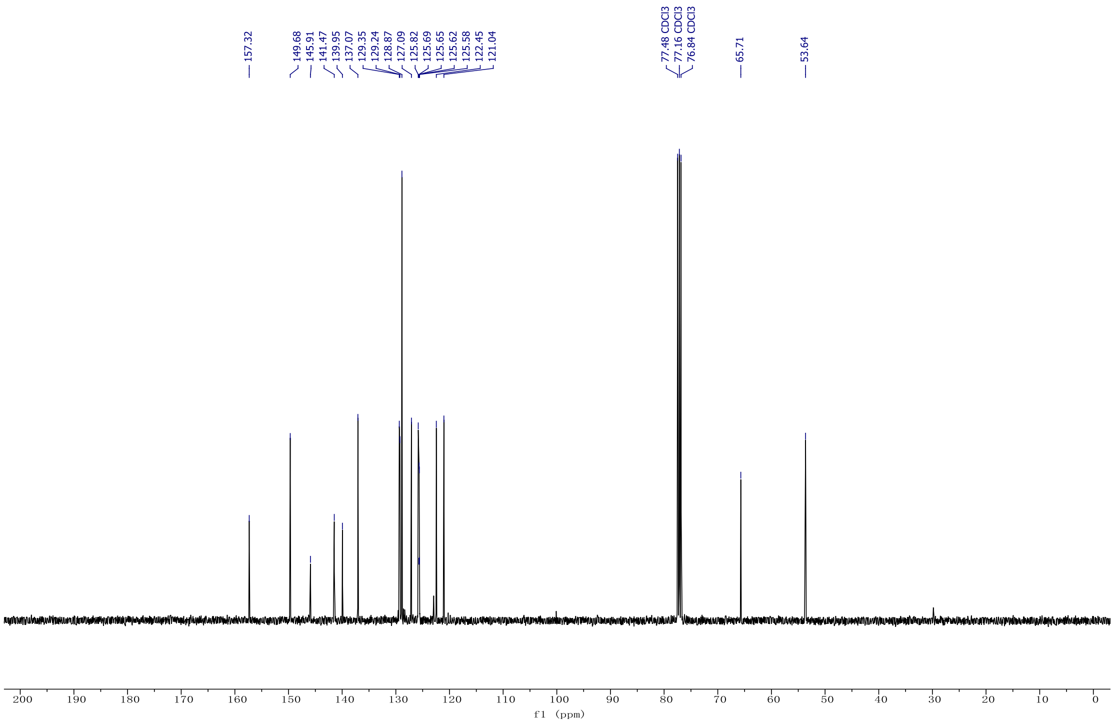

Supplementary Figure 90. ^{13}C NMR spectrum (101 MHz, $CDCl_3$, at rt) of **3w**

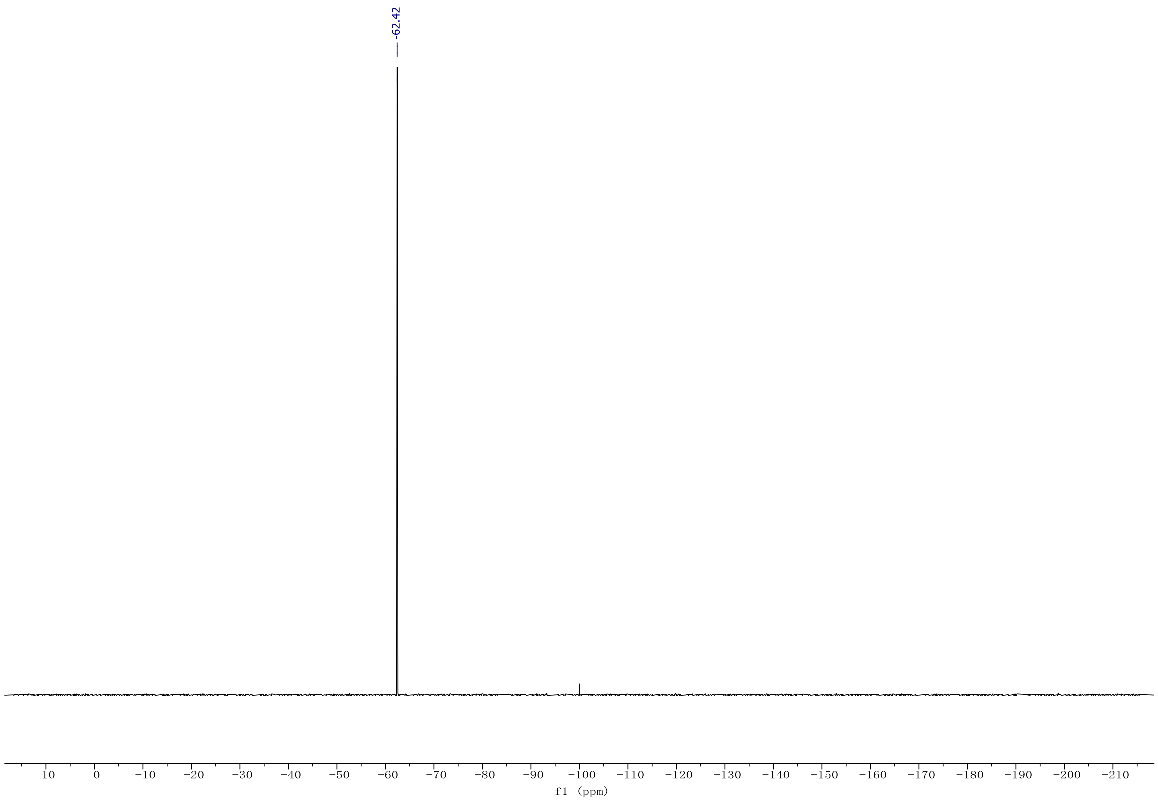

Supplementary Figure 91. ^{19}F NMR spectrum (376 MHz, $CDCl_3$, at rt) of **3w**

Methyl 4-(2-hydroxy-1-(3-(pyridin-2-yl)phenyl)ethyl)benzoate **3x**

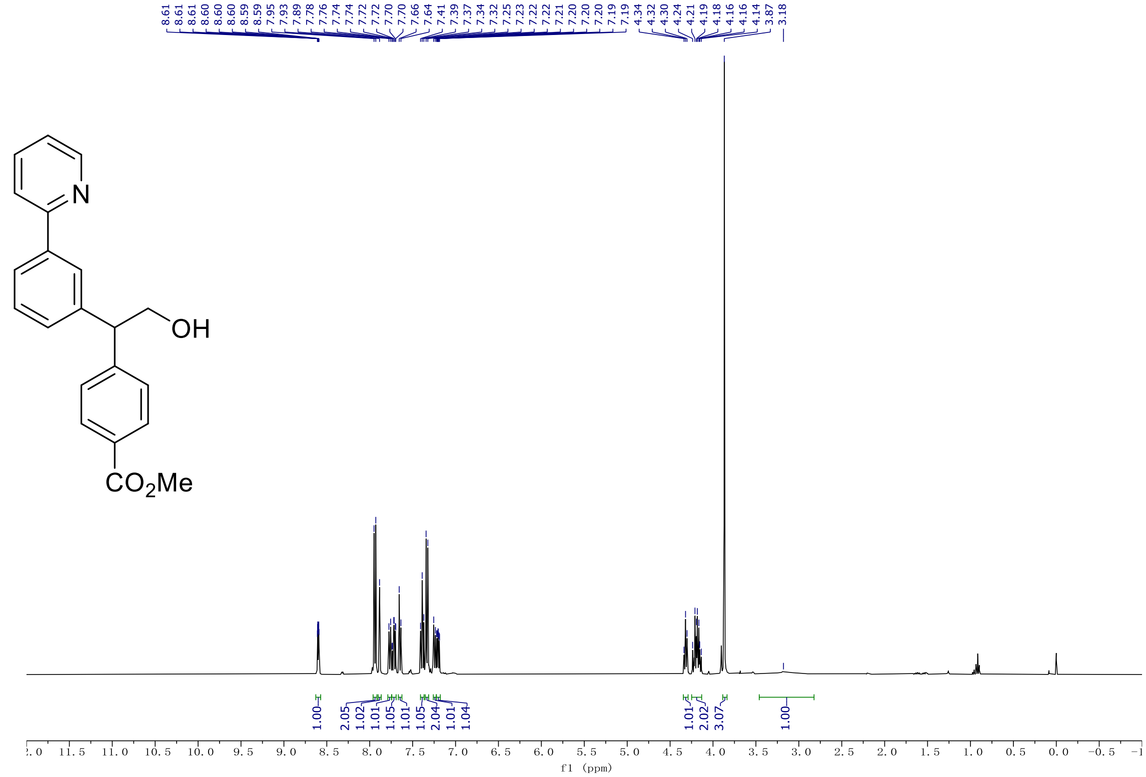

Supplementary Figure 92. ^{1}H NMR spectrum (400 MHz, $CDCl_3$, at rt) of **3x**

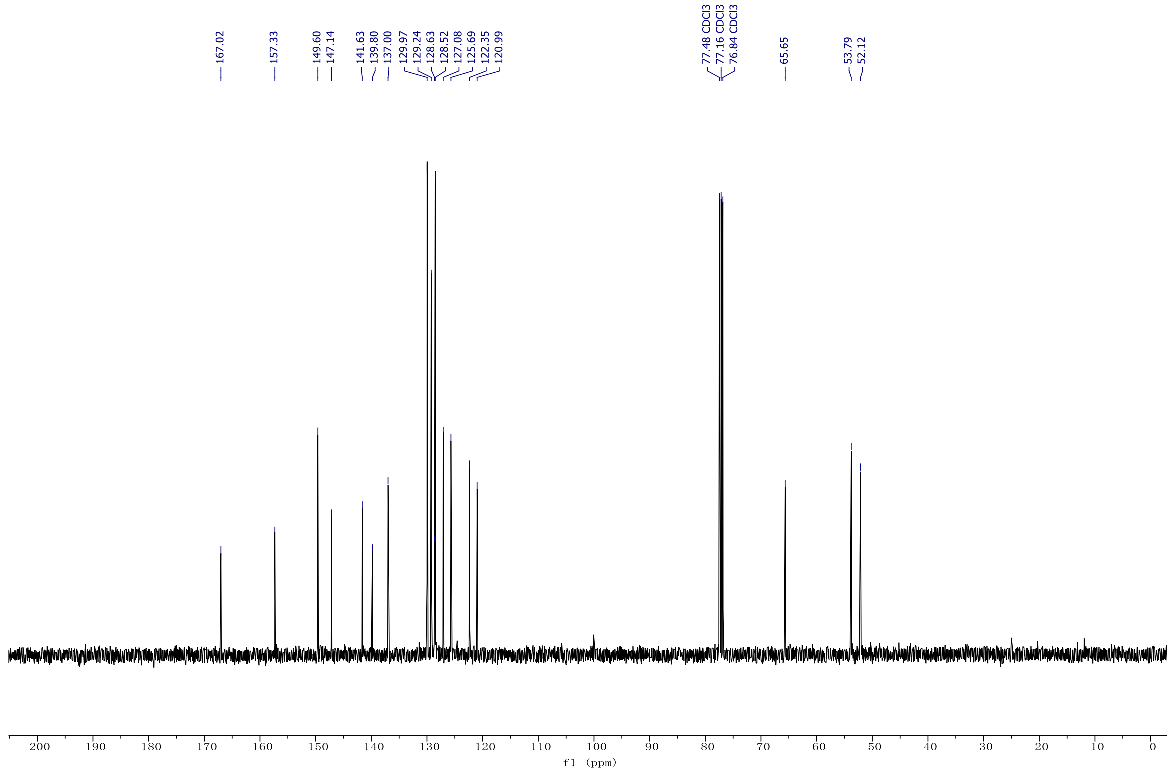

Supplementary Figure 93. ^{13}C NMR spectrum (101 MHz, $CDCl_3$, at rt) of **3x**

2-(3-(Pyridin-2-yl)phenyl)-2-(o-tolyl)ethan-1-ol **3y**

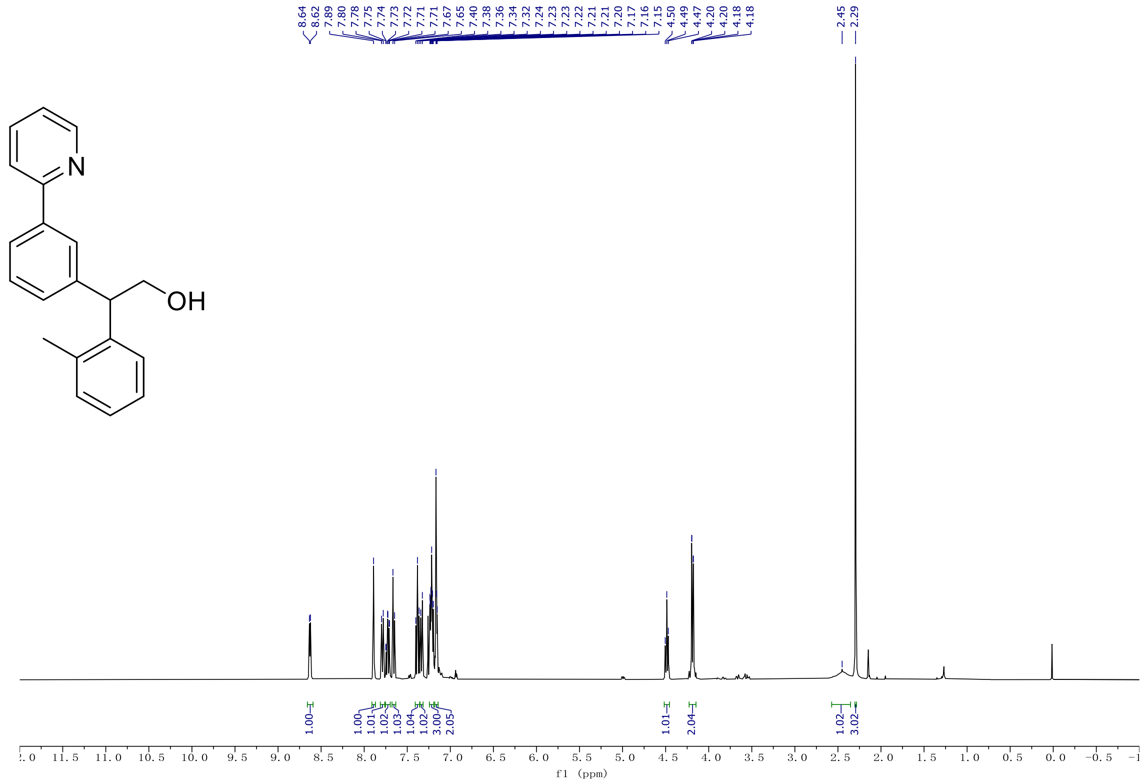

Supplementary Figure 94. ^{1}H NMR spectrum (400 MHz, $CDCl_3$, at rt) of **3y**

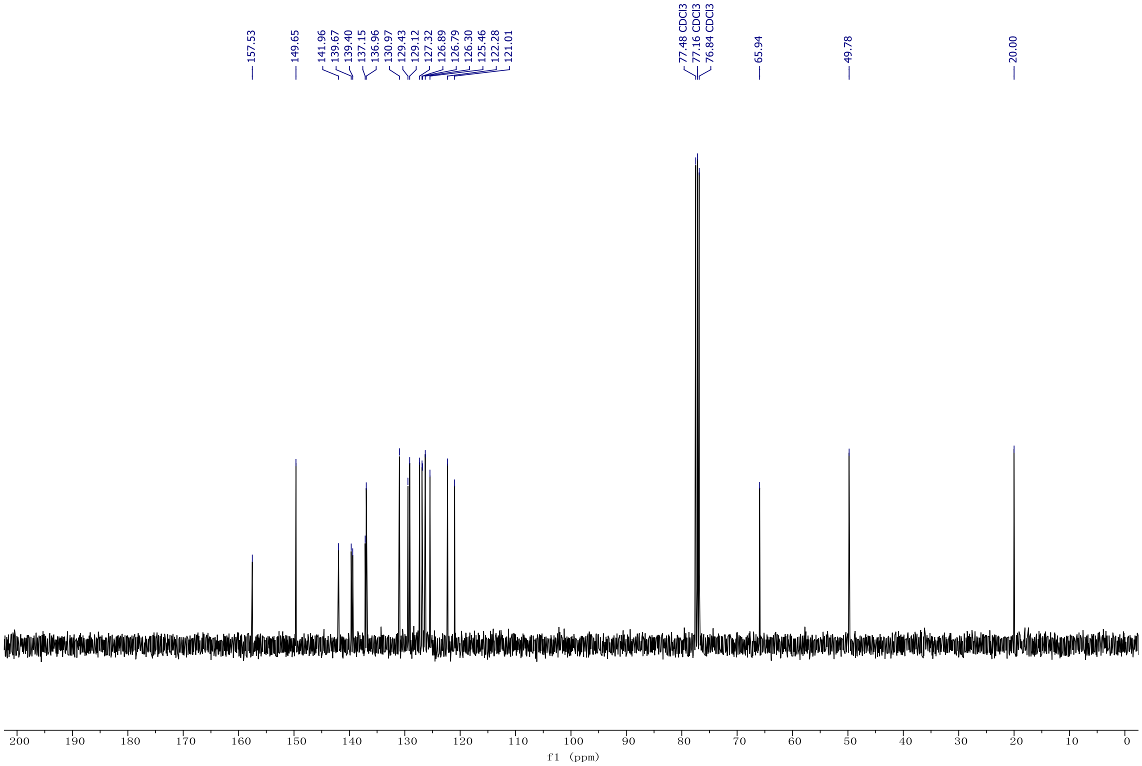

Supplementary Figure 95. ^{13}C NMR spectrum (101 MHz, $CDCl_3$, at rt) of **3y**

2-(2-Methoxyphenyl)-2-(3-(pyridin-2-yl)phenyl)ethan-1-ol **3z**

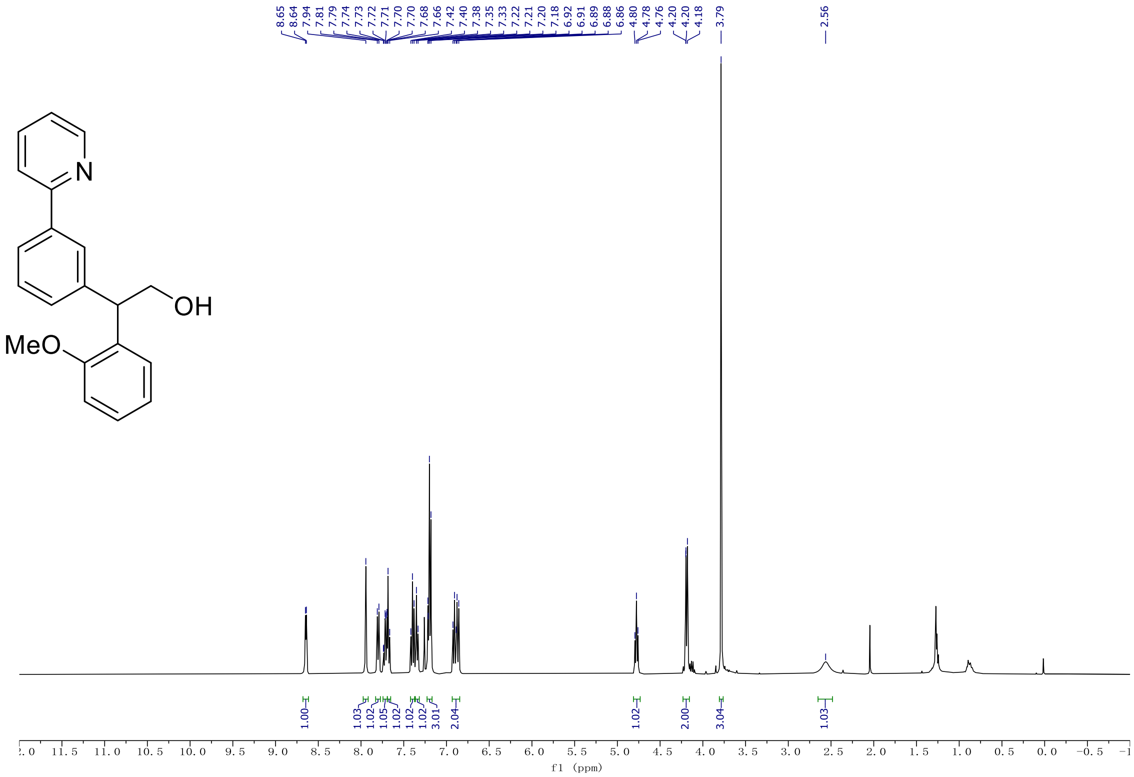

Supplementary Figure 96. ^{1}H NMR spectrum (400 MHz, $CDCl_3$, at rt) of **3z**

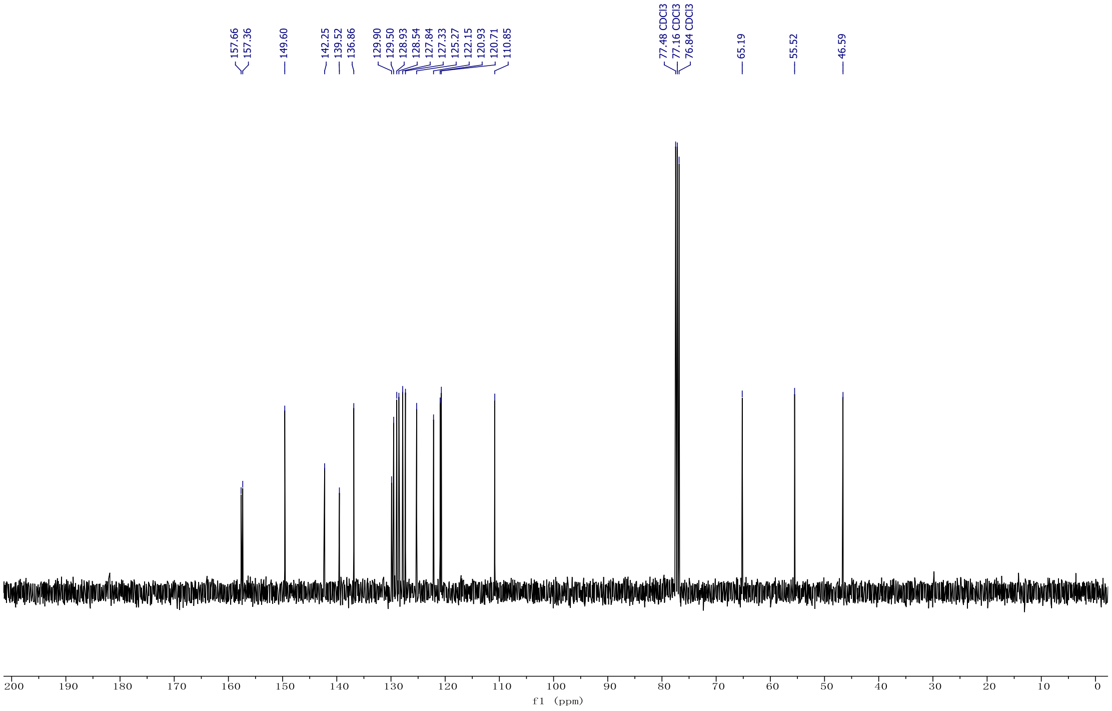

Supplementary Figure 97. ^{13}C NMR spectrum (101 MHz, $CDCl_3$, at rt) of **3z**

2-(2-Fluorophenyl)-2-(3-(pyridin-2-yl)phenyl)ethan-1-ol **3aa**

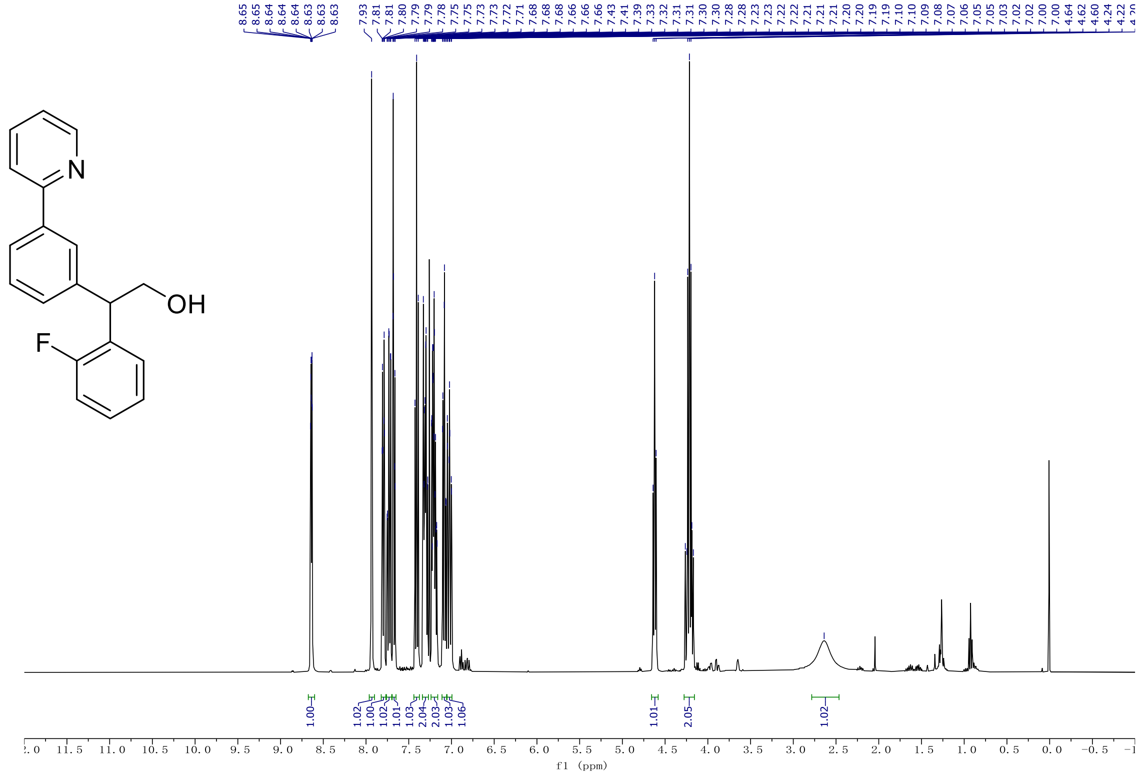

Supplementary Figure 98. ^{1}H NMR spectrum (400 MHz, $CDCl_3$, at rt) of **3aa**

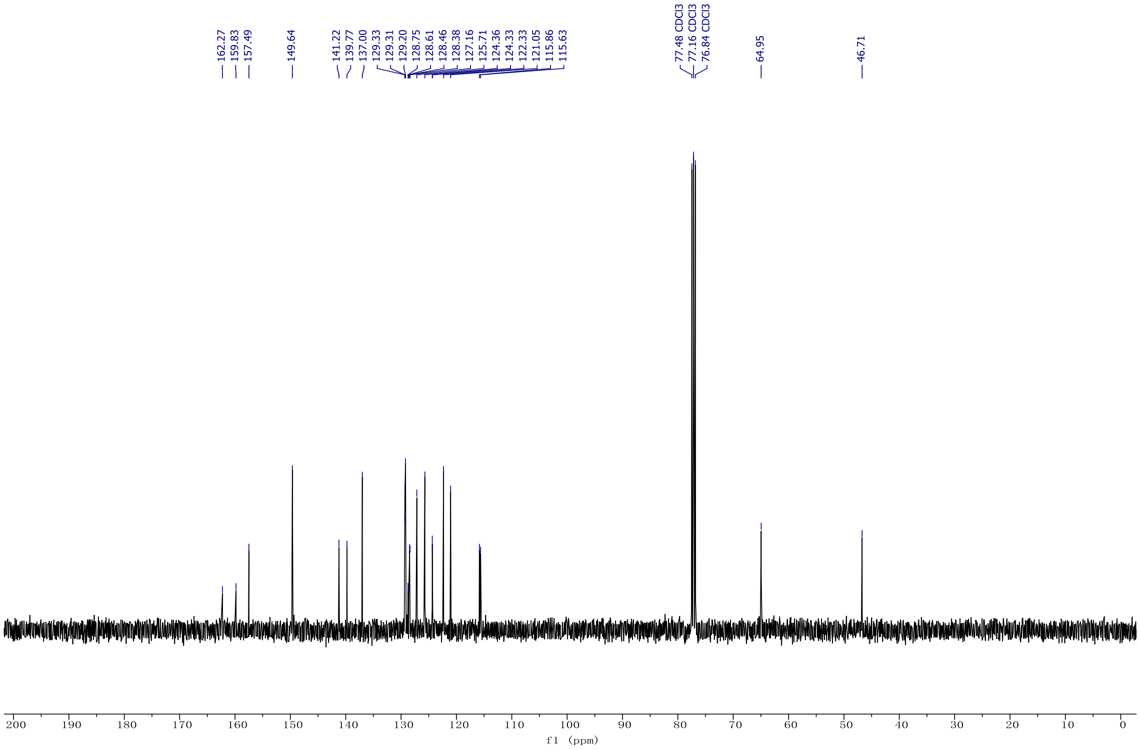

Supplementary Figure 99. ^{13}C NMR spectrum (101 MHz, $CDCl_3$, at rt) of **3aa**

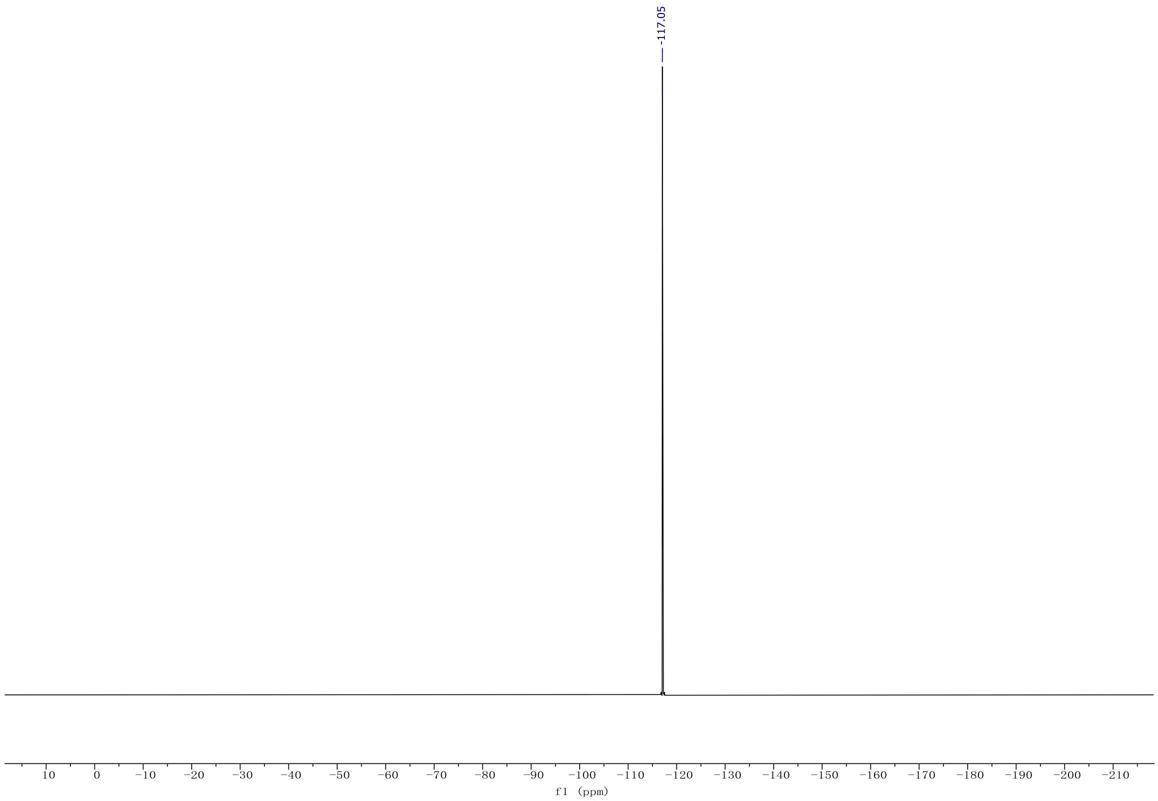

Supplementary Figure 100. ^{19}F NMR spectrum (376 MHz, $CDCl_3$, at rt) of **3aa**

2-(3-Methoxyphenyl)-2-(3-(pyridin-2-yl)phenyl)ethan-1-ol **3ab**

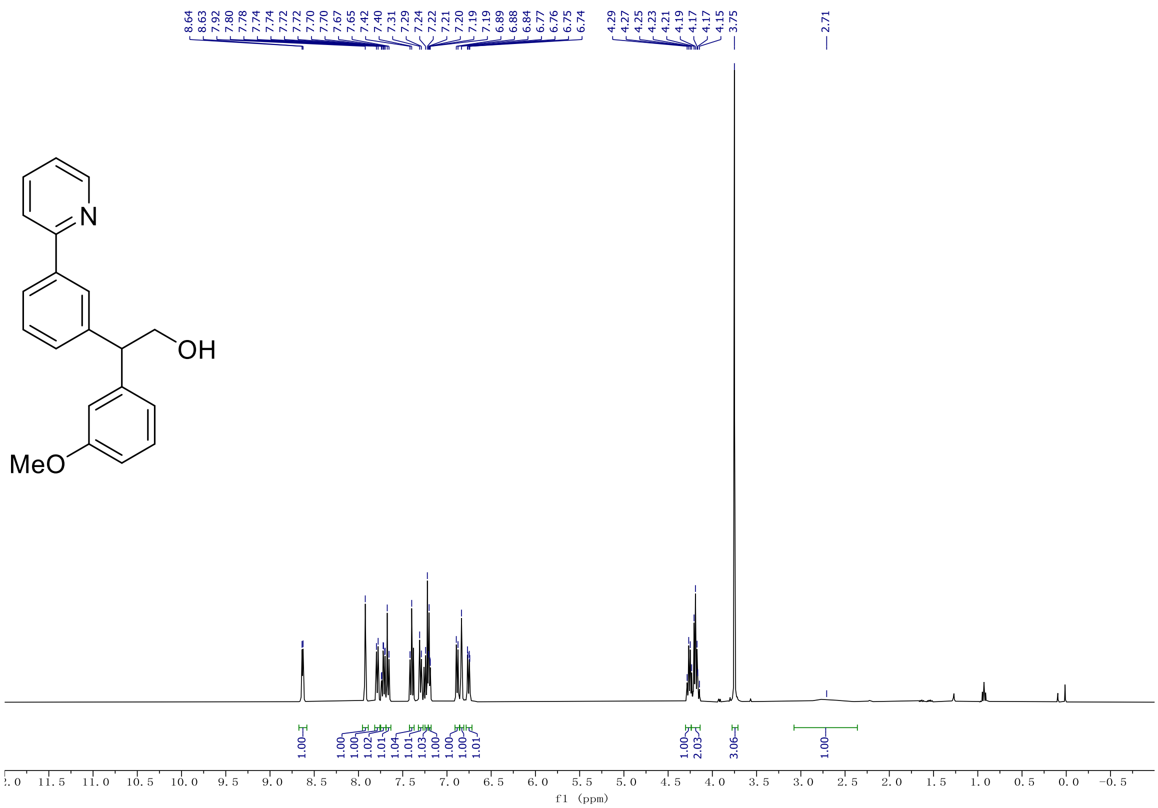

Supplementary Figure 101. ^{1}H NMR spectrum (400 MHz, $CDCl_3$, at rt) of **3ab**

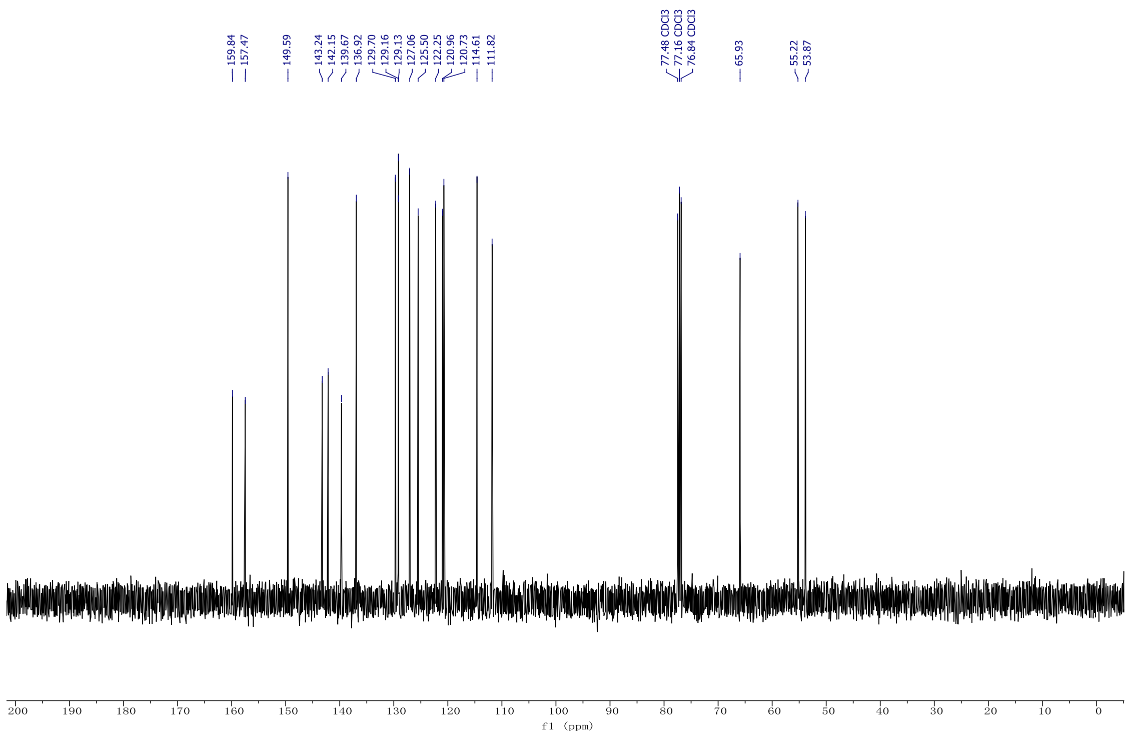

Supplementary Figure 102. ^{13}C NMR spectrum (101 MHz, $CDCl_3$, at rt) of **3ab**

2-(3-Bromophenyl)-2-(3-(pyridin-2-yl)phenyl)ethan-1-ol **3ac**

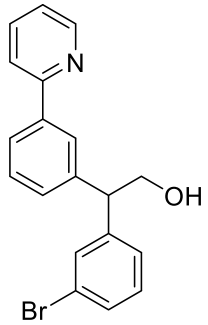

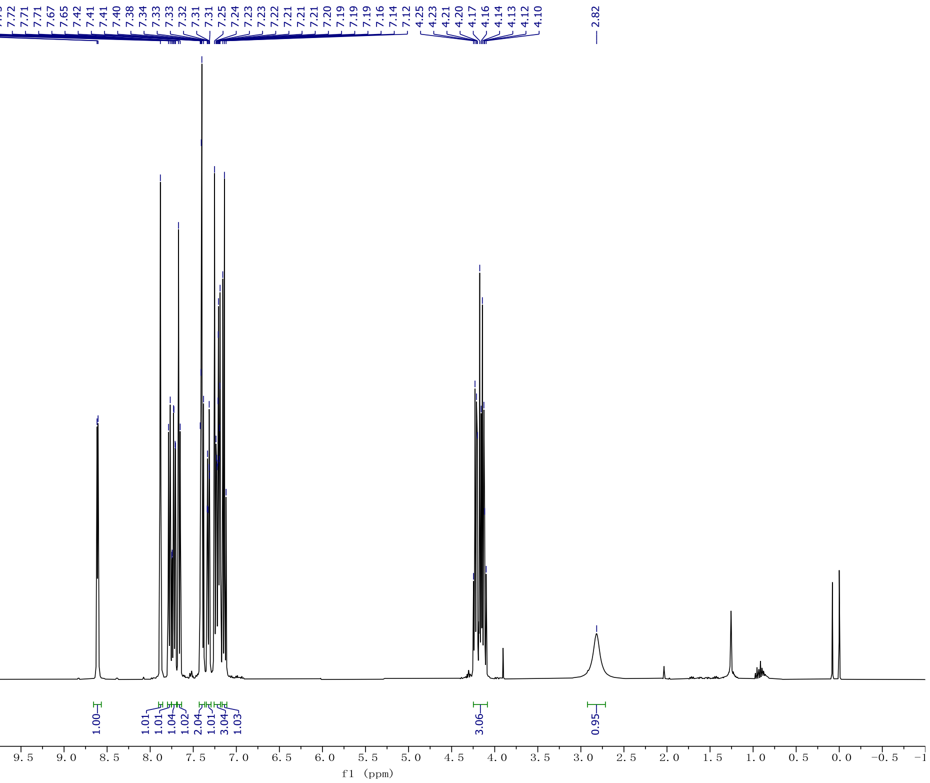

Supplementary Figure 103. ^{1}H NMR spectrum (400 MHz, $CDCl_3$, at rt) of **3ac**

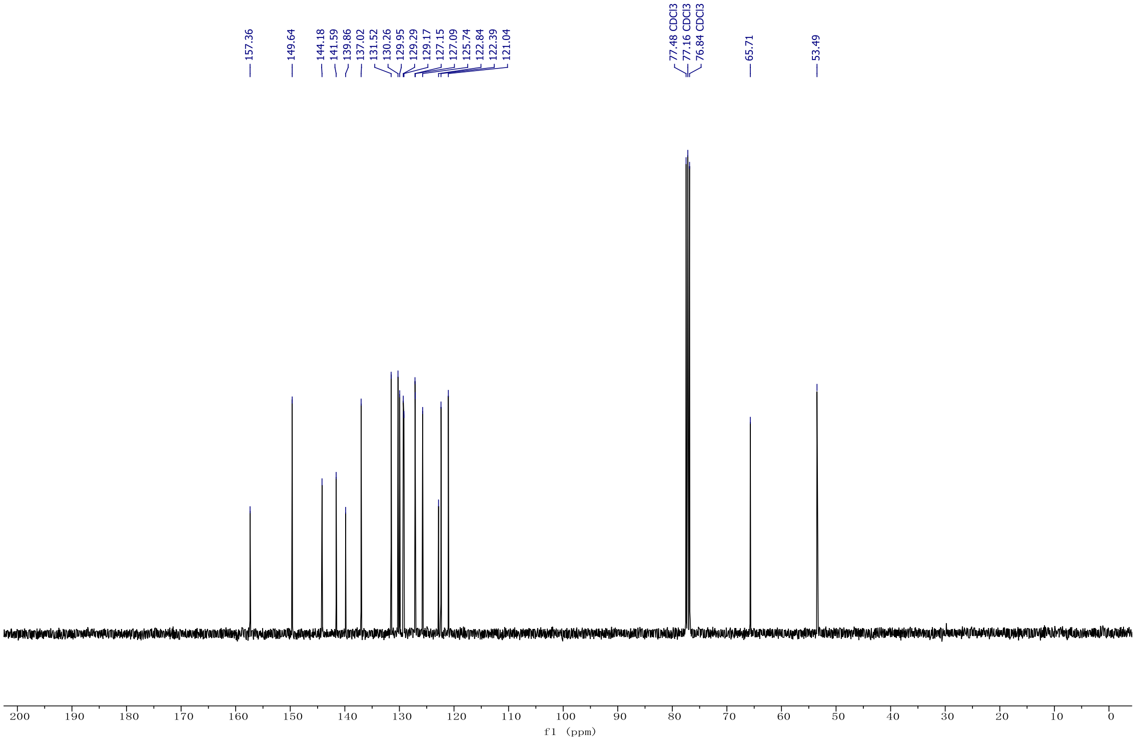

Supplementary Figure 104. ^{13}C NMR spectrum (101 MHz, $CDCl_3$, at rt) of **3ac**

2-(3-Chlorophenyl)-2-(3-(pyridin-2-yl)phenyl)ethan-1-ol **3ad**

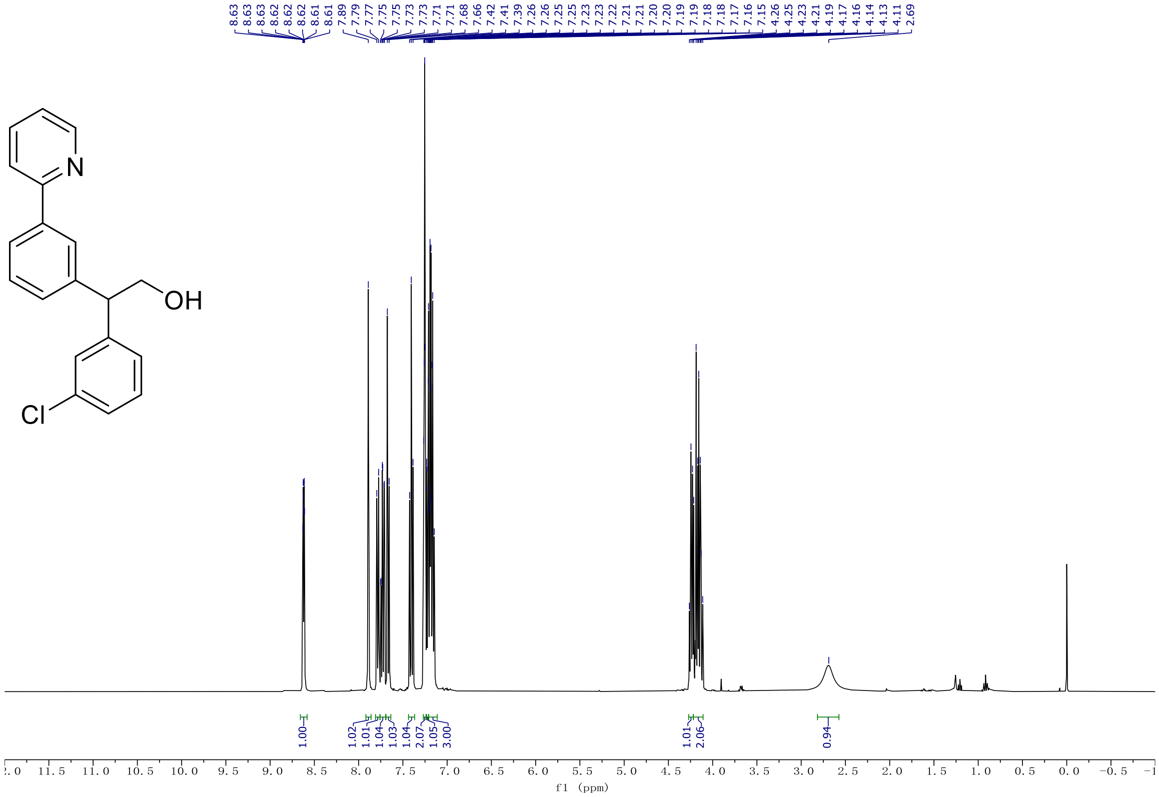

Supplementary Figure 105. ^{1}H NMR spectrum (400 MHz, $CDCl_3$, at rt) of **3ad**

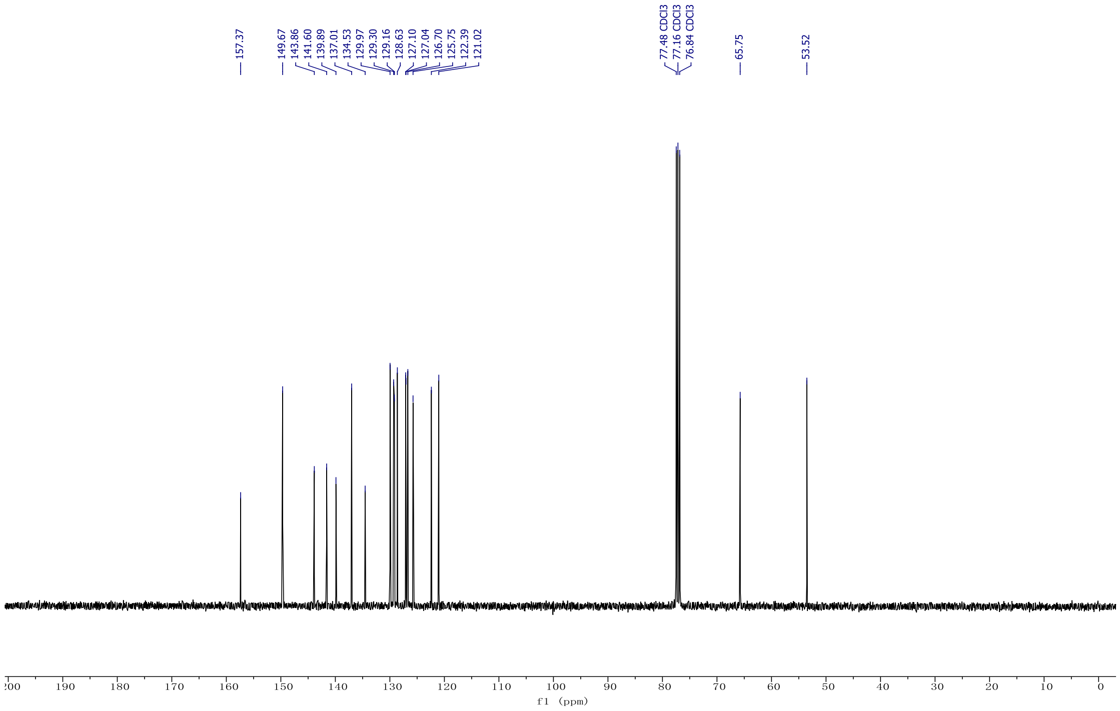

Supplementary Figure 106. ^{13}C NMR spectrum (101 MHz, $CDCl_3$, at rt) of **3ad**

5-(2-Hydroxy-1-(3-(pyridin-2-yl)phenyl)ethyl)-2,3-dihydro-1*H*-inden-1-one **3ae**

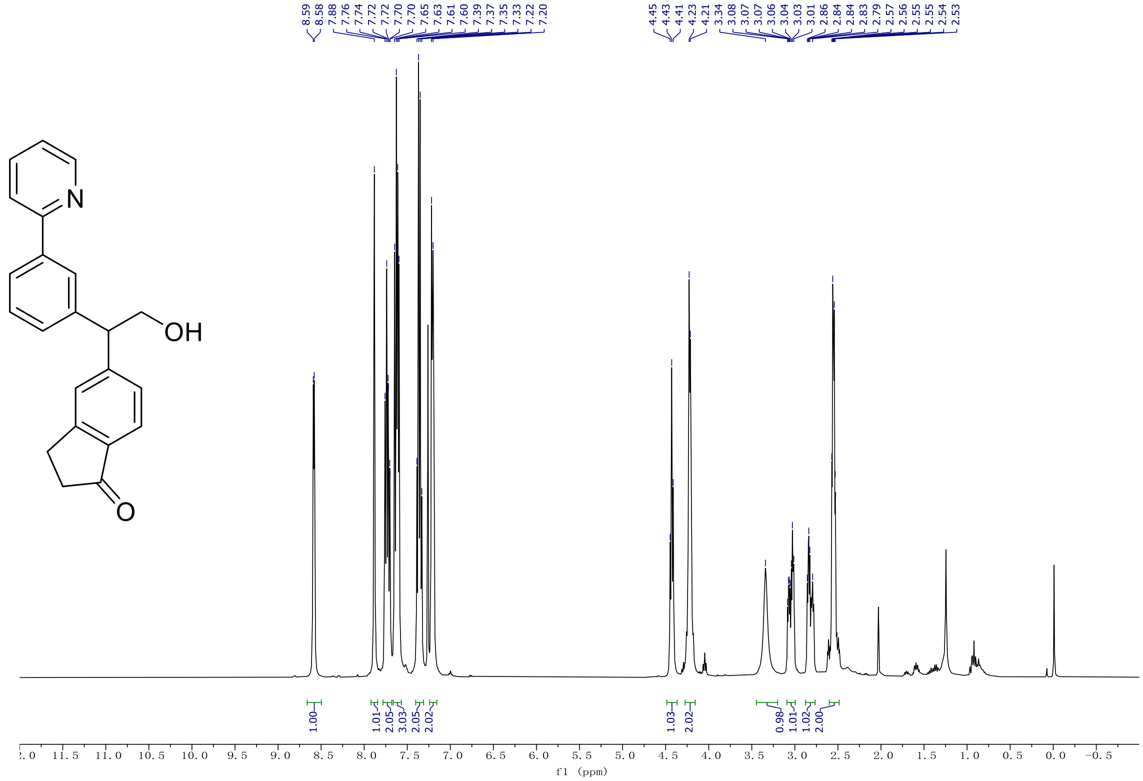

Supplementary Figure 107. ^{1}H NMR spectrum (400 MHz, $CDCl_3$, at rt) of **3ae**

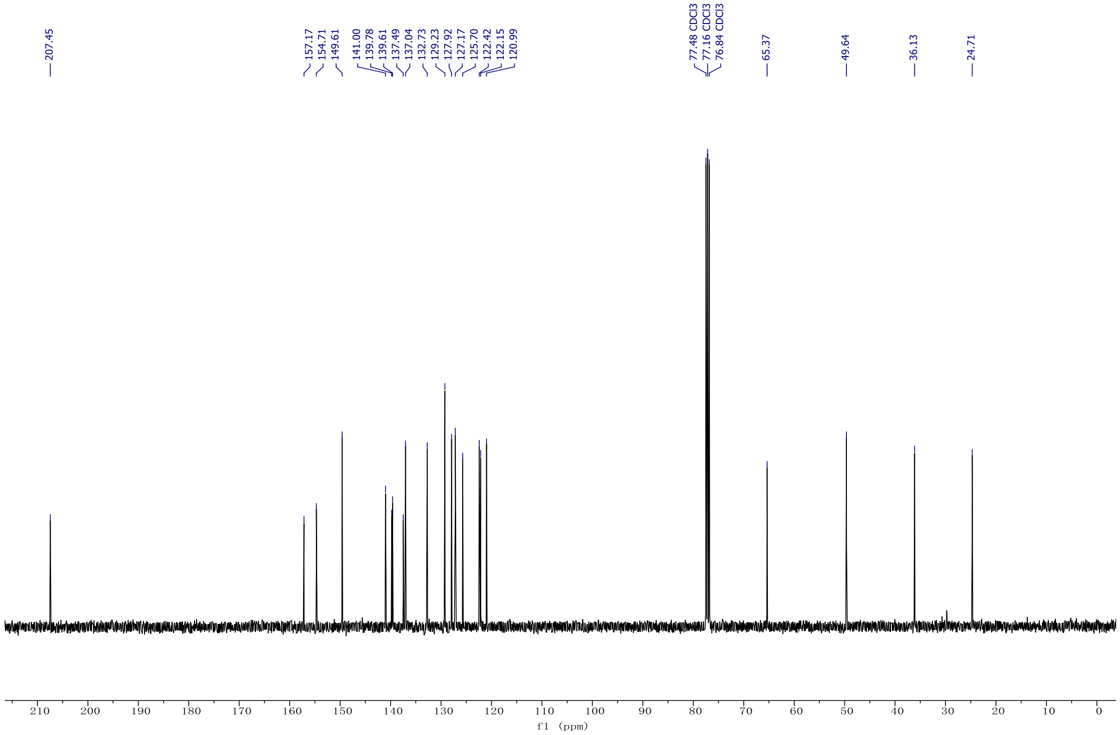

Supplementary Figure 108. ^{13}C NMR spectrum (101 MHz, $CDCl_3$, at rt) of **3ae**

2-(Naphthalen-1-yl)-2-(3-(pyridin-2-yl)phenyl)ethan-1-ol **3af**

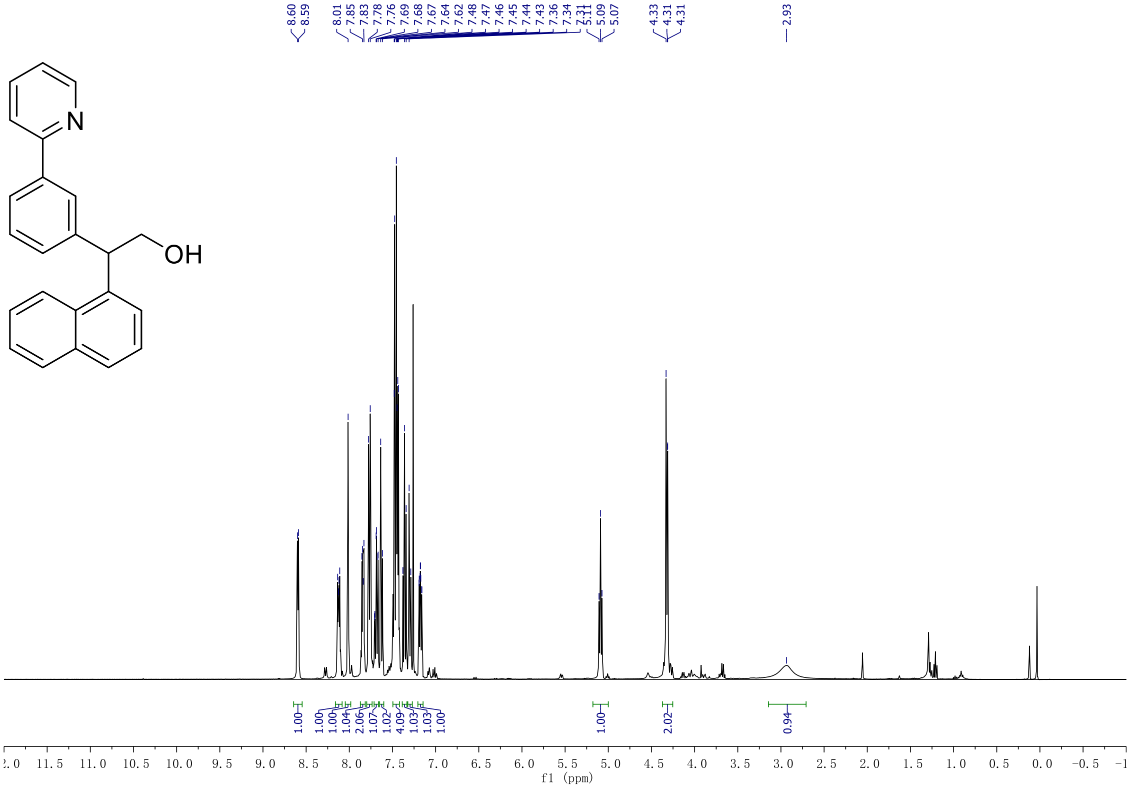

Supplementary Figure 109. ^{1}H NMR spectrum (400 MHz, $CDCl_3$, at rt) of **3af**

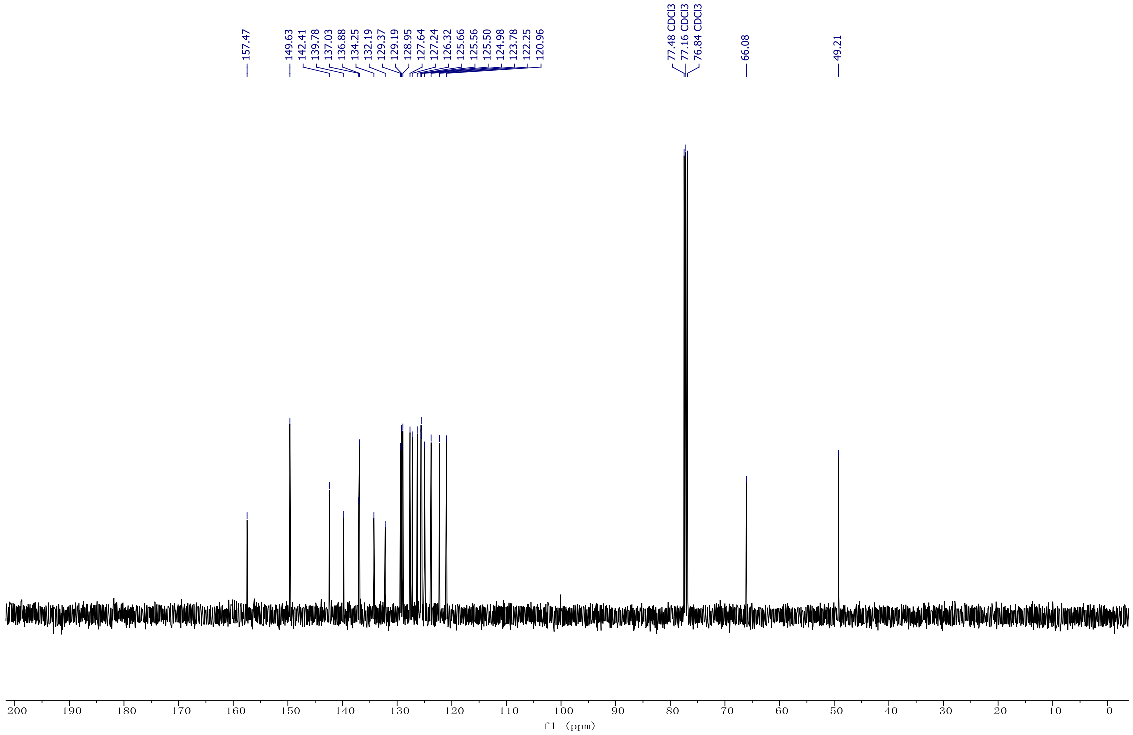

Supplementary Figure 110. ^{13}C NMR spectrum (101 MHz, $CDCl_3$, at rt) of **3af**

2-([1,1'-Binaphthalen]-2-yl)-2-(3-(pyridin-2-yl)phenyl)ethan-1-ol **3ag** (two diastereomers mixed, 1:1 ratio)

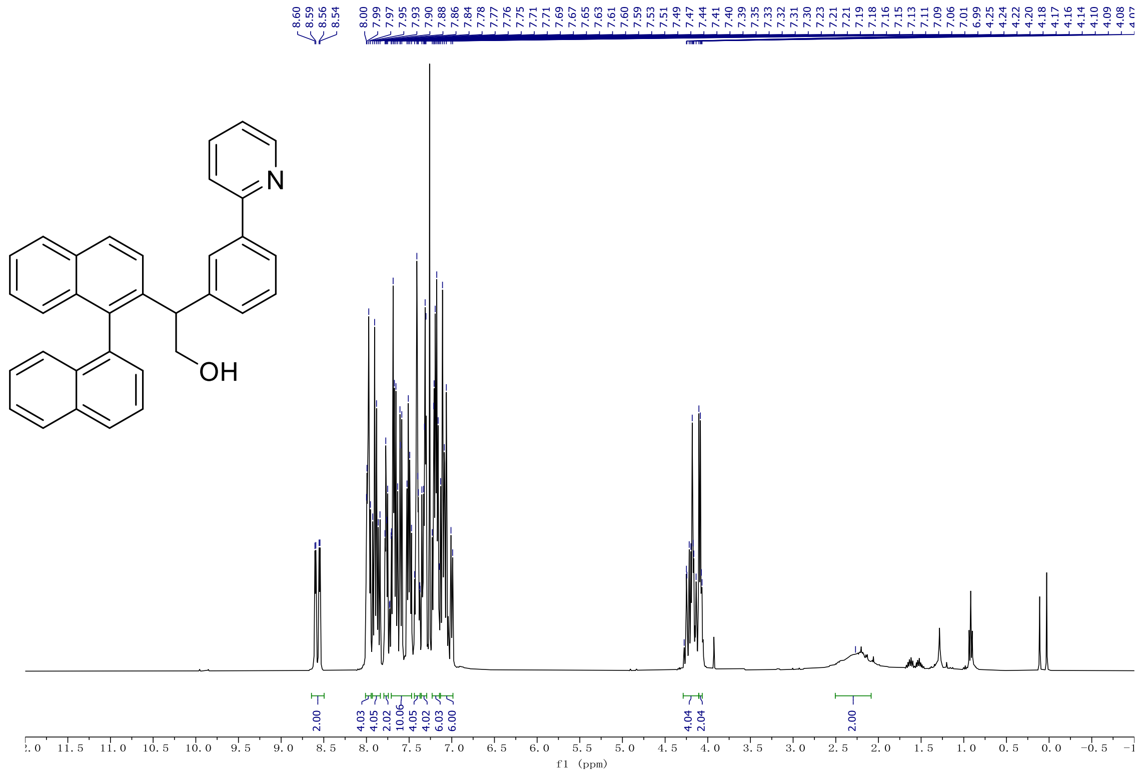

Supplementary Figure 111. ^{1}H NMR spectrum (400 MHz, $CDCl_3$, at rt) of **3ag**

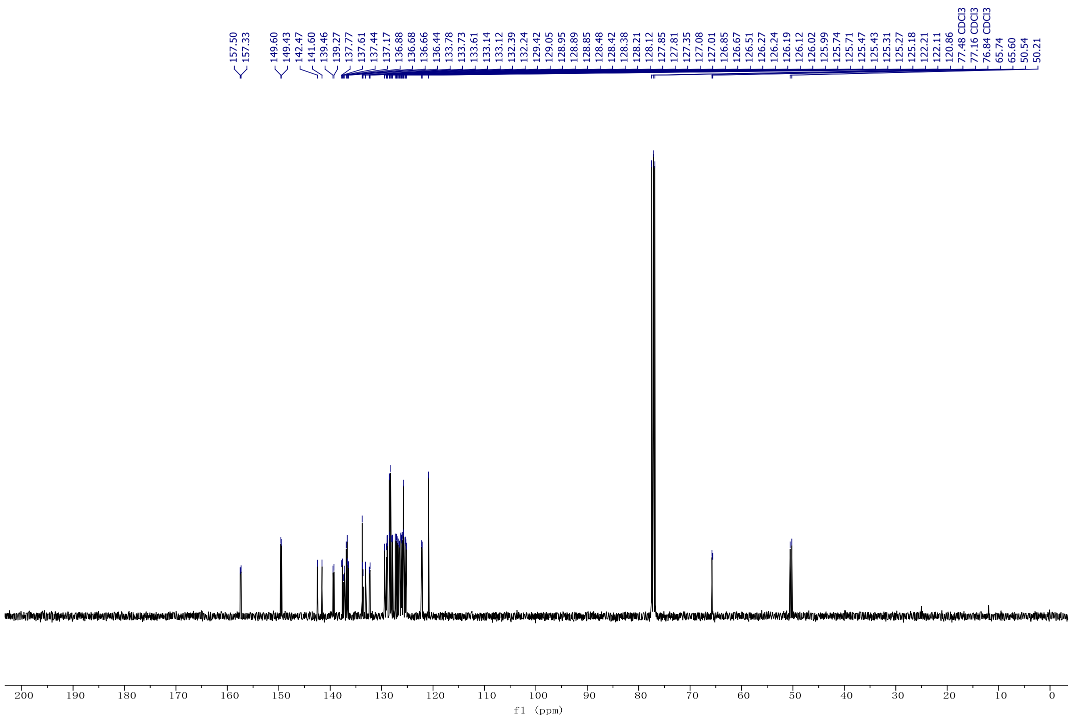

Supplementary Figure 112. ^{13}C NMR spectrum (101 MHz, $CDCl_3$, at rt) of **3ag**

2-(Furan-3-yl)-2-(3-(pyridin-2-yl)phenyl)ethan-1-ol **3ah**

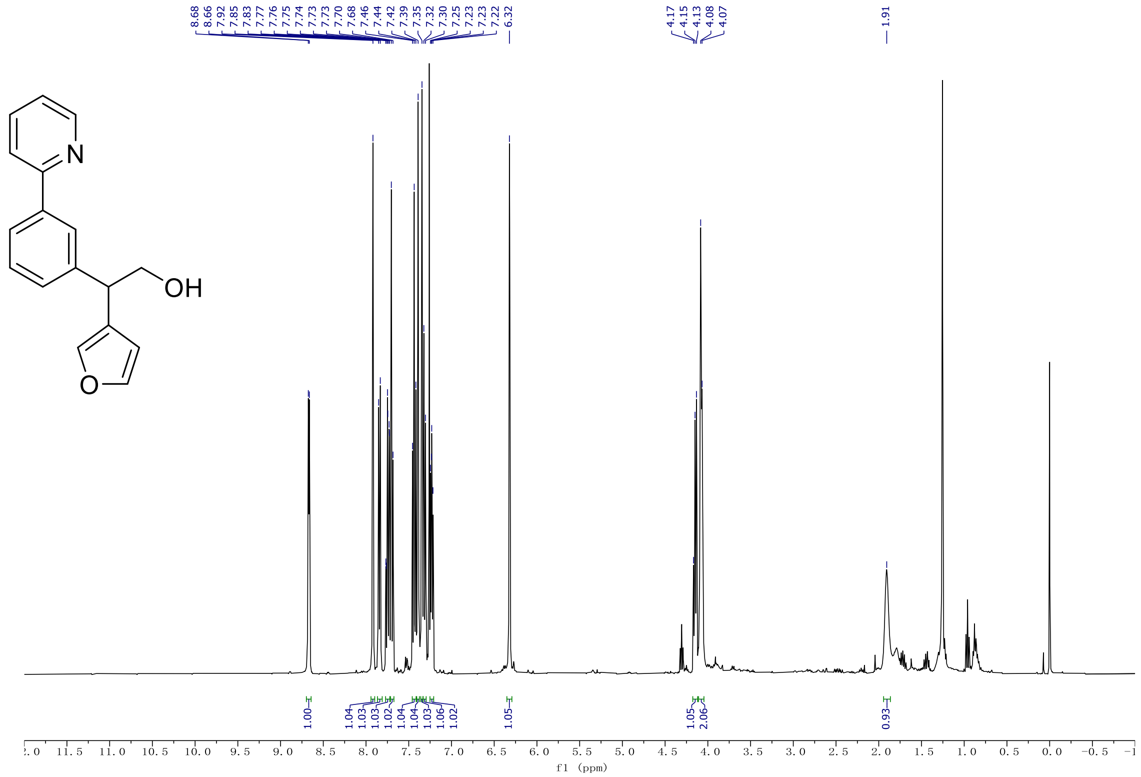

Supplementary Figure 113. ^{1}H NMR spectrum (400 MHz, $CDCl_3$, at rt) of **3ah**

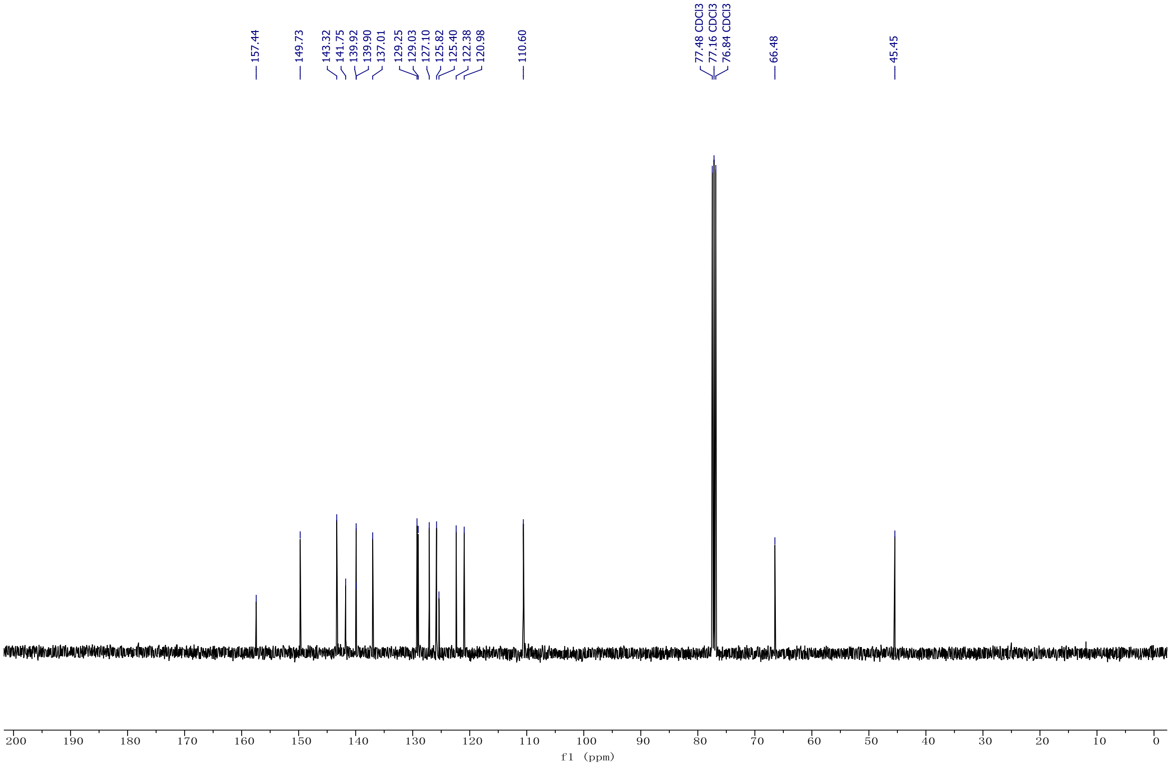

Supplementary Figure 114. ^{13}C NMR spectrum (101 MHz, $CDCl_3$, at rt) of **3ah**

2-(3-(Pyridin-2-yl)phenyl)-2-(thiophen-3-yl)ethan-1-ol **3ai**

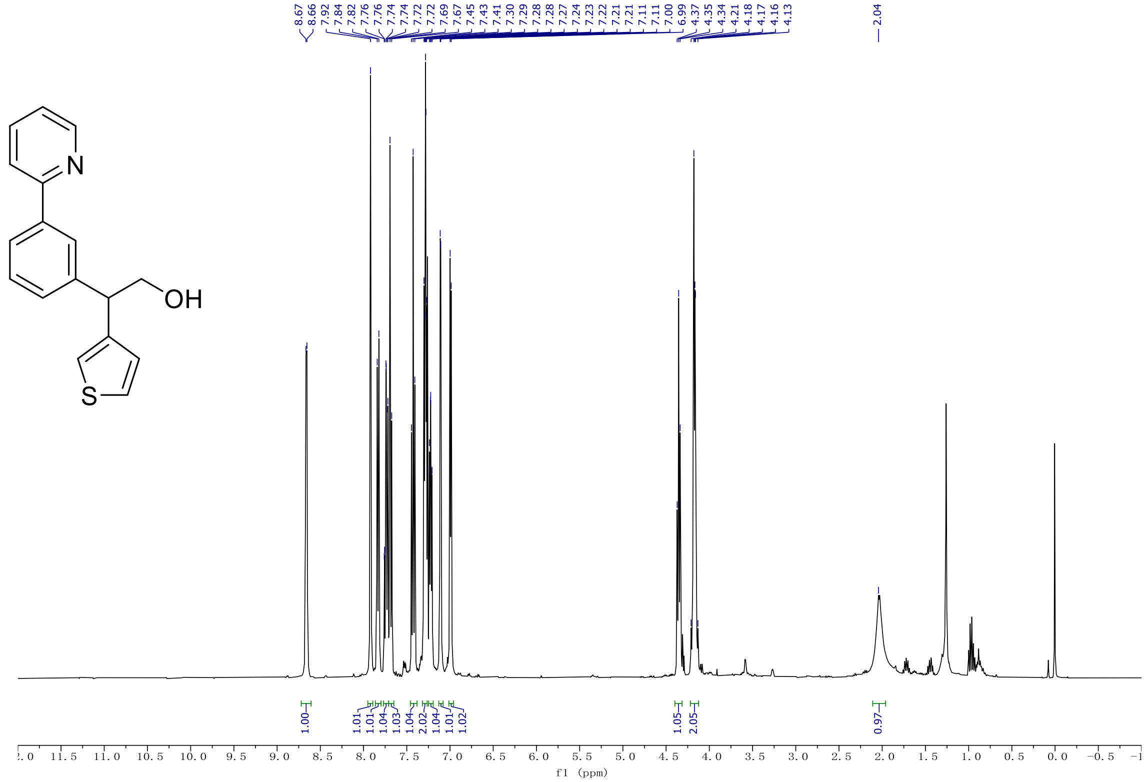

Supplementary Figure 115. ^{1}H NMR spectrum (400 MHz, $CDCl_3$, at rt) of **3ai**

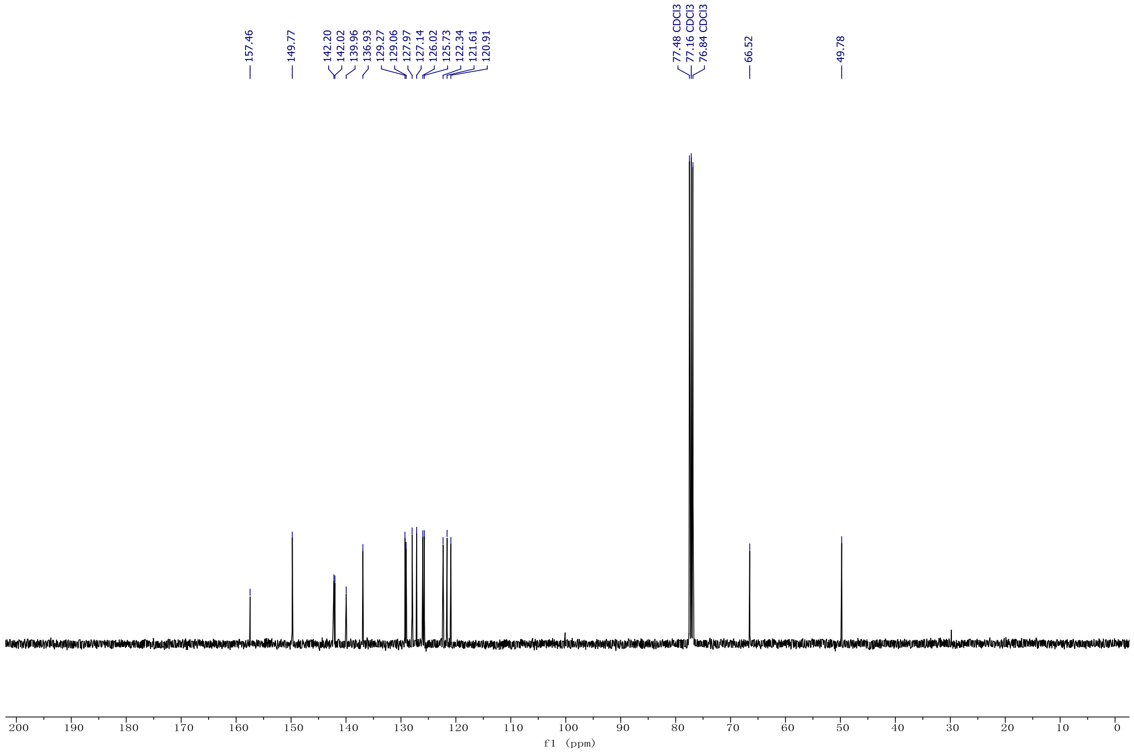

Supplementary Figure 116. ^{13}C NMR spectrum (101 MHz, $CDCl_3$, at rt) of **3ai**

2-(3-(Pyridin-2-yl)phenyl)-2-(quinolin-6-yl)ethan-1-ol **3aj**

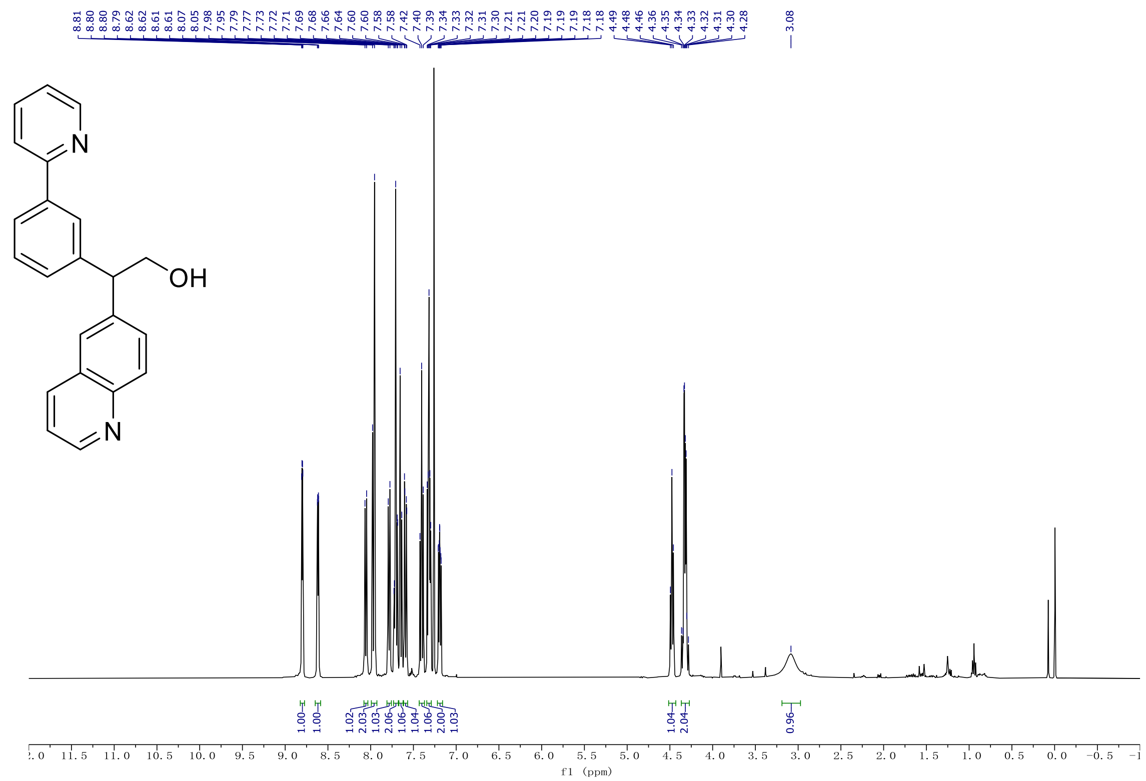

Supplementary Figure 117. ^{1}H NMR spectrum (400 MHz, $CDCl_3$, at rt) of **3aj**

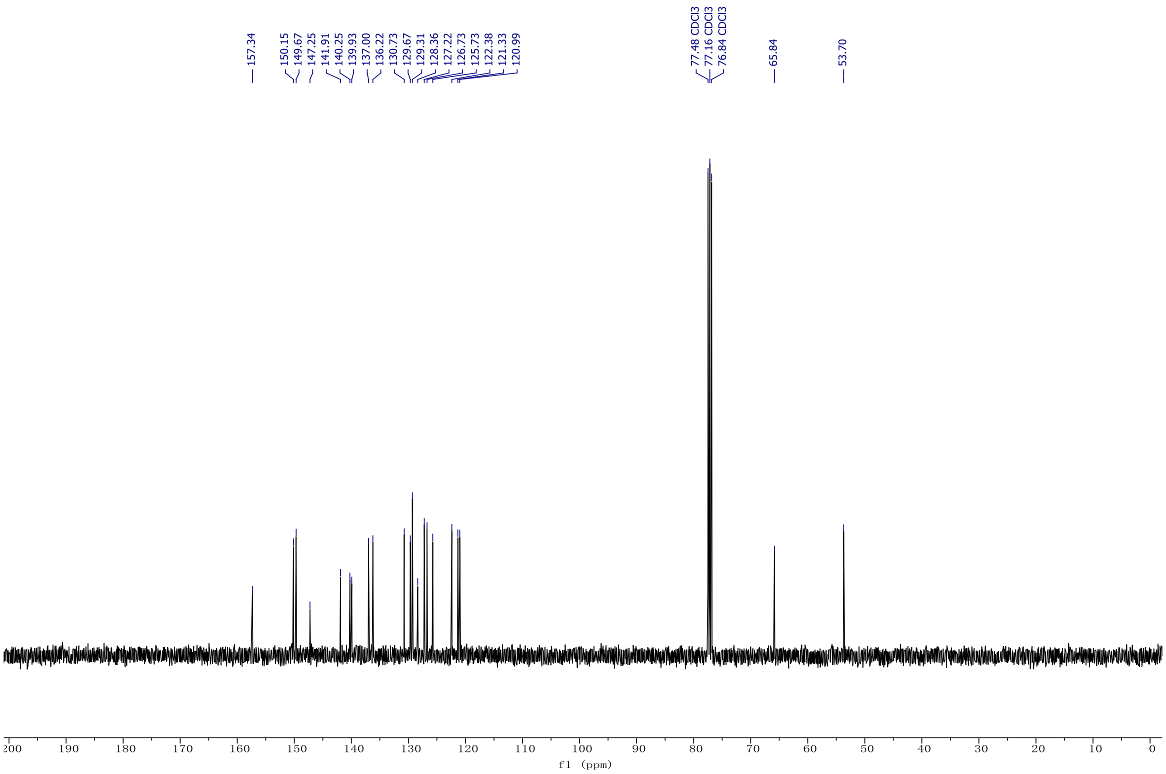

Supplementary Figure 118. ^{13}C NMR spectrum (101 MHz, $CDCl_3$, at rt) of **3aj**

4-(3-(Pyridin-2-yl)phenyl)isochroman-1-one **3ak**

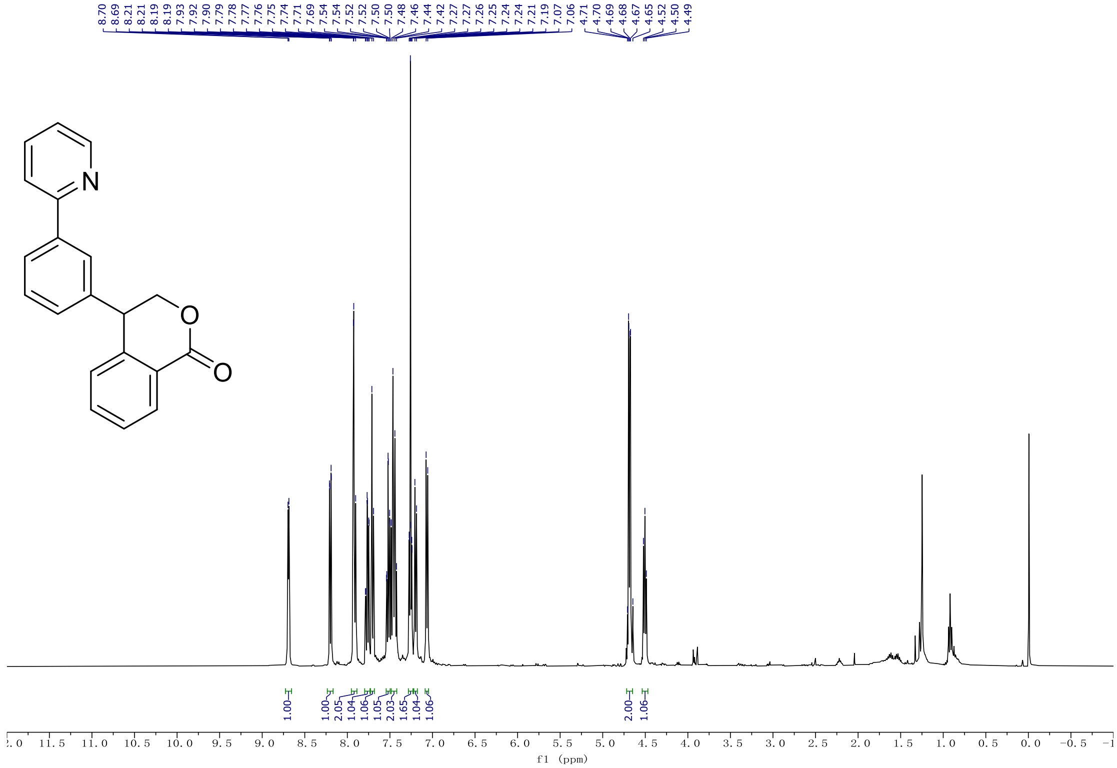

Supplementary Figure 119. ^{1}H NMR spectrum (400 MHz, $CDCl_3$, at rt) of **3ak**

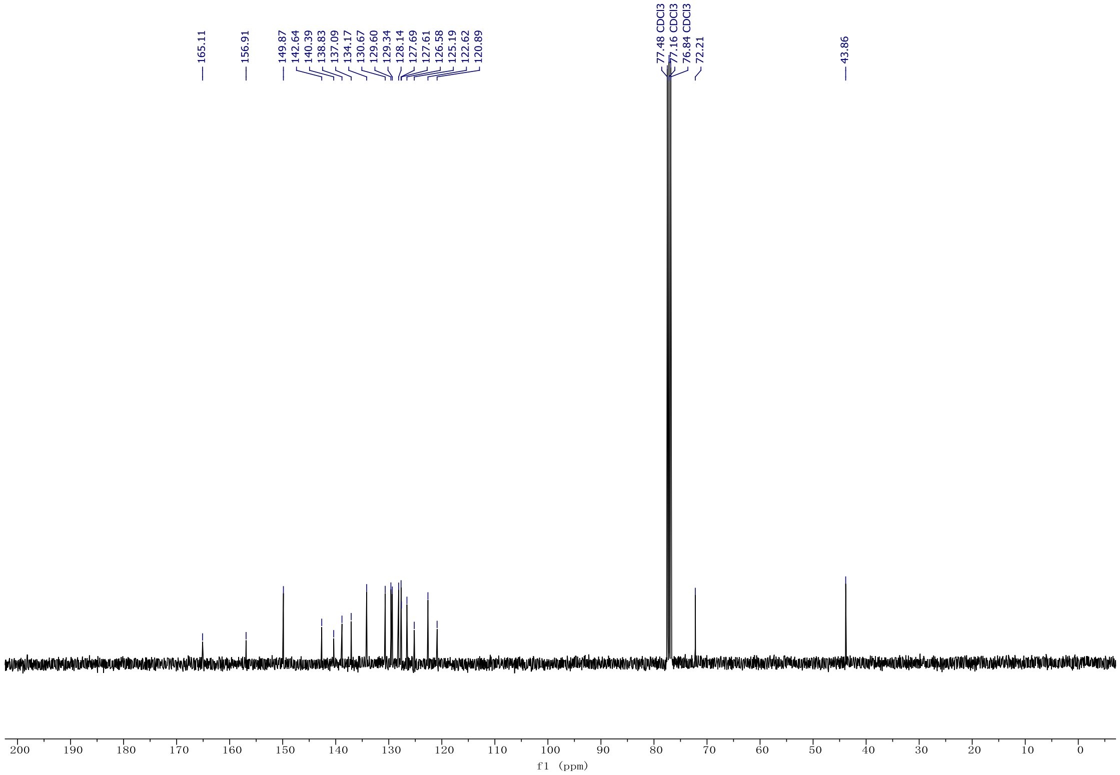

Supplementary Figure 120. ^{13}C NMR spectrum (101 MHz, $CDCl_3$, at rt) of **3ak**

1-Phenyl-1-(3-(pyridin-2-yl)phenyl)propan-2-ol **3al-major diastereoisomer**

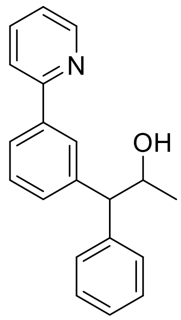

Major

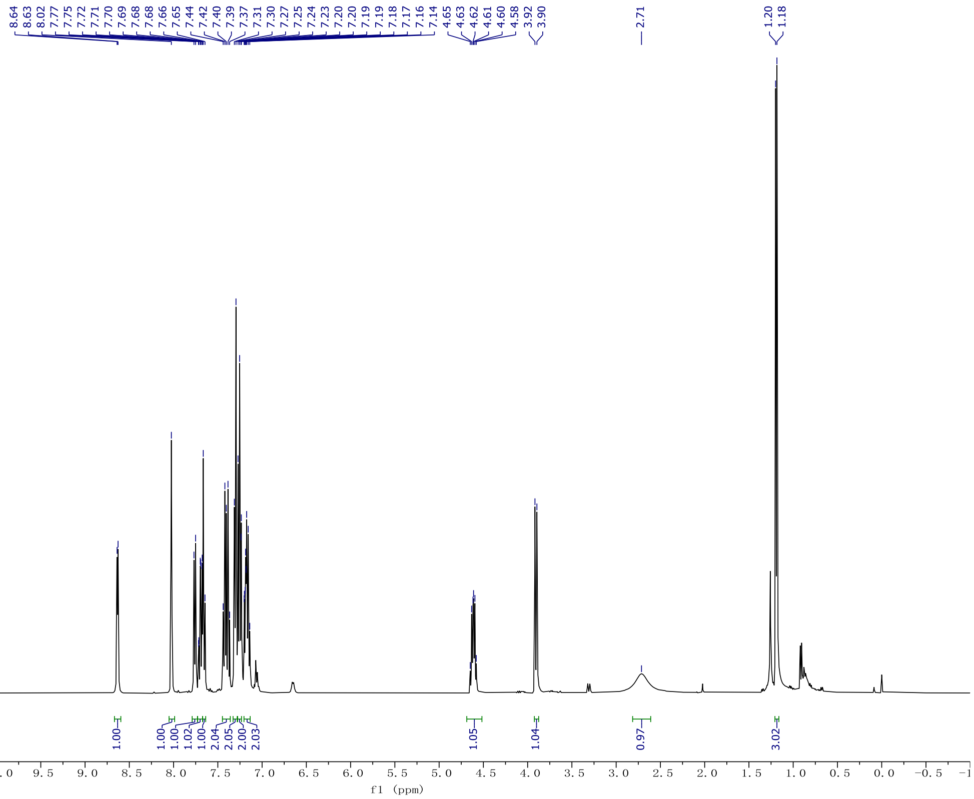

Supplementary Figure 121. ^{1}H NMR spectrum (400 MHz, $CDCl_3$, at rt) of **3al-major**

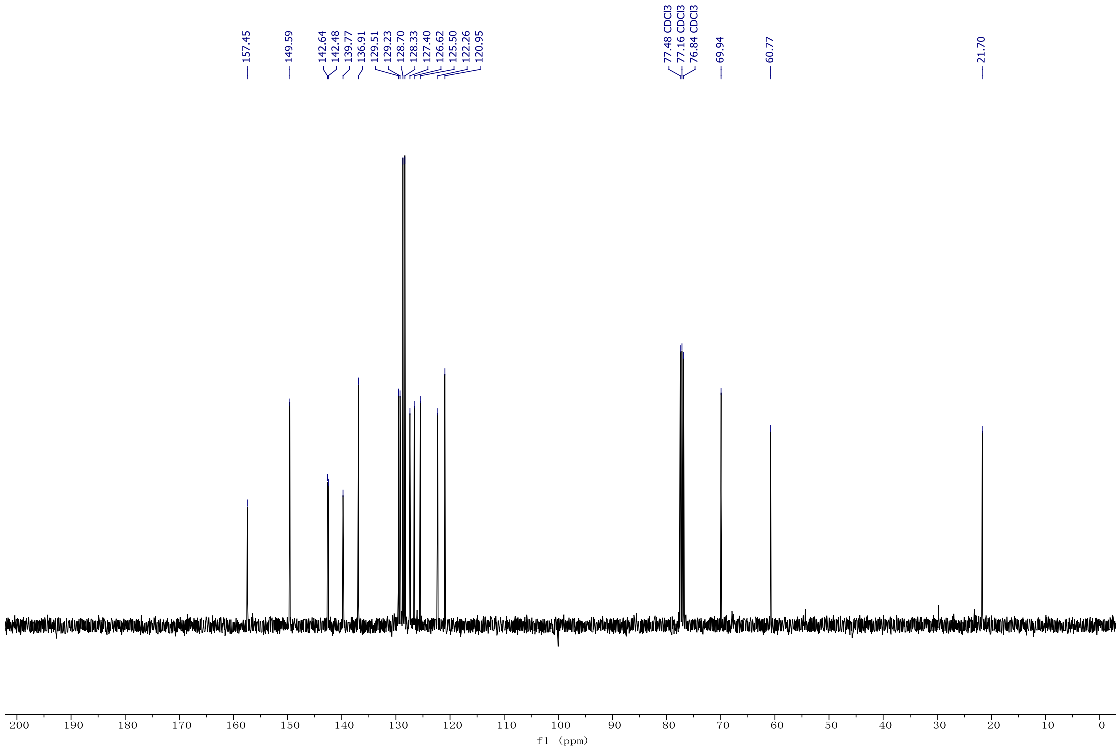

Supplementary Figure 122. ^{13}C NMR spectrum (101 MHz, $CDCl_3$, at rt) of **3al-major**

1-Phenyl-1-(3-(pyridin-2-yl)phenyl)propan-2-ol **3al-minor diastereoisomer**

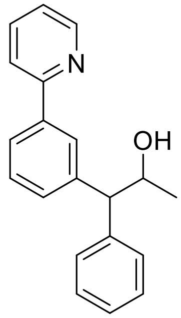

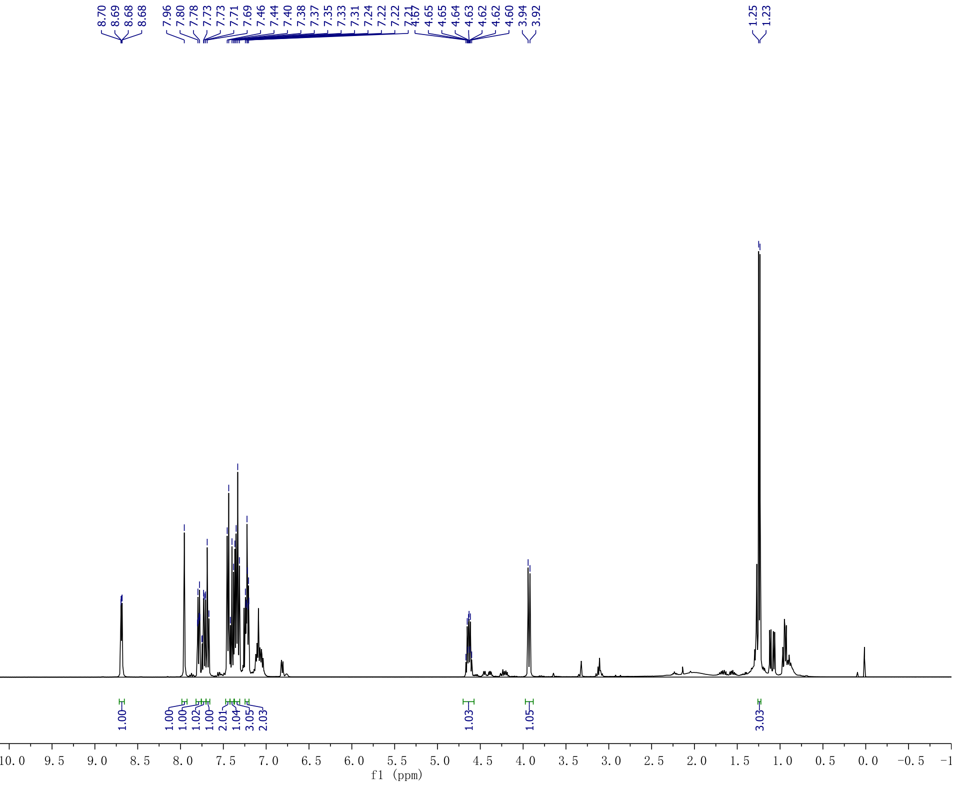

Supplementary Figure 123. ^{1}H NMR spectrum (400 MHz, $CDCl_3$, at rt) of **3al-minor**

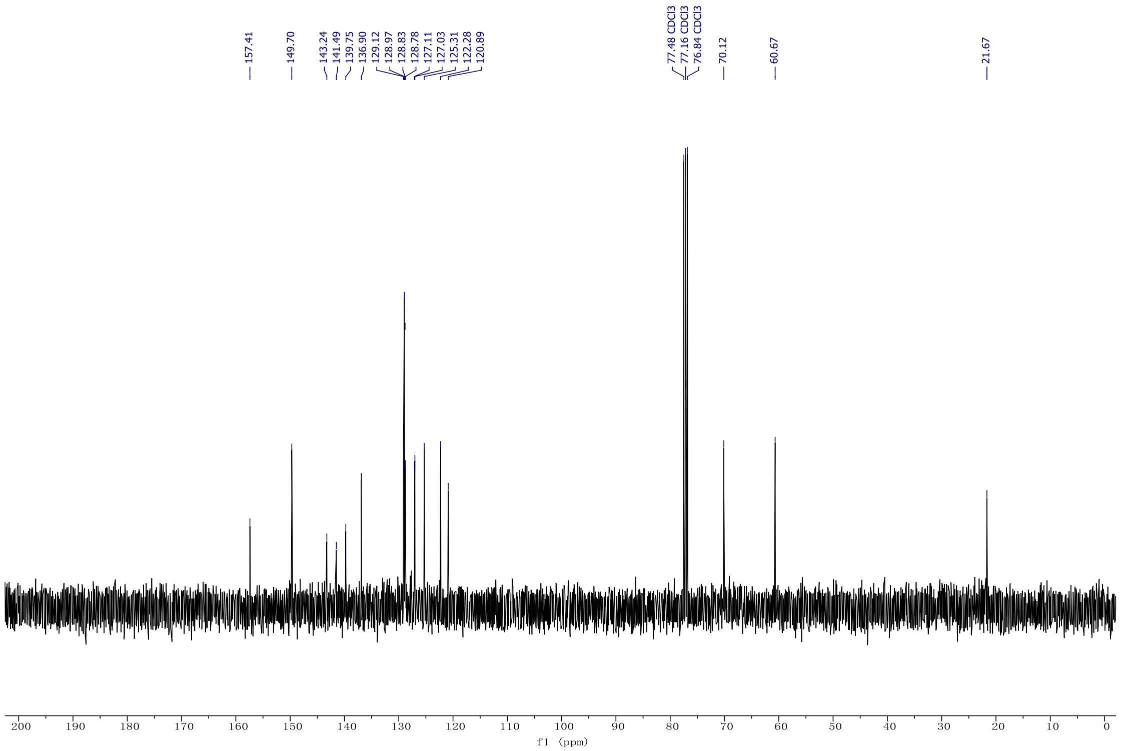

Supplementary Figure 124. ^{13}C NMR spectrum (101 MHz, $CDCl_3$, at rt) of **3al-minor**

3-Methyl-1-phenyl-1-(3-(pyridin-2-yl)phenyl)butan-2-ol **3am-major diastereoisomer**

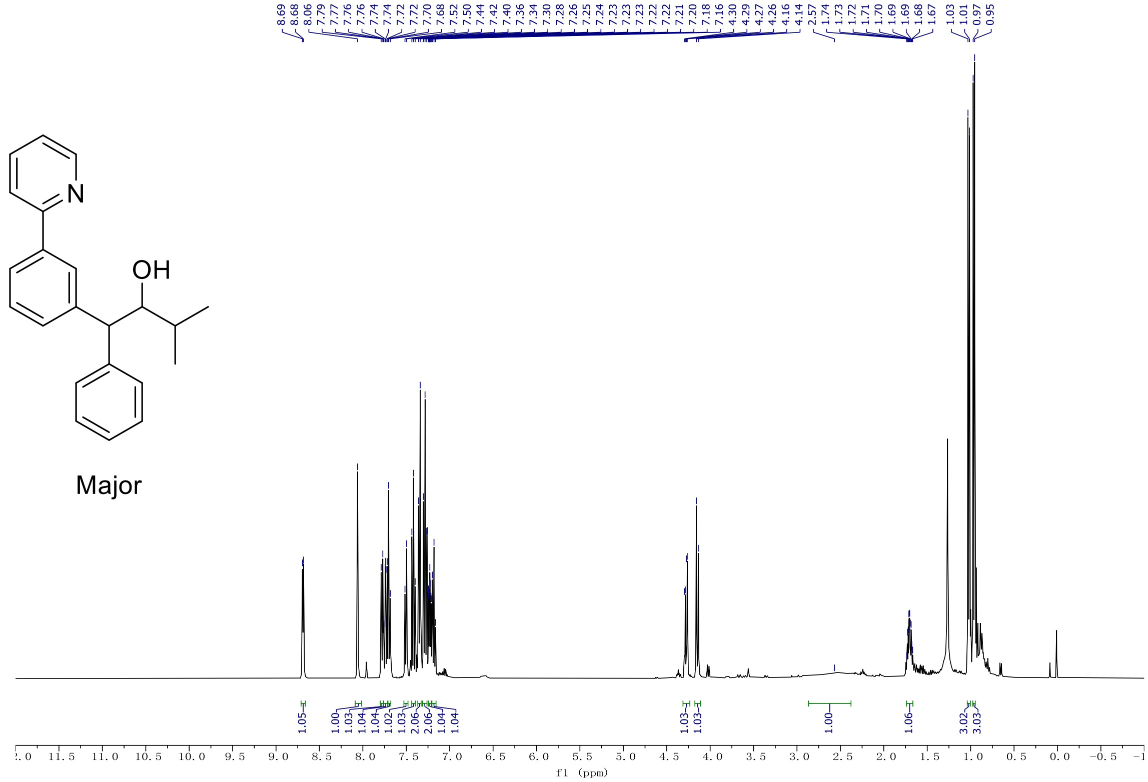

Supplementary Figure 125. ^{1}H NMR spectrum (400 MHz, $CDCl_3$, at rt) of **3am-major**

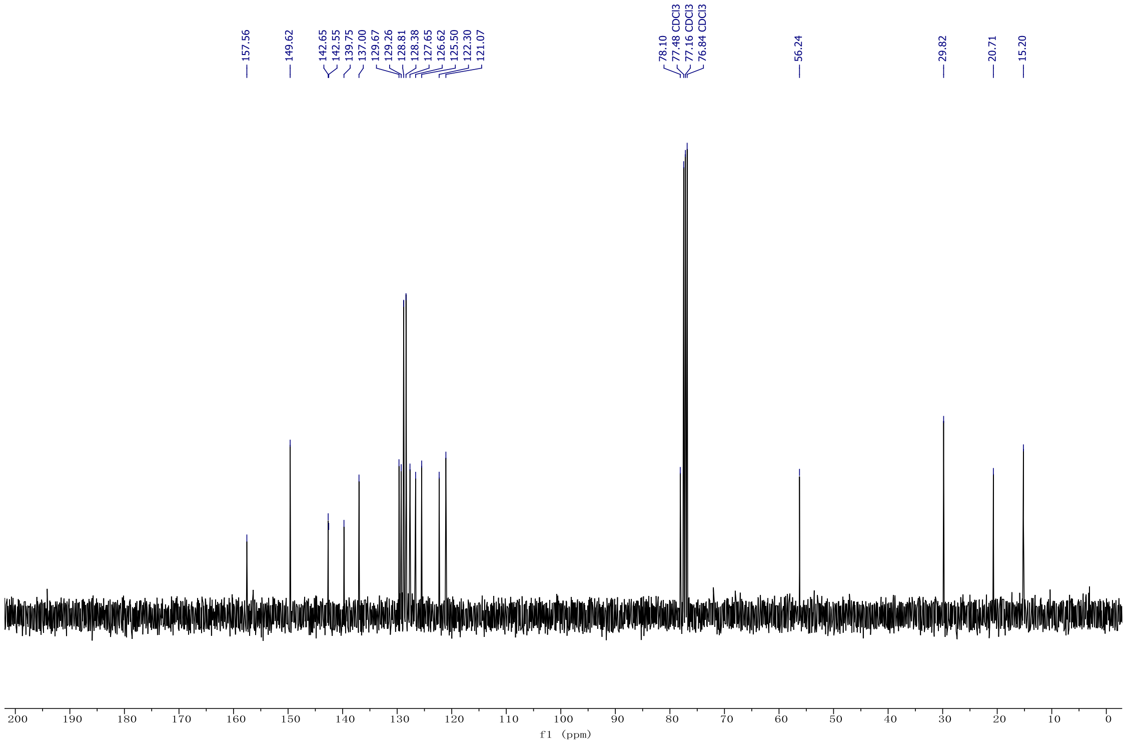

Supplementary Figure 126. ^{13}C NMR spectrum (101 MHz, $CDCl_3$, at rt) of **3am-major**

3-Methyl-1-phenyl-1-(3-(pyridin-2-yl)phenyl)butan-2-ol **3am-minor diastereoisomer**

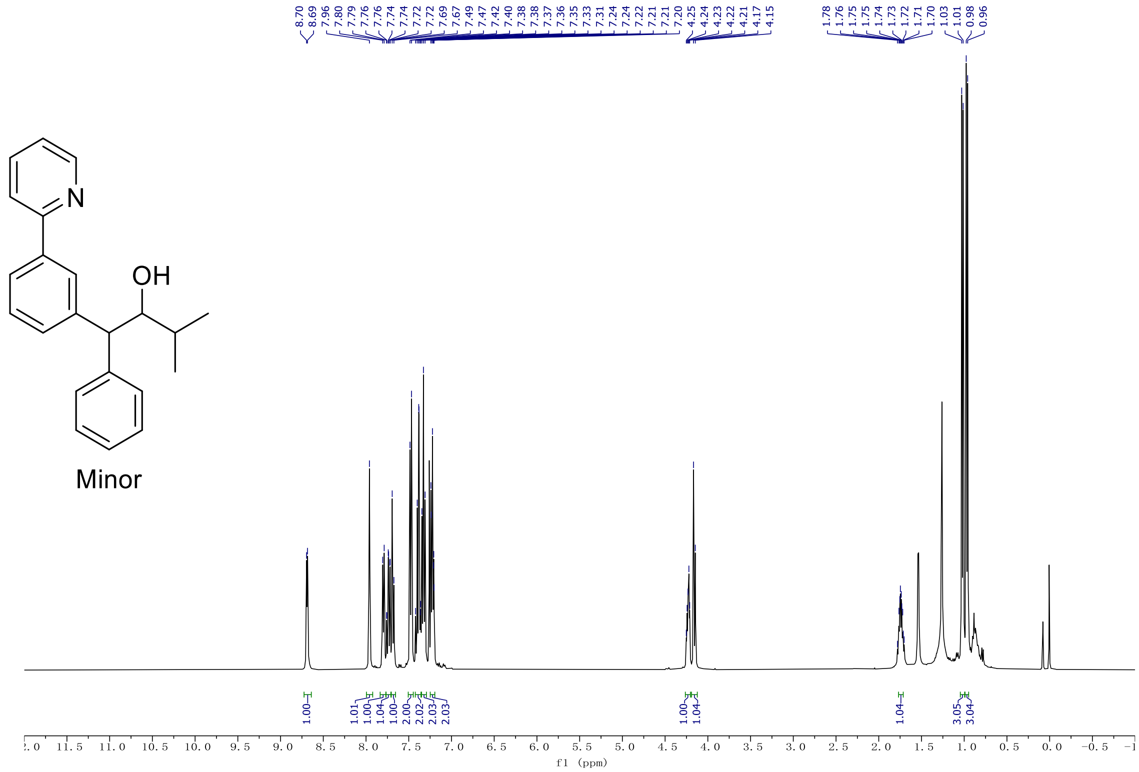

Supplementary Figure 127. ^{1}H NMR spectrum (400 MHz, $CDCl_3$, at rt) of **3am-minor**

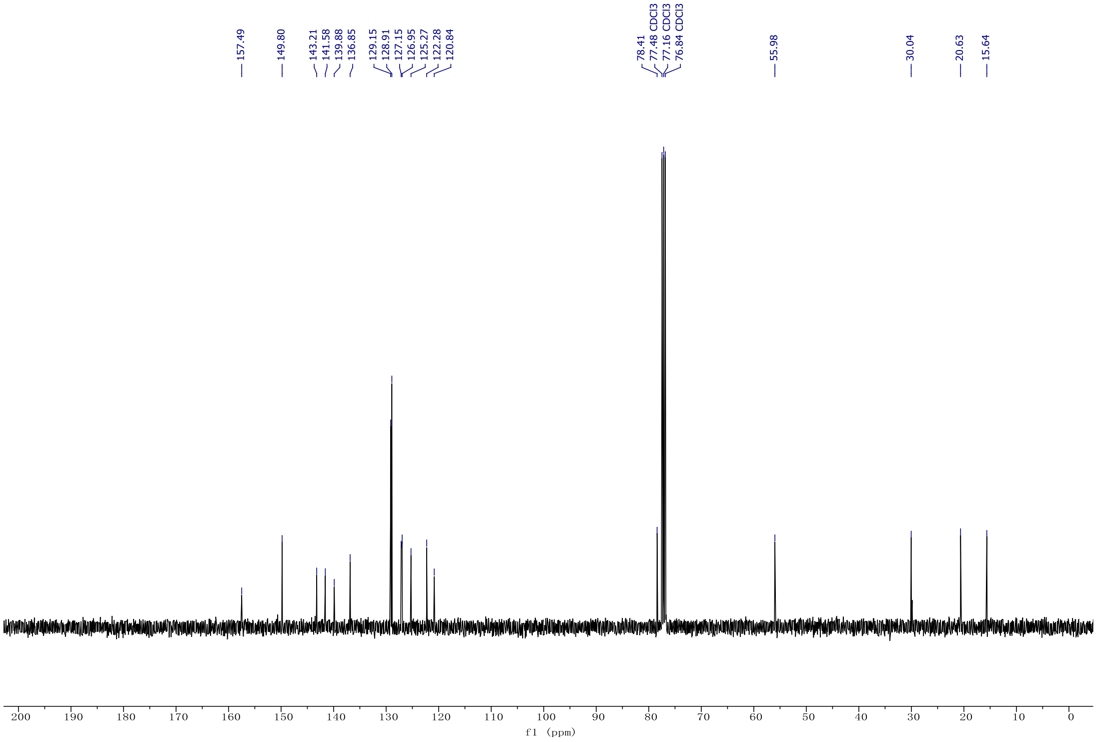

Supplementary Figure 128. ^{13}C NMR spectrum (101 MHz, $CDCl_3$, at rt) of **3am-minor**

1,2-Diphenyl-2-(3-(pyridin-2-yl)phenyl)ethan-1-ol **3an-major diastereoisomer**

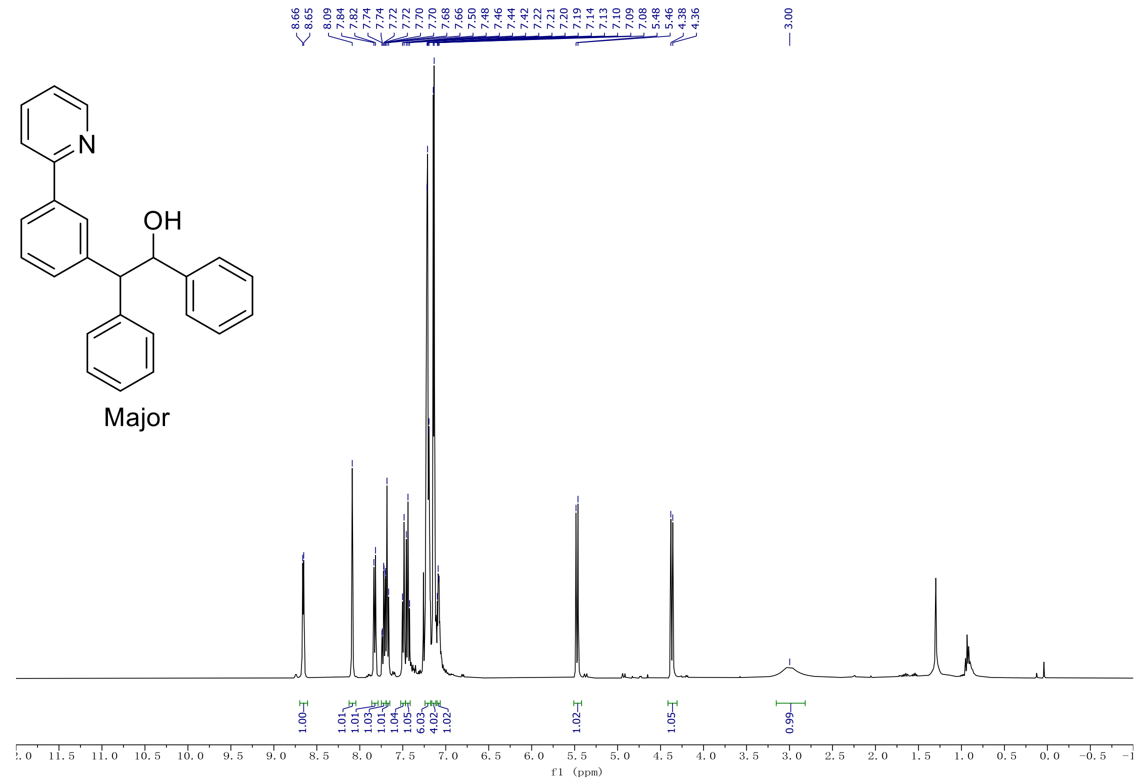

Supplementary Figure 129. ^{1}H NMR spectrum (400 MHz, $CDCl_3$, at rt) of **3an-major**

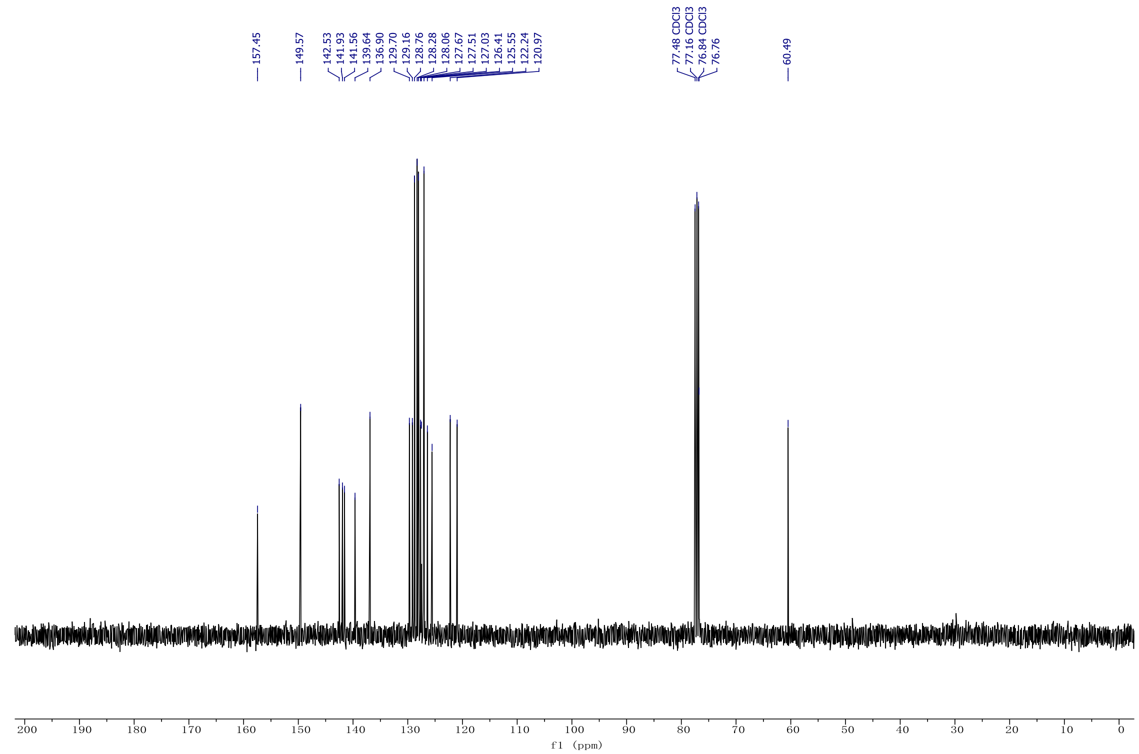

Supplementary Figure 130. ^{13}C NMR spectrum (101 MHz, $CDCl_3$, at rt) of **3an-major**

1,2-Diphenyl-2-(3-(pyridin-2-yl)phenyl)ethan-1-ol **3an-minor diastereoisomer**

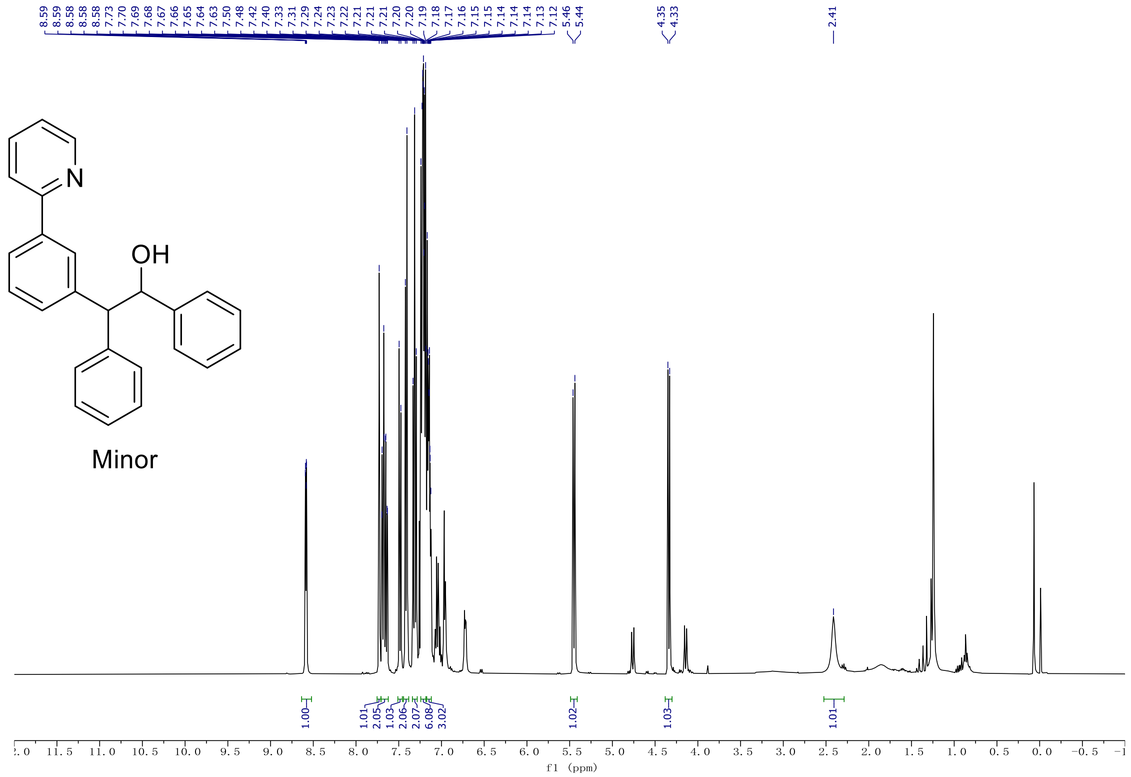

Supplementary Figure 131. ^{1}H NMR spectrum (400 MHz, $CDCl_3$, at rt) of **3an-minor**

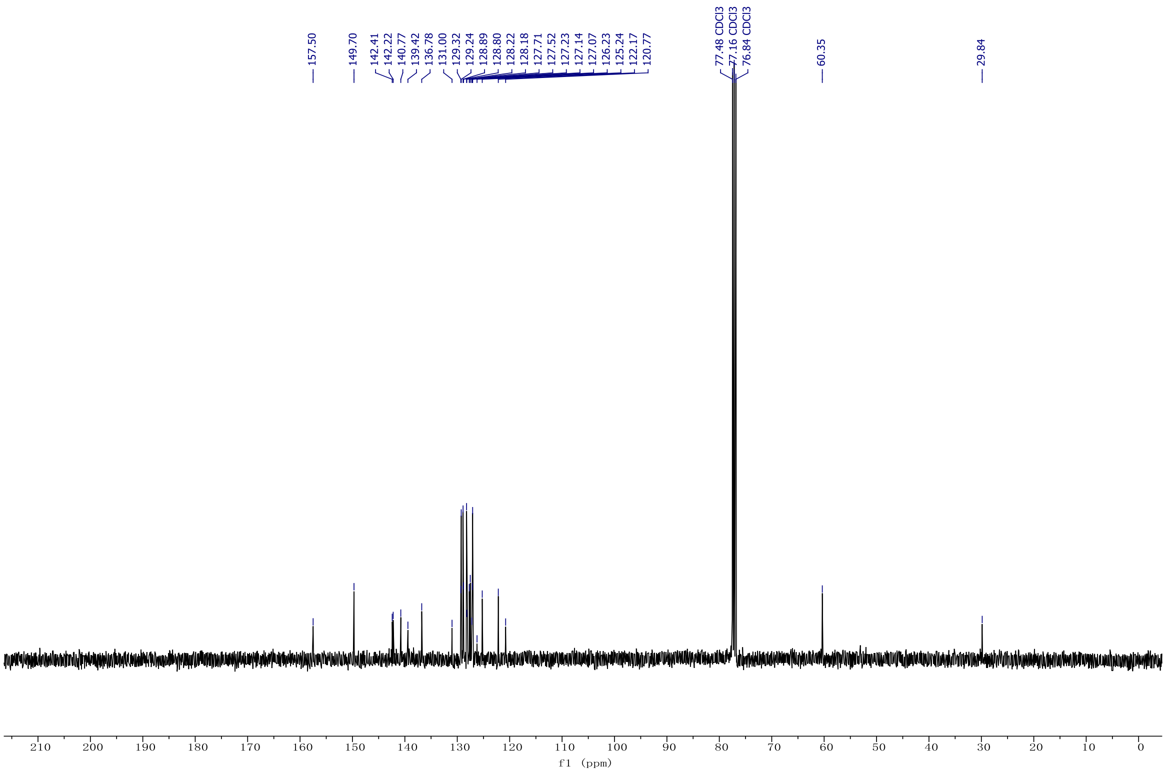

Supplementary Figure 132. ^{13}C NMR spectrum (101 MHz, $CDCl_3$, at rt) of **3an-minor**

1-(3-(Pyridin-2-yl)phenyl)-2,3-dihydro-1*H*-inden-2-ol **3ao** (two diastereomers mixed, 25:1 ratio)

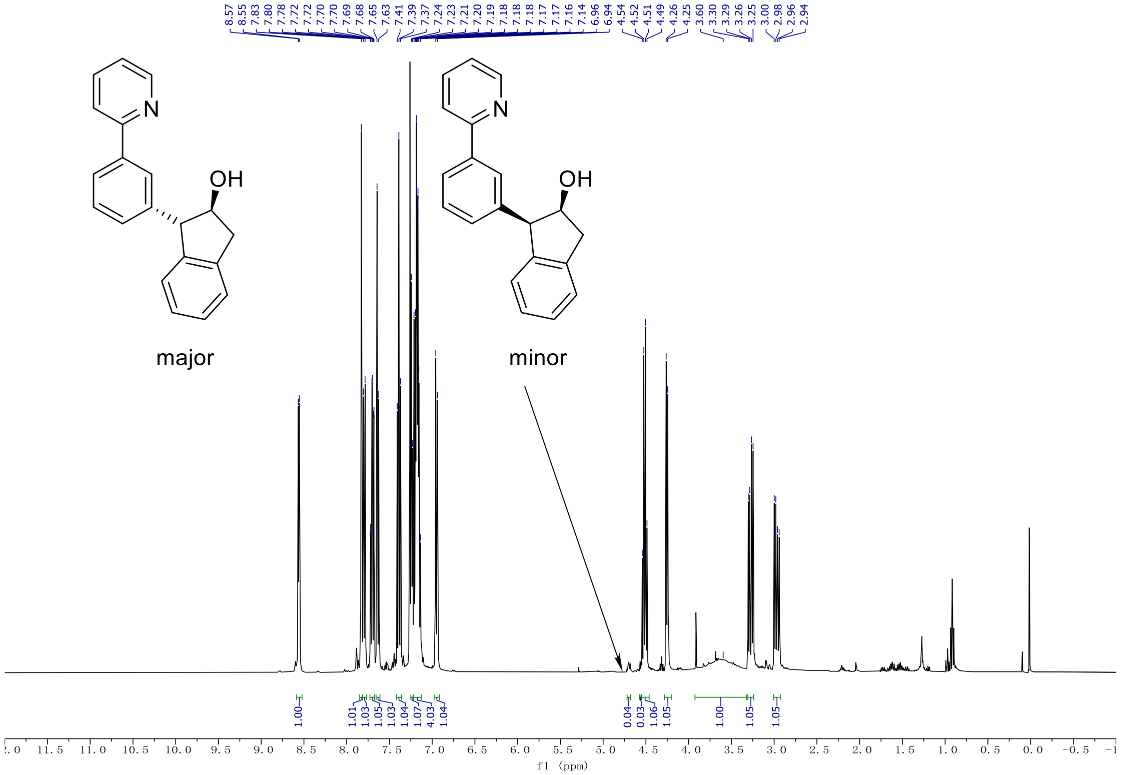

Supplementary Figure 133. ^{1}H NMR spectrum (400 MHz, $CDCl_3$, at rt) of **3ao**

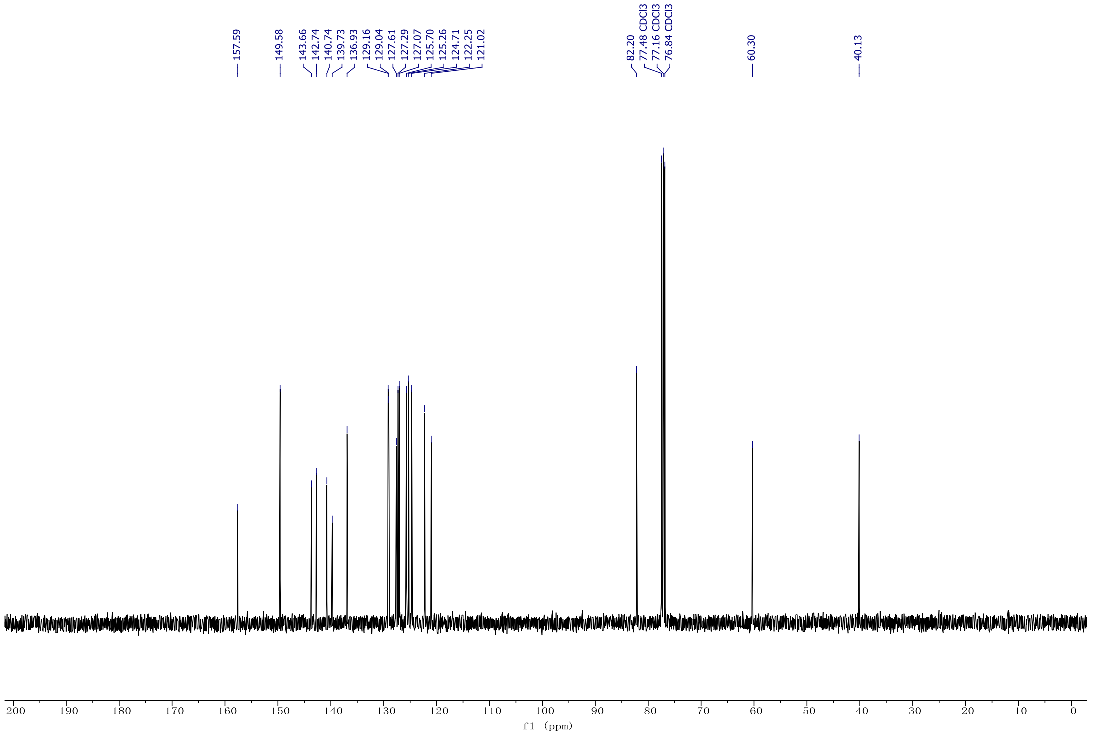

Supplementary Figure 134. ^{13}C NMR spectrum (101 MHz, $CDCl_3$, at rt) of **3ao**

7-Chloro-5-(3-(2-hydroxy-1-phenylethyl)phenyl)-1-methyl-1,3-dihydro-2*H*-benzo[e][1,4]diazepin-2-one **3ap** (two diastereomers mixed, 1:1 ratio)

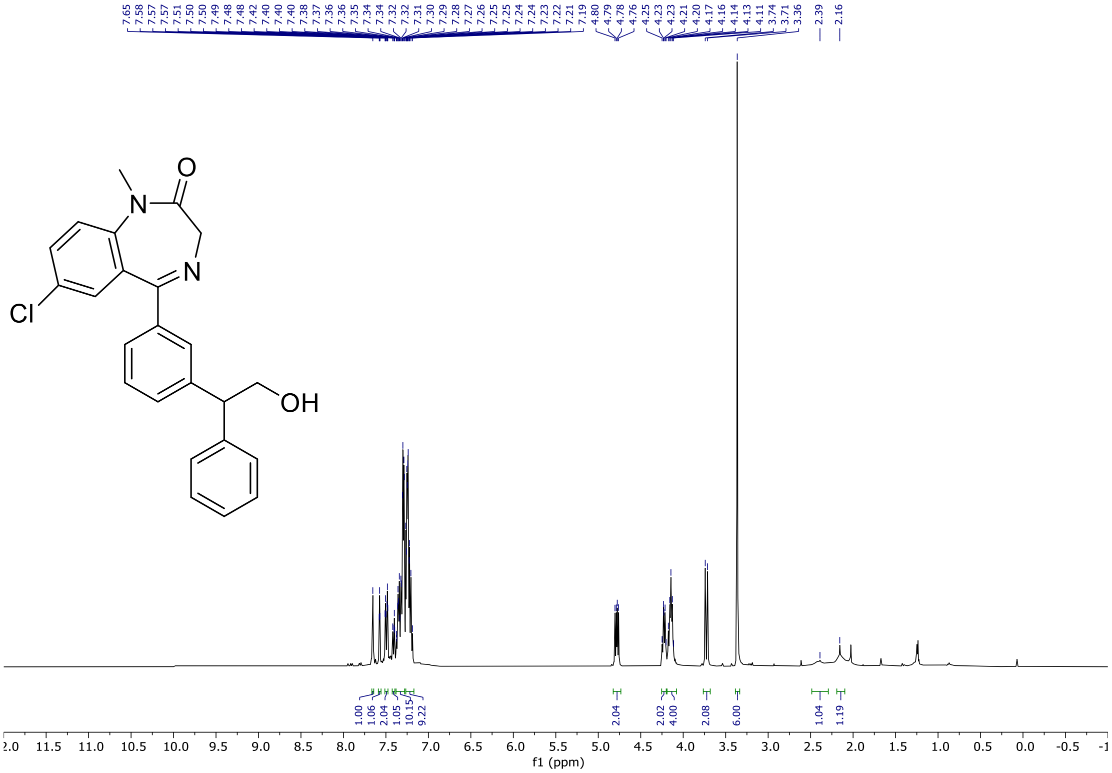

Supplementary Figure 135. ^{1}H NMR spectrum (400 MHz, $CDCl_3$, at rt) of **3ap**

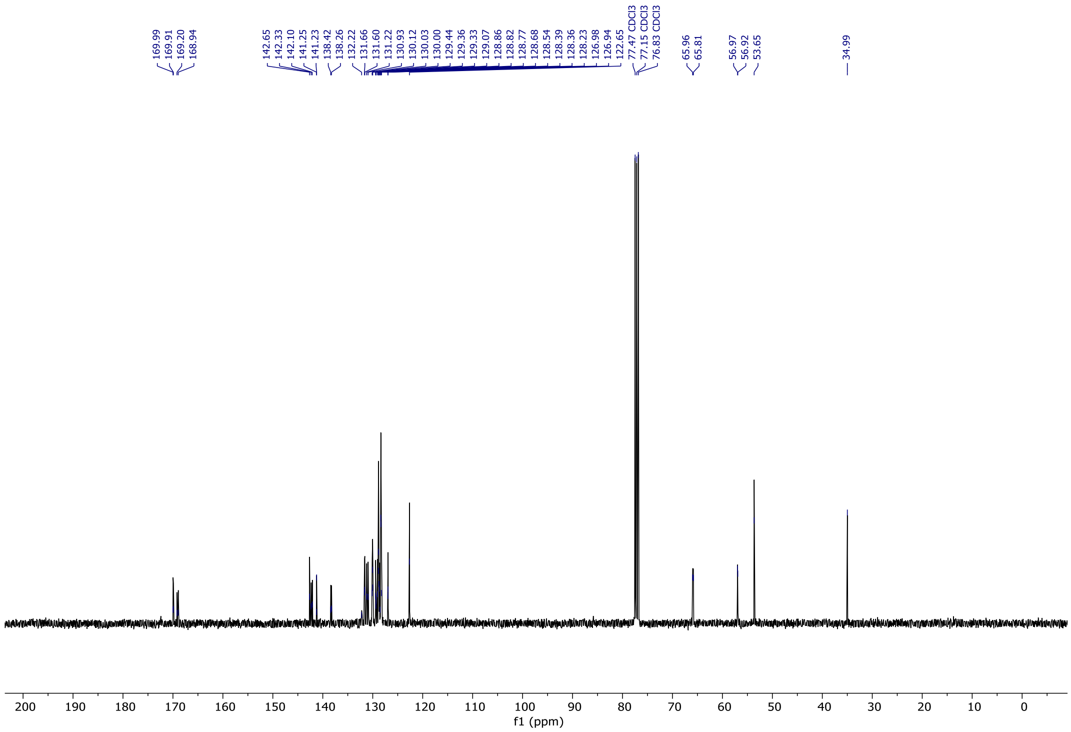

Supplementary Figure 136. ^{13}C NMR spectrum (101 MHz, $CDCl_3$, at rt) of **3ap**

Estrone derivates **3aq** (major + minor diastereomers)

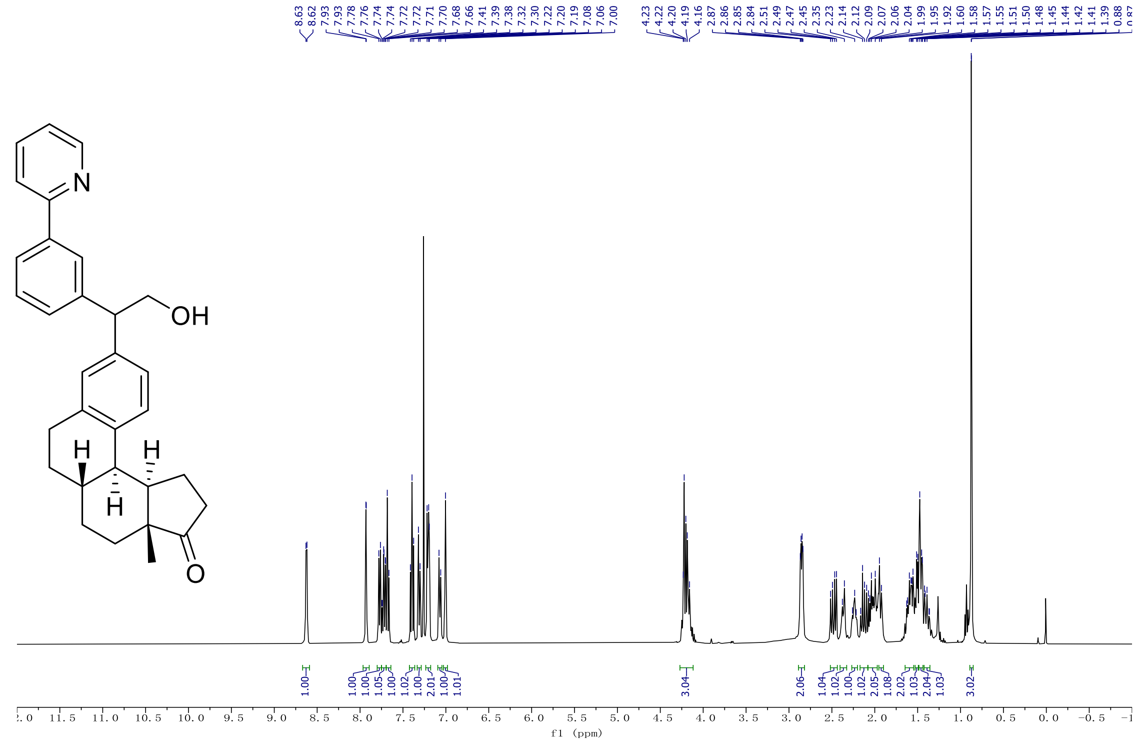

Supplementary Figure 137. ^{1}H NMR spectrum (400 MHz, $CDCl_3$, at rt) of **3aq**

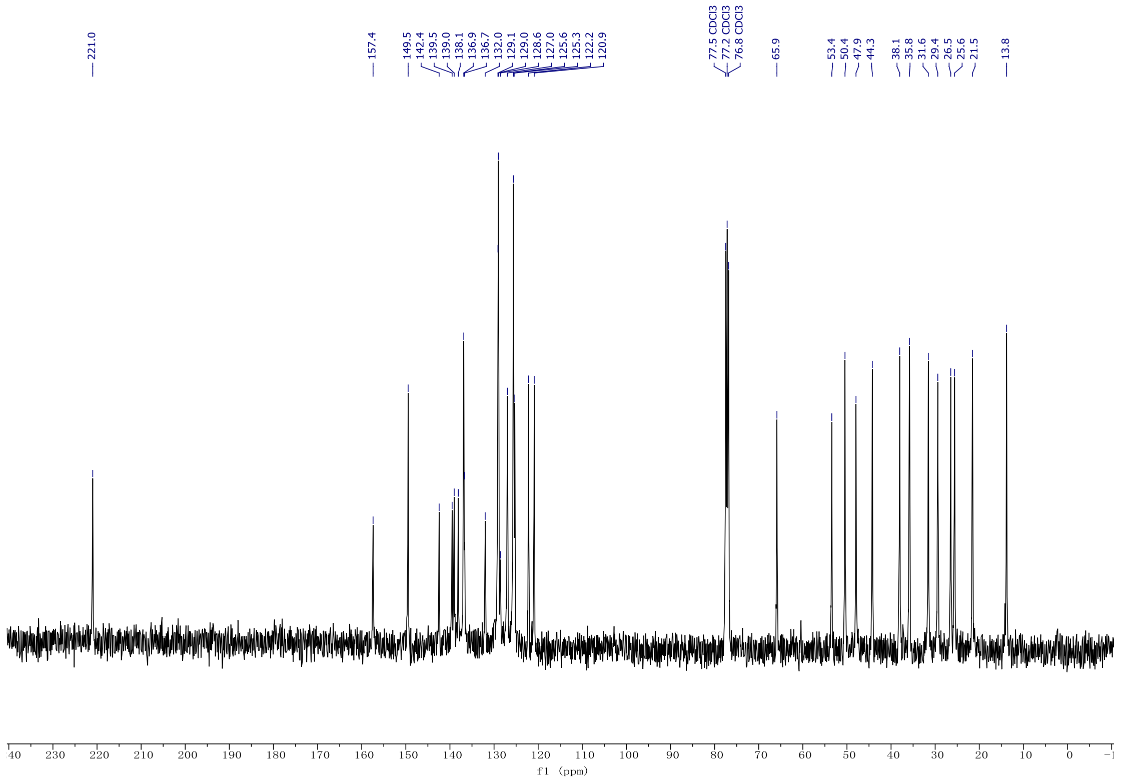

Supplementary Figure 138. ^{13}C NMR spectrum (101 MHz, $CDCl_3$, at rt) of **3aq**

Adapalene derivates **3ar**

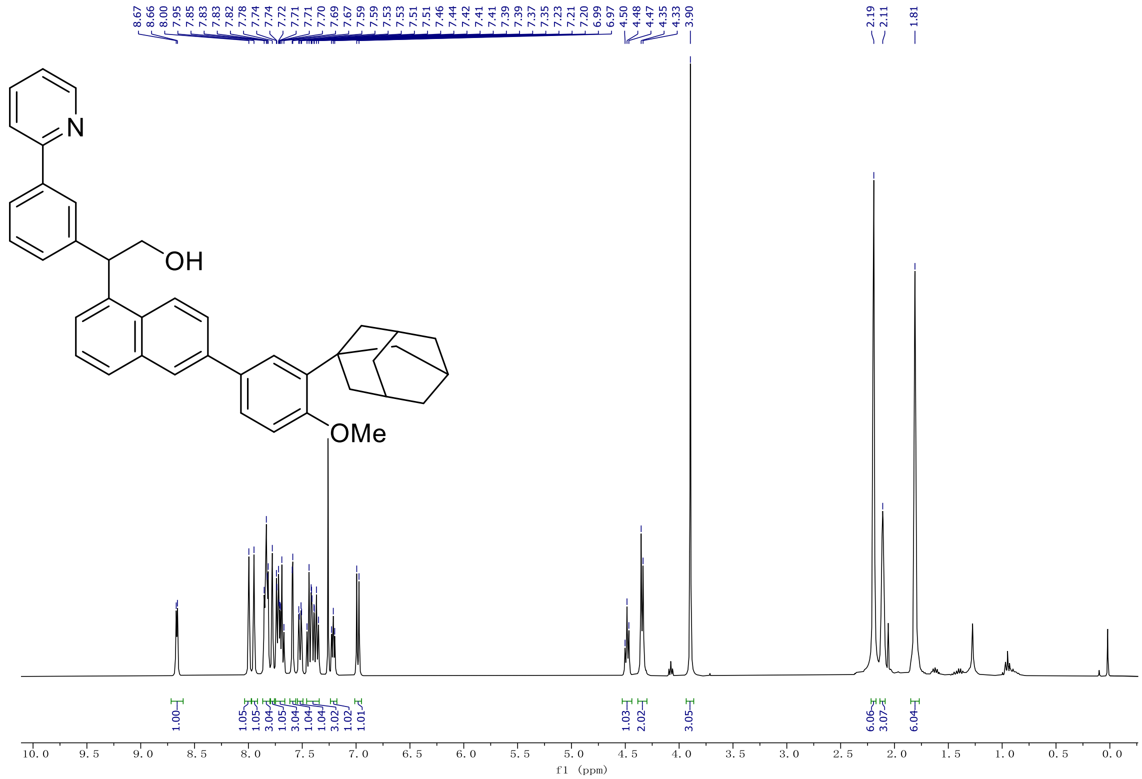

Supplementary Figure 139. ^{1}H NMR spectrum (400 MHz, $CDCl_3$, at rt) of **3ar**

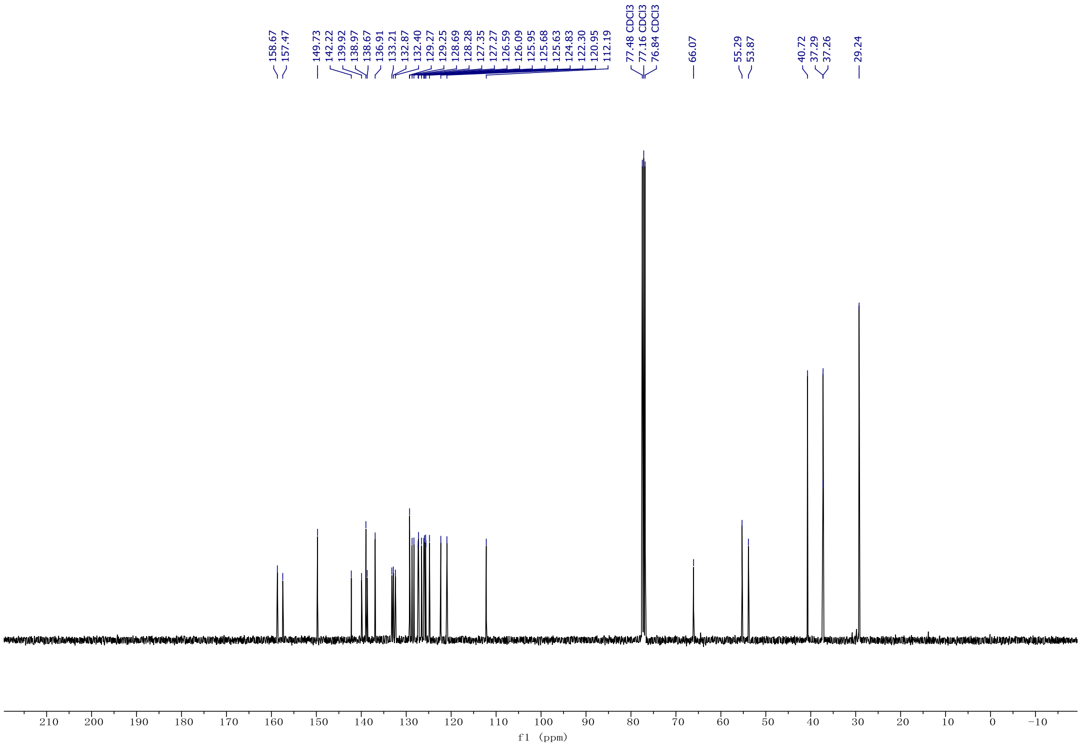

Supplementary Figure 140. ^{13}C NMR spectrum (101 MHz, $CDCl_3$, at rt) of **3ar**

Telmisartan derivates **3as**

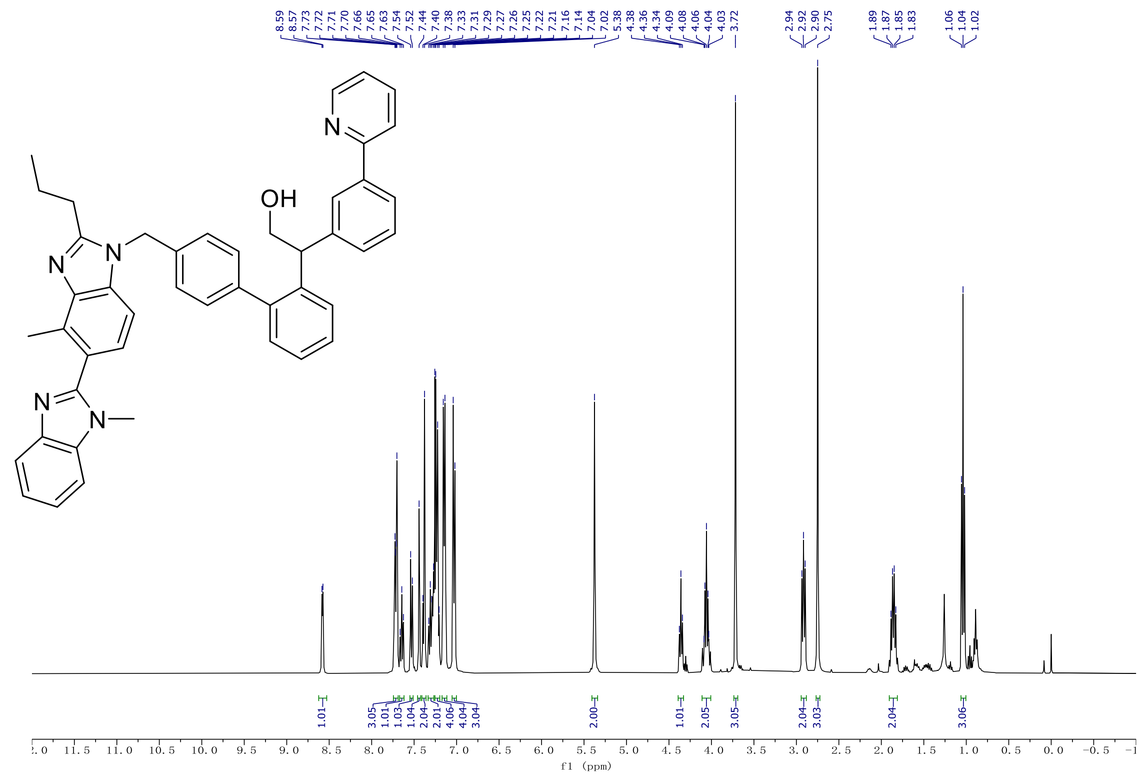

Supplementary Figure 141. ^{1}H NMR spectrum (400 MHz, $CDCl_3$, at rt) of **3as**

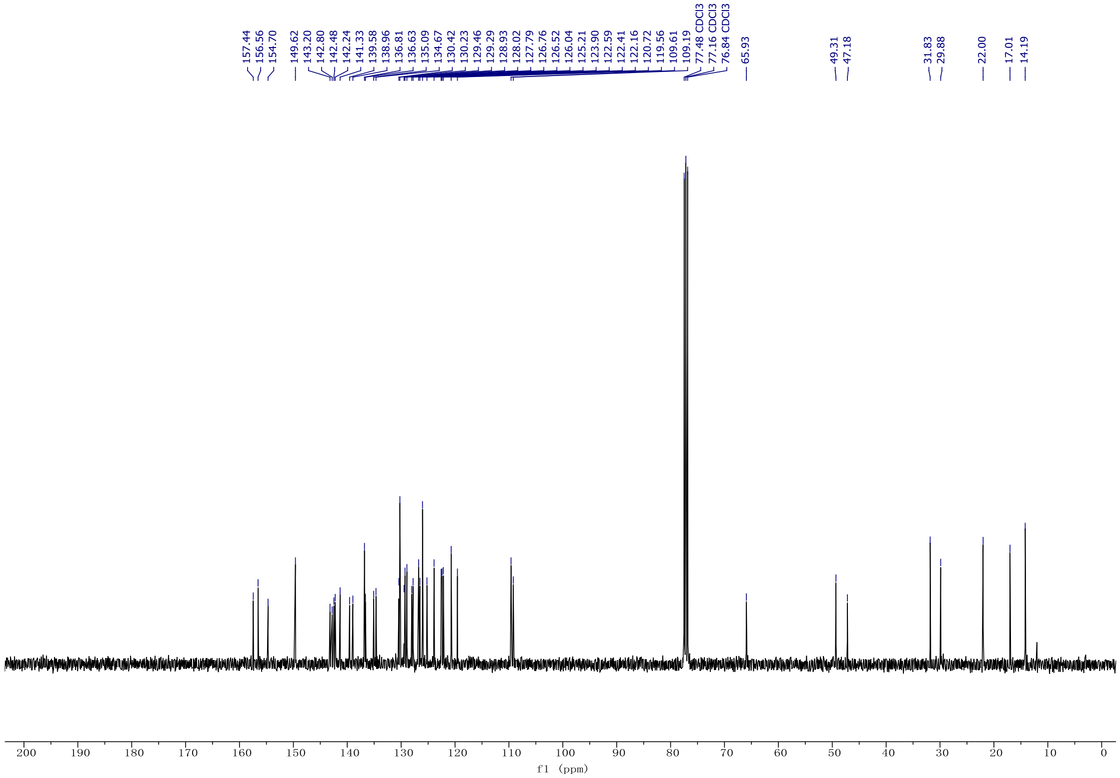

Supplementary Figure 142. ^{13}C NMR spectrum (101 MHz, $CDCl_3$, at rt) of **3as**

3-Phenyl-3-(3-(pyridin-2-yl)phenyl)propan-1-ol **5a**

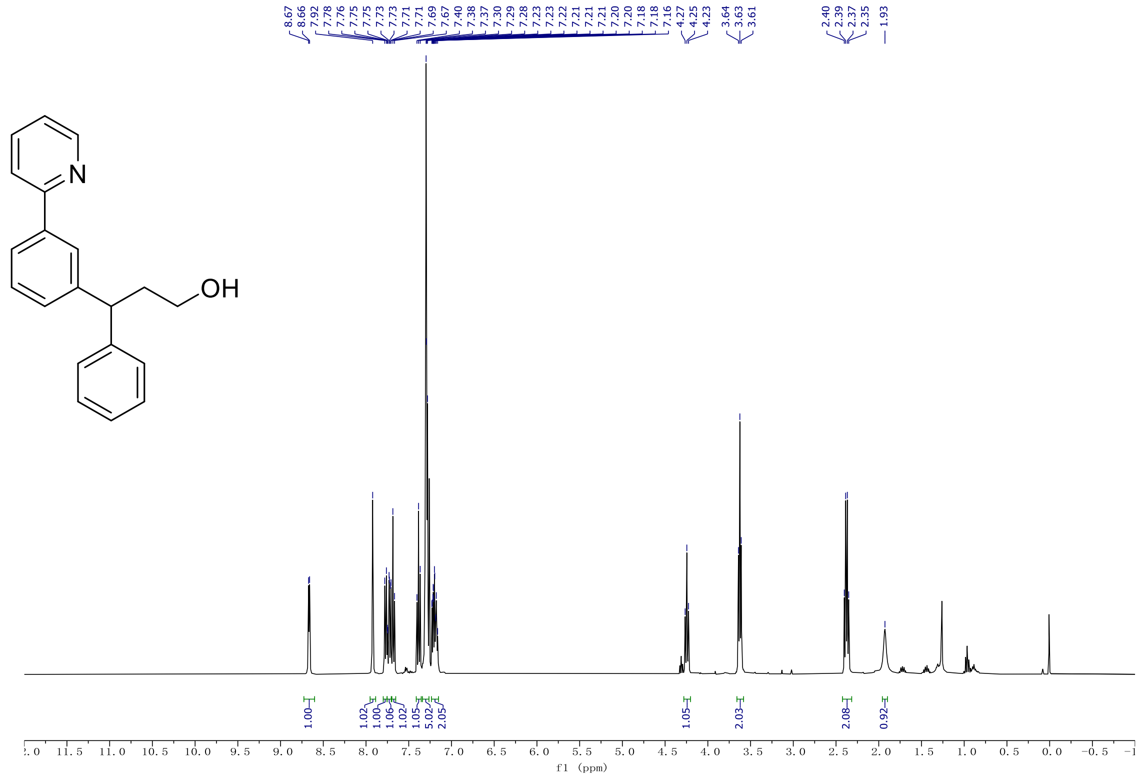

Supplementary Figure 143. ^{1}H NMR spectrum (400 MHz, $CDCl_3$, at rt) of **5a**

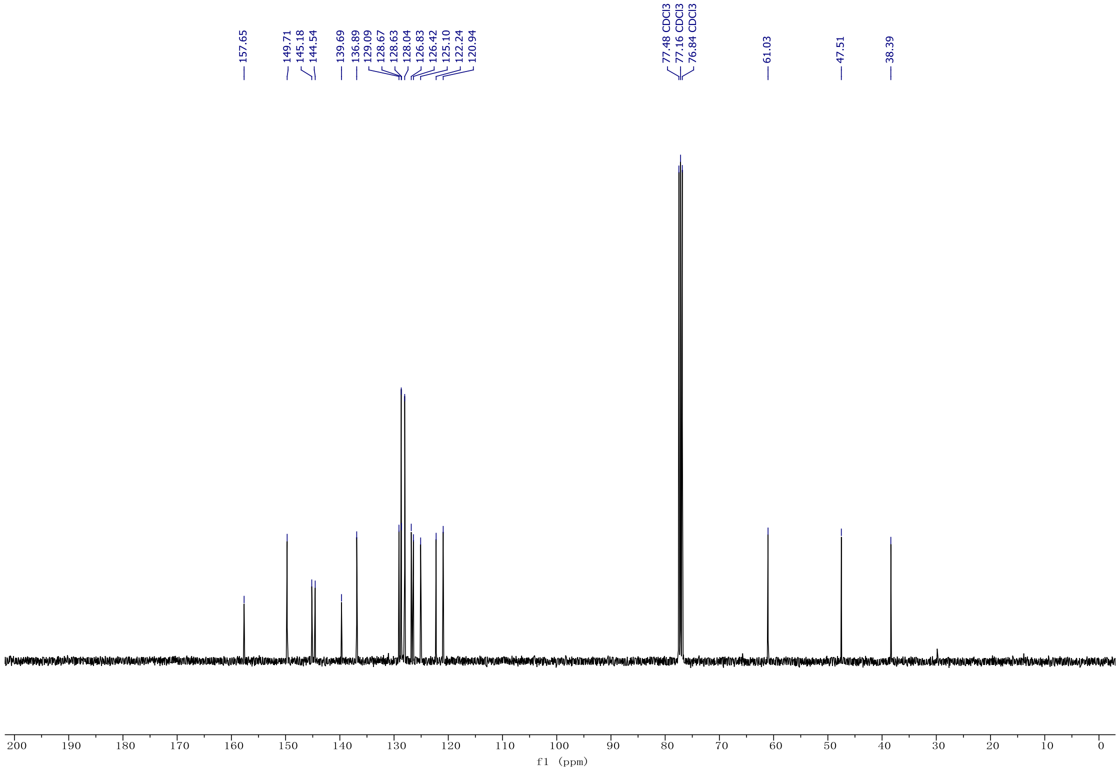

Supplementary Figure 144. ^{13}C NMR spectrum (101 MHz, $CDCl_3$, at rt) of **5a**

Methyl 2-(3-hydroxy-1-phenylpropyl)-4-(pyridin-2-yl)benzoate **5b**

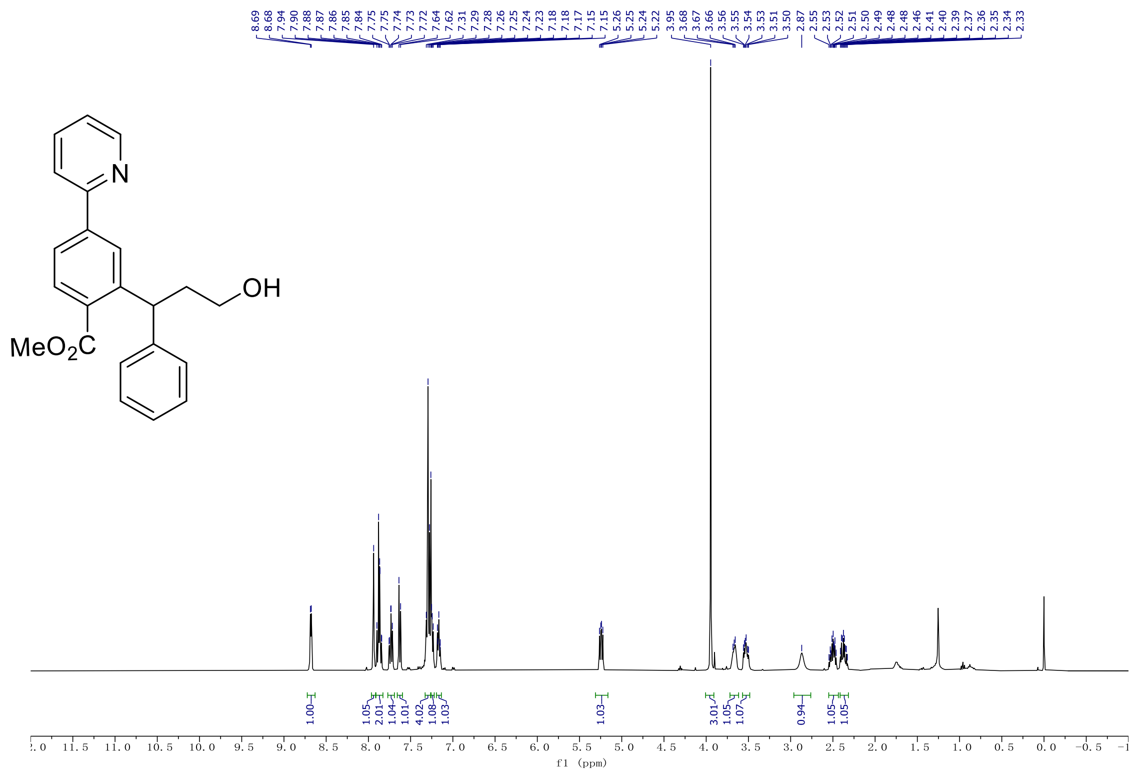

Supplementary Figure 145. ^{1}H NMR spectrum (101 MHz, $CDCl_3$, at rt) of **5b**

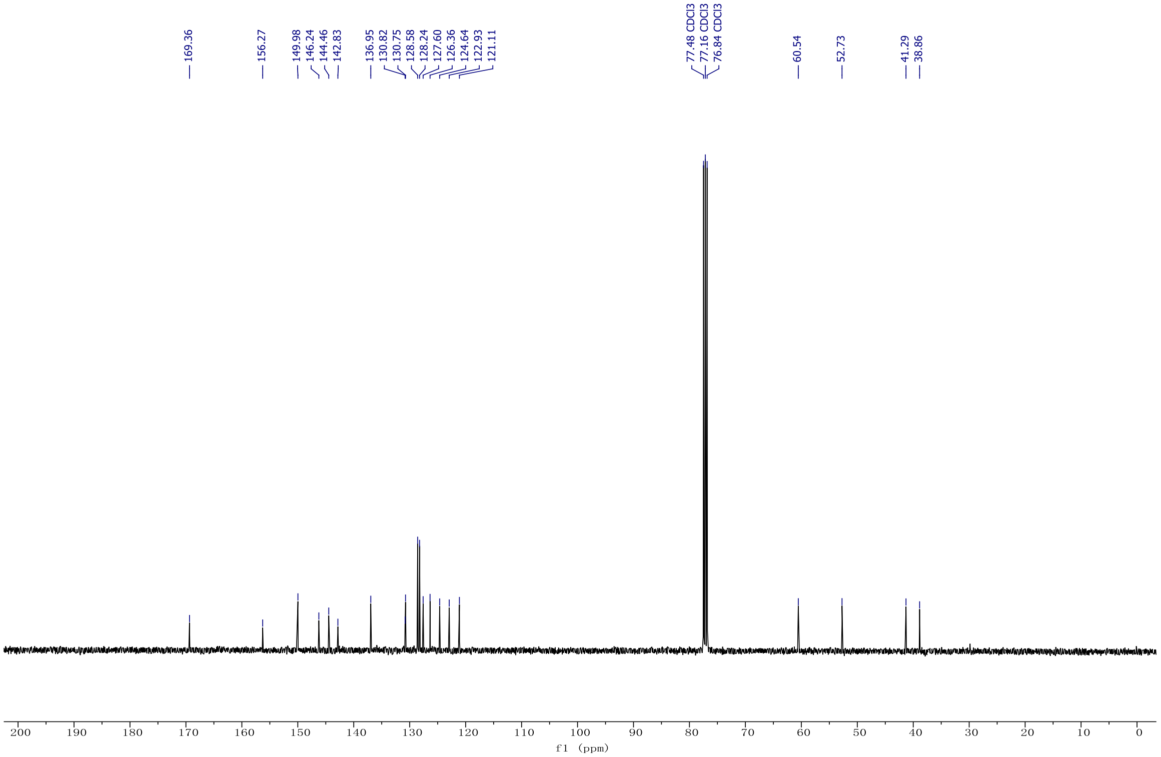

Supplementary Figure 146. ^{13}C NMR spectrum (101 MHz, $CDCl_3$, at rt) of **5b**

3-([1,1'-Biphenyl]-4-yl)-3-(3-(pyridin-2-yl)phenyl)propan-1-ol **5c**

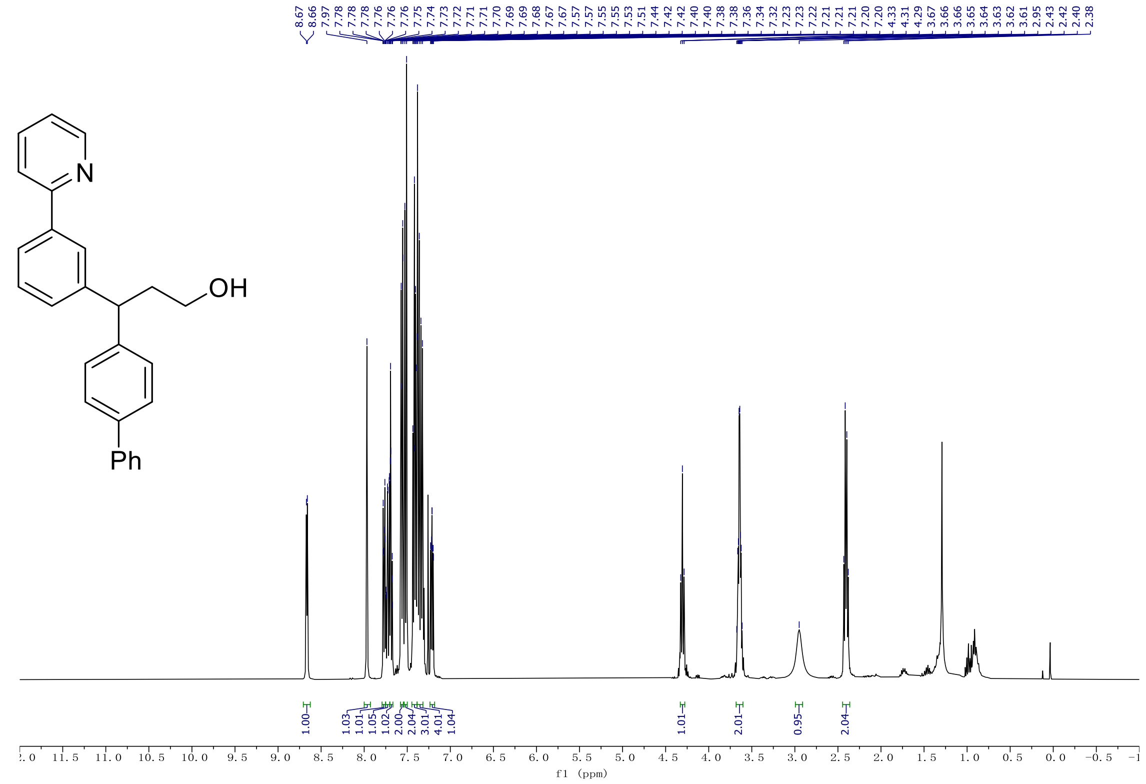

Supplementary Figure 147. ^{1}H NMR spectrum (400 MHz, $CDCl_3$, at rt) of **5c**

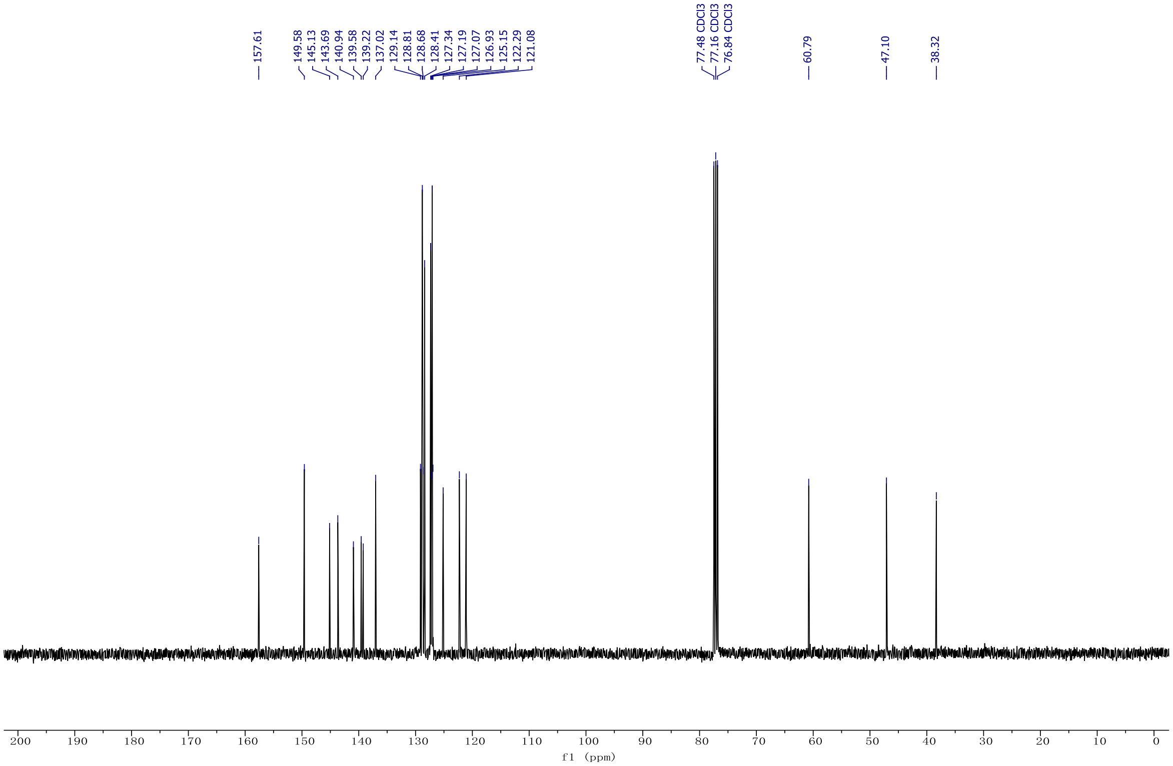

Supplementary Figure 148. ^{13}C NMR spectrum (101 MHz, $CDCl_3$, at rt) of **5c**

3-(4-Fluorophenyl)-3-(3-(pyridin-2-yl)phenyl)propan-1-ol **5d**

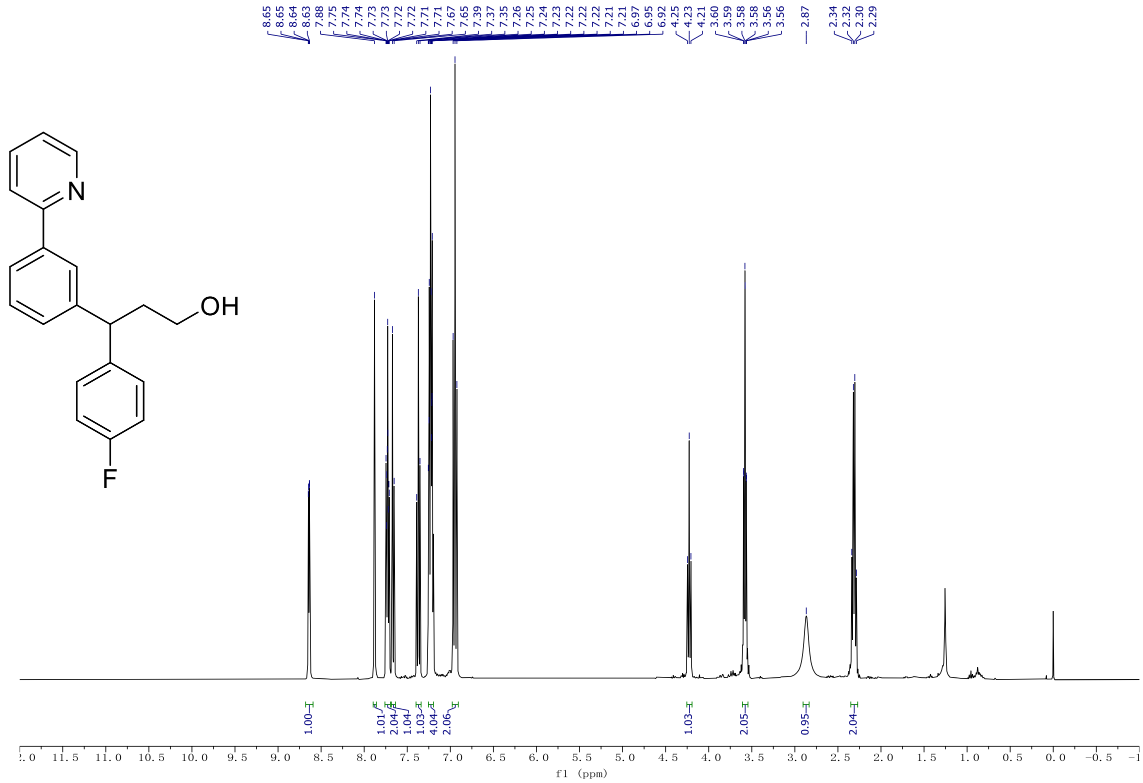

Supplementary Figure 149. ^{1}H NMR spectrum (400 MHz, $CDCl_3$, at rt) of **5d**

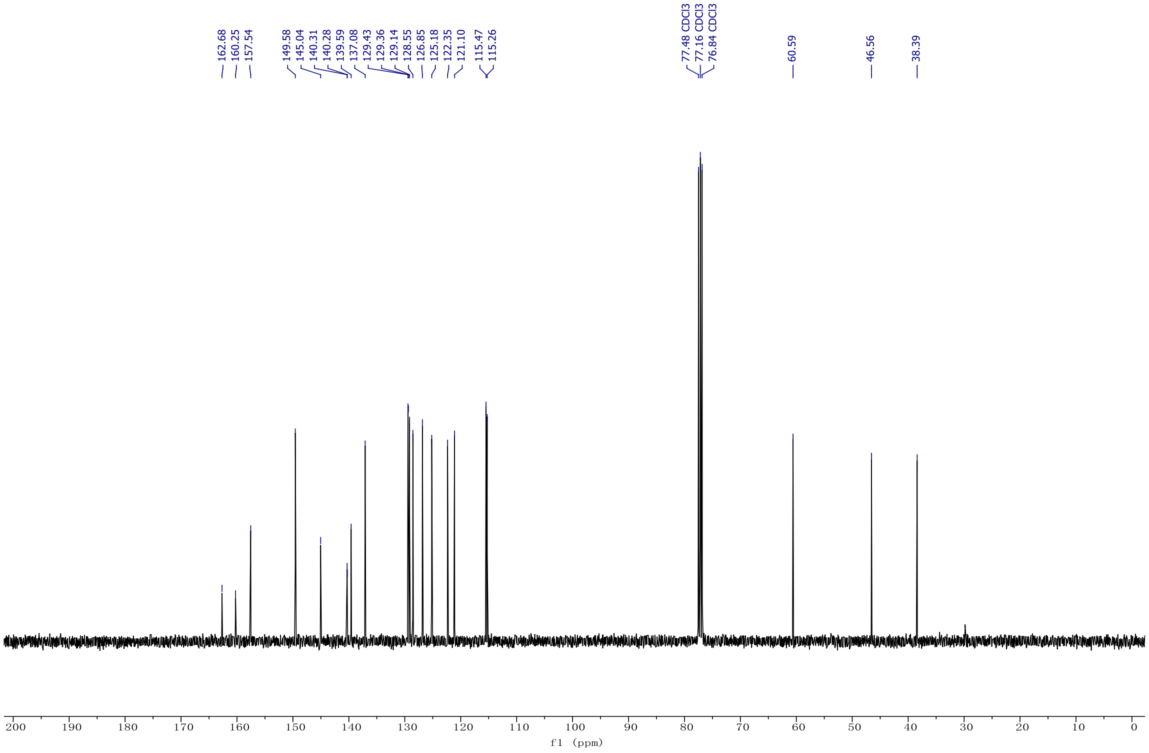

Supplementary Figure 150. ^{13}C NMR spectrum (101 MHz, $CDCl_3$, at rt) of **5d**

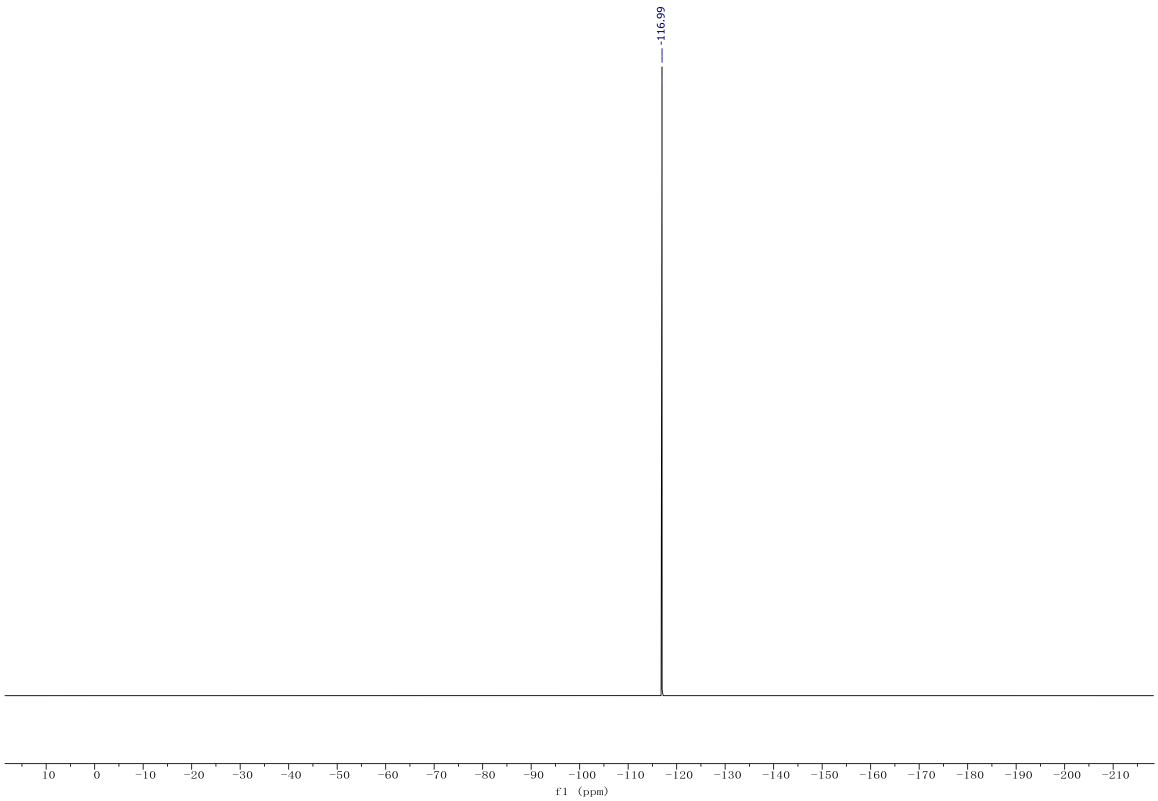

Supplementary Figure 151. ^{19}F NMR spectrum (376 MHz, $CDCl_3$, at rt) of **5d**

3-(4-Chlorophenyl)-3-(3-(pyridin-2-yl)phenyl)propan-1-ol **5e**

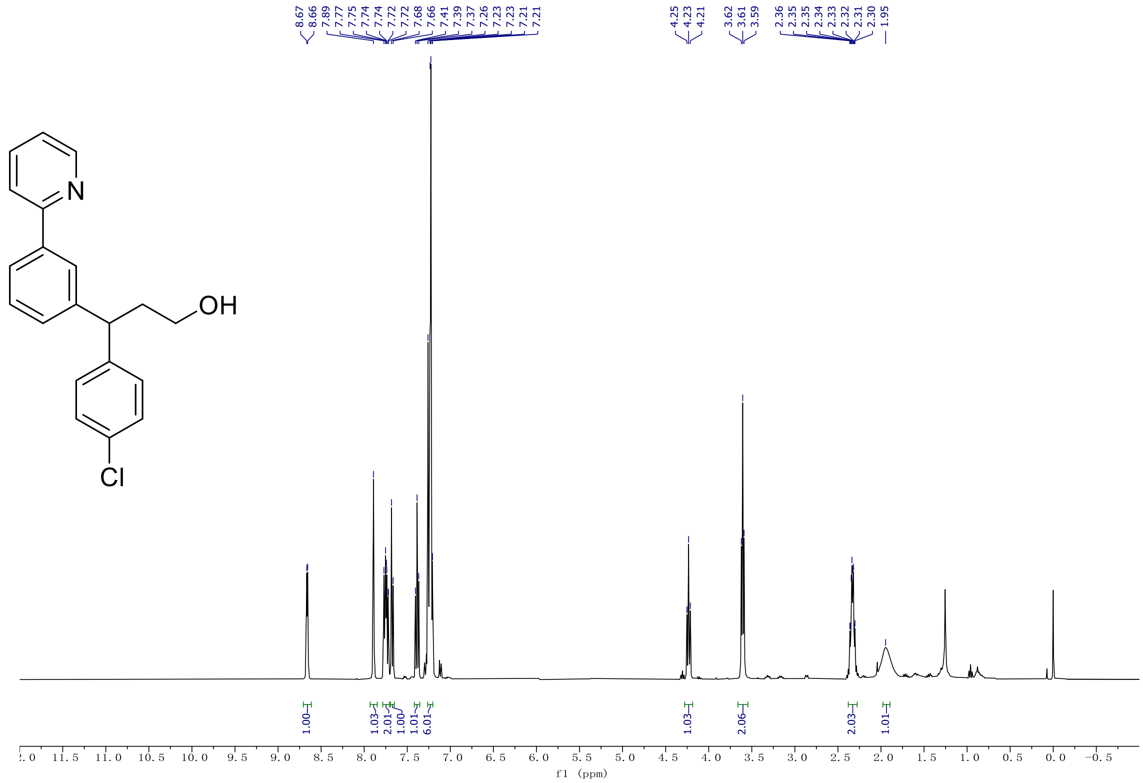

Supplementary Figure 152. ^{1}H NMR spectrum (400 MHz, $CDCl_3$, at rt) of **5e**

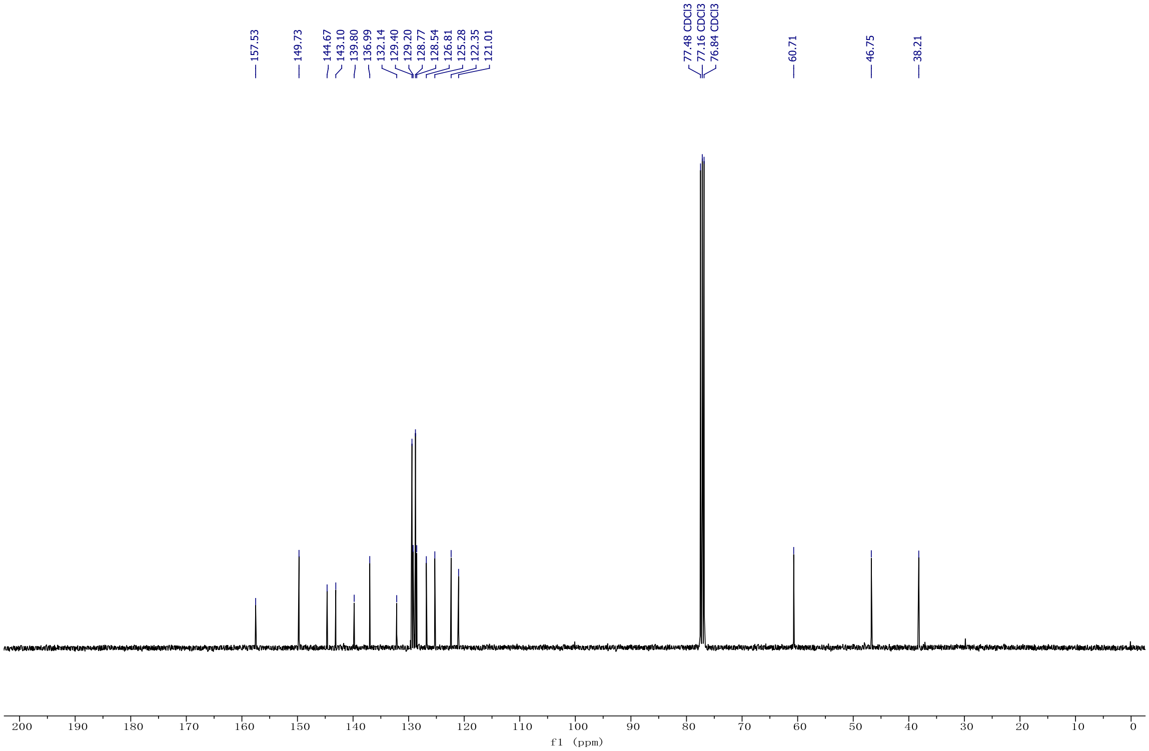

Supplementary Figure 153. ^{13}C NMR spectrum (101 MHz, $CDCl_3$, at rt) of **5e**

3-(4-Bromophenyl)-3-(3-(pyridin-2-yl)phenyl)propan-1-ol **5f**

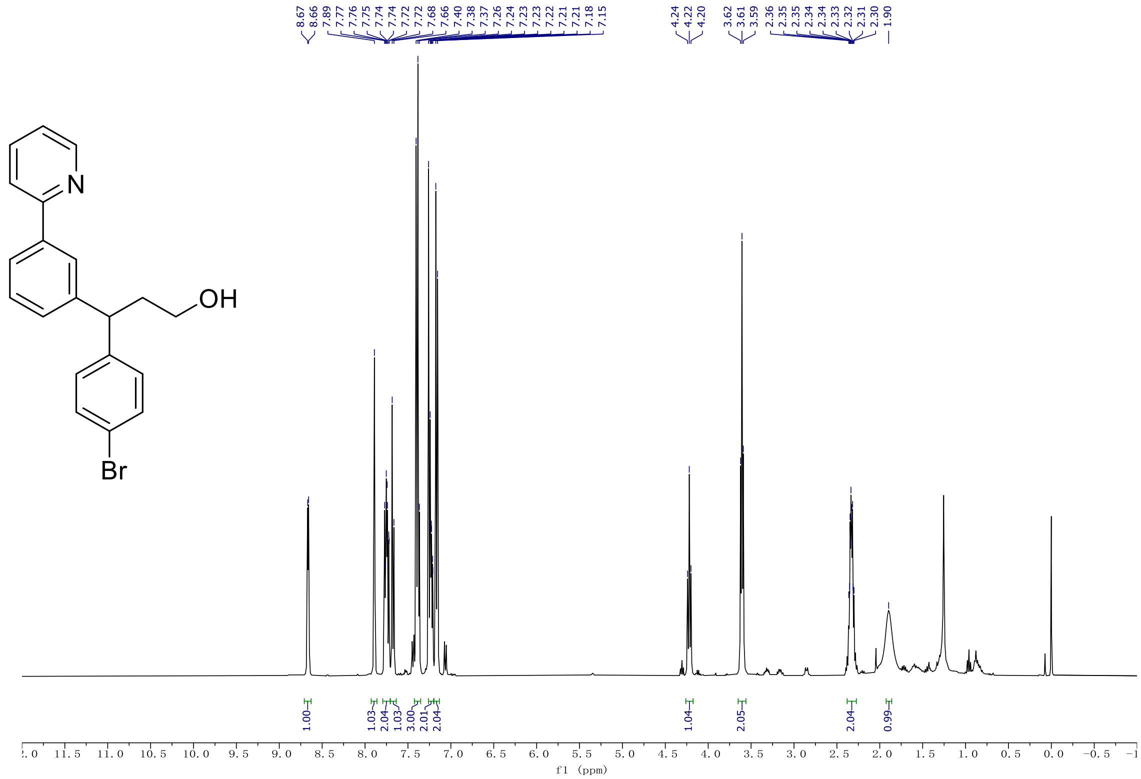

Supplementary Figure 154. ^{1}H NMR spectrum (400 MHz, $CDCl_3$, at rt) of **5f**

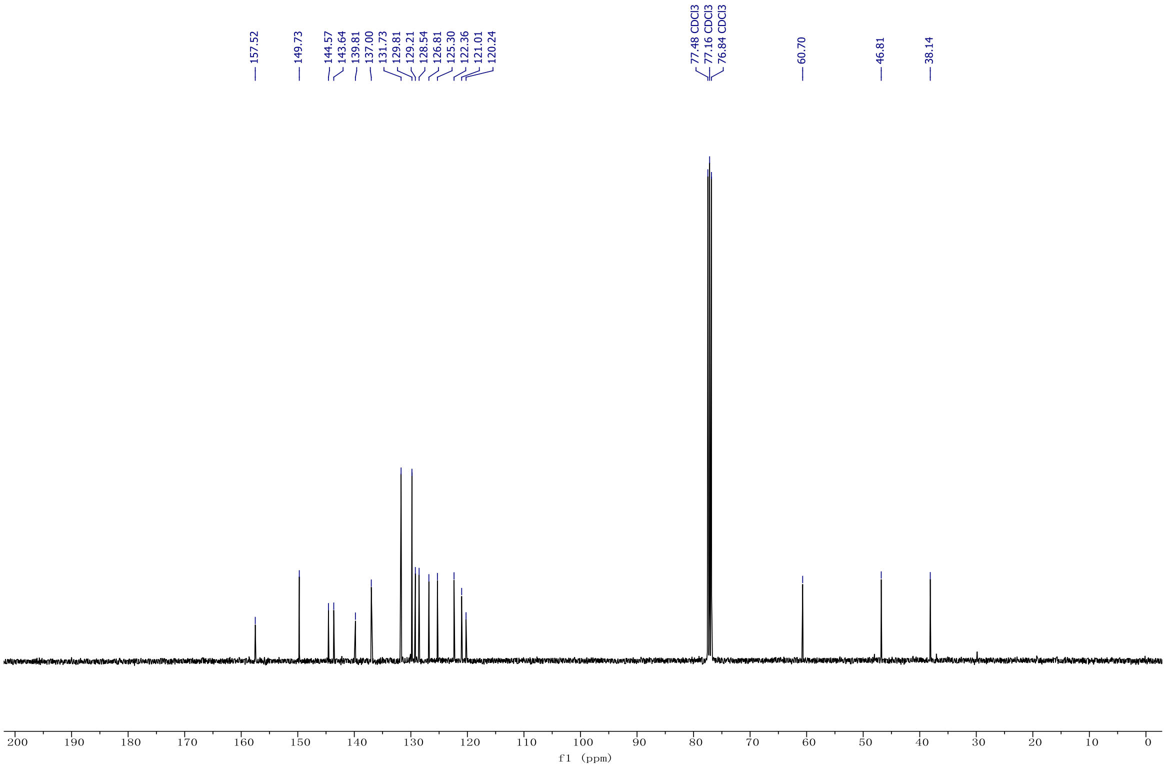

Supplementary Figure 155. ^{13}C NMR spectrum (101 MHz, $CDCl_3$, at rt) of **5f**

3-(3-(Pyridin-2-yl)phenyl)-3-(p-tolyl)propan-1-ol **5g**

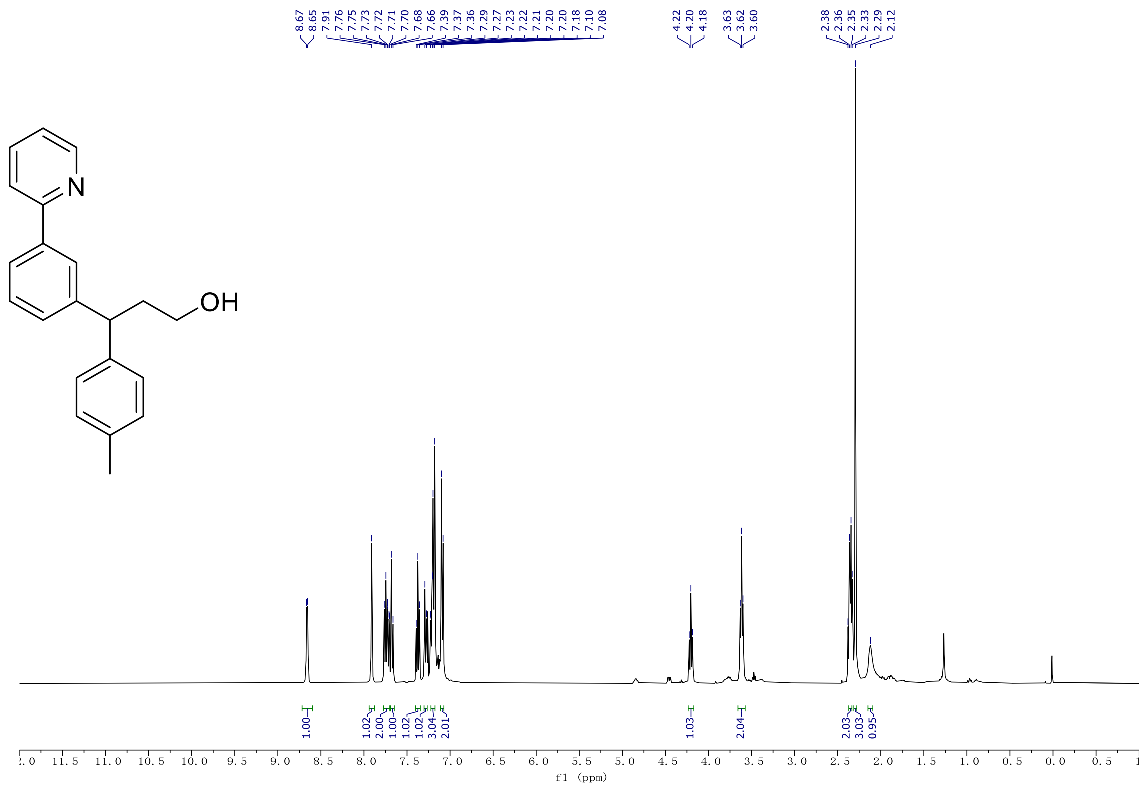

Supplementary Figure 156. ^{1}H NMR spectrum (400 MHz, $CDCl_3$, at rt) of **5g**

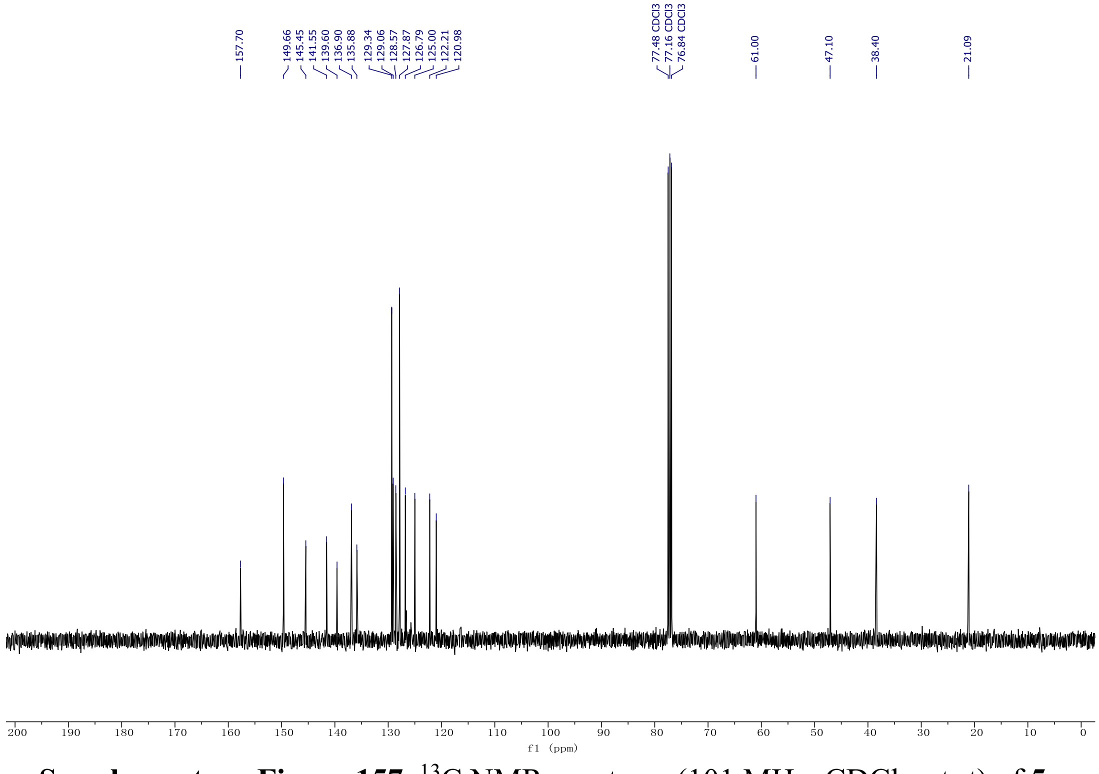

Supplementary Figure 157. ^{13}C NMR spectrum (101 MHz, $CDCl_3$, at rt) of **5g**

2-Hydroxy-1-phenylethyl 2-ethylbutanoate **8**

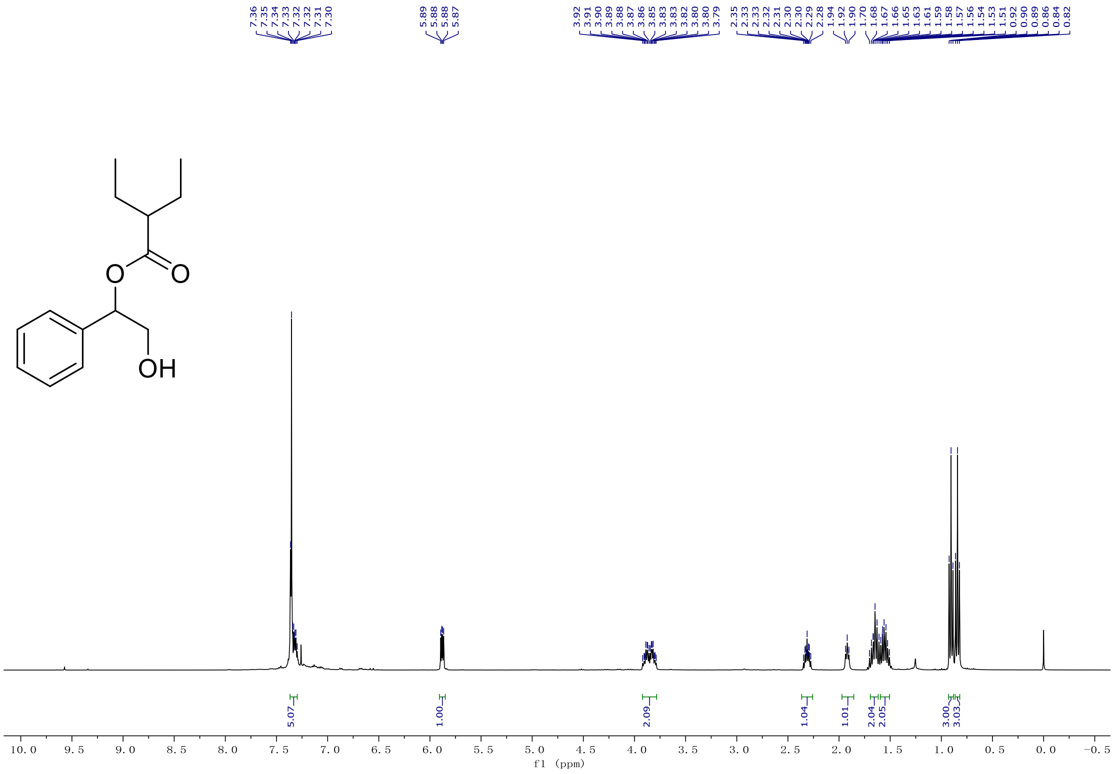

Supplementary Figure 158. ^{1}H NMR spectrum (400 MHz, $CDCl_3$, at rt) of **8**

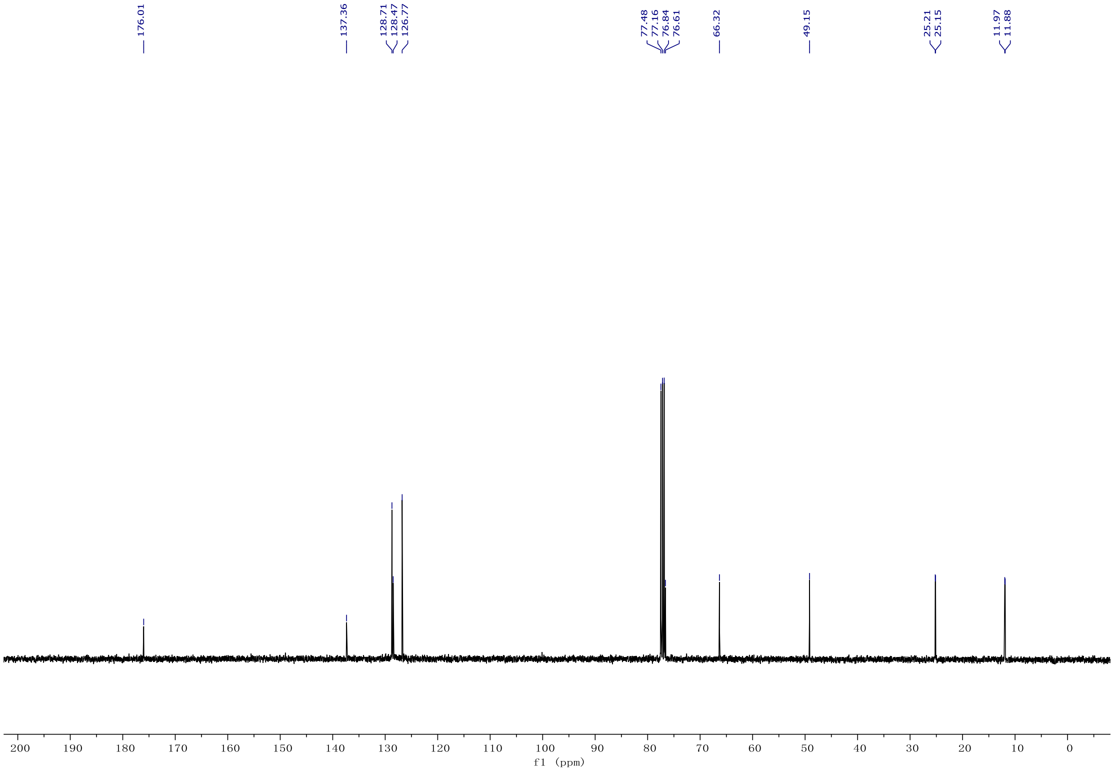

Supplementary Figure 159. ^{13}C NMR spectrum (101 MHz, $CDCl_3$, at rt) of **8**

2-Hydroxy-2-phenylethyl 2-ethylbutanoate **9**

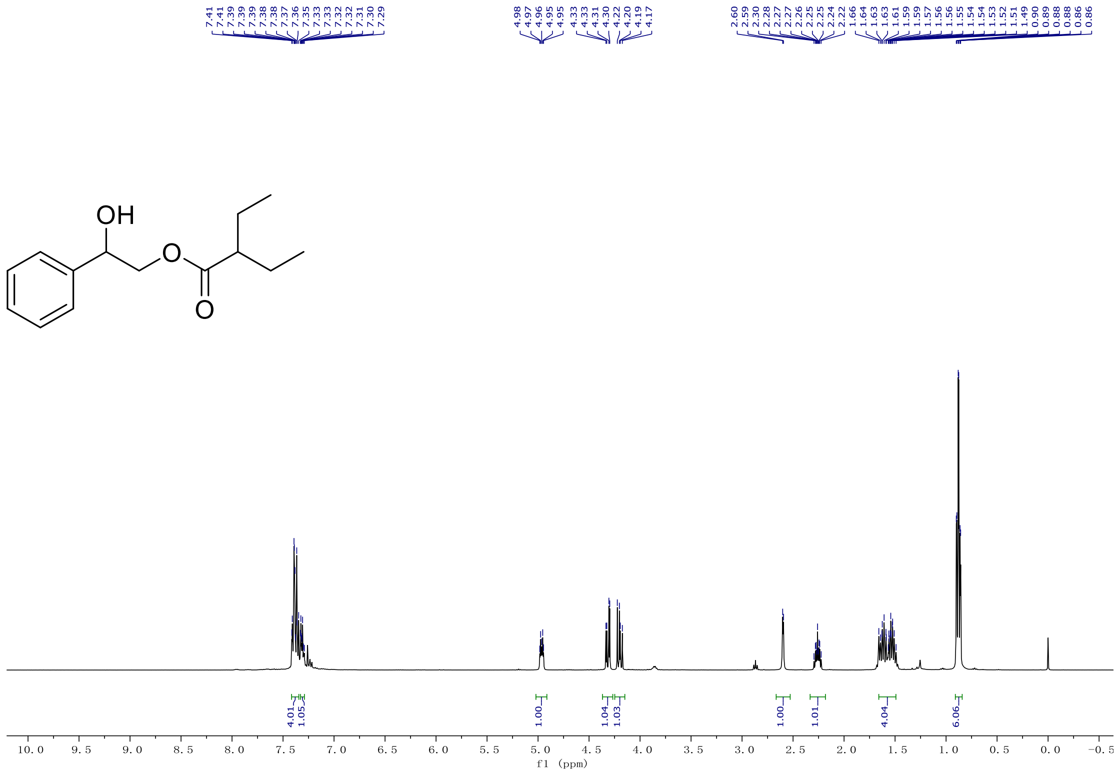

Supplementary Figure 160. ^{1}H NMR spectrum (400 MHz, $CDCl_3$, at rt) of **9**

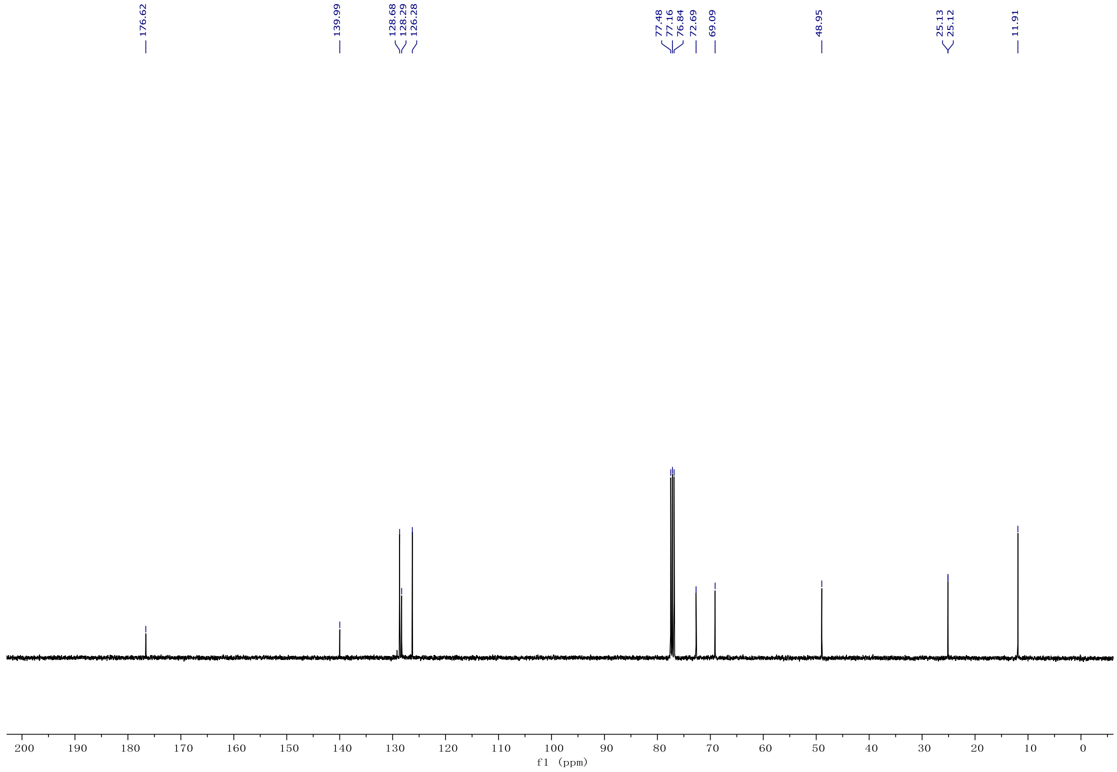

Supplementary Figure 161. ^{13}C NMR spectrum (101 MHz, $CDCl_3$, at rt) of **9**

IV. Supplementary References

1. Kim, J. Y. et al. Rhodium-catalyzed intermolecular amidation of arenes with sulfonyl azides via chelation-assisted C–H bond activation. *J. Am. Chem. Soc.* **134**, 9110-91113 (2012).

2. Kim, H., Park, J., Kim, J. G. & Chang, S. Synthesis of phosphoramidates: a facile approach based on the C–N bond formation via Ir-catalyzed direct C–H amidation. *Org. Lett.* **16**, 5466–5469 (2014).

3. Eisele, P., Ullwer, F., Scholz, S. & Plietker, B. Mild, selective Ru-catalyzed deuteration using D_2O as a deuterium source. *Chem. Eur. J.* **25**, 16550–16554 (2019).

4. Yang, M. S. Y. et al. Gold-catalyzed C(sp^2)-C(sp) coupling by alkynylation through oxidative addition of bromoalkynes. *Chem. Eur. J.* **25**, 9624 –9628 (2019).

5. Zhang, S.-S. et al. Access to branched allylarenes via rhodium (III)-catalyzed C–H allylation of (hetero)arenes with 2-methylidenetrimethylene carbonate. *Org. Lett.* **23**, 5719–5723 (2021).

6. Nielsen, D. K. & Doyle, A. G. Nickel-catalyzed cross-coupling of styrenyl epoxides with boronic acids. *Angew. Chem. Int. Ed.* **50**, 6056 –6059 (2011).

7. Qu, J. et al. Nickel-catalyzed cross-coupling of epoxides with aryltriflates: rapid and regioselective construction of aryl ketones. *Chem. Commun.* **58**, 9214–9217 (2022).

8. Tyagi, A., Khan, J., Yadav, N., Mahato, R. & Hazra, C. K. Catalyst-switchable divergent synthesis of bis(indolyl)alkanes and 3-alkylated indoles from styrene oxides. *J. Org. Chem.* **87**, 10229−10240 (2022).

9. Wenz, J., Wadepohl, H. & Gade, L. H. Regioselective hydrosilylation of epoxides catalysed by nickel(ii) hydrido complexes. *Chem. Commun.* **53**, 4308–4311 (2017).

10. Liu, Z.-P., Meng, Z.-L. & Wang, D.-F. Synthesis of novel nucleoside analogues based on 1,3-dihydrobenzo[c]furan core. *Chin. J. Chem.* **24**, 504-508 (2006).

11. Han, D. et al. Asymmetric epoxidation of unfunctionalized olefins catalyzed by chiral (pyrrolidine salen) Mn (III) complexes with proline sidearms. *Molecular Catalysis* **524**, 112268 (2022).

12. Zhou, Z., Dai, G., Ru, S., Yu, H. & Wei, Y. Highly selective and efficient olefin epoxidation with pure inorganic-ligand supported iron catalysts. *Dalton Trans.* **48**, 14201-14205 (2019).

13. Guo, Z. et al. Kinetic resolution of N-aryl β-amino alcohols via asymmetric aminations of anilines. *Chem. Commun.* **57**, 9394–9397 (2021).

14. Rayati, S. et al. A manganese (iii) Schiff base complex immobilized on silica-coated magnetic nanoparticles showing enhanced electrochemical catalytic performance toward sulfide and alkene oxidation. *RSC Adv.* **10**, 17026–17036 (2020).

15. Maltais, R. et al. Discovery of a non-estrogenic irreversible inhibitor of 17 β-Hydroxysteroid dehydrogenase type from 3-substituted-16β-(m-carbamoylbenzyl)-estradiol derivatives. *J. Med. Chem.* **57**, 204−222 (2014).

16. Loman, J. J. et al. A combined computational and experimental investigation of the oxidative ring-opening of cyclic ethers by oxoammonium cations. *Org. Biomol. Chem.* **14***,* 3883–3888 (2016).

17. Griesbeck, A. G. & Sokolova, T. Product class 3: oxetanes and oxetan-3-ones. *Science of Synthesis* **37***,* 433-471 (2008).

18. Vitale, P. et al. Stereoselective chemoenzymatic synthesis of optically active aryl-substituted oxygen-containing heterocycles. *Catalysts* **7***,* 37-39 (2017).

19. Bertolini, F., Crotti, S., Bussolo, V. D., Macchia, F. & Pineschi, M. Regio- and stereoselective ring opening of enantiomerically enriched 2-aryl oxetanes and 2-aryl azetidines with aryl borates. *J. Org. Chem.* **73***,* 8998-9007 (2008).

20. Gao, K.; Yoshikai, N. Regioselectivity-switchable hydro-arylation of styrenes. *J. Am. Chem. Soc.* **133**, 400−402 (2011).

21. Arnold, D. R., Fahie, B. J., Lamont, L. J., Wierzchowski, J. & Young, K. M. 1, *n*-Radical ions. the photosensitized (electron transfer) formation of 1,5-radical cations. *Can. J. Chem.* **65**, 2734 (1987).

22. Zheng, Y. et al. Oxoammonium salt-mediated regioselective vicinal dioxidation of alkenes: relying on transient and persistent nitroxides. *Org. Lett.* **23***,* 8533−8538 (2021).

23. Lau, S. H. et al. Ni/photoredox-catalyzed enantioselective cross-electrophile coupling of styrene oxides with aryl iodides. *J. Am. Chem. Soc.* **143***,* 15873−15881 (2021).

24. Xiong, B. et al. Metal-free, N-iodosuccinimide-induced regioselective iodophosphoryloxylation of alkenes with P(O)−OH bonds. *Chem. Eur. J.* **26***,* 9556 –9560 (2020).

25. Kamal, A., Ramesh, G. & Laxman, N. New halogenation reagent system for one-pot conversion of alcohol into iodides and azides. *Synth. Commun*. **31**, 827–833 (2001).

26. Ahmed-Omera, B. & Sanderson. A. J. Preparation of fluoxetine by multiple flow processing steps. *Org. Biomol. Chem*. **9**, 3854-3862 (2011).

27. Leyva, Lida et al. Synthesis of cycloruthenated compounds as potential anticancer agents. *Eur. J. Inorg. Chem.* 3055–3066 (2007).

28. Lynam, J. M., Welby, C. E., Whitwood, A. C. Exploitation of a chemically non-innocent acetate ligand in the synthesis and reactivity of ruthenium vinylidene complexes. *Organometallics*, **28**, 1320–1328 (2009).

29. Andersen, R. A., Mainz, V. V. Preparation of $RuCH_2PMe_2(PMe_3)_3Cl$, $Ru(CH_2PMe_2)_2(PMe_3)_2$, and Rh2(CH2PMe2)2(PMe3)4 and their reactions with hydrogen. *Organometallics*, **3**, 675–678 (1984).
